# Resveratrol ameliorates glioblastoma inflammatory response by reducing NLRP3 inflammasome activation through inhibition of the JAK2/STAT3 pathway

**DOI:** 10.1007/s00432-024-05625-5

**Published:** 2024-03-28

**Authors:** Chao Zhang, Qian Peng, Yuhang Tang, Chengcheng Wang, Shuai Wang, Dong Yu, Shiqiang Hou, Yu Wang, Lanlan Zhang, Ning Lin

**Affiliations:** 1 Department of Neurosurgery, The First People’s Hospital of Chuzhou, The Affiliated Chuzhou Hospital of Anhui Medical University, 12 Zhongyou road, Chuzhou, 239001 China; 2 Hematology Department, The Second Affiliated Hospital of Anhui Medical University, Hefei, 230601 China; 3 Hematologic Diseases Research Center of Anhui Medical University, Hefei, 230601 China; 4 Department of Science and Education, The First People’s Hospital of Chuzhou, The Affiliated Chuzhou Hospital of Anhui Medical University, 12 Zhongyou road, Chuzhou, 239001 China

**Keywords:** Resveratrol, Glioblastoma, JAK2/STAT3 signaling, NLRP3 inflammasome

## Abstract

**Objectives:**

The aim of this study was to investigate the anti-tumor effect of resveratrol (RSV) on glioblastoma (GBM) and its specific mechanism in improving the inflammatory response of the tumor microenvironment. The tumor microenvironment of GBM is highly neuroinflammatory, inducing tumor immunosuppression. Therefore, ameliorating the inflammatory response is an important focus for anti-tumor research.

**Methods:**

The anti-tumor effect of RSV on GBM was demonstrated through in vitro cellular assays, including CCK-8, EdU, PI staining, Transwell, wound healing assay, and flow cytometry. Potential mechanisms of RSV's anti-GBM effects were identified through network pharmacological analysis. In addition, the relationship of RSV with the JAK2/STAT3 signaling pathway and the inflammasome NLRP3 was verified using Western blot.

**Results:**

RSV significantly inhibited cell viability in GBM cell lines LN-229 and U87-MG. Furthermore, it inhibited the proliferation and invasive migration ability of GBM cells, while promoting apoptosis. Network pharmacological analysis revealed a close association between the anti-GBM effects of RSV and the JAK/STAT signaling pathway, as well as inflammatory responses. Western blot analysis confirmed that RSV inhibited the over-activation of the inflammasome NLRP3 through the JAK2/STAT3 signaling pathway. Partial reversal of RSV's inhibition of inflammasome NLRP3 was observed with the addition of the JAK/STAT agonist RO8191.

**Conclusions:**

In vitro, RSV can exert anti-tumor effects on GBM and improve the inflammatory response in the GBM microenvironment by inhibiting the activation of the JAK2/STAT3 signaling pathway. These findings provide new insights into potential therapeutic targets for GBM.

**Supplementary Information:**

The online version contains supplementary material available at 10.1007/s00432-024-05625-5.

## Introduction

GBM, classified as a WHO grade IV glioma, is a highly aggressive and lethal primary malignant brain tumor (Ghosh et al. 2017). The current conventional treatment of surgery followed by combined radiotherapy has limited efficacy in improving patients' prognosis (Perry et al. 2017). Despite temozolomide (TMZ) being the first-line chemotherapy for GBM, its clinical application is limited due to resistance and serious adverse effects such as infertility and myelosuppression (Patel et al. 2020). Therefore, there is an urgent need to explore new therapeutic modalities, such as emerging immunotherapies. However, the GBM microenvironment is characterized by a high degree of inflammation that mediates the immunosuppressive state of GBM (Rolim et al. 2022). Consequently, targeting inflammasome that promote tumor progression appears to be a promising direction to enhance therapeutic efficacy and improve survival in GBM.

In the inflammatory environment of the central nervous system (CNS), inflammatory factors promote a cascade reaction that activates microglia and recruits leukocyte infiltration, resulting in the production of additional inflammatory mediators. One such mediator is the NOD-like receptor family pyrin domain-containing 3 (NLRP3) inflammasome, which is known to contribute to brain edema, hemorrhage, damage to the blood–brain barrier (BBB), and neuronal death (Bellut et al. 2021). The NLRP3 inflammasome is a multiprotein complex that initiates inflammasome formation by interacting with the adaptor protein ASC. This interaction recruits and activates pro-caspase-1, leading to the production of active caspase-1. Active caspase-1 then cleaves inactive cytokines pro-IL-1β and pro-IL-18 into their mature forms, IL-1β and IL-18, respectively. The activation of IL-1β and IL-18 triggers a series of inflammatory responses (Christgen et al. 2020). Inflammation plays a significant role in the tumor microenvironment (TME) and is considered a hallmark of cancer (Hanahan 2022). Among the heterogeneous factors in the TME, aggressive inflammation stands out as one of the contributors to poor treatment outcomes in GBM (Litak et al. 2019). The malignant progression of GBM is associated with an inflammatory response in the TME, which promotes tumor growth, invasion, angiogenesis, and metastasis (Basheer et al. 2021). The inflammatory-rich TME, created by inflammatory cells through the production of pro-inflammatory cytokines, chemokines, and growth factors, contributes to immunosuppression and supports the survival of neural GBM cells (Solinas et al. 2010). Given its crucial role in TME-mediated inflammation, targeting the NLRP3 inflammasome presents a valuable therapeutic approach for CNS diseases (Wang and Hauenstein 2020).

Two protein families, Janus kinase (JAK)—signal transducer and activator of transcription (STAT), are involved in the JAK/STAT signaling pathway. The JAK/STAT signaling pathway is a crucial regulatory pathway for growth factors and inflammatory cytokines. It is activated in cancerous tissues of GBM patients and closely associated with the proliferation, differentiation, and function of various cells (Park et al. 2019). The JAK/STAT signaling pathway has been implicated in the pathogenesis of GBM (Ou et al. 2021; Smedley and Patra 2023). Among the members of the JAK/STAT family, JAK2 and STAT3 have been extensively studied in relation to GBM, and their increased activity is correlated with the severity of the disease (Iwamaru et al. 2007; Kim et al. 2014). Studies have shown that elevated JAK/STAT signaling upregulates and maintains the expression of various stem cell genes (such as CD44, NESTIN, PROMININ, PAX6, etc.) to preserve the stemness of GBM cells (Prasad et al. 2020). In addition, the JAK/STAT signaling pathway plays a role in tumor recurrence following surgery and chemotherapy. Therefore, targeting the JAK/STAT signaling pathway remains a potential therapeutic option for GBM.

**Fig. 1:**
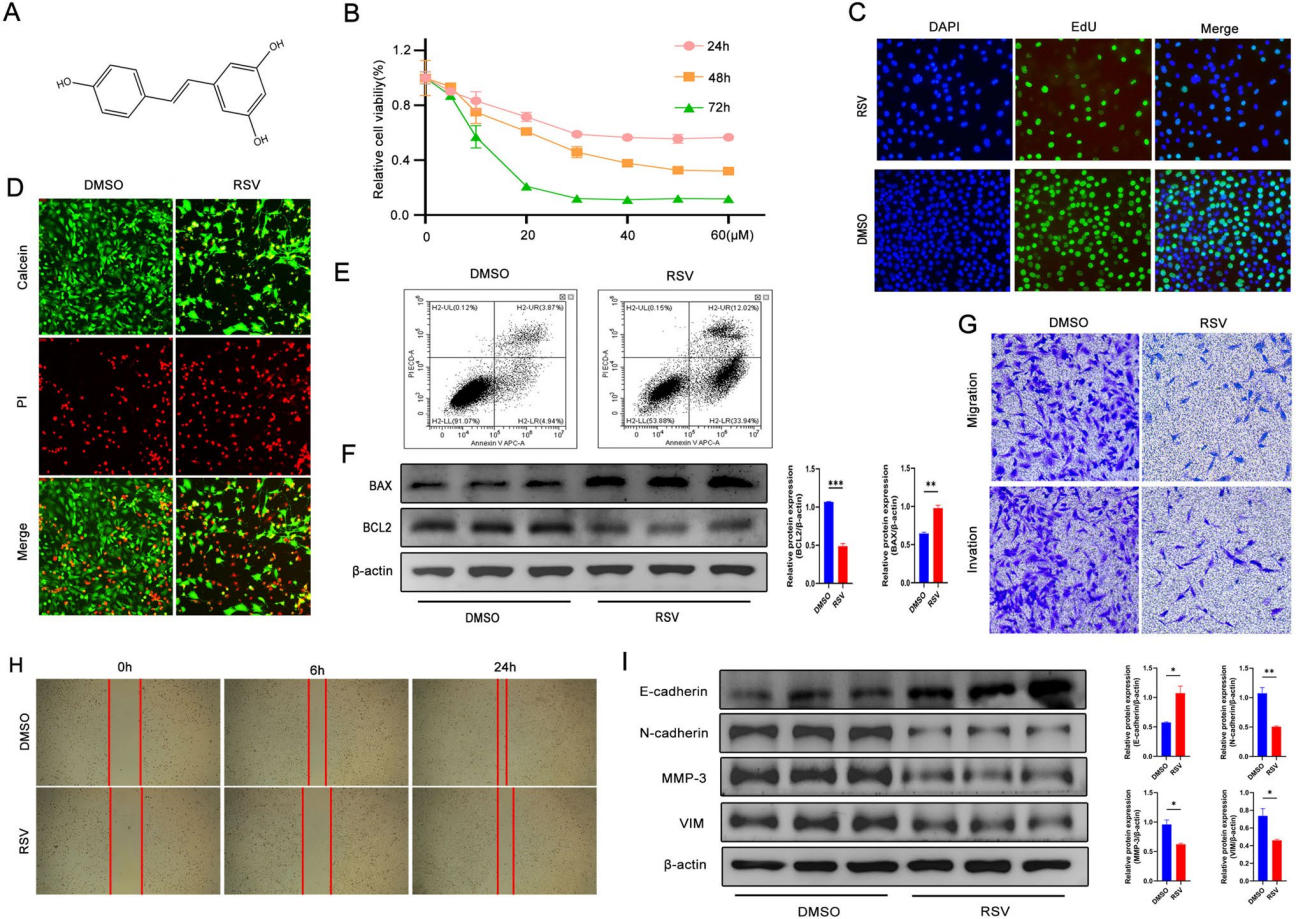
RSV ameliorates the malignant progression of GBM. **A** The chemical structural formula of RSV; **B** The effect of different concentrations of RSV and time treatments on cell viability of the LN-229 cell line was assessed using CCK8; **C** EdU proliferation assay was performed after treating the LN-229 cell line with an indicated concentration of RSV (20 μM) for 48 h; **D** AM/PI staining was conducted in the LN-229 cell line after treatment with the indicated concentration of RSV (20 μM) for 48 h; **E** Flow cytometry was employed to investigate the impact of RSV on apoptosis in GBM cells; **F** Western blot analysis was carried out to observe changes in the expression of apoptosis-related markers due to RSV treatment; **G** Transwell assay was implemented to assess the effect of RSV on the vertical migration and invasive ability of U87-MG cells; **H** Wound-healing assay was performed to evaluate the effect of RSV on the horizontal migration ability of LN-229 cells; **I** Western blot analysis was used to detect changes in the expression of EMT-related markers induced by RSV. The quantitative results of the protein levels are presented in the corresponding bar graphs

RSV, a polyphenolic compound (3,5,4′-trihydroxy-trans-stilbene), is widely present in sources such as red wine, grapes, soybeans, and peanuts (Fig. 1A). Natural RSV exists in both cis and trans isomers, with the trans-isomer being more stable and exhibiting greater physiological activities. Its ability to penetrate the blood–brain barrier with minimal toxicity on normal brain cells has garnered significant interest in the field of central nervous system diseases (Shu et al. 2011; Pallàs et al. 2014). However, there have been no reports on RSV's ability to regulate the inflammatory microenvironment of GBM. The impact of RSV on NLRP3 inflammasome expression and the JAK2/STAT3 pathway, as well as the potential interactions between NLRP3 inflammasome and the JAK2/STAT3 pathway, remain elusive. Therefore, this study aimed to uncover the specific mechanism through which RSV mitigates the inflammatory microenvironment in GBM cells, thereby inhibiting the malignant progression of GBM.

In this study, we investigated the antitumor effects of RSV on GBM and analyzed the relationship between its effects on the NLRP3 inflammasome and the JAK2/STAT3 pathway to offer new insights for the research on anti-GBM drugs.

## Materials and methods

### Reagents and antibodies

RSV was purchased from Shanghai Yuanye Biotechnology Co., Ltd (CAS: 501–36-0) and diluted with dimethyl sulfoxide (DMSO). The JAK/STAT activator RO8191 was purchased from Shanghai MCE Biotechnology (CAS: 691,868–88-9). JAK2, P-JAK2, STAT3, P-STAT3, and β-actin antibodies were purchased from Wuhan Sanying Biotechnology Co. E-Cadherin, N-Cadherin, MMP-3, and VIM antibodies were purchased from Taizhou Baijia Biotechnology Co. The Cell Counting Kit-8 (CCK-8) was purchased from White Shark Bio-technology Co. Ltd. The EdU Cell Proliferation Detection Kit and the Calcein (AM)/Propidium Iodide (PI) Staining kit were purchased from Baijia Biotechnology Co.

### Cell culture and processing

We purchased the human GBM cell lines LN-229 and U87-MG from Wuhan Punosai Life Science and Technology Co. Ltd. and cultured in Dulbecco's Modified Eagle's Medium (DMEM) purchased from Shanghai Datsunghil Biotechnology Co. Ltd. Cells were cultured at 37 °C in a humidified incubator with 5% CO2 in a medium supplemented with 10% fetal bovine serum (FBS) and 1% penicillin–streptomycin.

RSV powder and RO8191 were dissolved in DMSO to a final concentration of 10 mM and stored away from light. The GBM cells were cultured in complete medium for 24 h. Then, the appropriate concentration of RSV solvent was prepared by dilution as required. The culture was continued with complete medium containing RSV.

### Cell viability assay

We analyzed cell viability using the Cell Counting kit-8. Cells were seeded in 96-well plates and incubated in complete medium for 24 h prior to RSV treatment. Blank wells (containing only medium, no cells) were also prepared. After the specified incubation period, 10 μL of CCK-8 reagent was added to each well following the instructions provided with the CCK-8 kit. The absorbance was then measured at 450 nm using an enzyme marker after incubating at 37 °C for 2 h.

### Colony formation experiment

The appropriate number of cells was inoculated into 6-well plates. After the cells attached to the wall and resumed normal growth, the required amount of RSV solvent was added, and the cells were placed into the incubator for further incubation. After 1 week, the cells were fixed with 4% paraformaldehyde (FPA) for 15 min. The 6-well plates were washed three times with PBS. Then, they were stained with 0.1% crystal violet, washed again with PBS, and photographed using a digital camera.

### 5-Ethynyl-2'-deoxyuridine (EdU) proliferation assay

After treating the cells as required, according to the instruction manual of the Edu Cell Proliferation Assay Kit, the EdU incubation working solution was prepared and preheated at 37 °C. It was then added to the 96-well plate in half-exchange mode and incubated for 2 h. It was then washed twice with PBS buffer, fixed for 15 min at room temperature with 4% PFA, and washed again with PBS three times, 3 min at a time. The plate was then blotted and permeabilized with 0.5% Triton X-100 for 30 min at room temperature. After permeabilization, the plate was rinsed twice with PBS. After incubation for 30 min at room temperature away from light, the edu reaction solution was removed and the plate was washed three times with PBS. Nuclei were stained for 5 min with DAPI staining solution, twice washed in PBS, and photographed with an inverted fluorescence microscope.

### Calcein (AM)/propidium iodide (PI) staining

Live and dead cells were detected using the AM/PI kit. Cells were inoculated in a 96-well plate according to the manufacturer's instructions. After different stimuli, cells were washed with PBS and added with 100ul of prepared AM/PI working solution. After incubation at 37 °C for 30 min, the cells were observed and photographed with the help of a fluorescence microscope.

### Wound healing assay

Cells were inoculated into 6-well plates. When cell fusion reached approximately 80–90%, a pipette gun tip of the same specification (200 μL) was used to create a straight line in the wells. The wells were then washed twice with PBS before adding a mixture of drug and complete medium or drug-free medium for further culture. The healing of scratches in different groups was compared by observing and photographing the scratch area at 0h, 6h, and 24h under a light microscope.

### Transwell invasion and migration assays

Using a Transwell assay, migration and invasion abilities of cells were determined. Place the chambers without and with Matrigel matrix gel into a 24-well plate. Initially, 200 L of serum-free cell suspension were added to the upper chamber, and 600 L of complete medium were added to the lower chamber. The chambers were cultured in an incubator for 24–48 h after their additions. After incubation, cotton swabs were used to remove cells from the bottom of the chamber. The cells were fixed with 4% FPA for 15 min. As soon as the fixation was complete, the cells were washed in PBS and stained with 0.1% crystal violet for 15 min. After another round of PBS washing, the Transwell was inverted and placed onto a slide. Finally, the cells were observed and photographed under a microscope.

### Flow cytometric analysis

The cells in each group were treated as follows: First, the cells were digested with trypsin without ethylenediaminetetraacetic acid (EDTA) and collected in test tubes. Afterwards, the cells were centrifuged in pre-cooled PBS solution. Subsequently, the cells were resuspended in 1ml of 1 × binding buffer. Following the addition of 5 μL of Annexin V to each tube, they were incubated for 10 min at room temperature away from light. Finally, 5 μL of stain was added to the tubes, and the cells were subjected to flow-through assay.

### Western blot analysis

The treated cells were collected, and proteins were extracted using RIPA lysate. The protein extracts were electrophoretically separated and transferred to a polyvinylidene difluoride membrane (PVDF). After blocking with 5% skimmed milk powder for 2 h at room temperature, the membrane was washed three times with TBST (TBS with Tween-20) buffer. The target antibody was added and incubated overnight at 4 °C. It was then washed three times with TBST buffer, then the membrane was incubated for 2 h at room temperature with a horseradish peroxidase-conjugated secondary antibody. Finally, the membrane was again washed three times with TBST buffer. The membrane was then developed using ECL luminescence reagent. The Image J software was used to detect the grey value of each protein band and analyze the protein expression level.

### Immunofluorescence staining

Following removal of the supernatant, the cells were fixed with 4% FPA for 15 min, washed three times with PBS for 5 min each time, and permeabilized with 0.5% Triton X-100 for 30 min. To block non-specific binding sites, the cells were incubated for 30 min with 5% goat serum, then overnight at 4 °C with P-STAT3 antibody. The next day, the cells were washed three times in PBS before adding the fluorescent secondary antibody. After adding the fluorescent secondary antibody, the cells were incubated at room temperature for 2 h. After washing with PBS, the cells were stained with DAPI staining solution for 5 min and observed using a fluorescence microscope.

### Network pharmacology analysis

The 347 putative targets of RSV were obtained from various sources, including TCMSP (https://www.tcmsp-e.com/), SwissTargetPrediction (https://www.swisstargetprediction.ch/), TargetNet (http://targetnet.scbdd.com/), STITCH (https://www.uniprot.org/), and the SEA database (https://bkslab.org/).

GBM-associated genes were obtained from the GeneCards database, and 2,265 targets with correlation coefficients greater than the mean were selected. Out of these, 179 cross-targets were further analyzed using the String database for protein–protein interaction (PPI) analysis, and the DAVID database for functional enrichment analysis in terms of gene ontology (GO) and KEGG pathways.

### Statistical analysis

All data were statistically analyzed using GraphPad Prism 9.7 software. The data were expressed as mean ± standard error. Differences between two groups were analyzed using a *t* test. Differences were considered statistically significant at *P* < 0.05. *p < 0.05; **p < 0.01; ***p < 0.001.

## Result

### RSV inhibits GBM cell proliferation, migration and invasion and promotes apoptosis in GBM cells.

To determine the anti-GBM effects of RSV in vitro, we measured cell viability in two established human GBM cell lines, LN-229 and U87-MG, utilizing the CCK8 assay. The findings indicated that RSV reduced LN-229 cell viability in a dose- and time-dependent manner (Fig. 1B), with similar results observed in the U87-MG cells (Fig. S1B). Following RSV exposure, GBM cells exhibited morphological changes, including bubbling, shrinkage, indistinct edges, rounder shapes, and increased refractivity; yet, RSV's impact on HA cells (astrocytes) was not significant (Fig. S1A). Furthermore, a concentration-dependent inhibitory effect of RSV on GBM cell growth was confirmed by colony formation assays (Fig. S1C). The IC50 for the LN-229 cell line at 48-h post-RSV treatment was computed as 17.90 μM via the GraphPad Prism software, pursuant to a dose–response curve. LN-229 cells displayed greater sensitivity to RSV compared to U87-MG cells, whose 48-h IC50 was 25 μM. For consistency in subsequent experiments, a standard dose of 20 μM RSV was adopted. Additional examination of proliferation using the EdU assay revealed a substantial, concentration-dependent decrease in EdU-positive cells post-treatment (Figs. 1C and S1D). AM/PI staining results confirmed the aforementioned findings, as evidenced by a marked escalation in dead cells and a concurrent decline in the green fluorescent signal indicative of viable cells (Fig. 1D). Utilizing flow cytometry and Western blot analysis to probe the apoptotic effects of RSV on GBM cells revealed both early and late-stage apoptotic processes were enhanced following RSV administration at a steady 20 μM dose (Fig. 1E). In the mitochondria-dependent apoptotic pathway, Bcl-2 family proteins are pivotal regulators, with pro-apoptotic members like Bax precipitating, and anti-apoptotic members like Bcl-2 averting cell death. Western blot analyses substantiated that RSV treatment led to upregulation of pro-apoptotic protein Bax and downregulation of anti-apoptotic protein Bcl-2 (Fig. 1F). Assessing the influence of RSV on GBM cell migration and invasion, Transwell assays demonstrated a notable reduction in these capabilities among the RSV-treated cells in contrast to the untreated controls (Fig. 1G). Similarly, wound healing assays revealed that RSV considerably impeded the horizontal movement of GBM cells (Fig. 1H). Further evaluation through Western blot analysis of the factors inhibiting GBM cell migration and invasion post-RSV treatment indicated significant diminution in mesenchymal protein expression levels (N-cadherin, Vimentin, and MMP-3) and a suggestive upregulation of epithelial markers (E-cadherin) as opposed to control samples (Fig. 1I). Collectively, these findings advocate for the potential of RSV as a therapeutic agent against GBM.

### Network pharmacological analysis of potential mechanisms of RSV antitumour resistance

**Fig. 2:**
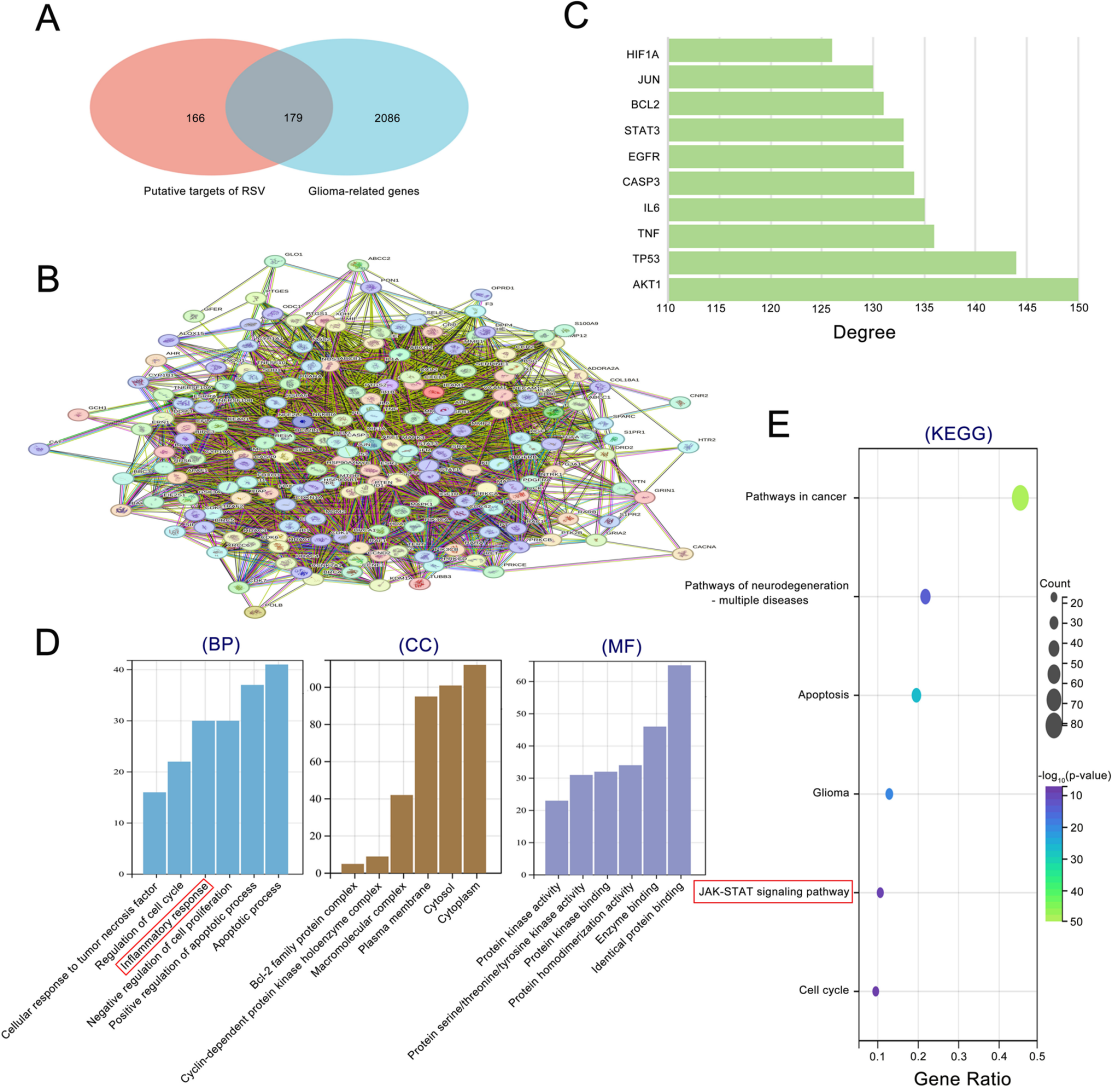
Network pharmacological analysis of potential mechanisms of RSV anti-GBM. **A** Venn diagram illustrating the cross-targets of RSV and GBM; **B** PPI network analysis investigating the interactions between RSV and GBM cross-targets; **C** The top 10 targets with significant degrees of freedom in the PPI network, including STAT3 as one of the core targets; **D** GO enrichment analysis. E. KEGG enrichment analysis

Based on data from TCMSP, Target Net, Swiss Target Prediction, STITCH, and SEA databases, we identified 347 RSV targets. GBM-associated genes were sourced from the GeneCards database, and 2,265 genes with correlation coefficients higher than the mean were screened. Subsequently, 179 crossover gene targets from both datasets were utilized for further analyses (Fig. 2A). To explore the interactions among the cross-targets, the 179 targets were input into the STRING database for network analysis (Fig. 2B). The top 10 targets were then refined based on node freedom. These targets comprised AKT1, TP53, TNF, IL6, CASP3, EGFR, STAT3, BCL2, JUN, and HIF1A (Fig. 2C). For a deeper understanding of the functions of the cross-targets, GO and KEGG analyses were conducted. The results of the GO analyses indicated that the most relevant biological processes included the apoptotic process, positive regulation of apoptosis, and inflammatory response. The most relevant cellular component was the cytoplasm, and the most relevant molecular function was identical protein binding (Fig. 2D). Pro-tumour inflammation is a critical component of the tumor microenvironment, mediating GBM invasion, metastasis, and promoting GBM immune resistance. KEGG analysis revealed cross-targeting with Pathways in cancer, Pathways of neurodegeneration—multiple diseases, pathways of apoptosis, glioma, JAK–STAT signaling pathway, and cell cycle (Fig. 2E). Network pharmacological analyses unveiled that the anti-tumor activity of RSV may be closely linked to the improvement of the inflammatory response in GBM. Furthermore, the JAK/STST signaling pathway might represent a potential mechanism for enhancing the inflammatory response, with STAT3 serving as one of the core targets.

### RSV selectively inhibits activation of the JAK2/STAT3 pathway.

**Fig. 3:**
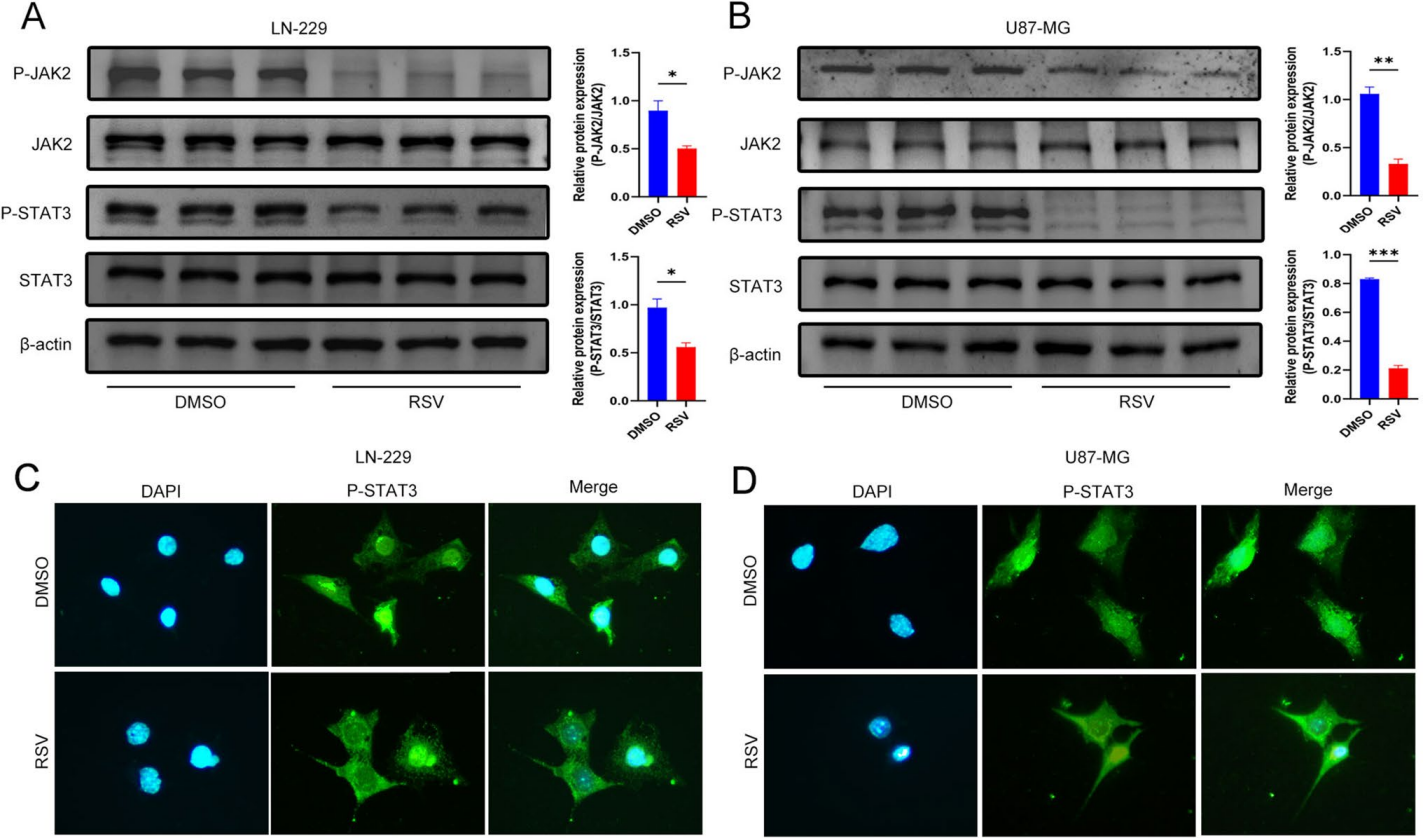
RSV inhibits activation of the JAK2/STAT3 pathway. **A** Western blot analysis showed that RSV reduced the levels of P-JAK2 and P-STAT3 in the LN-229 cell line; **B** Western blot analysis revealed that RSV also decreased the levels of P-JAK2 and P-STAT3 in the U87-MG cell line; **C** Immunofluorescence was used to examine the effect of RSV treatment on P-STAT3 nuclear translocation in the LN-229 cell line; **D** Immunofluorescence was employed to detect the effect of RSV treatment on P-STAT3 nuclear translocation in the U87-MG cell line. The quantitative results of the protein levels are presented in the corresponding bar graphs

Inflammatory processes are known to involve multiple signaling pathways, among which the JAK/STAT signaling pathway significantly impedes inflammation (Hu et al. 2020), and inhibiting the JAK2/STAT3 pathway can reduce NLRP3-mediated inflammatory responses (Zhu et al. 2021). Drawing from network pharmacology, we aimed to confirm these findings and determine whether the anti-GBM effects of RSV are mediated through the JAK2/STAT3 signaling pathway. Western blot analyses revealed that RSV differentially inhibited the activation of the JAK2/STAT3 pathway in the LN-229 cell line, as evidenced by prominent reductions in the phosphorylation levels of both JAK2 and STAT3. However, the total protein expression levels of JAK2 and STAT3 did not exhibit significant alterations (Fig. 3A). Parallel results were observed in the U87-MG cell line (Fig. 3B). As a classical transcription factor, STAT3 typically translocates to the nucleus to regulate target genes, thereby manifesting its biological functions. Delving deeper into the RSV-mediated regulation of the JAK2/STAT3 pathway, immunofluorescence assays demonstrated a diminution in the nuclear translocation of P-STAT3, with an increased accumulation in the cytoplasm following RSV treatment in the LN-229 cell line (Fig. 3C). Similar results were detected in the U87-MG cell line (Fig. 3D). Consequently, these findings suggest that RSV exerts its anti-GBM effects, at least in part, by inhibiting the activation of the JAK2/STAT3 signaling pathway.

### RSV improves the inflammatory microenvironment of GBM.

**Fig. 4:**
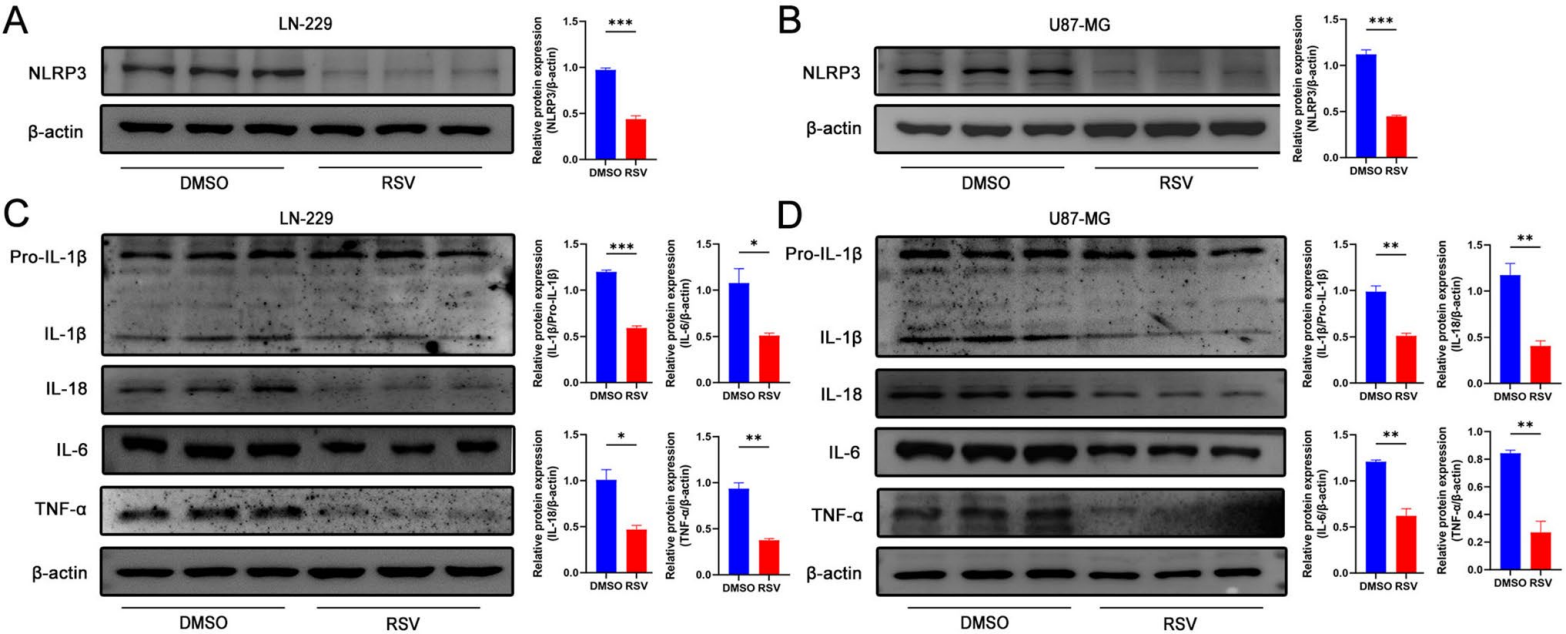
RSV improves the inflammatory response state of the GBM microenvironment. **A** RSV decreased the expression of inflammasome NLRP3 in LN-229 cell line; **B** RSV decreased the expression of inflammasome NLRP3 in U87-MG cell line; **C** RSV decreased the expression of inflammatory factors IL-1β, IL-18, IL-6, and TNFα in LN-229 cell line; **D** RSV decreased the expression of inflammatory factors IL-1β, IL-18, IL-6, and TNFα in U87-MG cell line. The quantitative results of the protein levels are presented in the corresponding bar graphs

The NLRP3 inflammasome is a multimolecular complex consisting of an adaptor protein called apoptosis-associated speck-like protein containing a CARD domain (ASC) and the effector protein, cysteine-1. Upon activation, the NLRP3 protein interacts with ASC and pro-caspase-1, resulting in the production of cleaved caspase-1. This mechanism subsequently initiates the cleavage and secretion of the proinflammatory cytokines pro-IL-1β and pro-IL-18 (Jo et al. 2016). IL-1β enhances the inflammatory cascade response by directly inducing IL-2 expression, recruiting neutrophils, and releasing other pro-inflammatory cytokines and chemokines to accelerate the inflammatory response (e.g., TNF-α and IL-6) (Drummond et al. 2019). Activation of the NLRP3 inflammasome and its associated proteins promotes GBM-related inflammatory responses and facilitates GBM progression. The JAK2/STAT3 pathway elevates the levels of several pro-inflammatory cytokines, which are known to play a critical role in the malignant progression of GBM. The NLRP3 inflammasome has been reported to be activated in GBM cells and function as a positive regulator of proliferation and metastasis. Network pharmacological analyses revealed that the anti-GBM effects of RSV may be related to the inflammatory response. To determine whether RSV is involved in regulating the expression of the inflammasome NLRP3 and its associated pro-inflammatory cytokines, the LN-229 cell line was treated with RSV. Western blot results showed that RSV indeed decreased the levels of inflammasome NLRP3 and its downstream IL-1β, IL-18, IL-6, and TNFα. However, the alteration of pro-IL-1β by RSV was not significant (Fig. 4A, C). Similar results were observed after treating the U87-MG cell line using the same approach (Fig. 4B, D). These results indicate that RSV is able to attenuate the inflammatory response of the GBM microenvironment, suggesting that the anti-tumor effect of RSV is partly mediated by improving the inflammatory state of GBM.

### The JAK/STAT agonist RO8191 partially reverses NLRP3

**Fig. 5:**
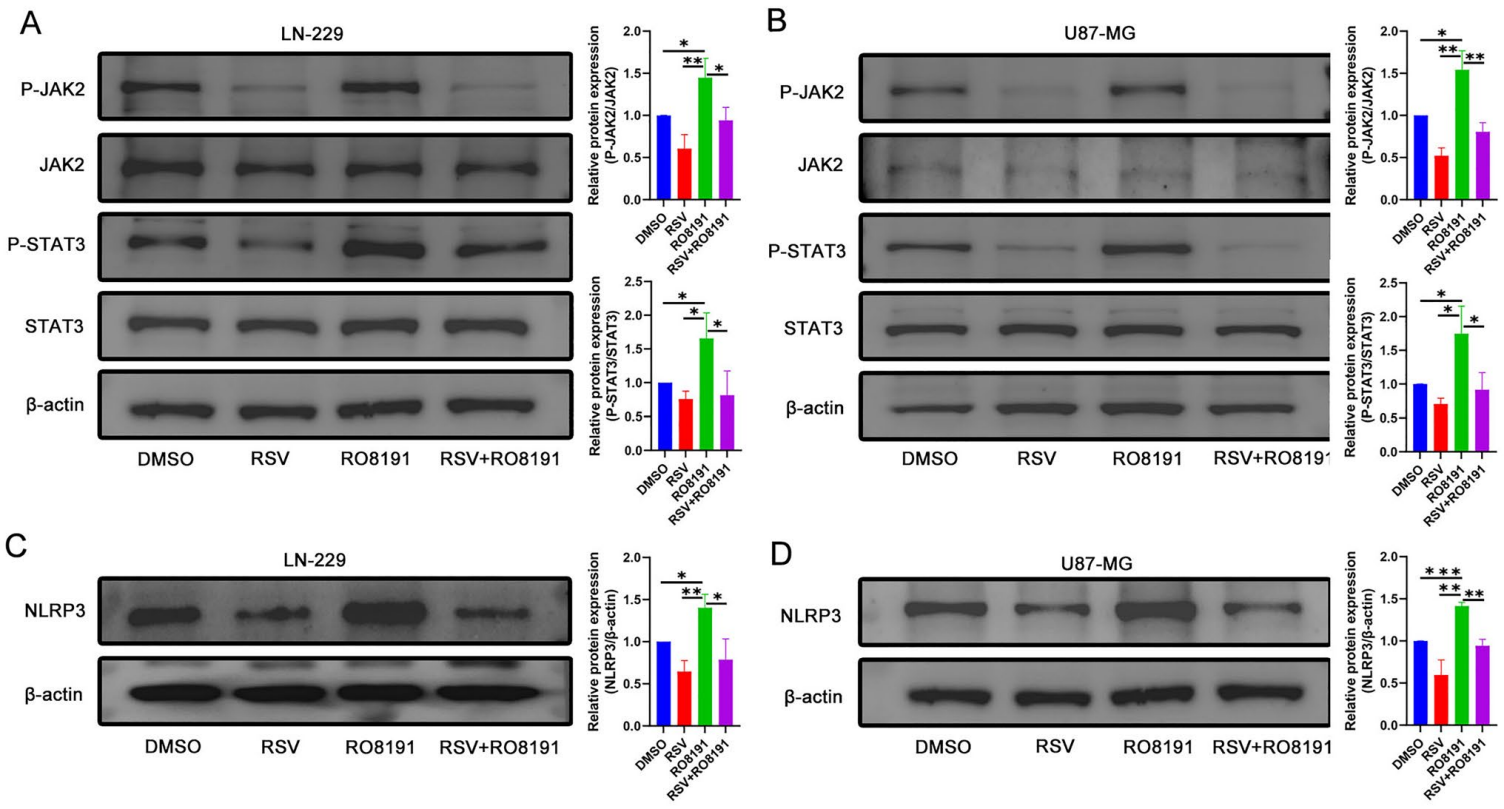
RSV inhibits the activation of NLRP3 through the regulation of JAK2/STAT3 signaling pathway. **A** The protein phosphorylation levels of JAK2 and STAT3 in LN-229 cell line activated by RO8191 treatment were detected using Western blot analysis; **B** The protein phosphorylation levels of JAK2 and STAT3 in U87-MG cell line activated by RO8191 treatment were also detected using Western blot analysis; **C** RO8191 treatment activated the expression level of NLRP3 in the LN-229 cell line and partially reversed the inhibition of NLRP3 by RSV; **D** RO8191 treatment also activated the expression level of NLRP3 in the U87-MG cell line and partially reversed the inhibition of NLRP3 by RSV. The quantitative results of the protein levels are presented in the corresponding bar graphs

The malignant progression of GBM is associated with a neuroinflammatory response, and the neuroinflammation-rich TME formed by inflammatory cells, through the production of proinflammatory cytokines, chemokines, and growth factors, contributes to the immunosuppressive response of GBM and increases its survivability. The NLRP3 inflammasome-mediated inflammatory response plays a key role in the development of GBM and has emerged as an important therapeutic target for its treatment. This study demonstrates the anti-GBM activity of RSV, at least in part, by improving the inflammatory state of the GBM microenvironment. However, the relationship between RSV's improvement of NLRP3-mediated inflammatory responses and the inhibition of the JAK2/STAT3 signaling pathway remains unclear. To further investigate this relationship in GBM progression, LN-229 and U87-MG cell lines were treated with the JAK/STAT agonist RO8191. The results showed that the addition of RO8191 increased the phosphorylation levels of JAK2 and STAT3, and partially reversed the inhibition of the JAK2/STAT3 signaling pathway by RSV (Fig. 5A, B). As expected and consistent with this, the expression level of NLRP3 was elevated after the addition of RO8191, partially reversing the inhibition of NLRP3 by RSV (Fig. 5C, D). The above information suggests that the malignant progression of GBM is partly caused by the activation of NLRP3 through the JAK2/STAT3 signalling pathway. The improvement of the inflammatory microenvironment of GBM by RSV through the targeting of NLRP3 via the JAK2/STAT3 signalling pathway provides a new direction for the immunotherapy of GBM.

## Discussion

GBM is characterized by a poor prognosis due to the presence of severe inflammation and a lack of effective therapeutic measures. The inflammatory response is believed to be associated with immunosuppression, and among the highly heterogeneous factors in TME, aggressive inflammation is a significant contributor to the poor outcome of GBM (Litak et al. 2019). GBM cells have the ability to create an inflammatory microenvironment that promotes tumor progression and infiltrative migration (Zimmer et al. 2019). Malignant progression of GBM has been linked to an inflammatory response, which has been implicated in the hallmarks of tumor growth, invasion, angiogenesis, and metastasis (Basheer et al. 2021). Given the association of the inflammatory response with immunosuppression, there is an urgent need to develop more effective anti-GBM drugs and to improve the inflammatory state of the GBM microenvironment.

The hyperactivation of the inflammasome is associated with the modulation of immune responses, cell proliferation, and the pathogenesis of cancer. Studies on the inflammasome in various cancers have demonstrated their potential as a therapeutic target. Inflammasomes contribute to many diseases, including cancer, when immune responses and inflammatory processes are uncontrolled (Karki and Kanneganti 2019). The association between neuroinflammation and inflammasomes is an emerging research topic in GBM (Alghamri et al. 2021; Missiroli et al. 2021). There is growing evidence that inflammasome-associated responses mediate immune system malfunction in the TME of GBM, and that the activation of inflammasome and production of proinflammatory cytokines can accelerate GBM progression (Rolim et al. 2022). Despite their role in the organism's defense function, inflammasomes can instead be disadvantageous to the organism when the immune system is imbalanced. The function of inflammasomes in GBM has been closely studied since 2014. It has been shown that the NLRP3 inflammasome is hyperactive in patients with GBM (Tarassishin et al. 2014), suggesting that it may serve as a marker of disease progression (Li and Liu 2014). In GBM cells, NLRP3 promotes epithelial–mesenchymal transition (EMT) and activates the PTEN/AKT signaling pathway (Yin et al. 2018). It has also been shown that the NLRP3 inflammasome promotes GBM cell proliferation and invasion by regulating IL-1β and NF-κB p65 signaling (Xue et al. 2019). Due to the significant biological relevance of inflammasomes in GBM, targeting inflammasomes to identify the specific mechanisms that regulate them may be an important direction for the treatment of GBM.

JAK2, an important member of the tyrosine kinase protein family, is involved in regulating various pathological responses, including apoptosis, pyroptosis, and inflammation (Rosée et al. 2020); (Shen et al. 2021); (Wang et al. 2022). The most crucial downstream molecule of JAK2 is STAT3. After binding to the membrane receptor, JAK2 transmits signals to the intracellular compartment. Activated JAK2 can recruit STAT3 and phosphorylate it, resulting in the formation of a dimer (Wang et al. 2021). This dimer can relocate to the nucleus to regulate gene transcription (Jaśkiewicz et al. 2020). Studies have shown that JAK/STAT signaling plays a key role in the inflammatory response by mediating the expression of all sorts of inflammatory proteins (Liu et al. 2021). Inflammatory responses are mediated by STAT3, but its role in inflammatory responses remains controversial, and certain anti-inflammatory agents activate STAT3 to reduce inflammation (Zhong et al. 2021; Zhu et al. 2021). Our research shows that RSV mitigates the inflammatory response in GBM by inhibiting the activation of the JAK2/STAT3 signaling pathway.

In this study, we reported the anti-GBM effect of RSV. RSV was able to reduce GBM cell viability, and its inhibitory effect on GBM cell proliferation showed concentration- and time-dependence, inhibiting the proliferation of GBM cells while also promoting their apoptosis. What's more, within the effective concentration range of RSV exerting anti-GBM activity, it was not found to produce obvious toxic effects on normal astrocytes. In addition, transwell showed that RSV also significantly inhibited the invasion and migration ability of GBM cells. Similarly, Western blot showed that RSV significantly reduced the expression levels of several markers associated with tumour EMT. Network pharmacological analyses revealed that JAK/STAT and inflammatory responses may be possible mechanisms by which RSV exerts anti-GBM. Our results also suggest that RSV exerts anti-GBM effects in vitro partly by inhibiting the activation of the JAK2/STAT3 signalling pathway, which in turn attenuates the inflammatory response in the GBM microenvironment. Based on the strong link between NLRP3 inflammasome activation and GBM cell metastasis, the results suggest that RSV inhibits the expression of NLRP3 and its downstream inflammatory factors by JAK2/STAT3 signalling pathway, suppresses the proliferation of GBM cells while promoting their apoptosis, and exerts an inhibitory effect on GBM cell invasion and migration.

## Conclusion

In this study, we discovered that RSV effectively hindered the malignant progression of GBM. RSV achieved this by inhibiting the activation of NLRP3 inflammatory vesicles and their downstream inflammatory factors through the JAK2/STAT3 signaling pathway. These findings imply that RSV demonstrates its anti-GBM effects by alleviating the inflammatory response in the TME of GBM. This insight offers valuable leads for the development of more potent anti-GBM medications.

## Supplementary Information

Below is the link to the electronic supplementary material.

Supplementary file1 (ZIP 36116 KB)

Supplementary file2 (DOCX 25 KB)

Supplementary file3 Supplementary Figure S1 RSV improves the malignant progression of GBM. A, Cellular morphology of HA (astrocyte cell line), LN-229, and U87-MG under light microscope after 48 hours of treatment with various concentrations of RSV; B, Assessment of cell viability in U87-MG cell line treated with different concentrations of RSV for different periods of time using CCK8 assay; C, Colony formation assay to evaluate the impact of RSV treatment at different concentrations on GBM proliferation; D, EdU proliferation assay conducted on LN-229 cell line treated with various concentrations of RSV for 48 hours (TIF 22541 KB)

## Data Availability

All data are contained within the manuscript. The datasets used and/or analysed during the current study are available from the corresponding author on reasonable request.

## Abbreviations

RSV: Resveratrol
GBM: Glioblastoma
TMZ: Temozolomide
CNS: Central nervous system
BBB: Blood–brain barrier
TME: Tumor microenvironment
JAK: Janus kinase
STAT: Signal transducer and activator of transcription
GO: Gene Ontology
KEGG: Kyoto encyclopedia of genes and genomes
DMSO: Dimethyl sulfoxide
CCK-8: Cell counting Kit-8
AM/PI: Calcein/Propidium Iodide
DMEM: Dulbecco's modified eagle's medium
FBS: Fetal bovine serum
FPA: Paraformaldehyde
EdU: 5-Ethynyl-2'-deoxyuridine
DAPI: 4,6-Diamidino-2-phenylindole
EDTA: Ethylene diamine tetra-acetic acid
PVDF: Polyvinylidene difluoride membrane
TBST: TBS with Tween-20
