# Supplementary figures and images for: Resveratrol ameliorates glioblastoma inflammatory response by reducing NLRP3 inflammasome activation through inhibition of the JAK2/STAT3 pathway (part 2 of 2)
Source: J Cancer Res Clin Oncol. 2024 Mar 28;150(3):168. doi: 10.1007/s00432-024-05625-5 (PMC10978631; doi:10.1007/s00432-024-05625-5)

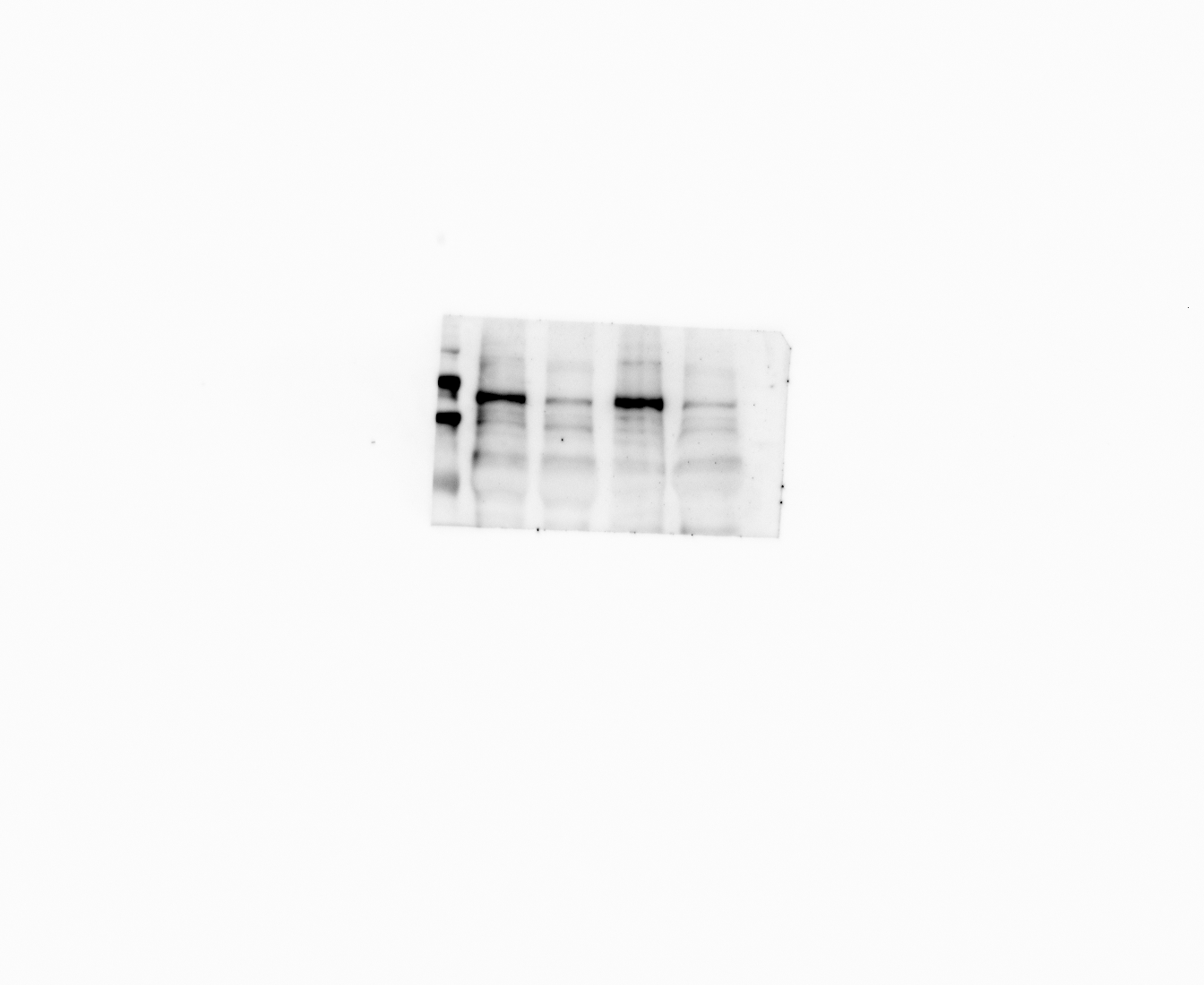

Supplement: Supplementary file 1 — Supplementary file1 (ZIP 36116 KB) [file 432_2024_5625_MOESM1_ESM.zip › Original Images for BlotsGels/5.Figure 5/LN229/2.P-JAK2/1/1-3-JAK2(Y).tif]

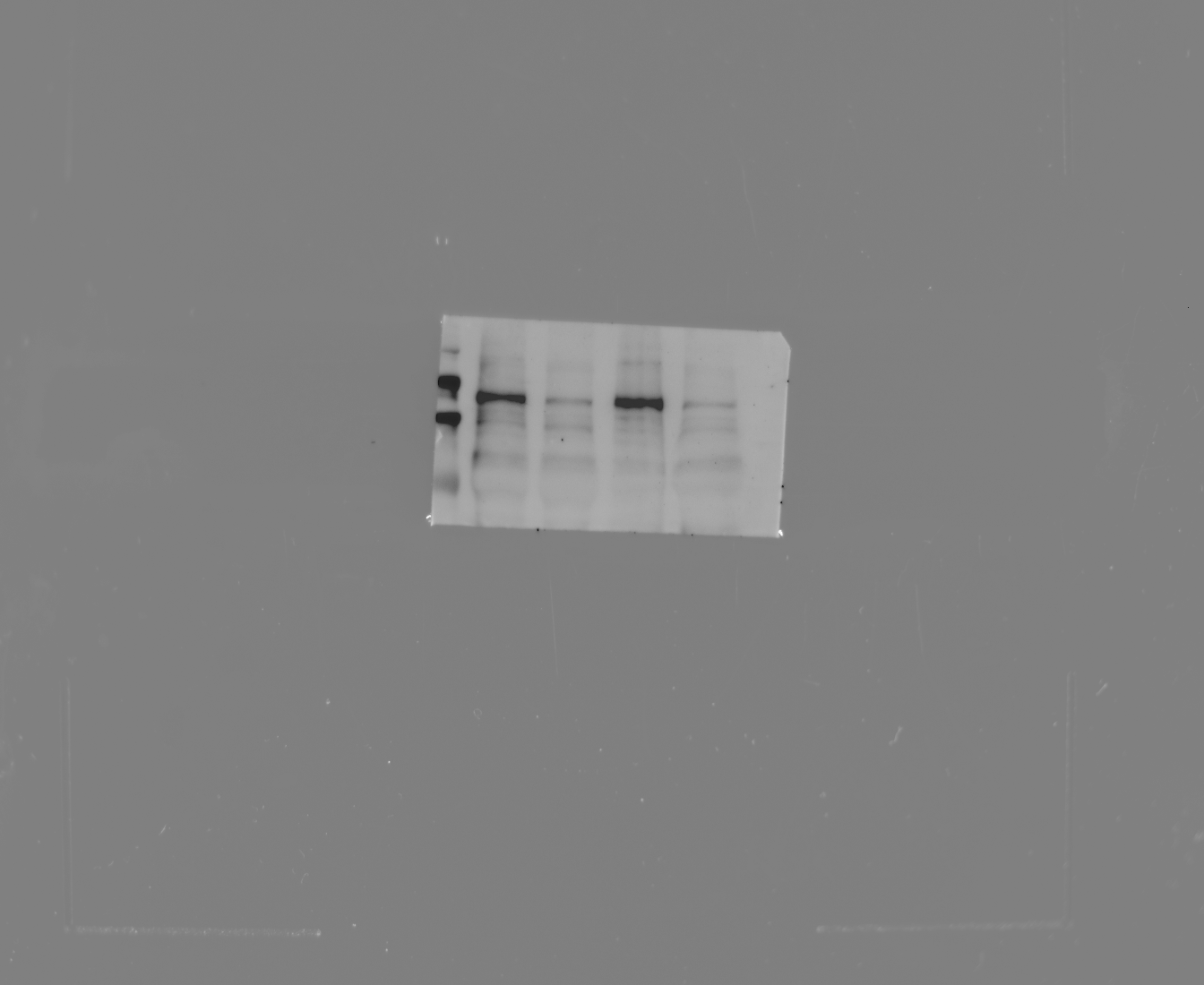

Supplement: Supplementary file 1 — Supplementary file1 (ZIP 36116 KB) [file 432_2024_5625_MOESM1_ESM.zip › Original Images for BlotsGels/5.Figure 5/LN229/2.P-JAK2/1/1-3-JAK2.tif]

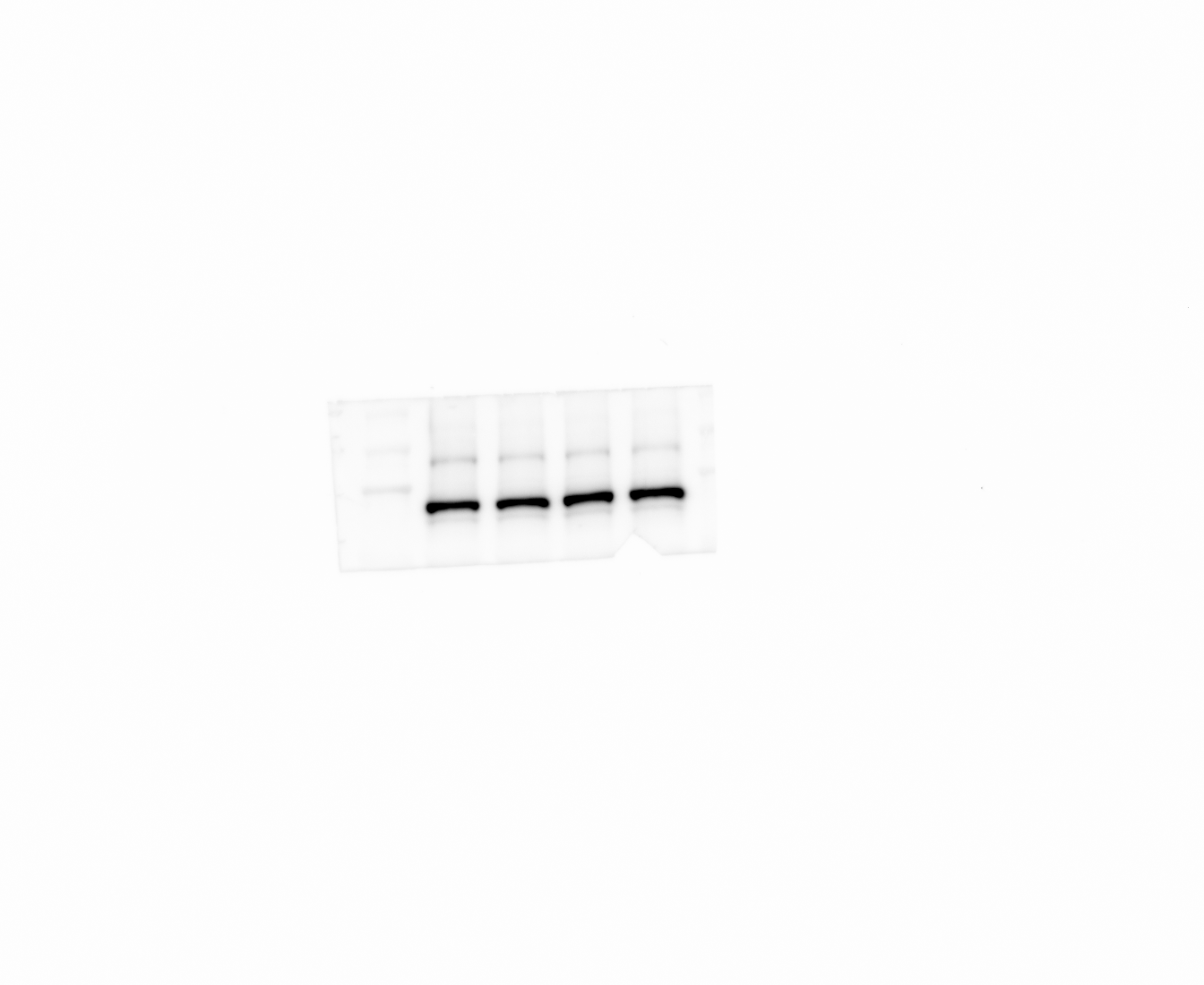

Supplement: Supplementary file 1 — Supplementary file1 (ZIP 36116 KB) [file 432_2024_5625_MOESM1_ESM.zip › Original Images for BlotsGels/5.Figure 5/LN229/3.STAT3/二孵(内参同JAK2)/1/1-1-STAT3(Y).tif]

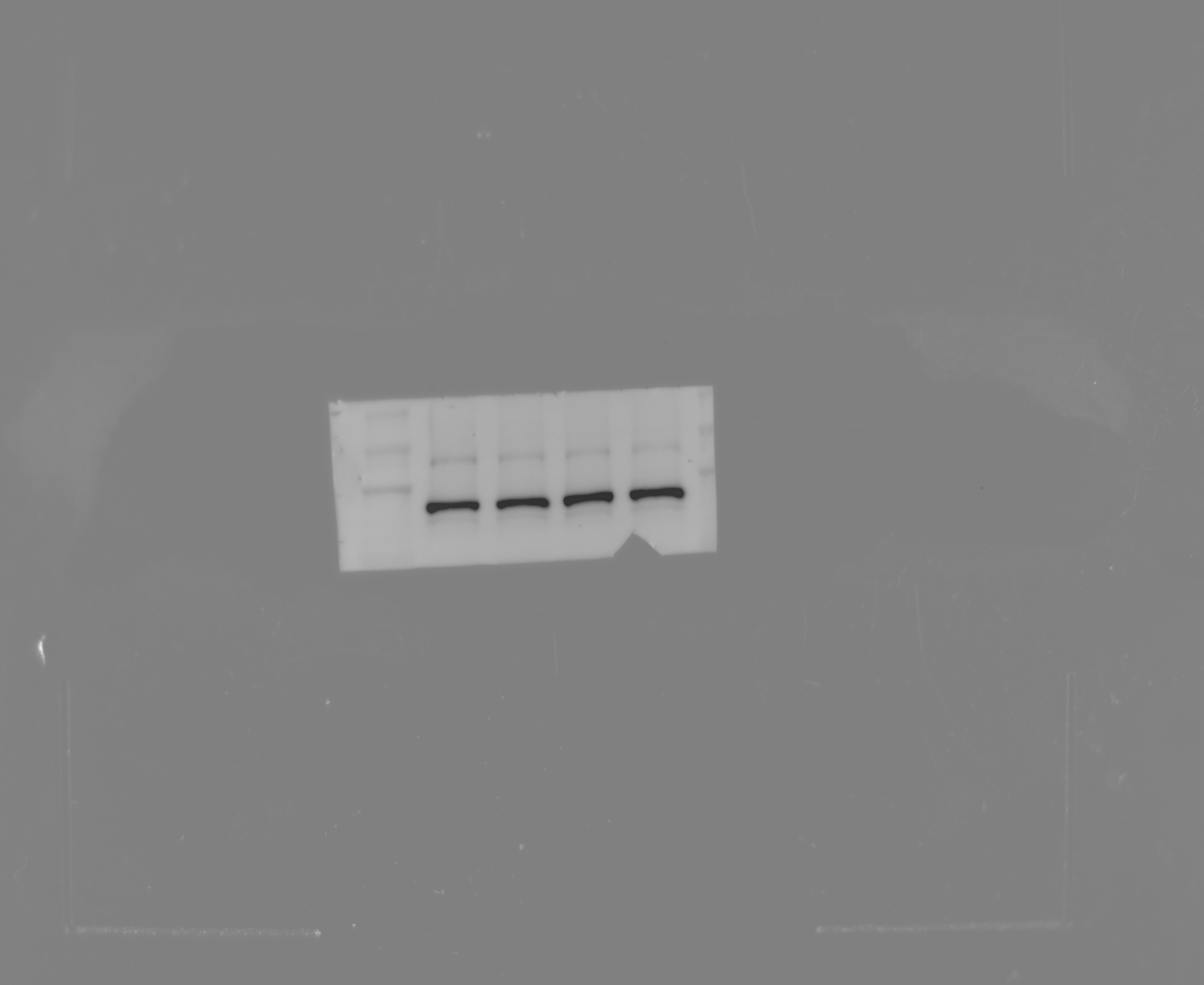

Supplement: Supplementary file 1 — Supplementary file1 (ZIP 36116 KB) [file 432_2024_5625_MOESM1_ESM.zip › Original Images for BlotsGels/5.Figure 5/LN229/3.STAT3/二孵(内参同JAK2)/1/1-1-STAT3.tif]

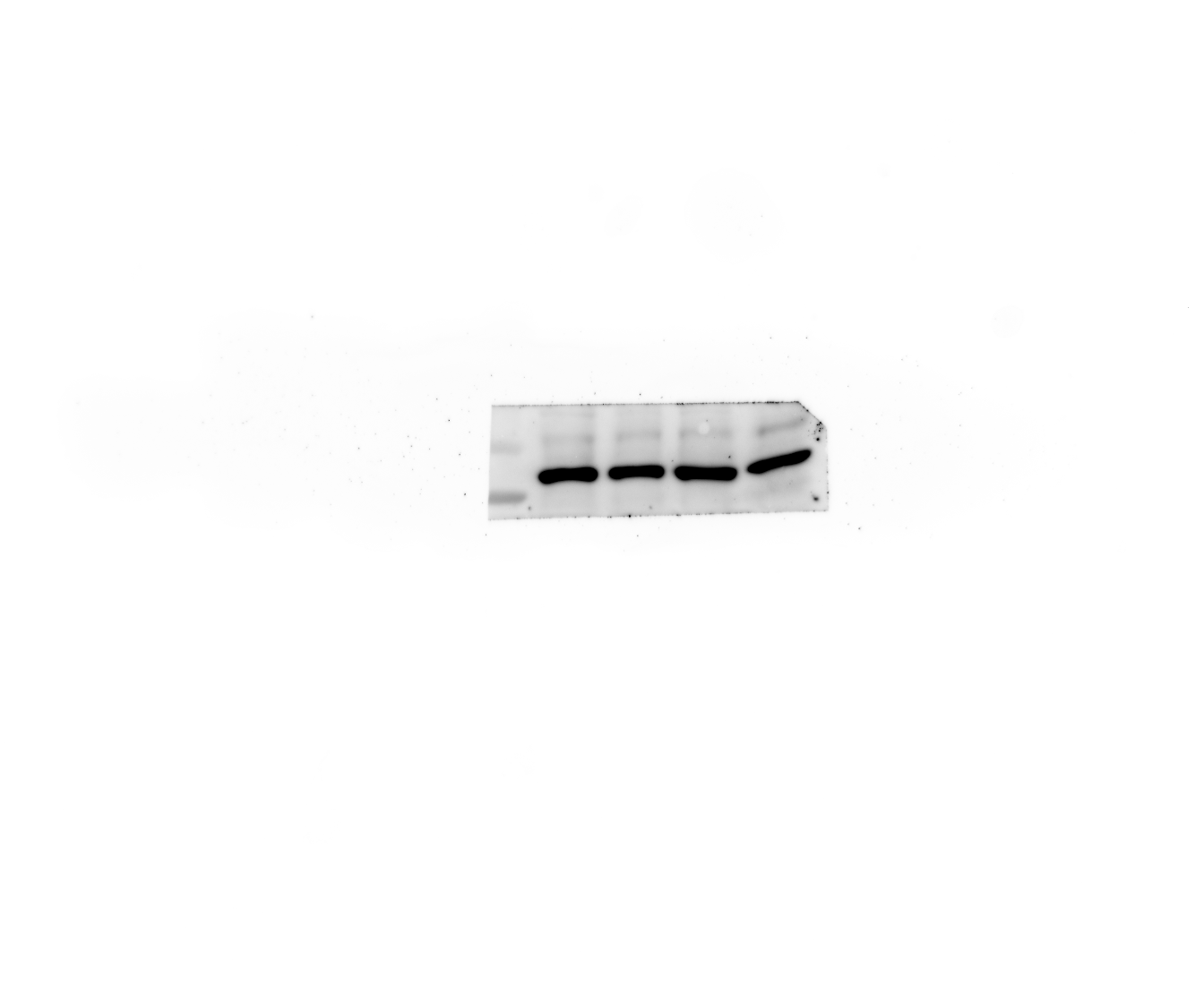

Supplement: Supplementary file 1 — Supplementary file1 (ZIP 36116 KB) [file 432_2024_5625_MOESM1_ESM.zip › Original Images for BlotsGels/5.Figure 5/LN229/4.P-STAT3/1/7.A(样品图).tif]

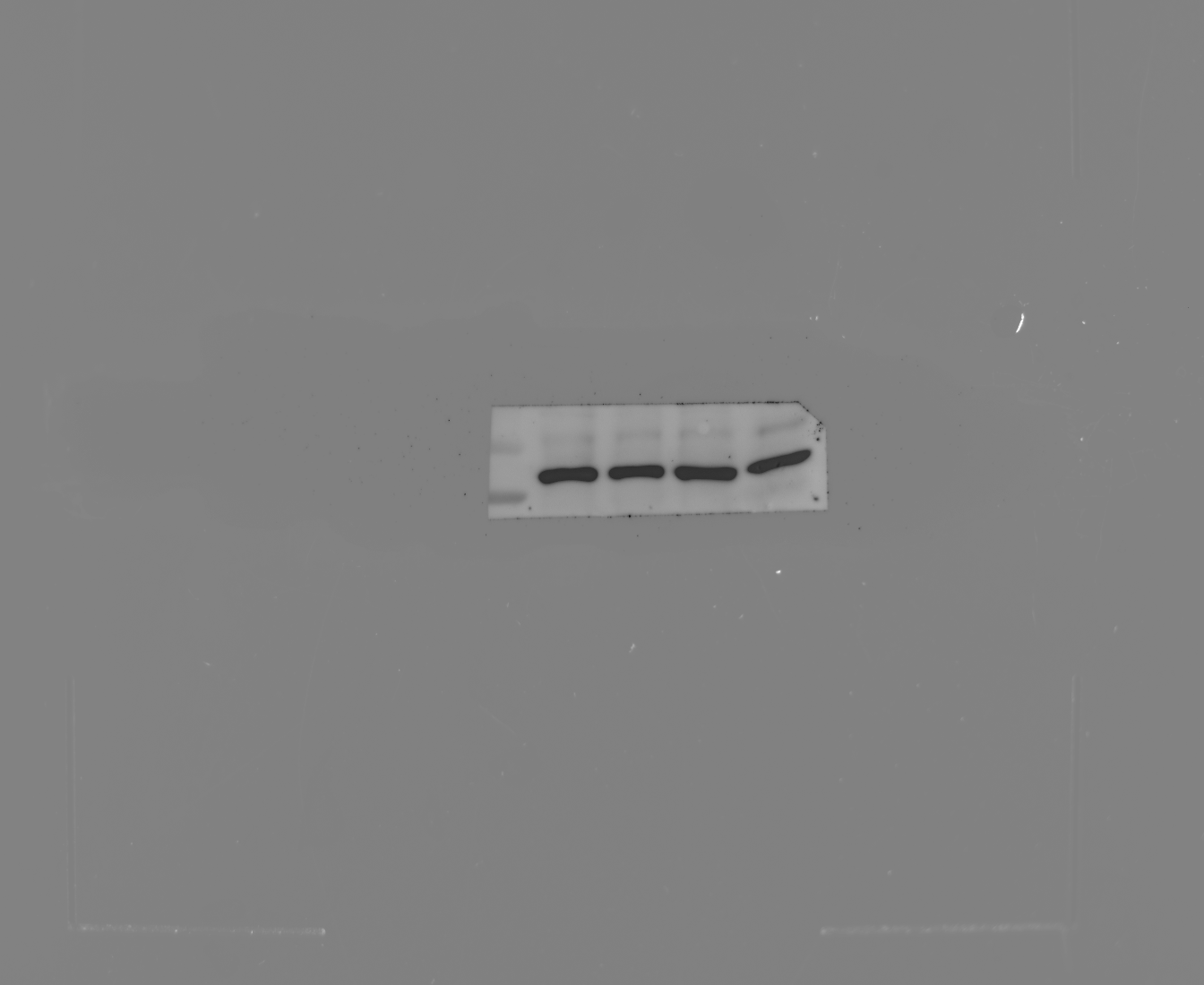

Supplement: Supplementary file 1 — Supplementary file1 (ZIP 36116 KB) [file 432_2024_5625_MOESM1_ESM.zip › Original Images for BlotsGels/5.Figure 5/LN229/4.P-STAT3/1/7.A(叠加图).tif]

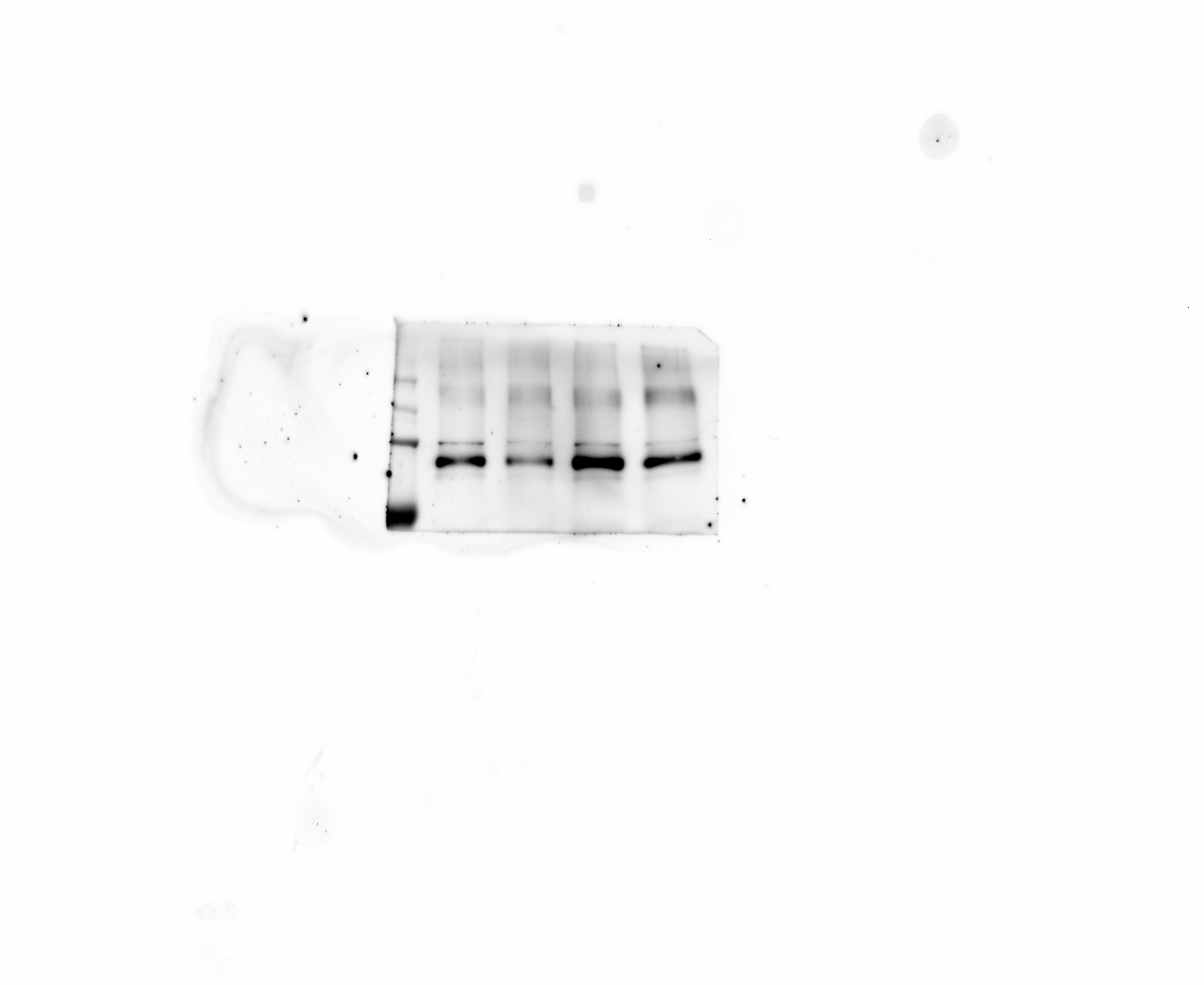

Supplement: Supplementary file 1 — Supplementary file1 (ZIP 36116 KB) [file 432_2024_5625_MOESM1_ESM.zip › Original Images for BlotsGels/5.Figure 5/LN229/4.P-STAT3/1/7.P-STAT3(样品图).tif]

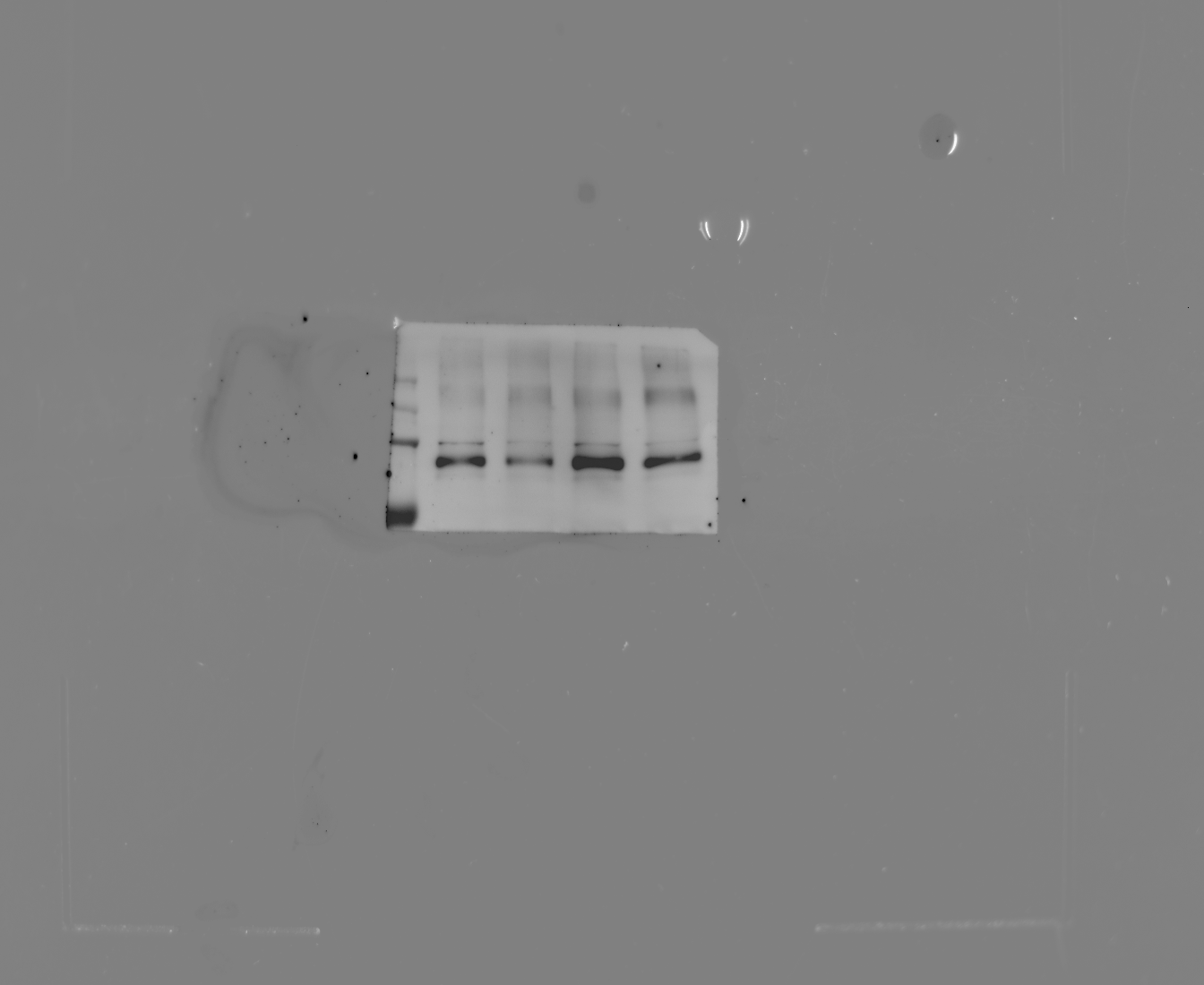

Supplement: Supplementary file 1 — Supplementary file1 (ZIP 36116 KB) [file 432_2024_5625_MOESM1_ESM.zip › Original Images for BlotsGels/5.Figure 5/LN229/4.P-STAT3/1/7.P-STAT3(叠加图).tif]

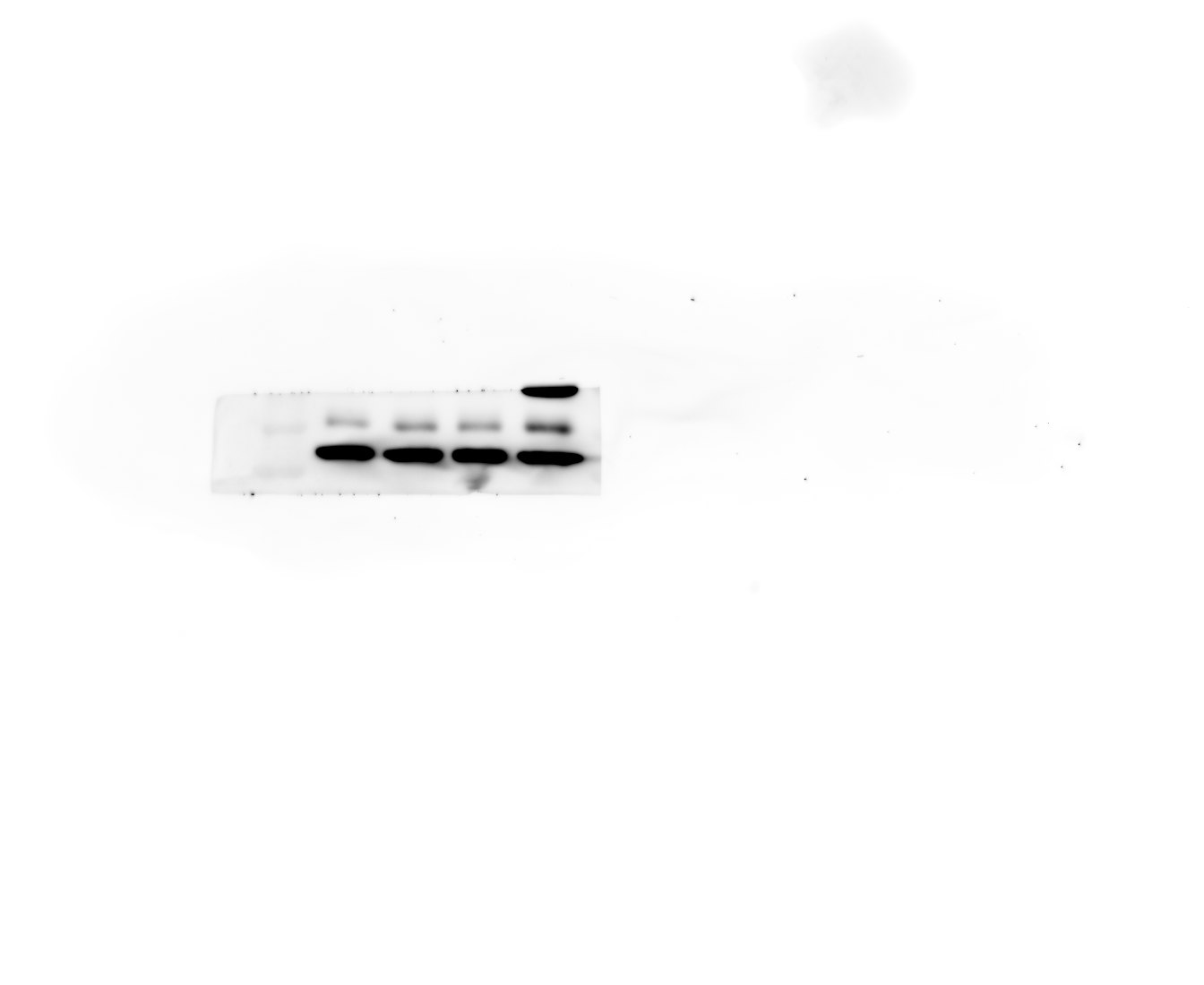

Supplement: Supplementary file 1 — Supplementary file1 (ZIP 36116 KB) [file 432_2024_5625_MOESM1_ESM.zip › Original Images for BlotsGels/5.Figure 5/LN229/5.NLRP3/A(样品图).tif]

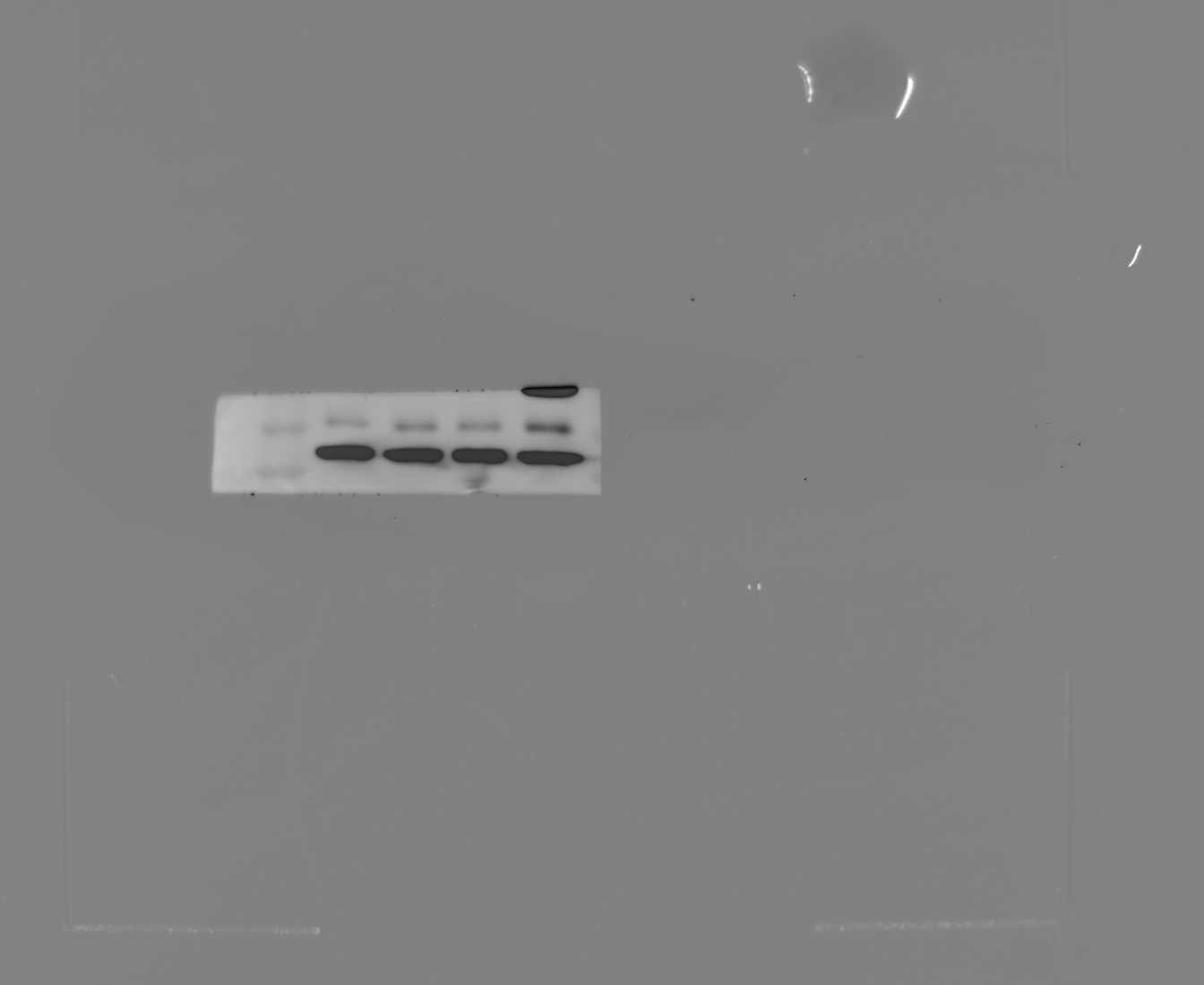

Supplement: Supplementary file 1 — Supplementary file1 (ZIP 36116 KB) [file 432_2024_5625_MOESM1_ESM.zip › Original Images for BlotsGels/5.Figure 5/LN229/5.NLRP3/A(叠加图).tif]

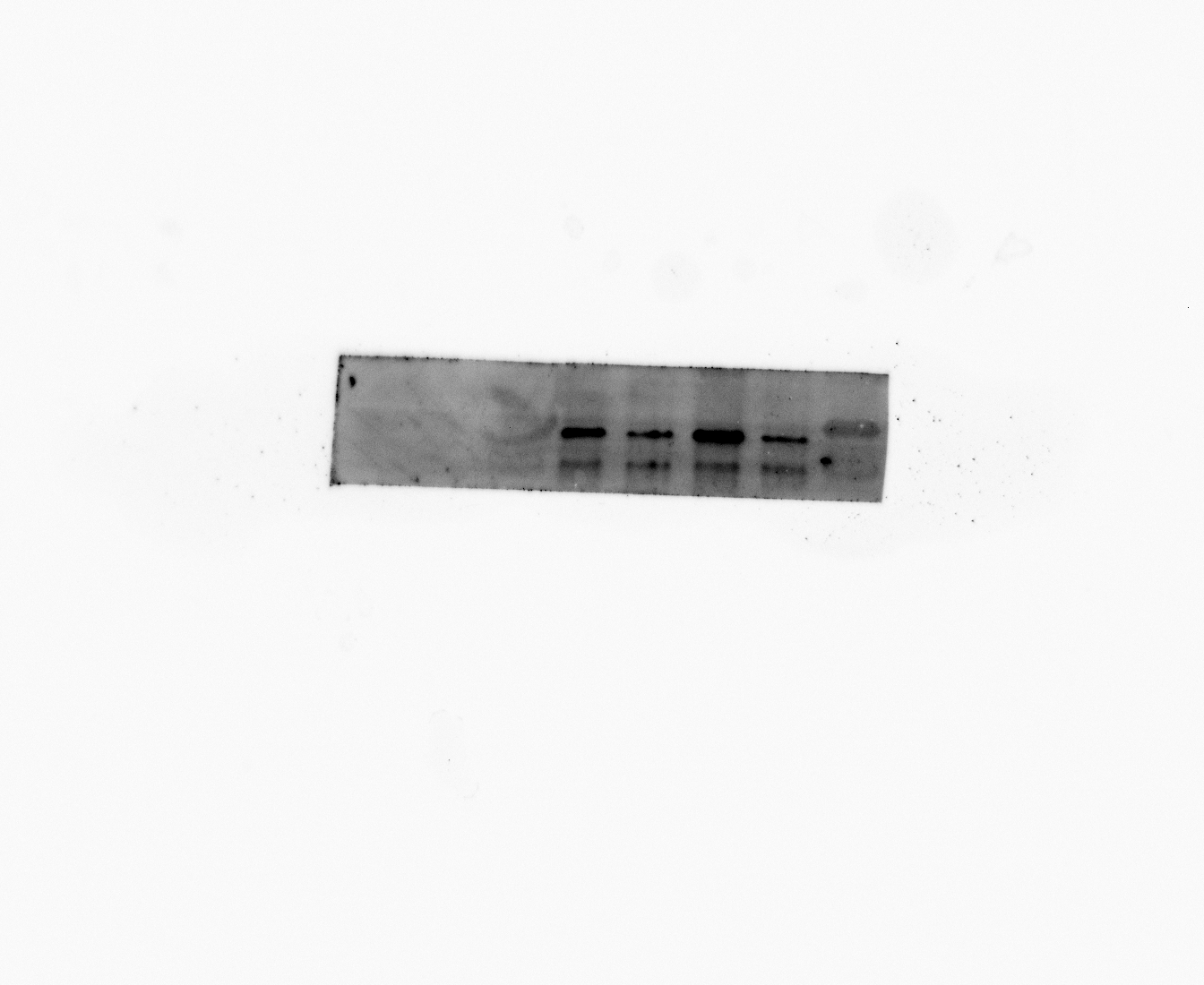

Supplement: Supplementary file 1 — Supplementary file1 (ZIP 36116 KB) [file 432_2024_5625_MOESM1_ESM.zip › Original Images for BlotsGels/5.Figure 5/LN229/5.NLRP3/NLRP3(Y).tif]

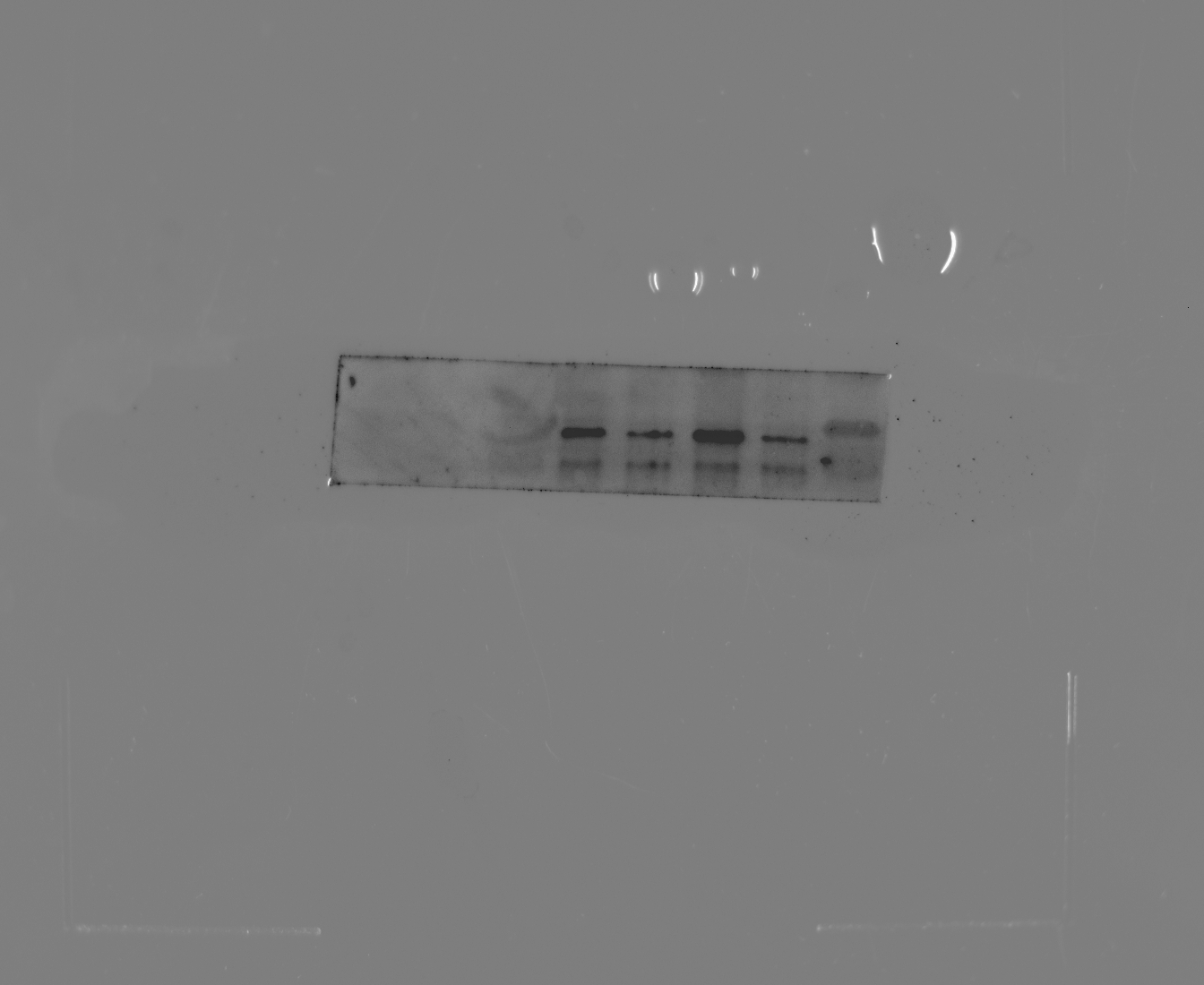

Supplement: Supplementary file 1 — Supplementary file1 (ZIP 36116 KB) [file 432_2024_5625_MOESM1_ESM.zip › Original Images for BlotsGels/5.Figure 5/LN229/5.NLRP3/NLRP3.tif]

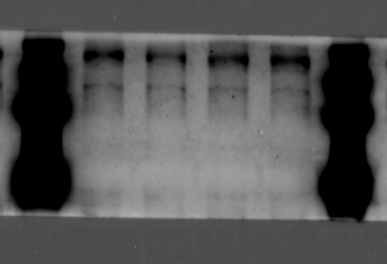

Supplement: Supplementary file 1 — Supplementary file1 (ZIP 36116 KB) [file 432_2024_5625_MOESM1_ESM.zip › Original Images for BlotsGels/5.Figure 5/U87/1.JAK2/1/JAK2.tif]

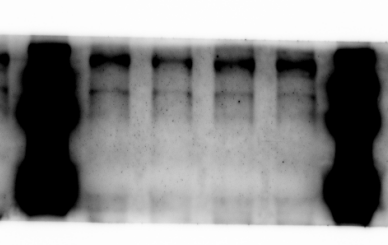

Supplement: Supplementary file 1 — Supplementary file1 (ZIP 36116 KB) [file 432_2024_5625_MOESM1_ESM.zip › Original Images for BlotsGels/5.Figure 5/U87/1.JAK2/1/JAK2(Y).tif]

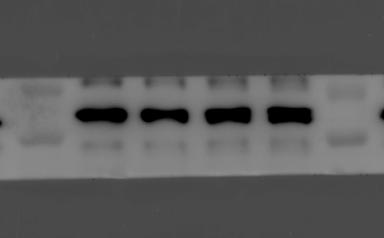

Supplement: Supplementary file 1 — Supplementary file1 (ZIP 36116 KB) [file 432_2024_5625_MOESM1_ESM.zip › Original Images for BlotsGels/5.Figure 5/U87/1.JAK2/1/β-ACTIN.tif]

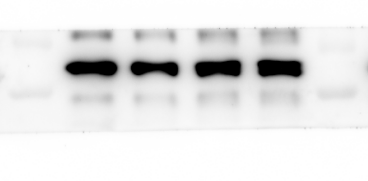

Supplement: Supplementary file 1 — Supplementary file1 (ZIP 36116 KB) [file 432_2024_5625_MOESM1_ESM.zip › Original Images for BlotsGels/5.Figure 5/U87/1.JAK2/1/β-ACTIN(y).tif]

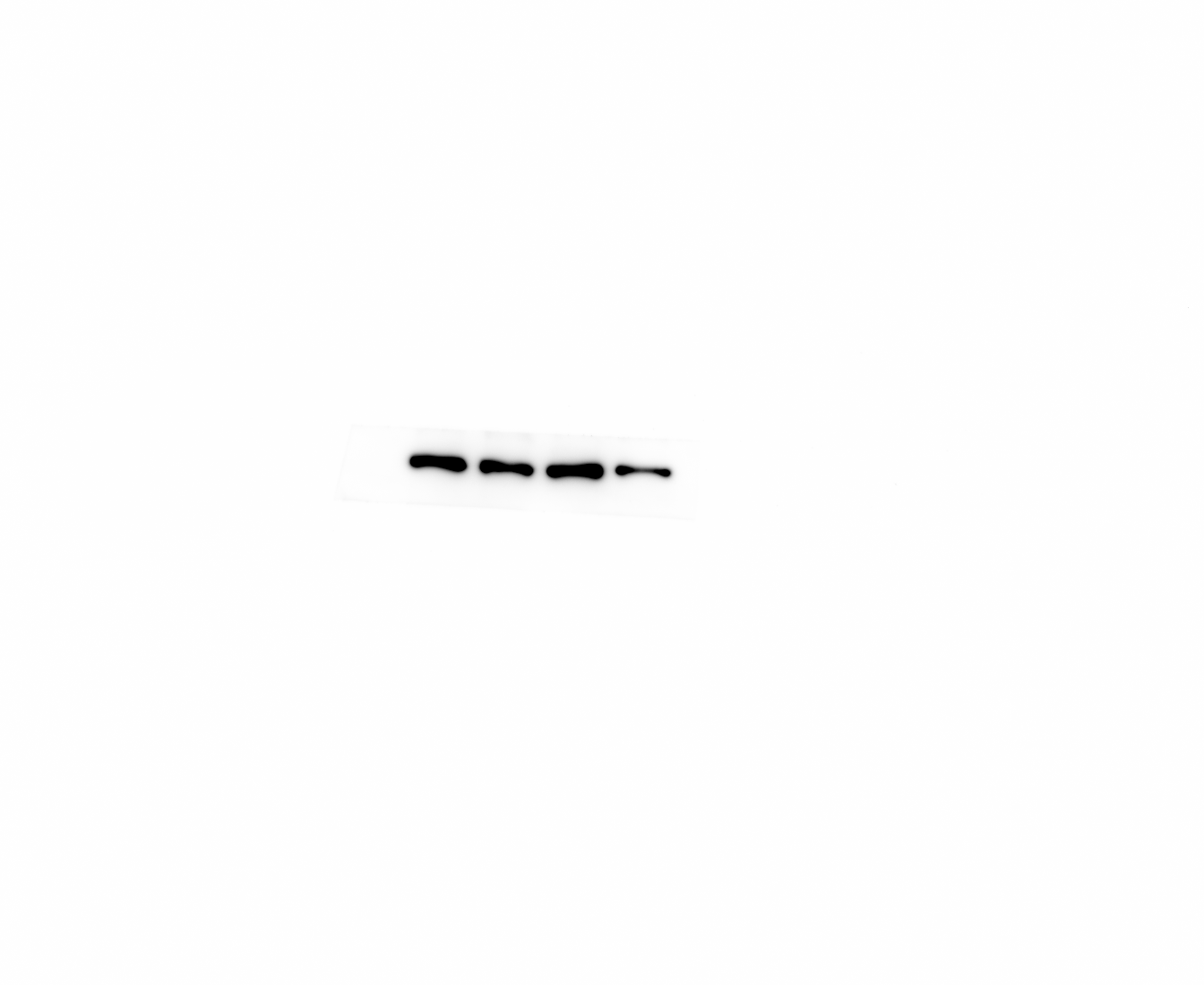

Supplement: Supplementary file 1 — Supplementary file1 (ZIP 36116 KB) [file 432_2024_5625_MOESM1_ESM.zip › Original Images for BlotsGels/5.Figure 5/U87/2.P-JAK2/1/1-1-A(Y).tif]

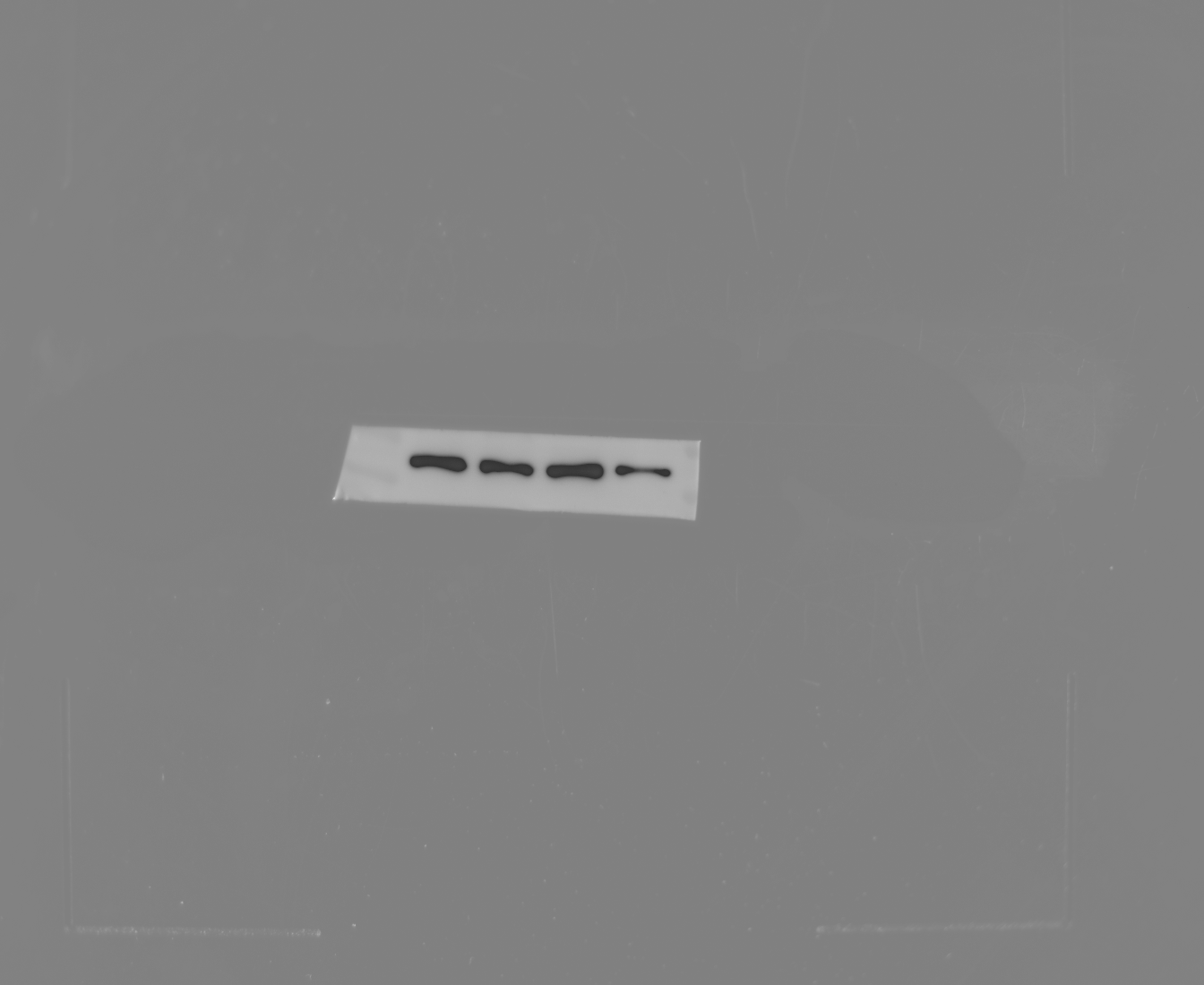

Supplement: Supplementary file 1 — Supplementary file1 (ZIP 36116 KB) [file 432_2024_5625_MOESM1_ESM.zip › Original Images for BlotsGels/5.Figure 5/U87/2.P-JAK2/1/1-1-A.tif]

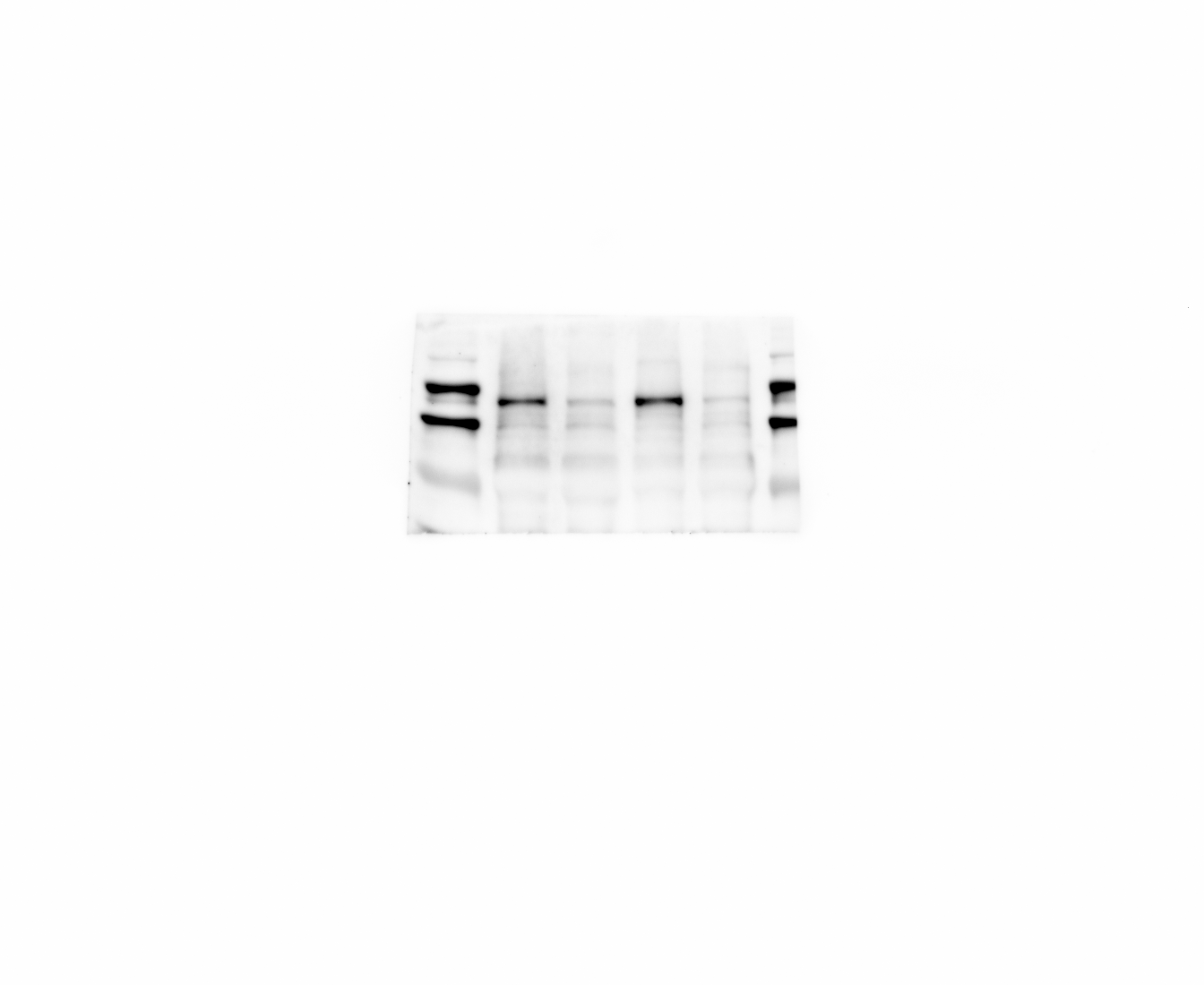

Supplement: Supplementary file 1 — Supplementary file1 (ZIP 36116 KB) [file 432_2024_5625_MOESM1_ESM.zip › Original Images for BlotsGels/5.Figure 5/U87/2.P-JAK2/1/1-1-P-JAK2(Y).tif]

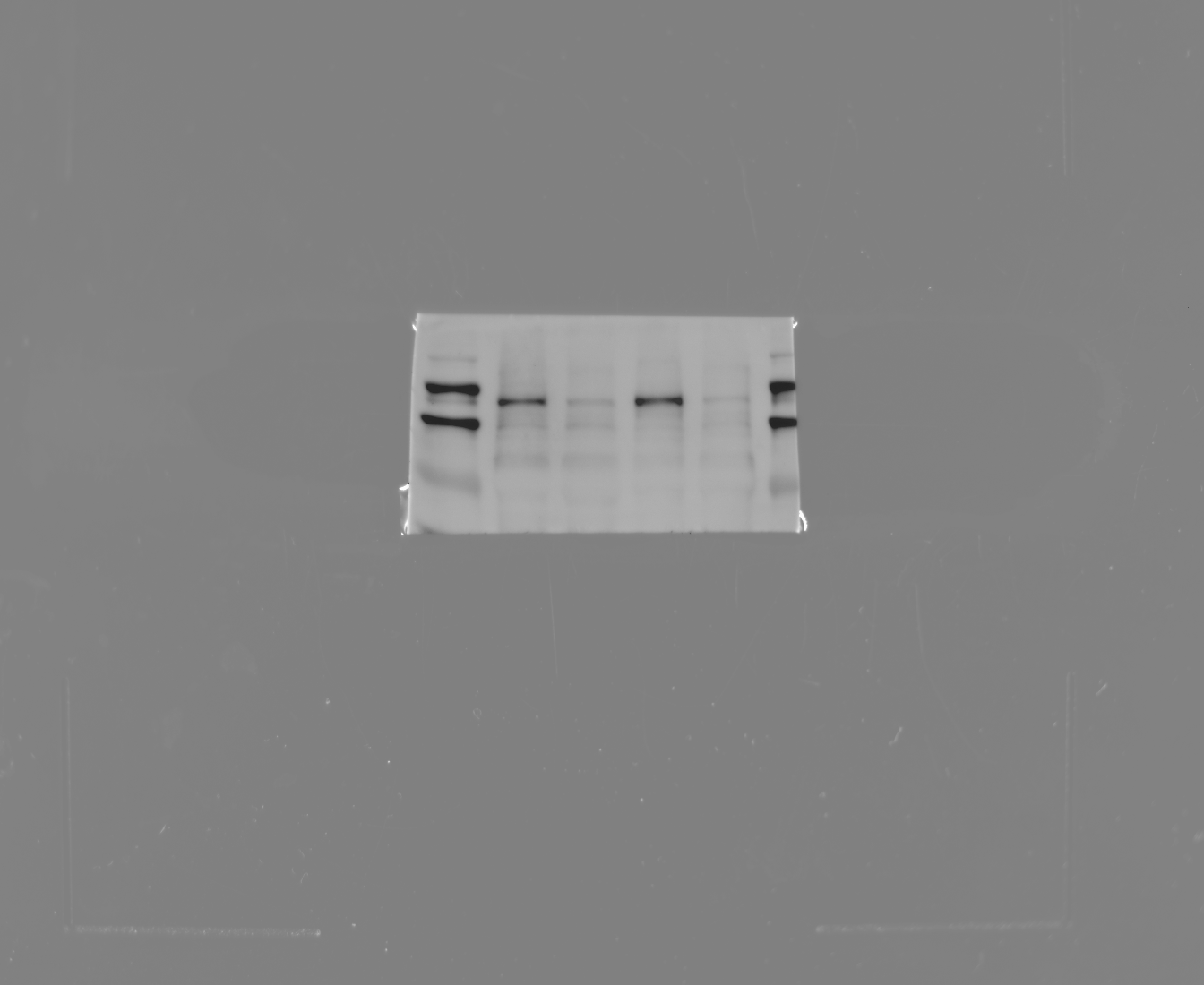

Supplement: Supplementary file 1 — Supplementary file1 (ZIP 36116 KB) [file 432_2024_5625_MOESM1_ESM.zip › Original Images for BlotsGels/5.Figure 5/U87/2.P-JAK2/1/1-1-P-JAK2.tif]

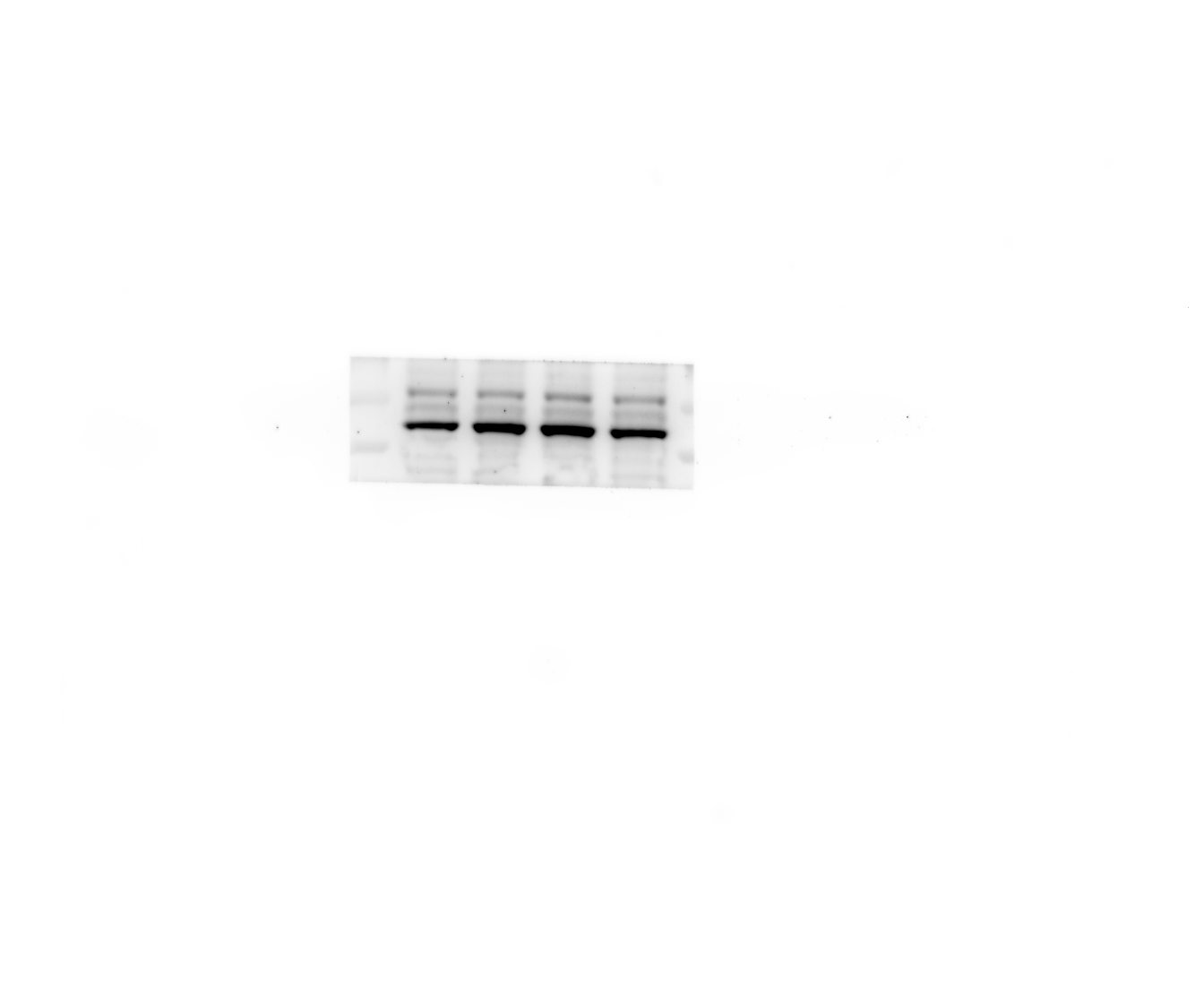

Supplement: Supplementary file 1 — Supplementary file1 (ZIP 36116 KB) [file 432_2024_5625_MOESM1_ESM.zip › Original Images for BlotsGels/5.Figure 5/U87/3.STAT3/1/4-2-A(Y).tif]

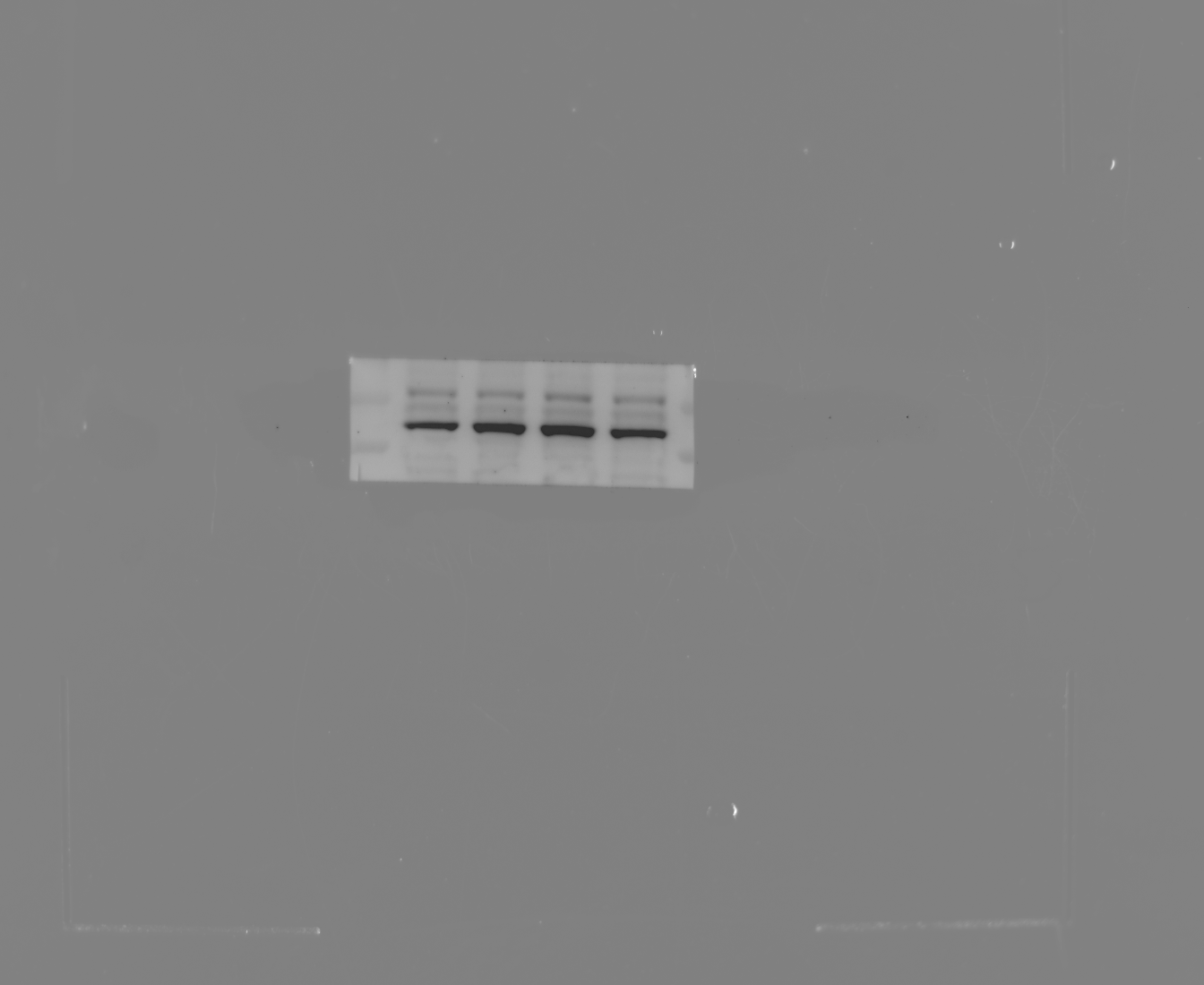

Supplement: Supplementary file 1 — Supplementary file1 (ZIP 36116 KB) [file 432_2024_5625_MOESM1_ESM.zip › Original Images for BlotsGels/5.Figure 5/U87/3.STAT3/1/4-2-A.tif]

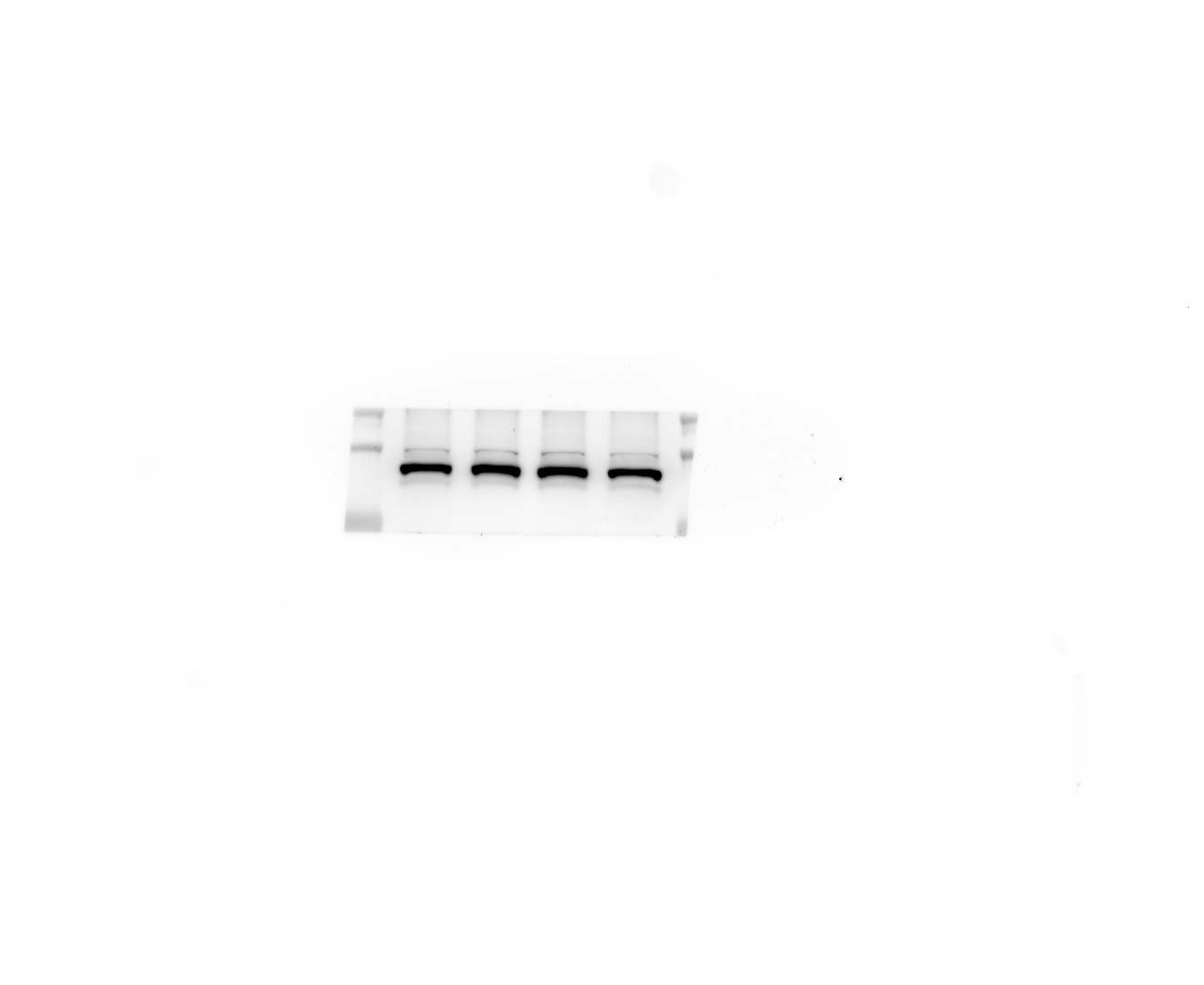

Supplement: Supplementary file 1 — Supplementary file1 (ZIP 36116 KB) [file 432_2024_5625_MOESM1_ESM.zip › Original Images for BlotsGels/5.Figure 5/U87/3.STAT3/1/4-2-STAT3(Y).tif]

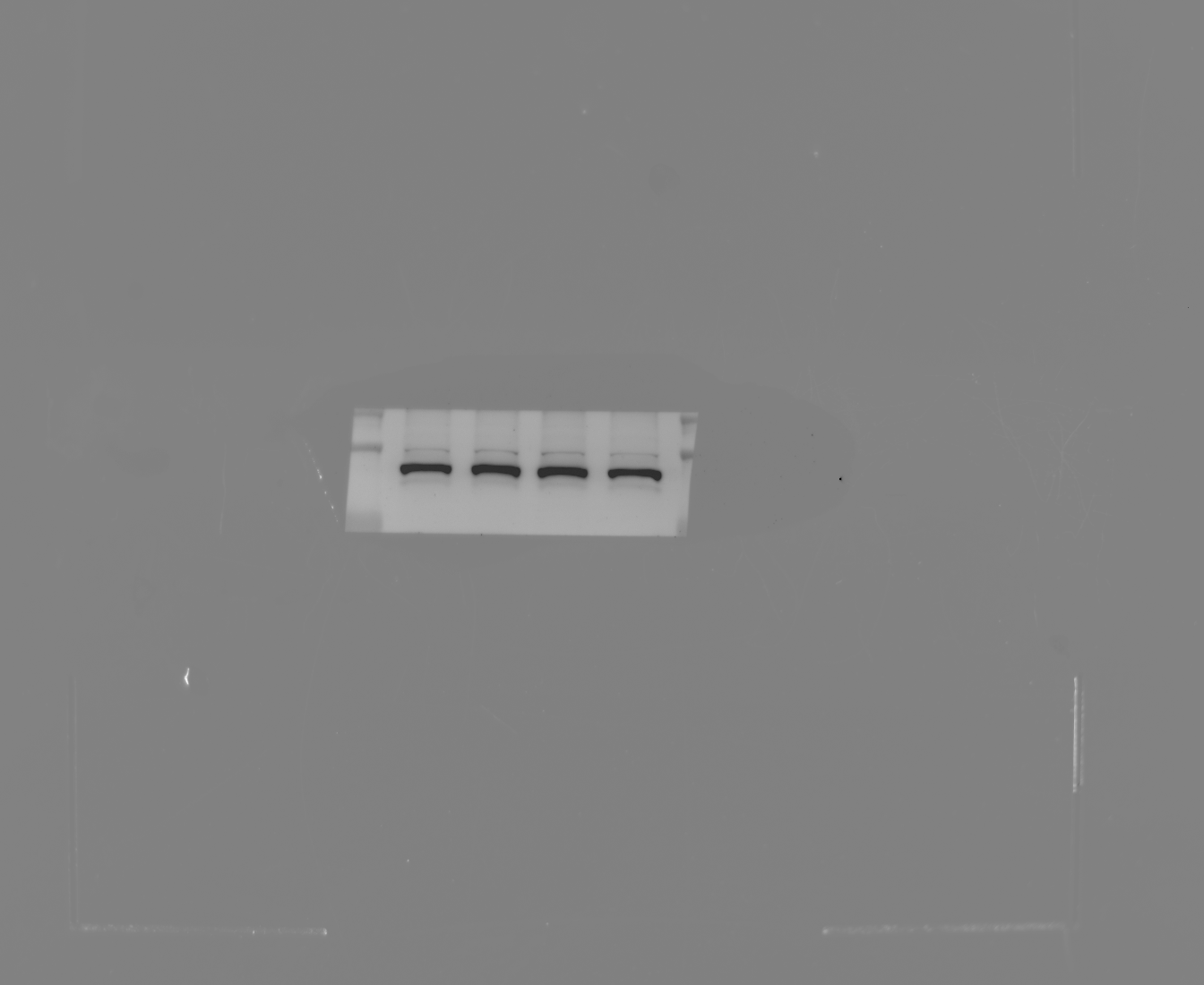

Supplement: Supplementary file 1 — Supplementary file1 (ZIP 36116 KB) [file 432_2024_5625_MOESM1_ESM.zip › Original Images for BlotsGels/5.Figure 5/U87/3.STAT3/1/4-2-STAT3.tif]

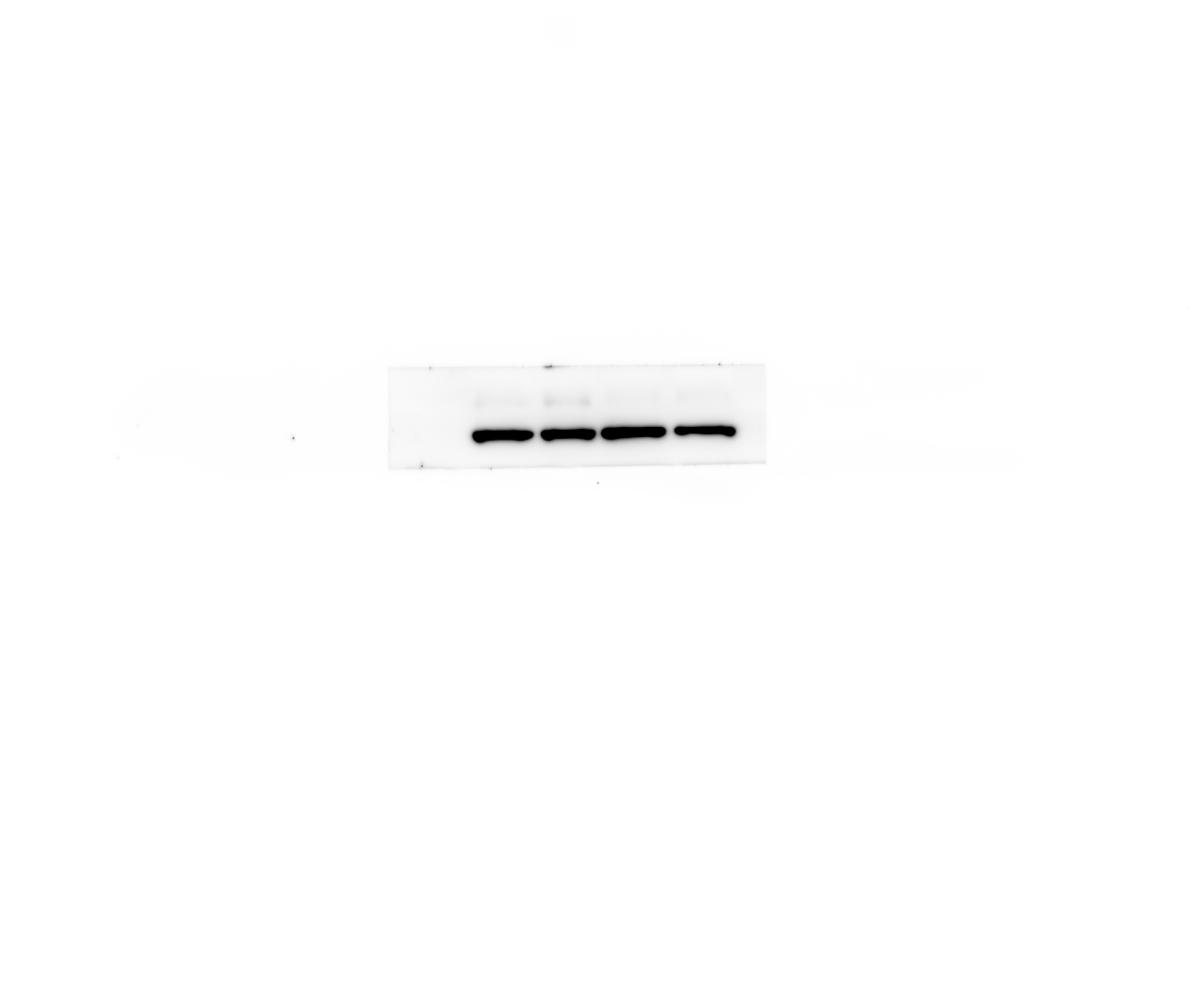

Supplement: Supplementary file 1 — Supplementary file1 (ZIP 36116 KB) [file 432_2024_5625_MOESM1_ESM.zip › Original Images for BlotsGels/5.Figure 5/U87/4.P-STAT3/1/2-1-A(Y).tif]

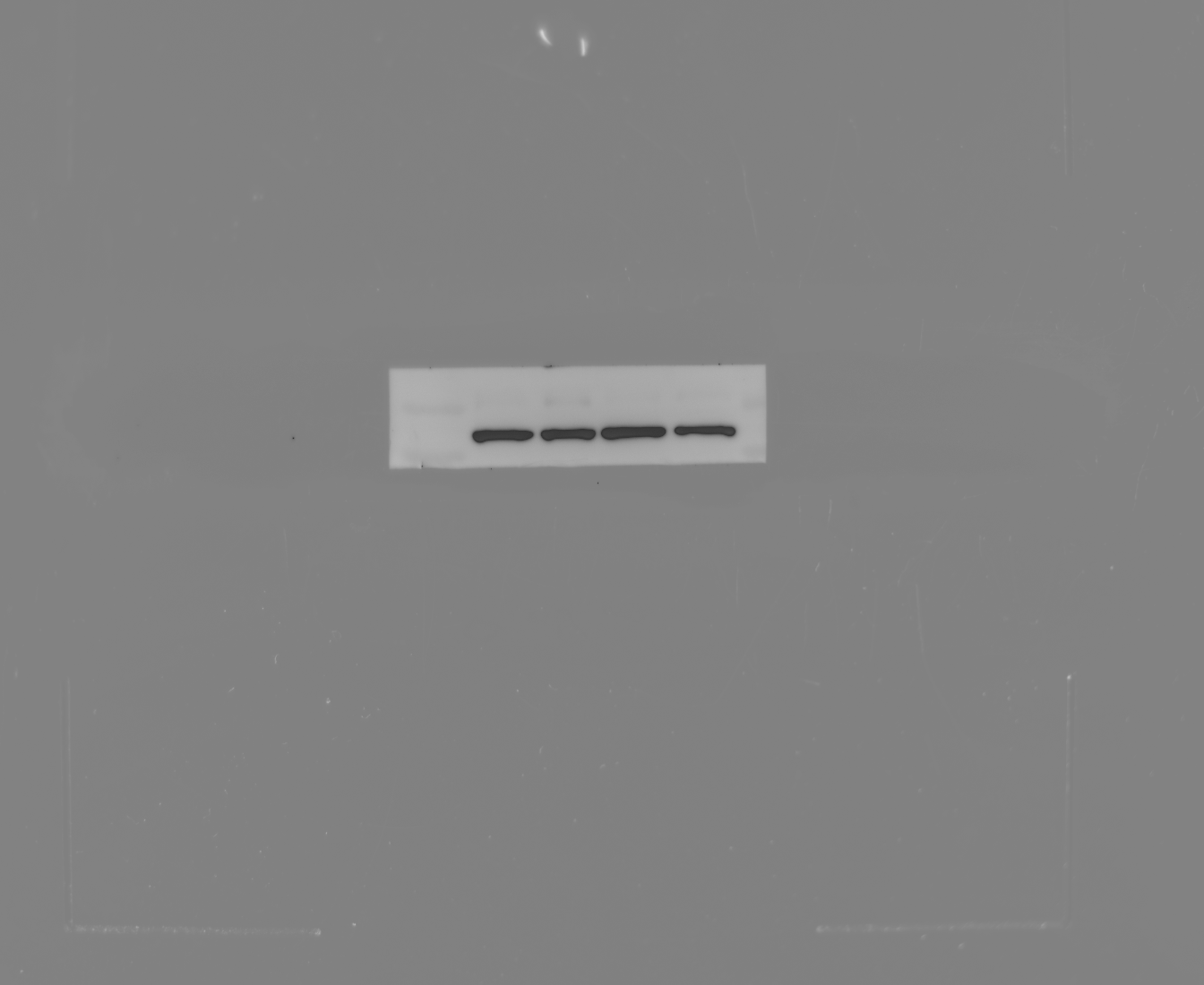

Supplement: Supplementary file 1 — Supplementary file1 (ZIP 36116 KB) [file 432_2024_5625_MOESM1_ESM.zip › Original Images for BlotsGels/5.Figure 5/U87/4.P-STAT3/1/2-1-A.tif]

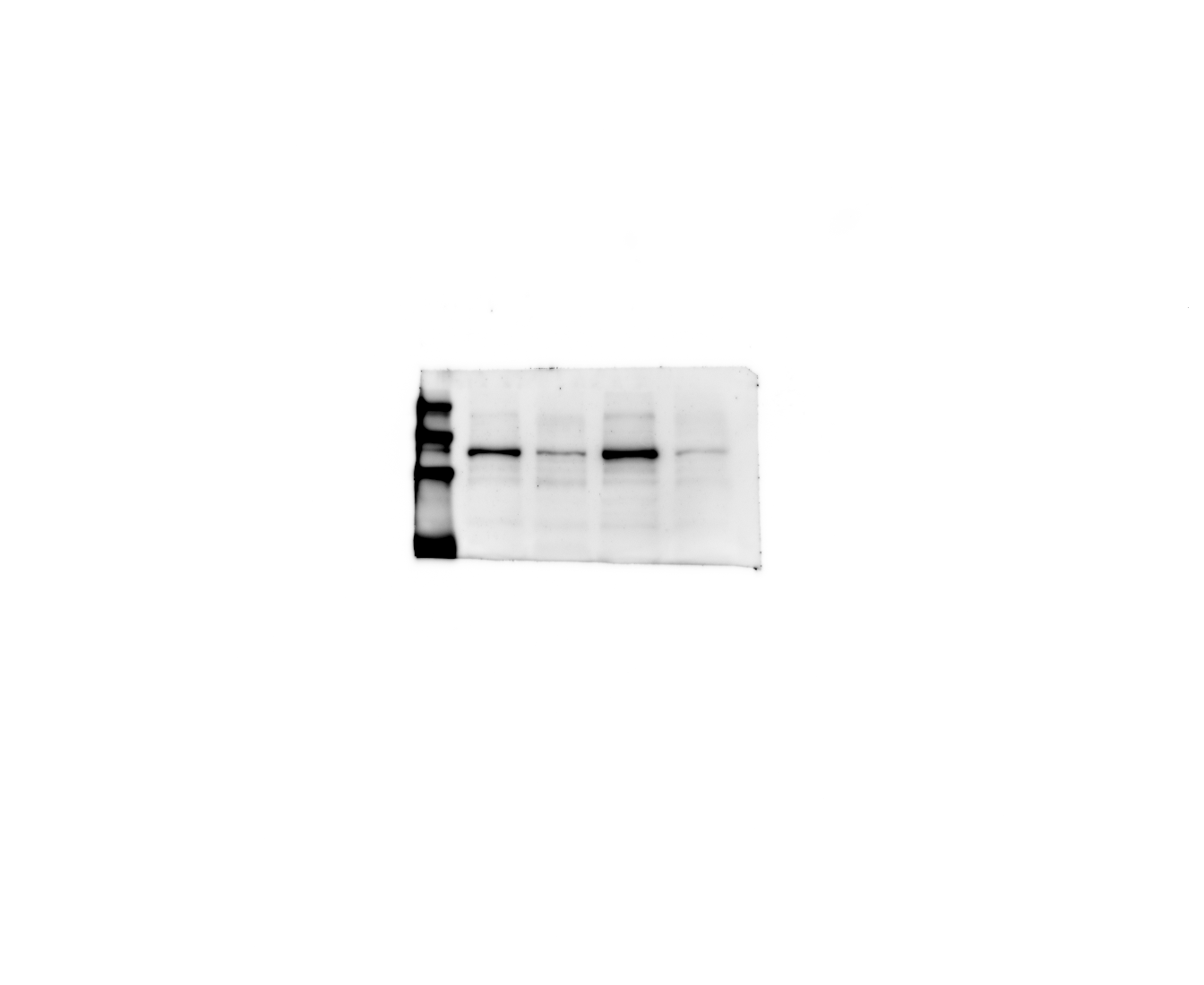

Supplement: Supplementary file 1 — Supplementary file1 (ZIP 36116 KB) [file 432_2024_5625_MOESM1_ESM.zip › Original Images for BlotsGels/5.Figure 5/U87/4.P-STAT3/1/2-1-P-JAK2(Y).tif]

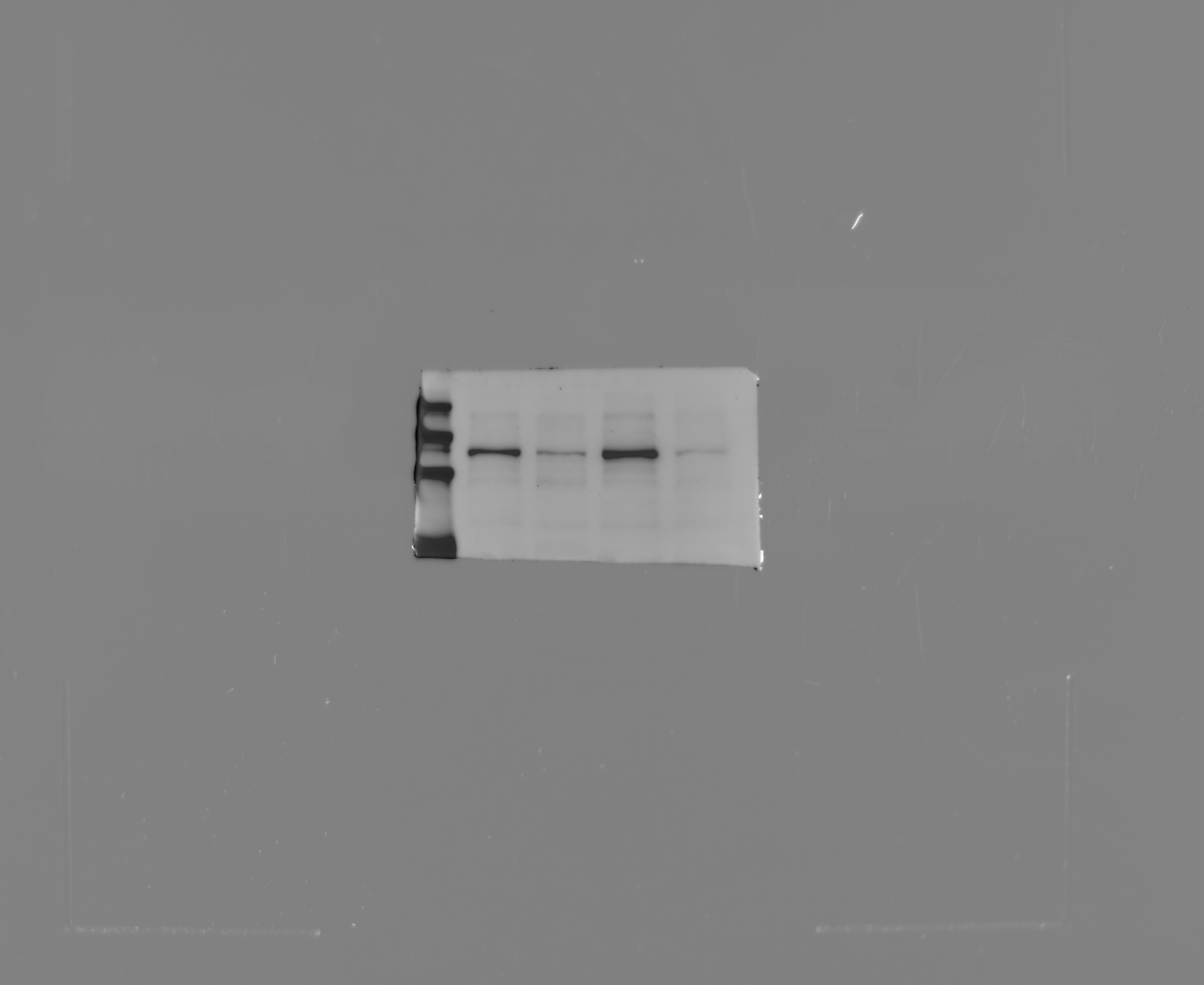

Supplement: Supplementary file 1 — Supplementary file1 (ZIP 36116 KB) [file 432_2024_5625_MOESM1_ESM.zip › Original Images for BlotsGels/5.Figure 5/U87/4.P-STAT3/1/2-1-P-JAK2.tif]

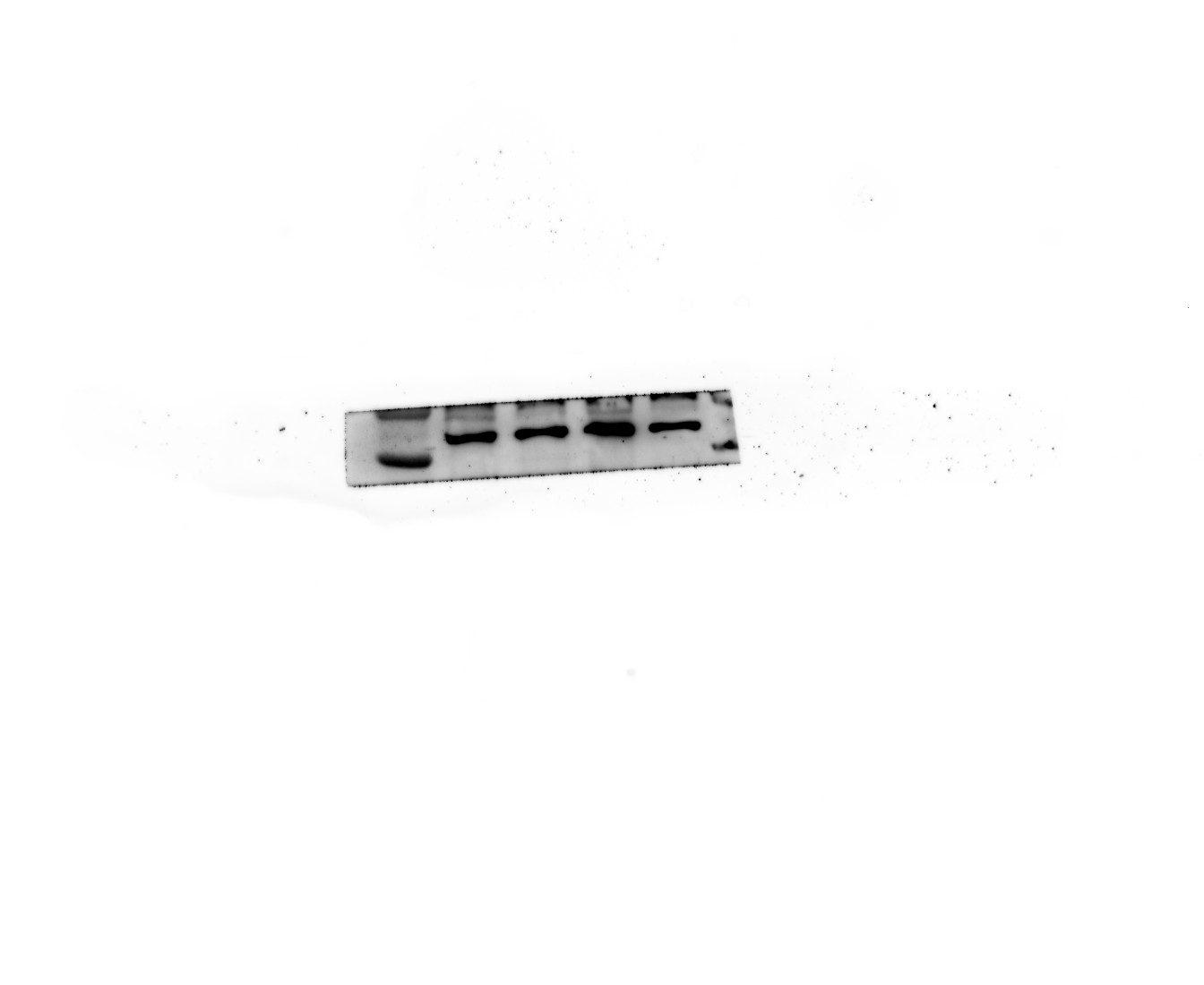

Supplement: Supplementary file 1 — Supplementary file1 (ZIP 36116 KB) [file 432_2024_5625_MOESM1_ESM.zip › Original Images for BlotsGels/5.Figure 5/U87/5.NLRP3/A(样品图).tif]

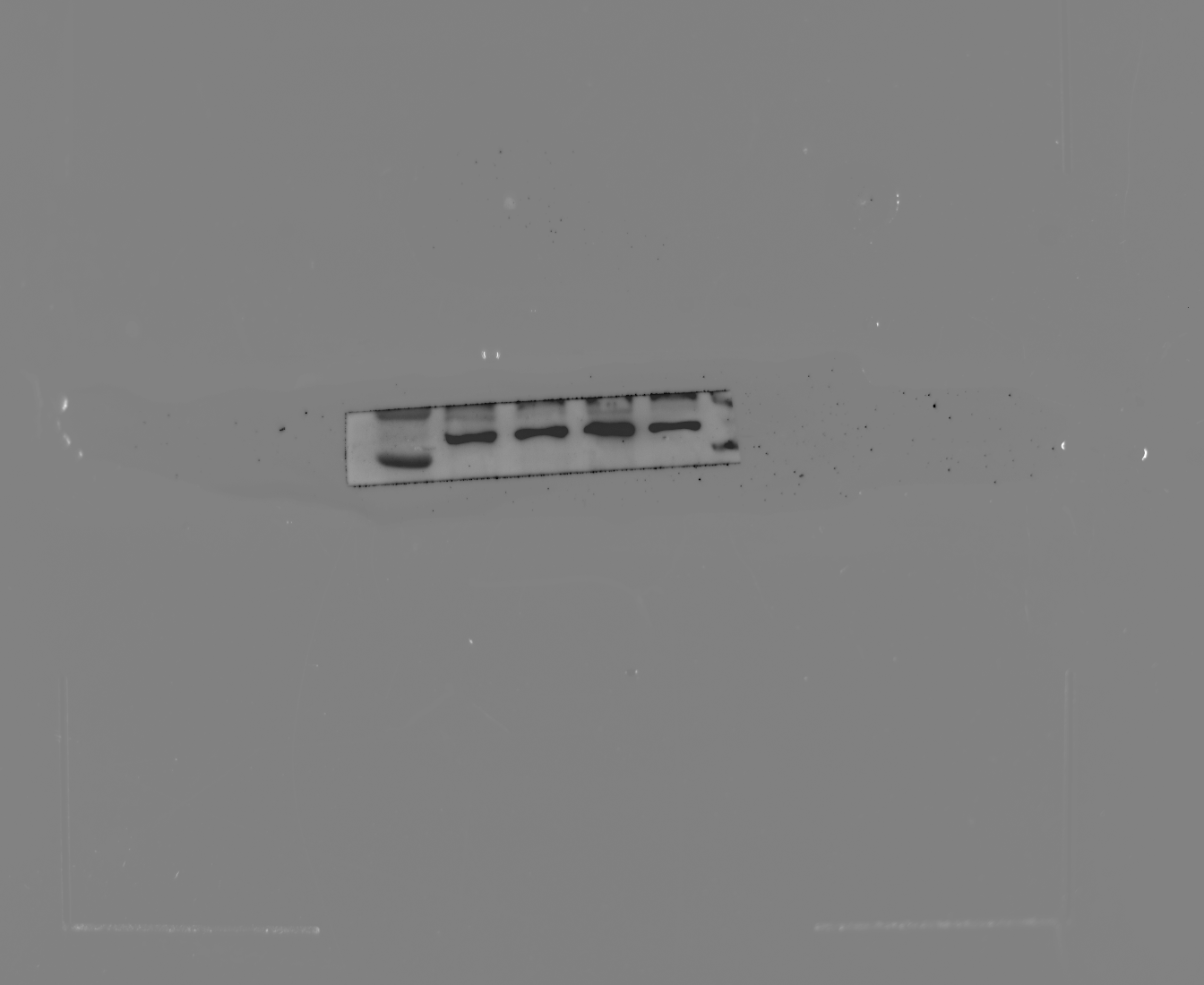

Supplement: Supplementary file 1 — Supplementary file1 (ZIP 36116 KB) [file 432_2024_5625_MOESM1_ESM.zip › Original Images for BlotsGels/5.Figure 5/U87/5.NLRP3/A(叠加图).tif]

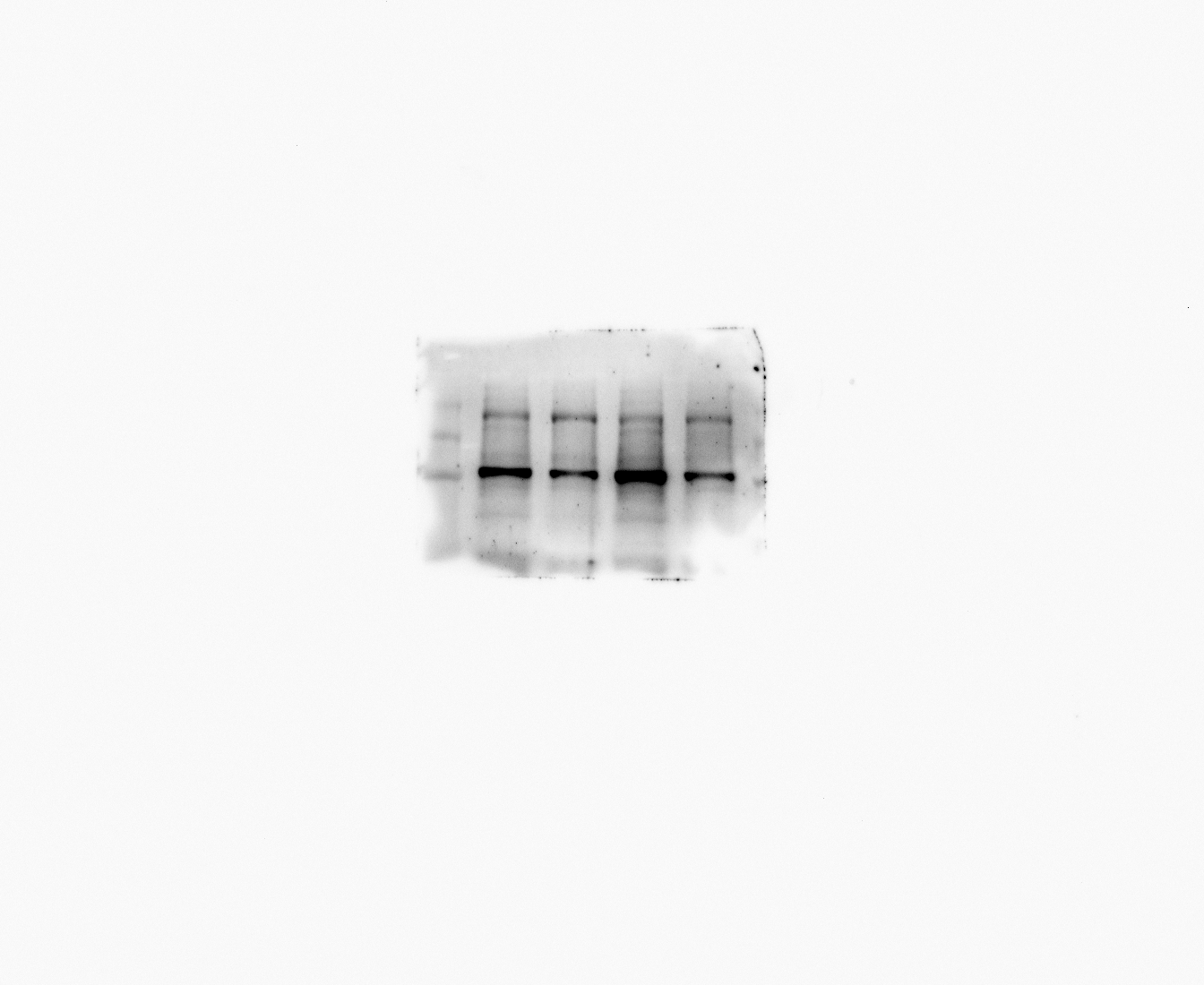

Supplement: Supplementary file 1 — Supplementary file1 (ZIP 36116 KB) [file 432_2024_5625_MOESM1_ESM.zip › Original Images for BlotsGels/5.Figure 5/U87/5.NLRP3/NLRP3(Y).tif]

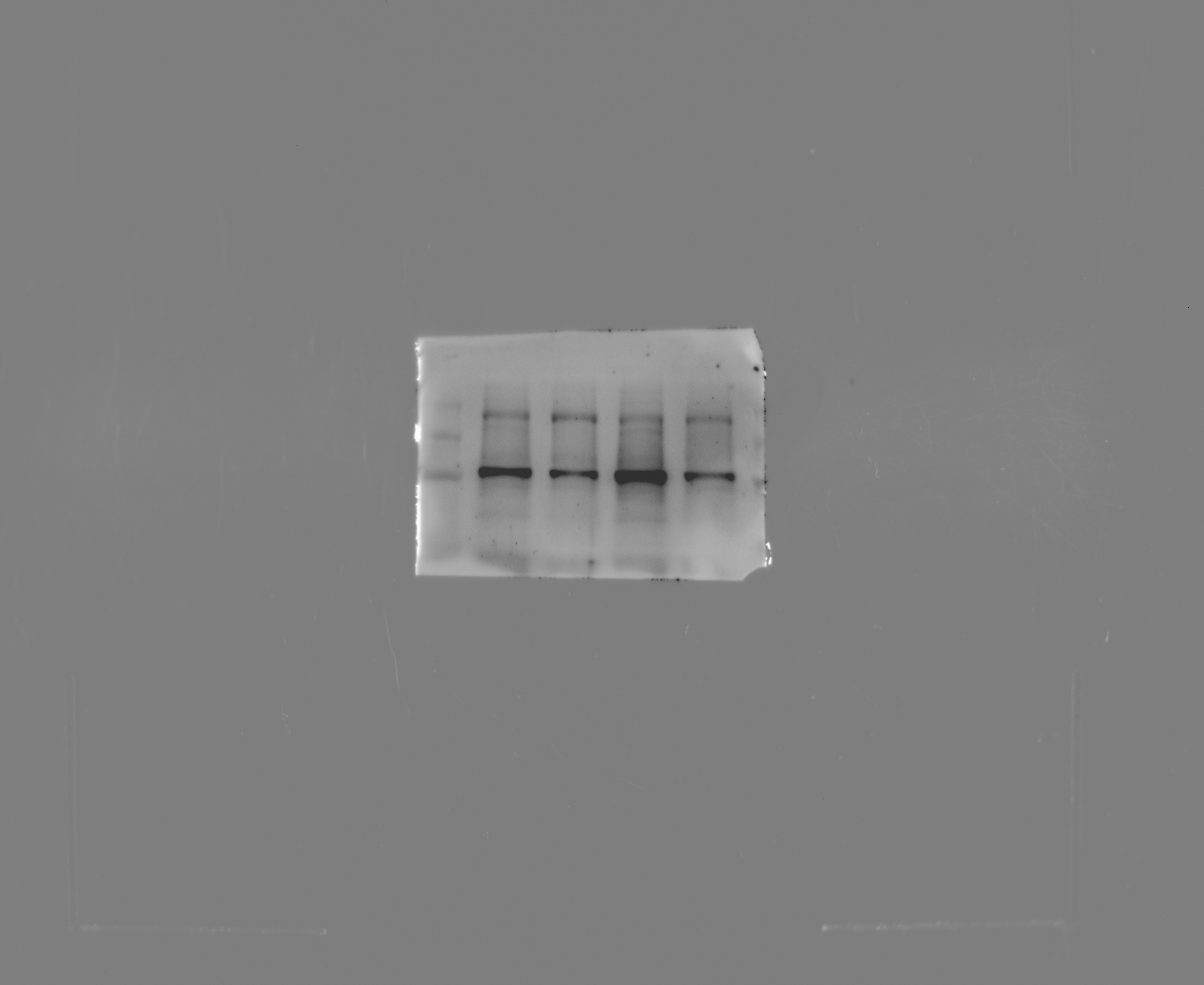

Supplement: Supplementary file 1 — Supplementary file1 (ZIP 36116 KB) [file 432_2024_5625_MOESM1_ESM.zip › Original Images for BlotsGels/5.Figure 5/U87/5.NLRP3/NLRP3.tif]

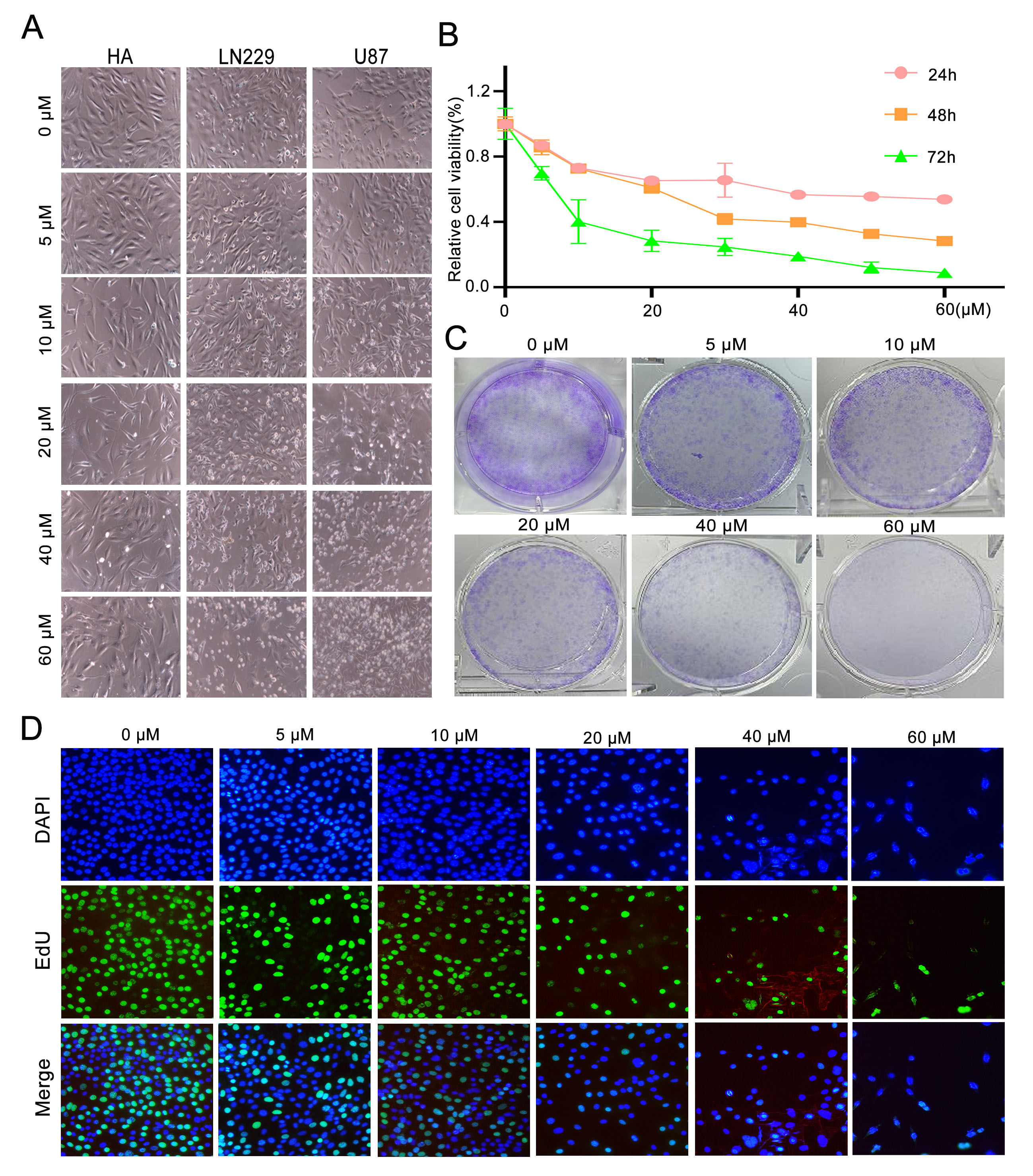

Supplement: Supplementary file 3 — Supplementary file3 Supplementary Figure S1 RSV improves the malignant progression of GBM. A, Cellular morphology of HA (astrocyte cell line), LN-229, and U87-MG under light microscope after 48 hours of treatment with various concentrations of RSV; B, Assessment of cell viability in U87-MG cell line treated with different concentrations of RSV for different periods of time using CCK8 assay; C, Colony formation assay to evaluate the impact of RSV treatment at different concentrations on GBM proliferation; D, EdU proliferation assay conducted on LN-229 cell line treated with various concentrations of RSV for 48 hours (TIF 22541 KB) [file 432_2024_5625_MOESM3_ESM.tif]
