# Supplementary figures and images for: Resveratrol ameliorates glioblastoma inflammatory response by reducing NLRP3 inflammasome activation through inhibition of the JAK2/STAT3 pathway (part 1 of 2)
Source: J Cancer Res Clin Oncol. 2024 Mar 28;150(3):168. doi: 10.1007/s00432-024-05625-5 (PMC10978631; doi:10.1007/s00432-024-05625-5)

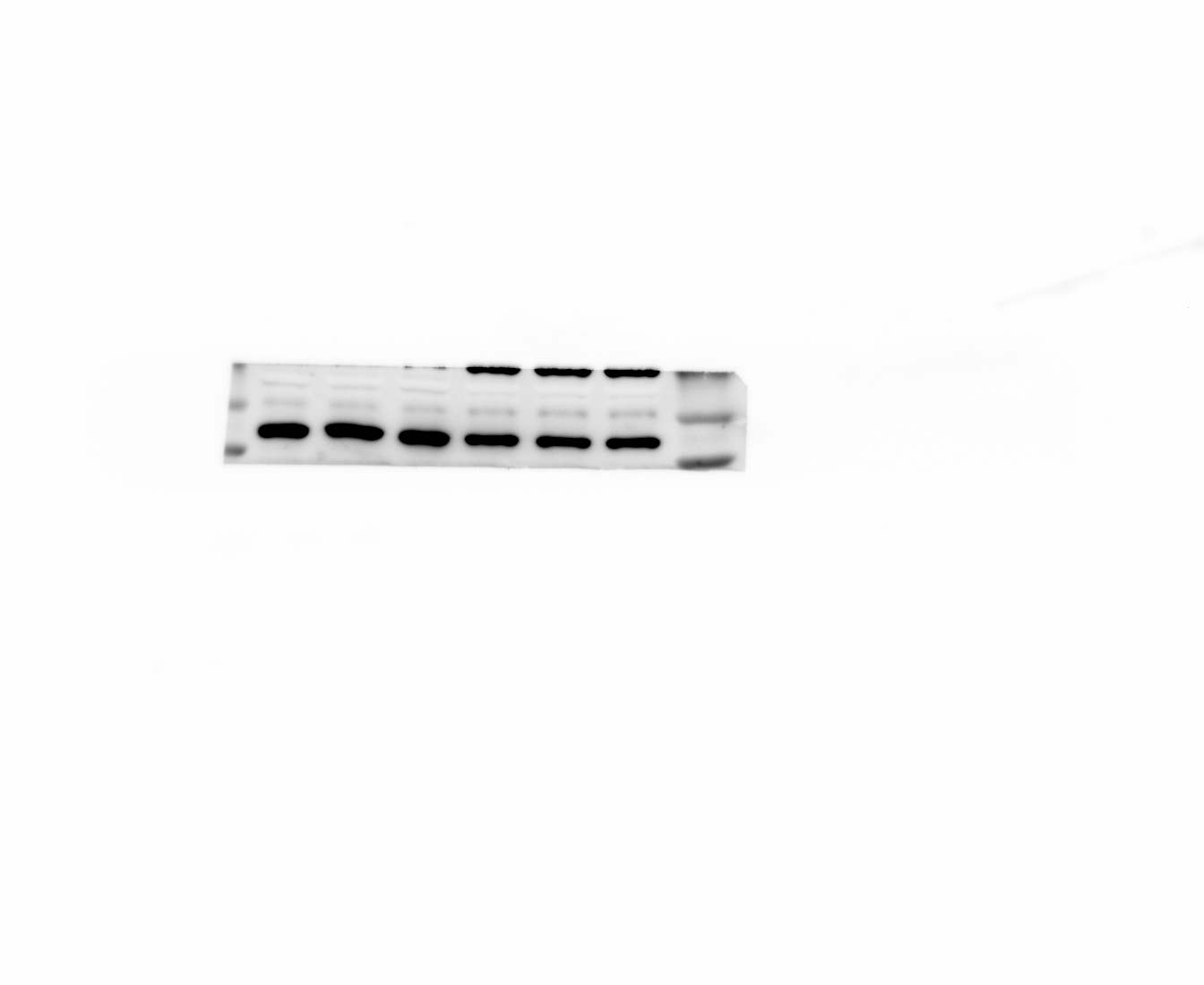

Supplement: Supplementary file 1 — Supplementary file1 (ZIP 36116 KB) [file 432_2024_5625_MOESM1_ESM.zip › Original Images for BlotsGels/1.Figure 1/E-cadherin/1/1-2-A(Y).tif]

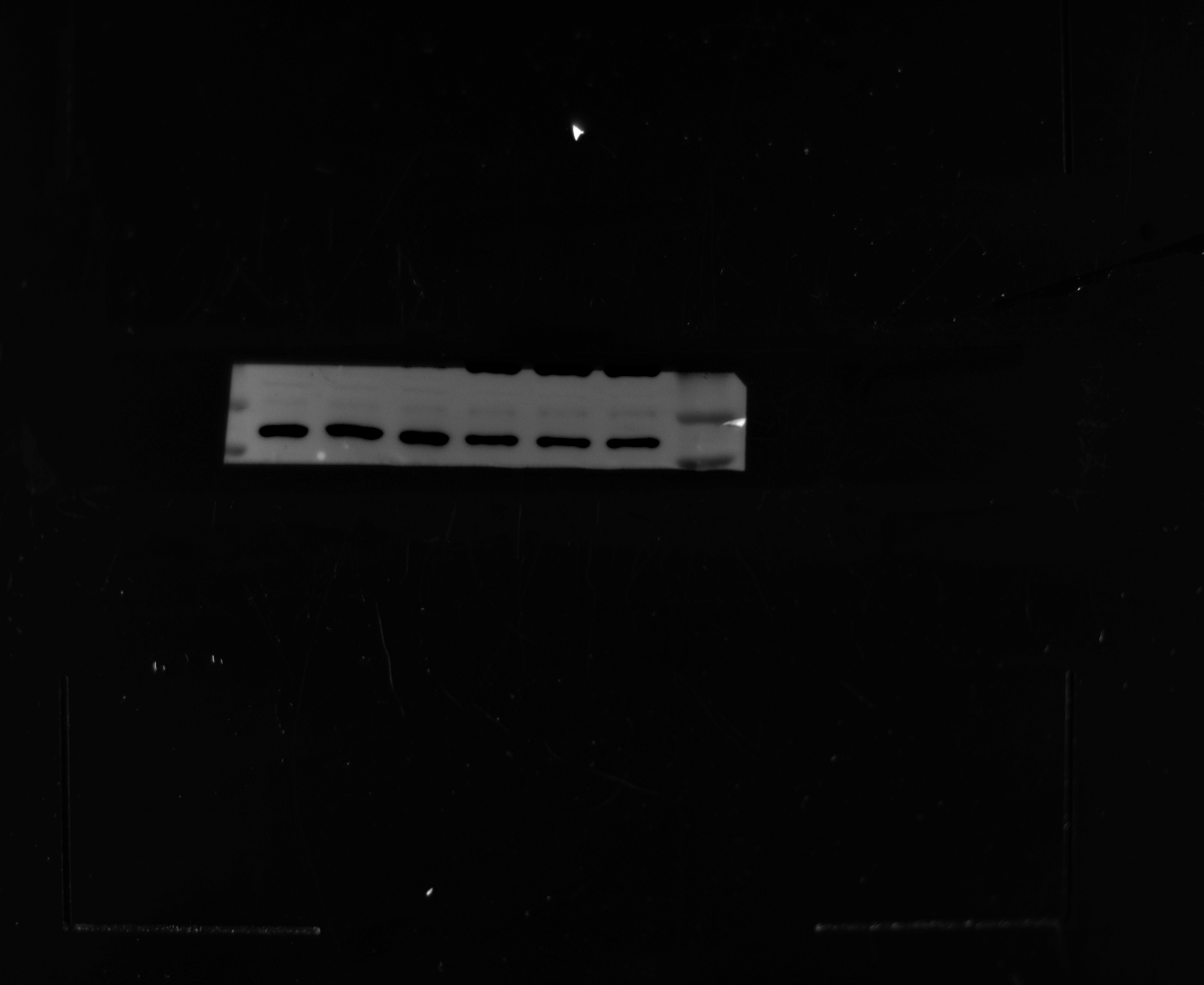

Supplement: Supplementary file 1 — Supplementary file1 (ZIP 36116 KB) [file 432_2024_5625_MOESM1_ESM.zip › Original Images for BlotsGels/1.Figure 1/E-cadherin/1/1-2-A.tif]

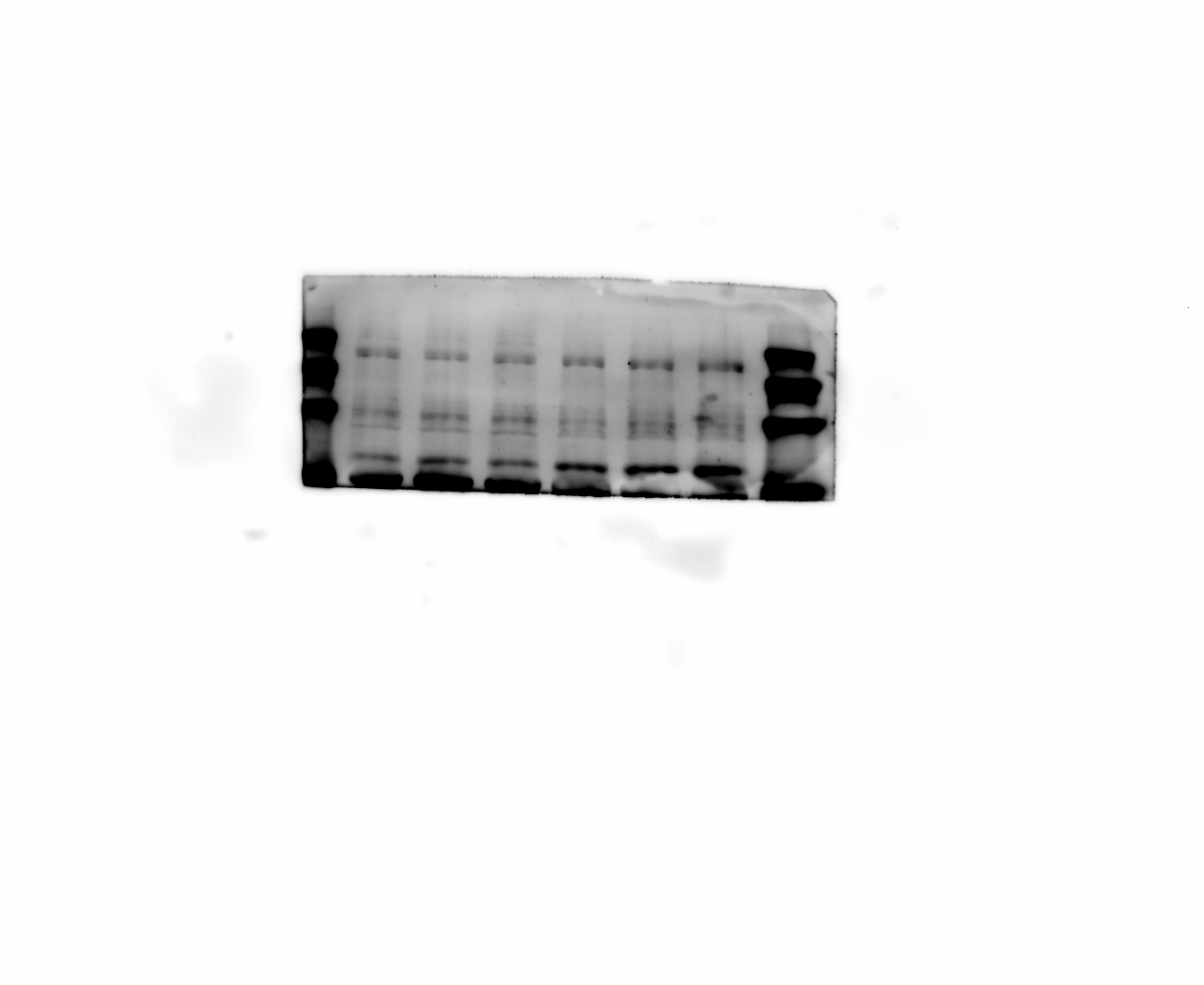

Supplement: Supplementary file 1 — Supplementary file1 (ZIP 36116 KB) [file 432_2024_5625_MOESM1_ESM.zip › Original Images for BlotsGels/1.Figure 1/E-cadherin/1/1-2-E-CA(Y).tif]

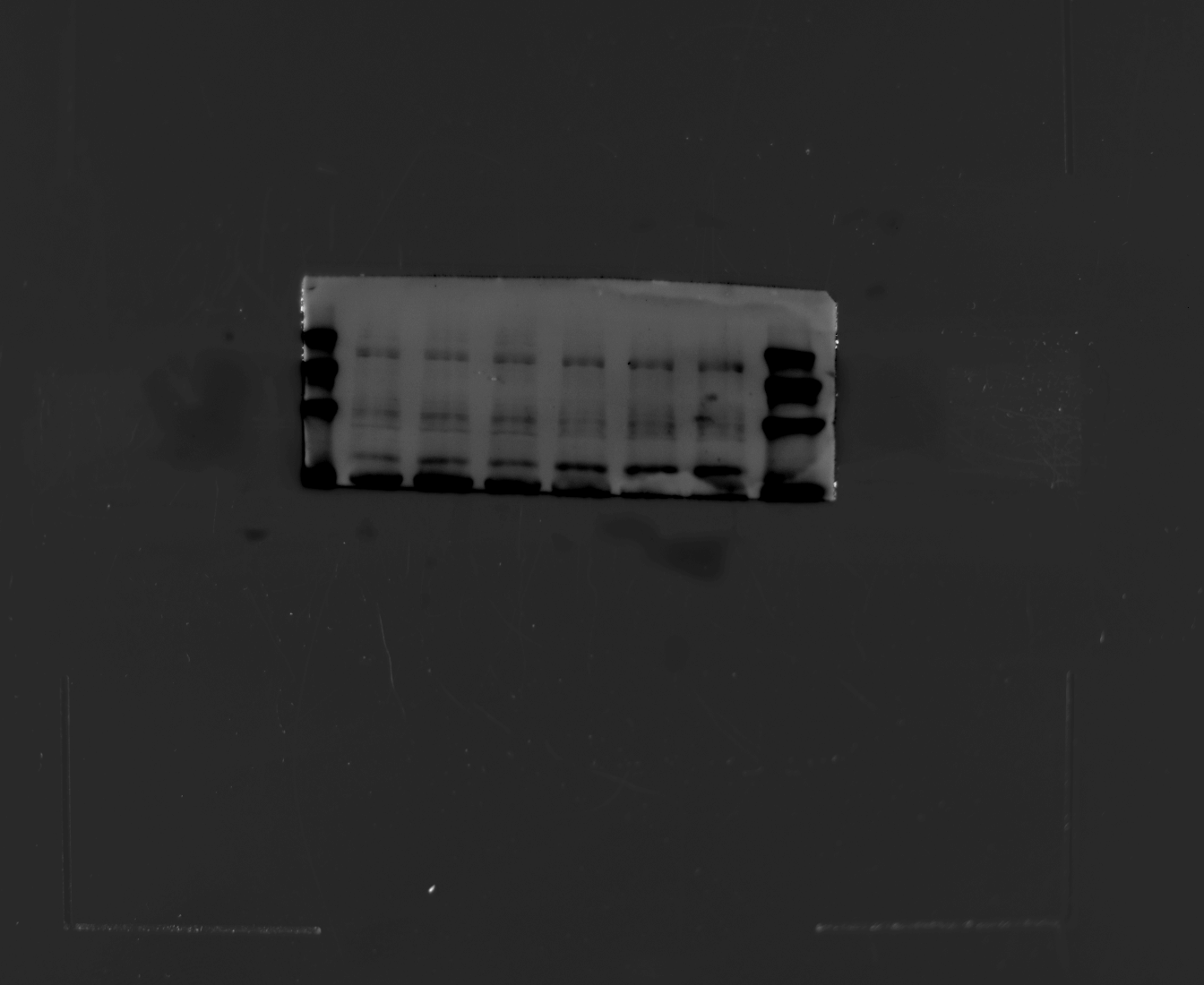

Supplement: Supplementary file 1 — Supplementary file1 (ZIP 36116 KB) [file 432_2024_5625_MOESM1_ESM.zip › Original Images for BlotsGels/1.Figure 1/E-cadherin/1/1-2-E-CA.tif]

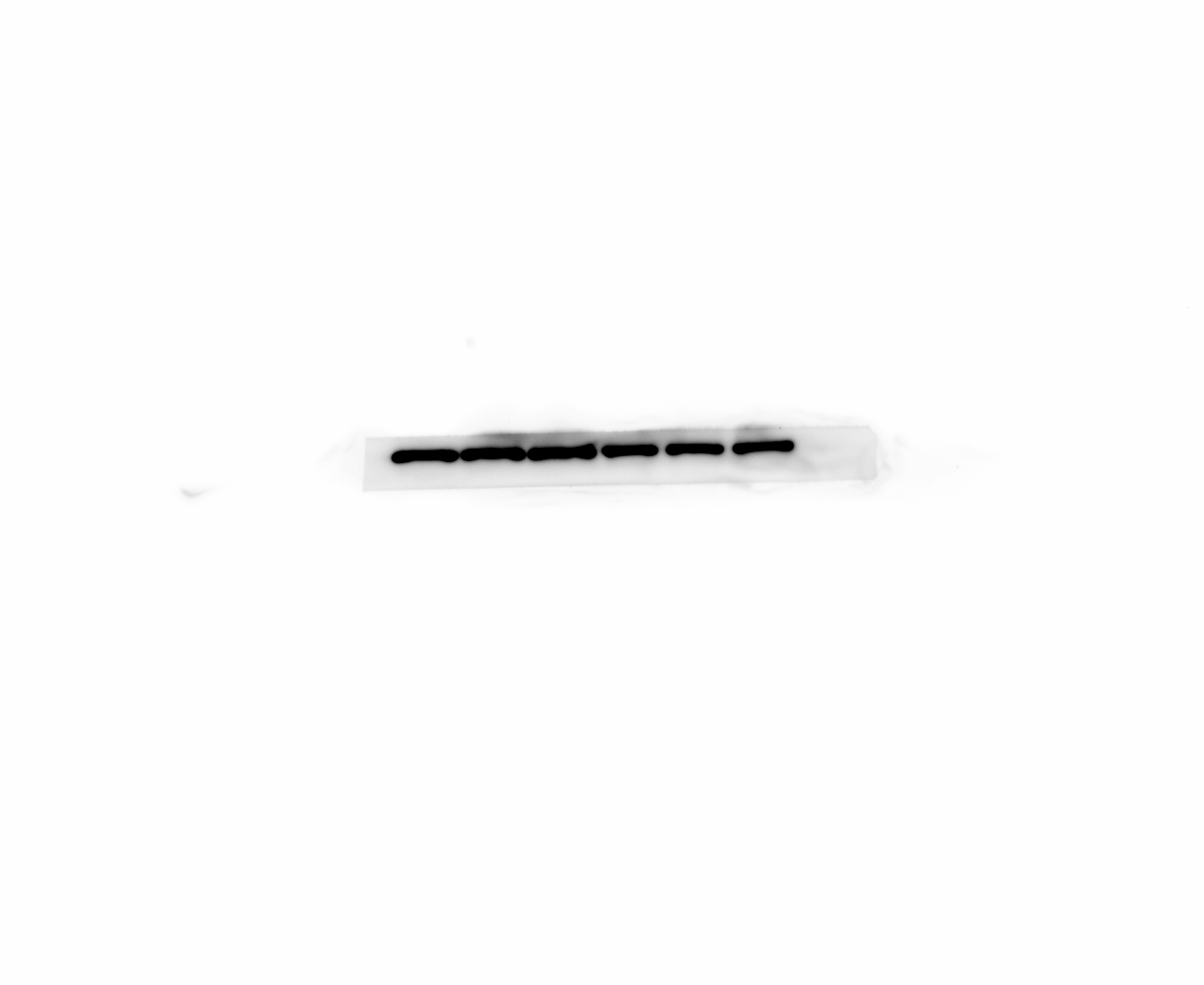

Supplement: Supplementary file 1 — Supplementary file1 (ZIP 36116 KB) [file 432_2024_5625_MOESM1_ESM.zip › Original Images for BlotsGels/1.Figure 1/MMP-3/1/1-1-A(Y).tif]

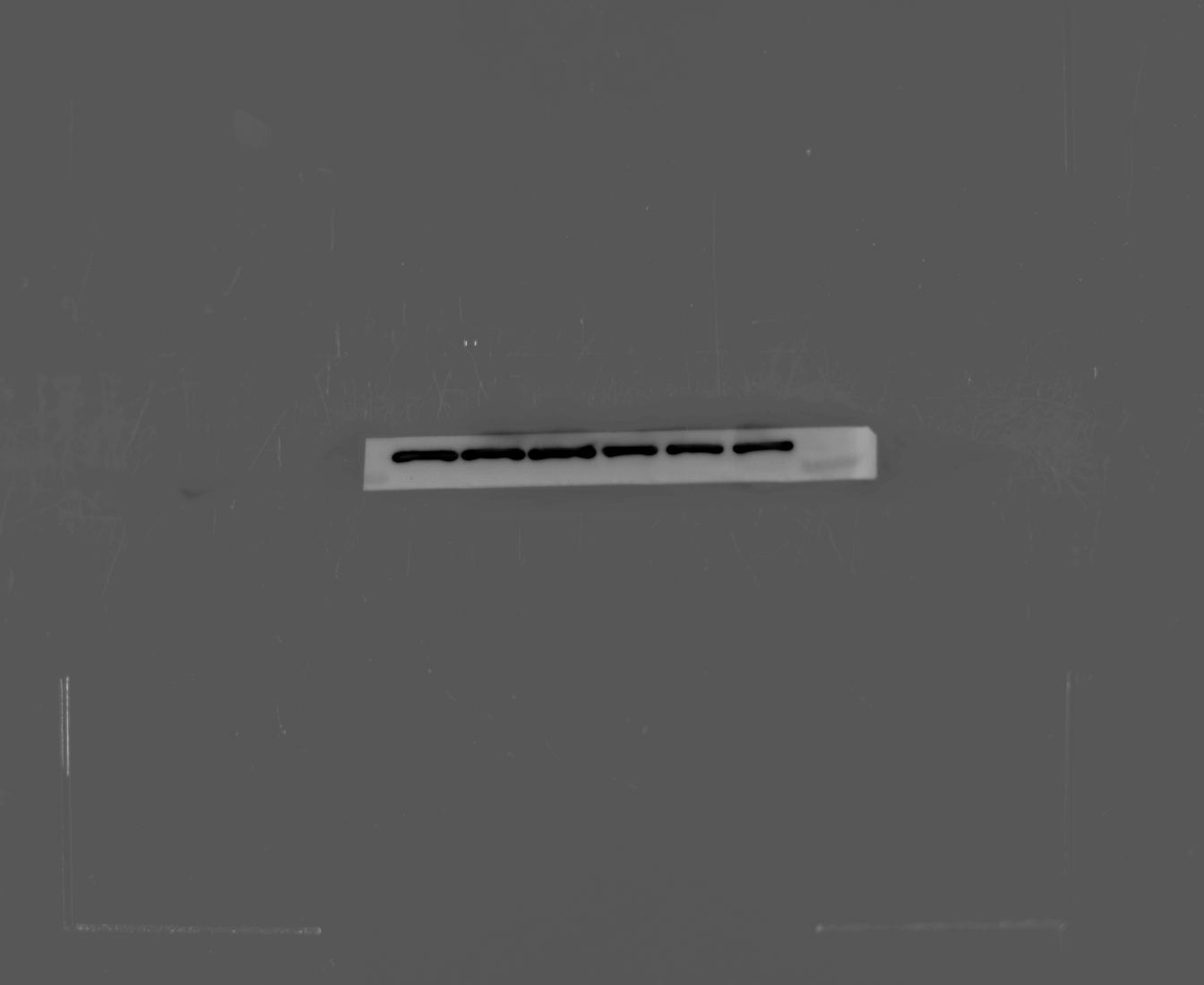

Supplement: Supplementary file 1 — Supplementary file1 (ZIP 36116 KB) [file 432_2024_5625_MOESM1_ESM.zip › Original Images for BlotsGels/1.Figure 1/MMP-3/1/1-1-A.tif]

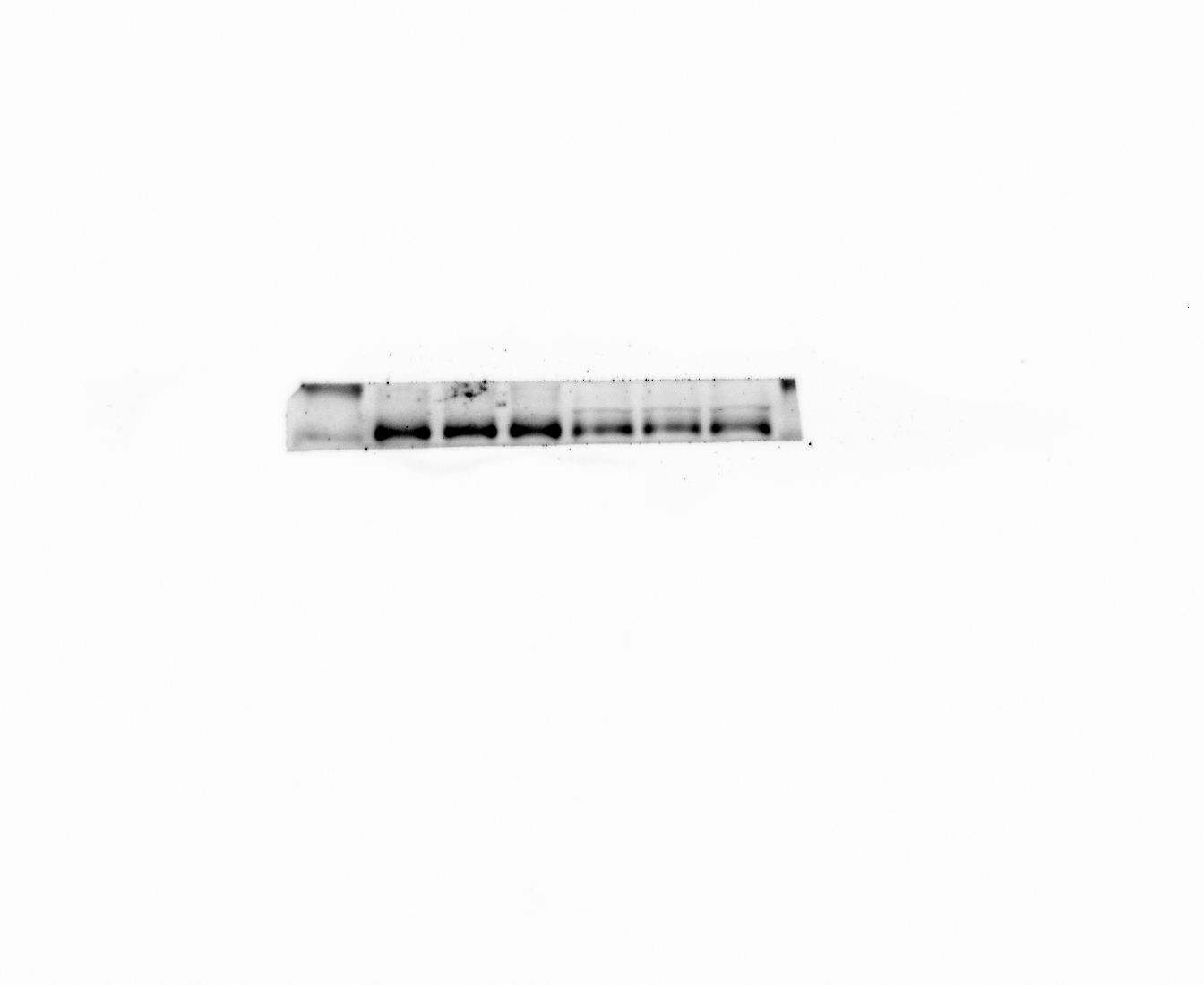

Supplement: Supplementary file 1 — Supplementary file1 (ZIP 36116 KB) [file 432_2024_5625_MOESM1_ESM.zip › Original Images for BlotsGels/1.Figure 1/MMP-3/1/1-1-MMP3(Y).tif]

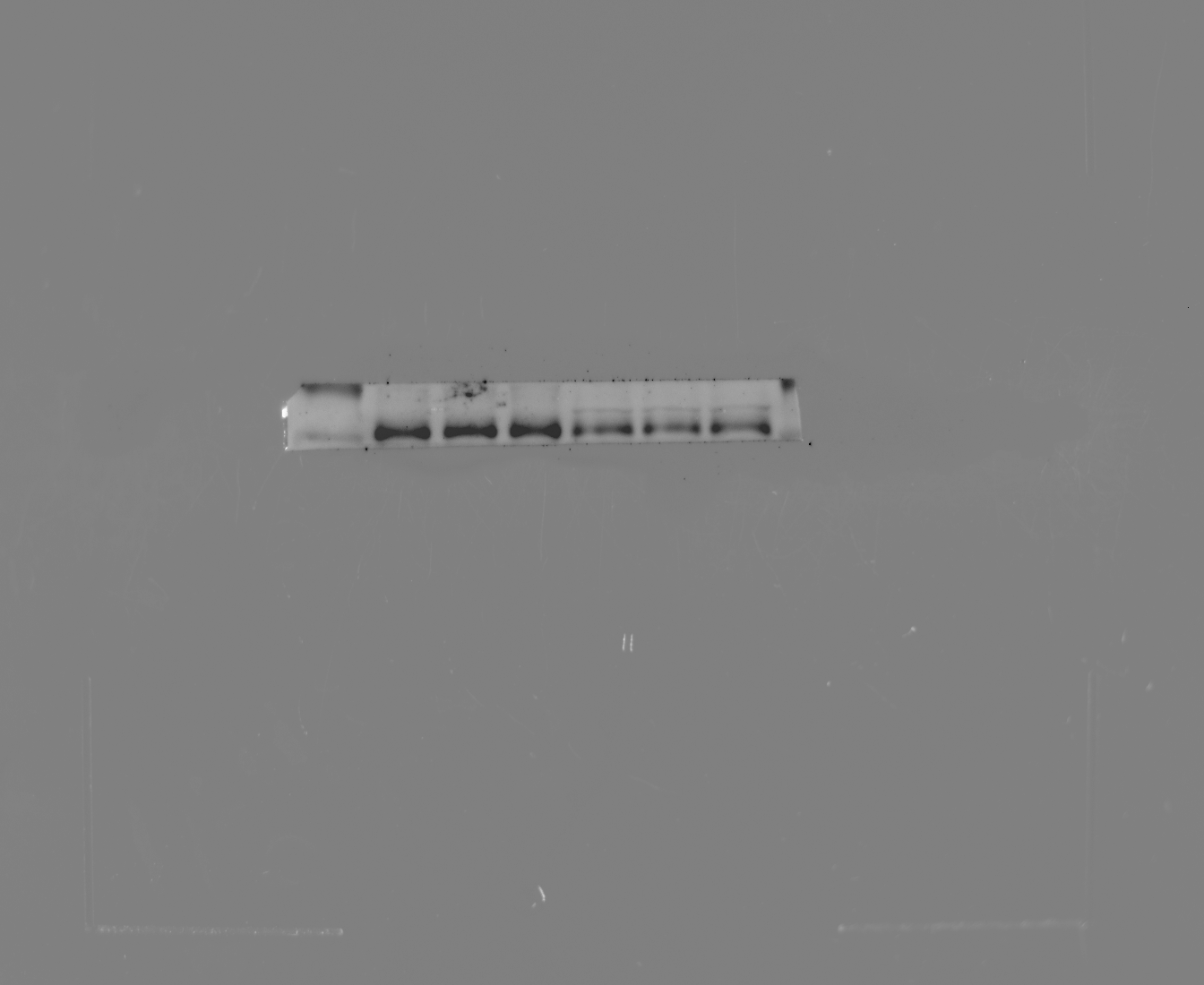

Supplement: Supplementary file 1 — Supplementary file1 (ZIP 36116 KB) [file 432_2024_5625_MOESM1_ESM.zip › Original Images for BlotsGels/1.Figure 1/MMP-3/1/1-1-MMP3.tif]

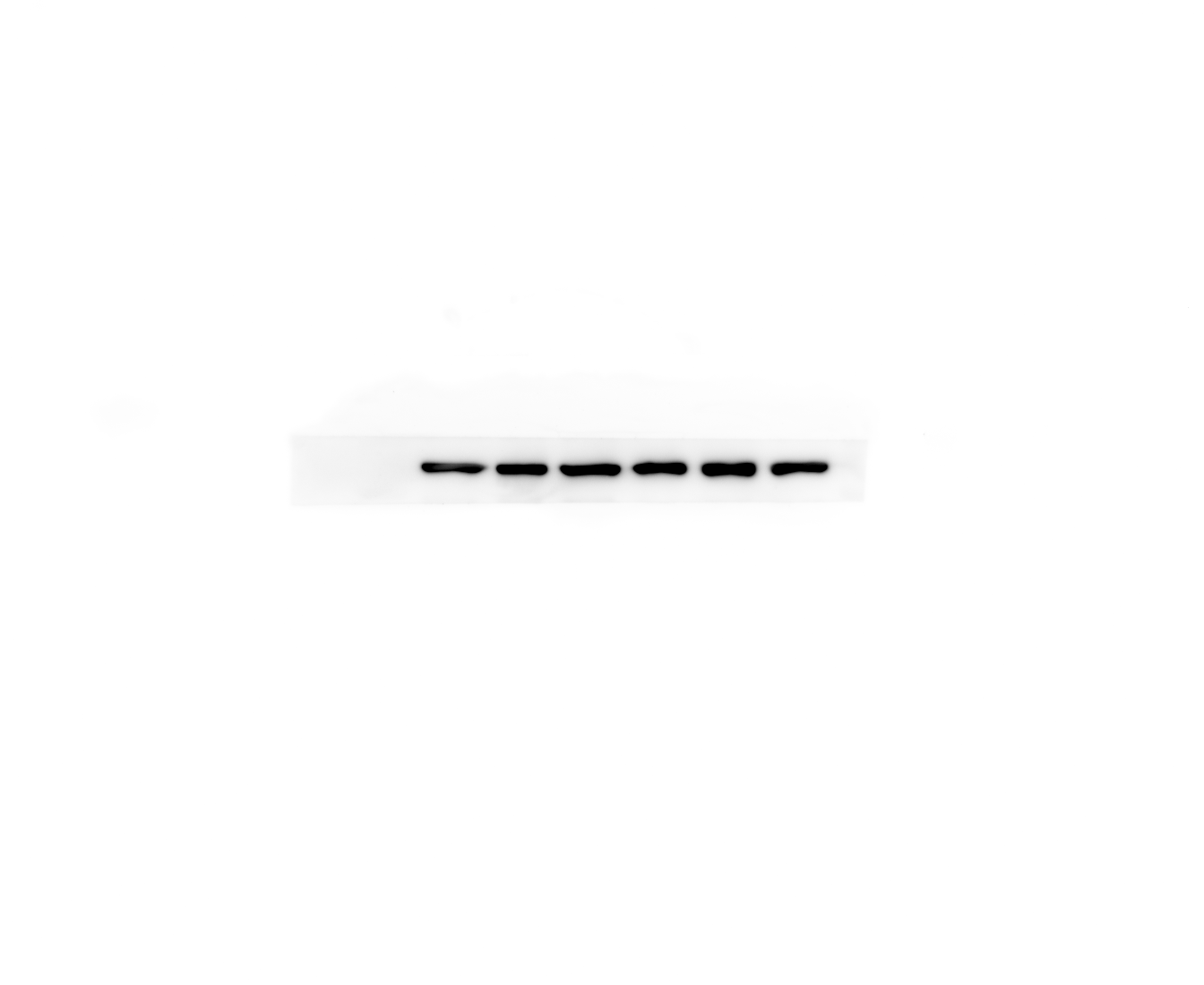

Supplement: Supplementary file 1 — Supplementary file1 (ZIP 36116 KB) [file 432_2024_5625_MOESM1_ESM.zip › Original Images for BlotsGels/1.Figure 1/N-cadherin/1/1-1-A(Y).tif]

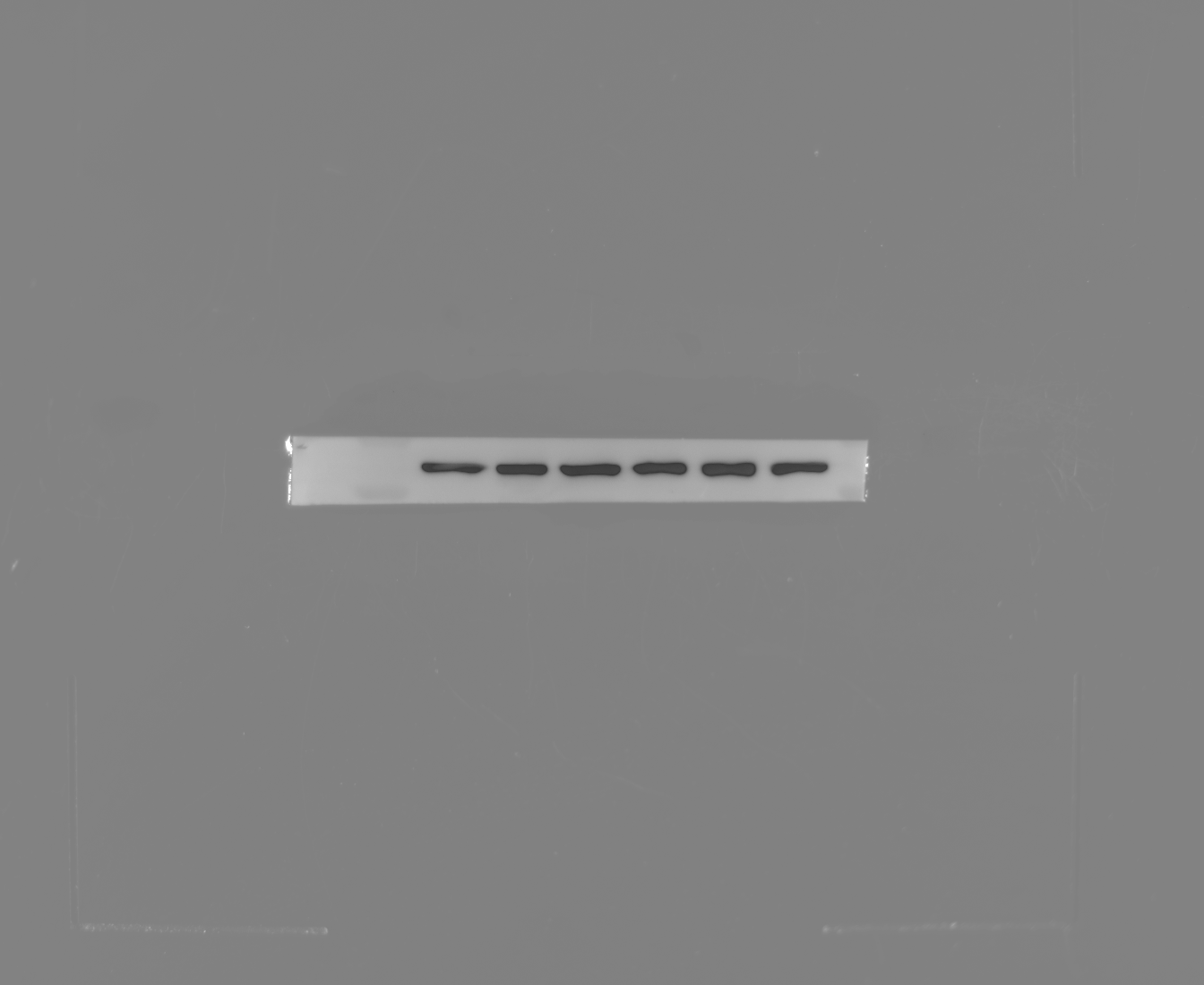

Supplement: Supplementary file 1 — Supplementary file1 (ZIP 36116 KB) [file 432_2024_5625_MOESM1_ESM.zip › Original Images for BlotsGels/1.Figure 1/N-cadherin/1/1-1-A.tif]

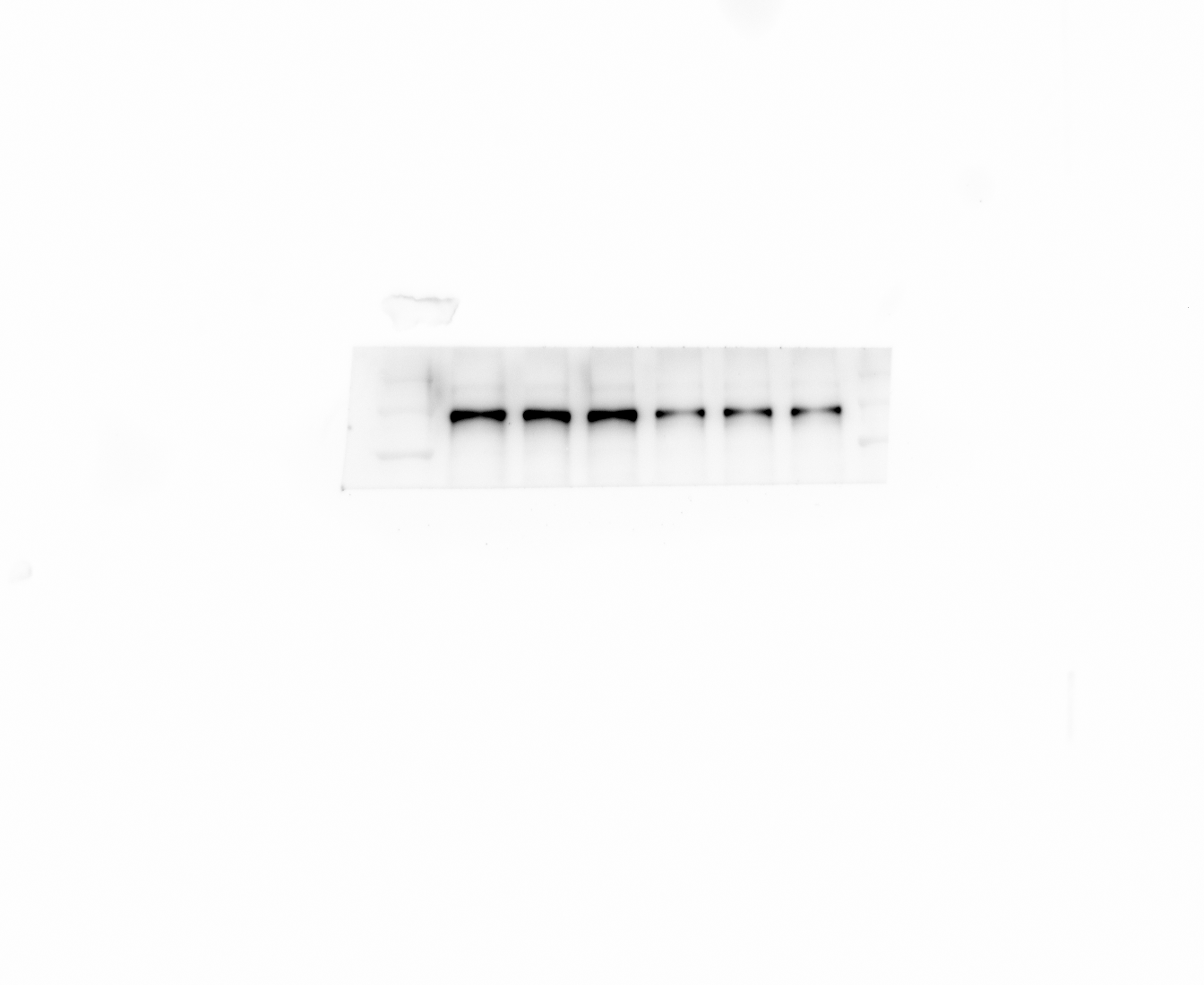

Supplement: Supplementary file 1 — Supplementary file1 (ZIP 36116 KB) [file 432_2024_5625_MOESM1_ESM.zip › Original Images for BlotsGels/1.Figure 1/N-cadherin/1/1-1-N-CA(Y).tif]

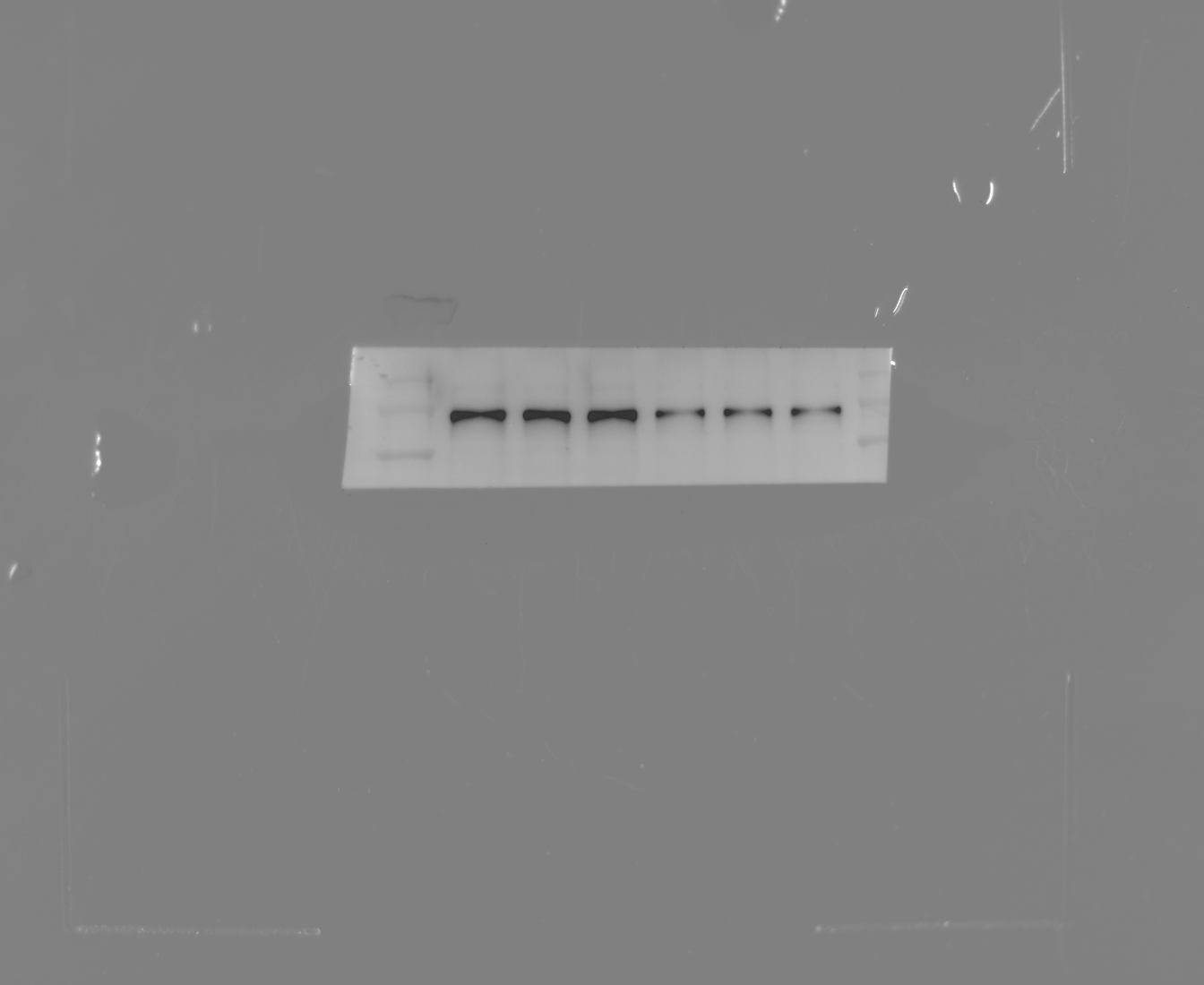

Supplement: Supplementary file 1 — Supplementary file1 (ZIP 36116 KB) [file 432_2024_5625_MOESM1_ESM.zip › Original Images for BlotsGels/1.Figure 1/N-cadherin/1/1-1-N-CA.tif]

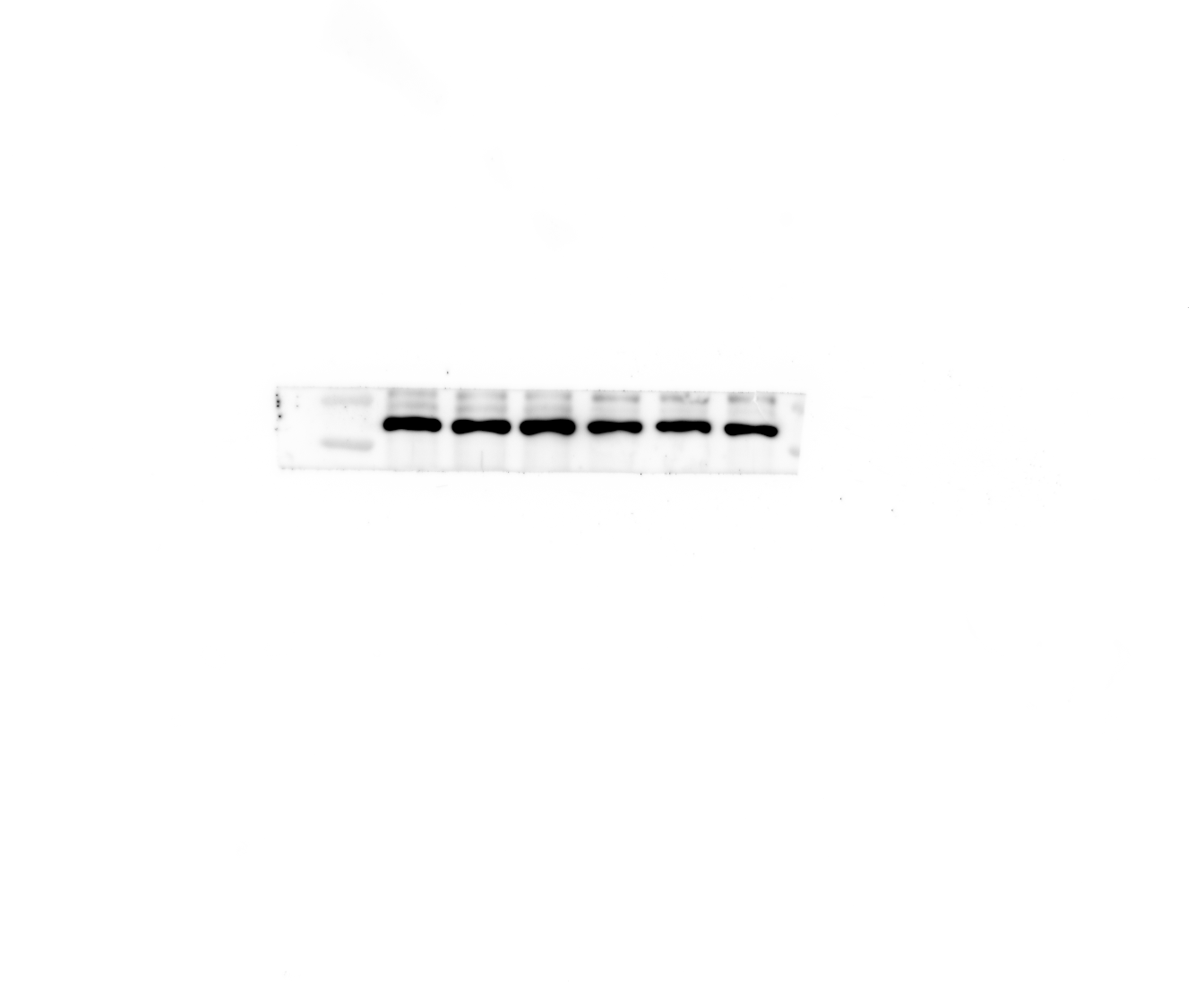

Supplement: Supplementary file 1 — Supplementary file1 (ZIP 36116 KB) [file 432_2024_5625_MOESM1_ESM.zip › Original Images for BlotsGels/1.Figure 1/Vimentin/1/2-1-A(Y).tif]

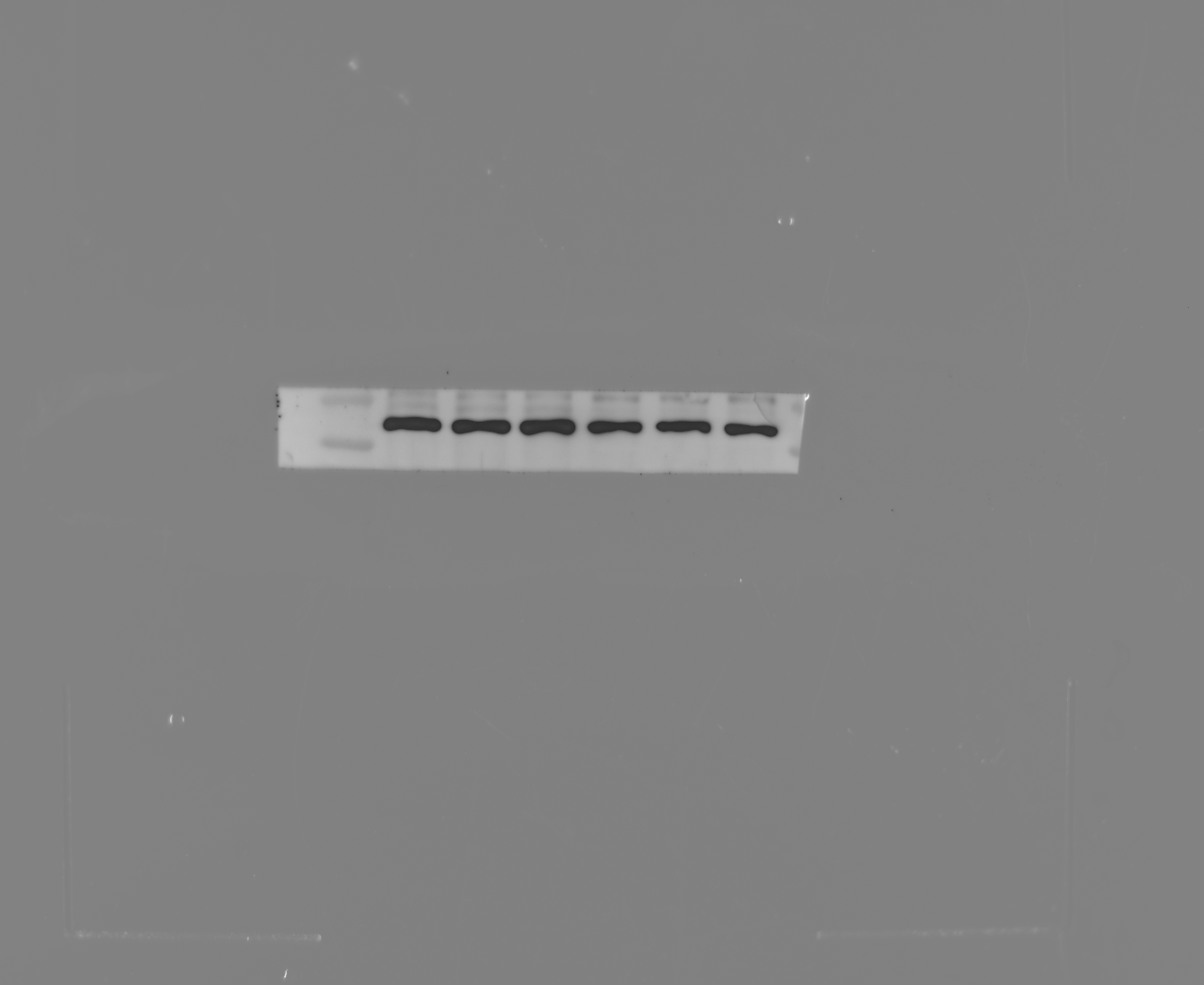

Supplement: Supplementary file 1 — Supplementary file1 (ZIP 36116 KB) [file 432_2024_5625_MOESM1_ESM.zip › Original Images for BlotsGels/1.Figure 1/Vimentin/1/2-1-A.tif]

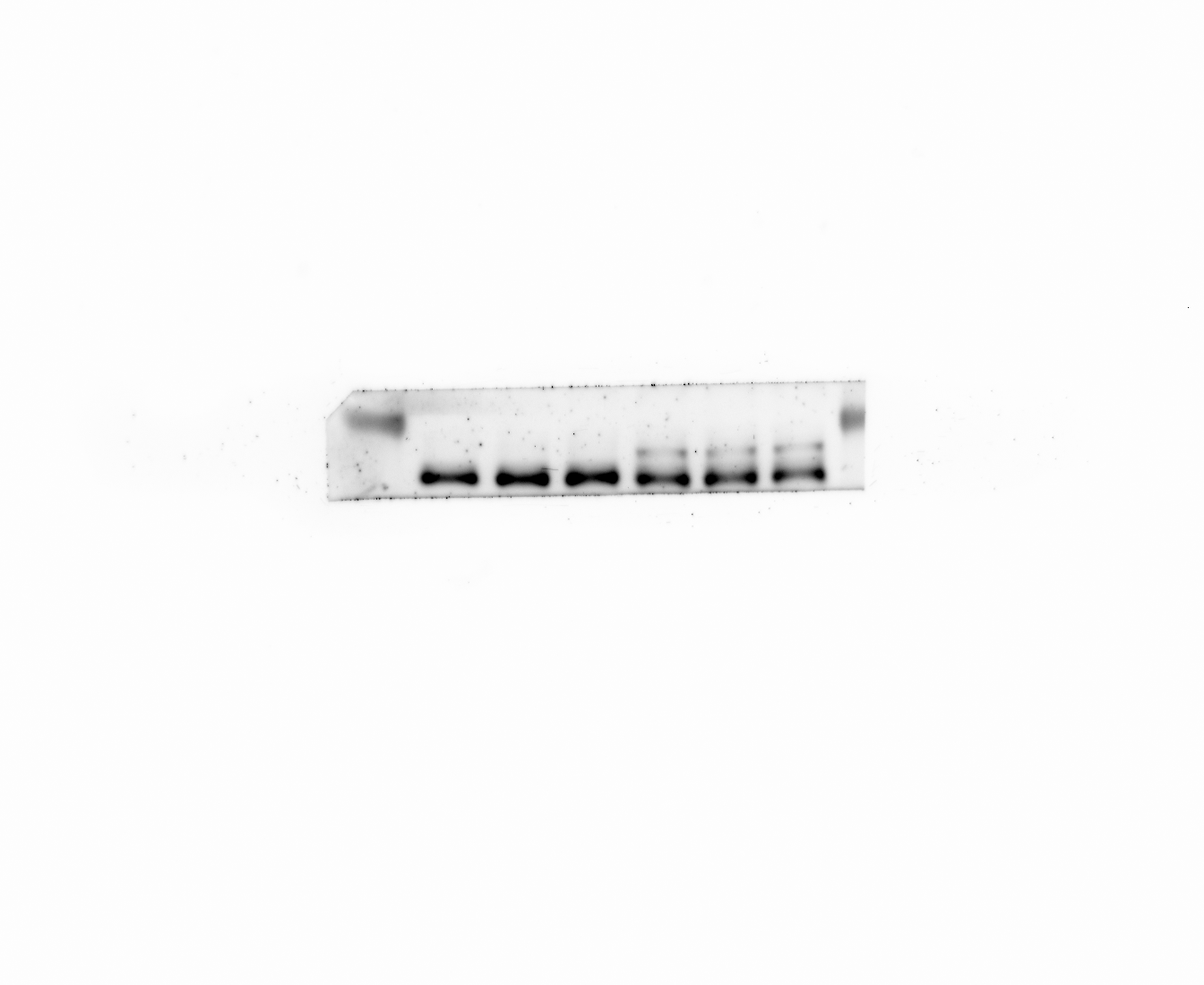

Supplement: Supplementary file 1 — Supplementary file1 (ZIP 36116 KB) [file 432_2024_5625_MOESM1_ESM.zip › Original Images for BlotsGels/1.Figure 1/Vimentin/1/2-1-Vimentin(Y).tif]

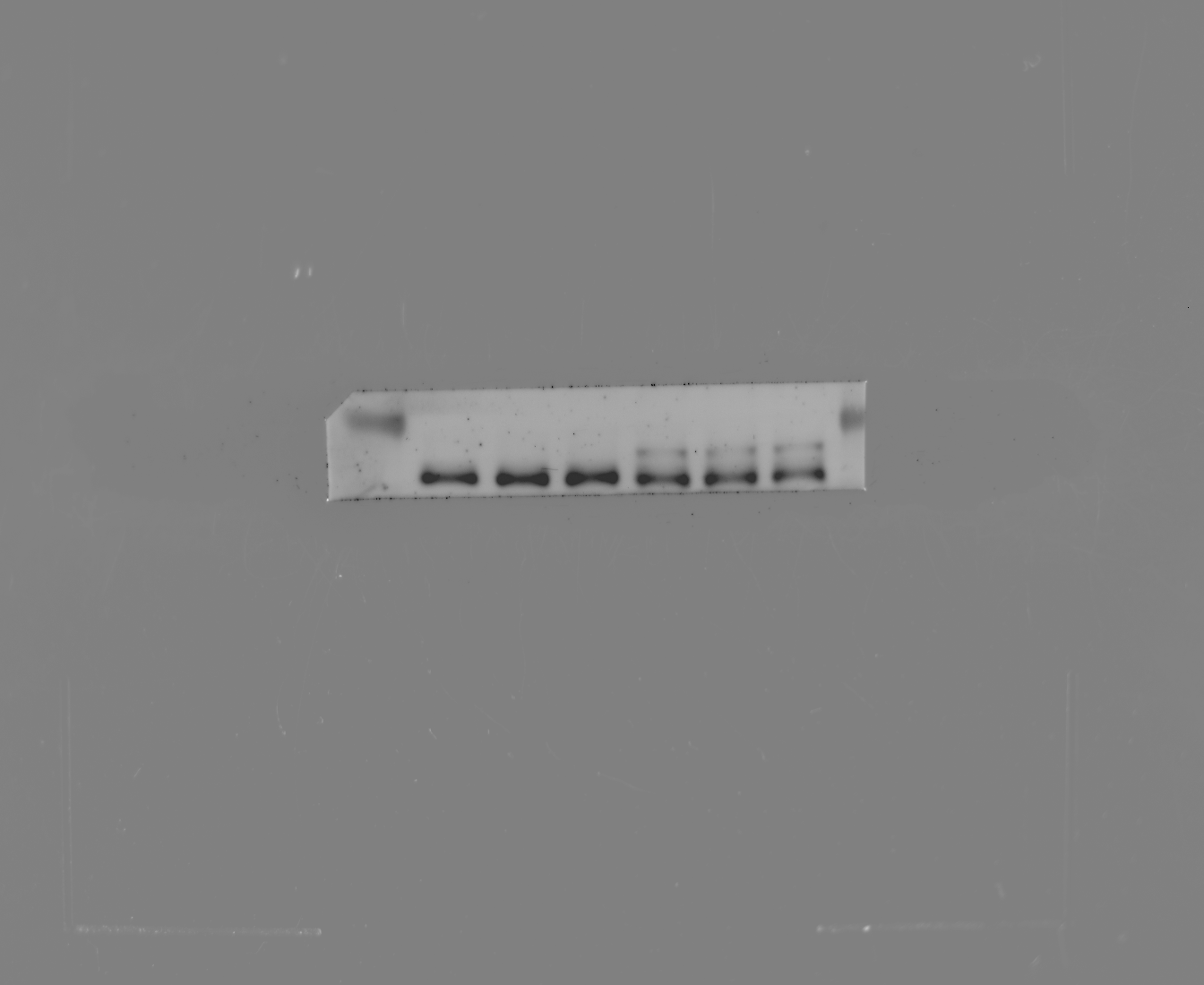

Supplement: Supplementary file 1 — Supplementary file1 (ZIP 36116 KB) [file 432_2024_5625_MOESM1_ESM.zip › Original Images for BlotsGels/1.Figure 1/Vimentin/1/2-1-Vimentin.tif]

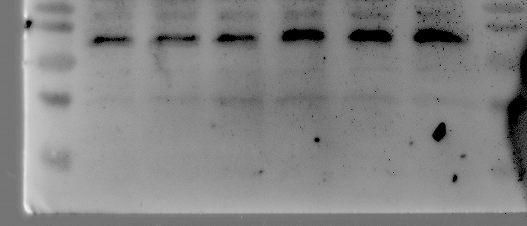

Supplement: Supplementary file 1 — Supplementary file1 (ZIP 36116 KB) [file 432_2024_5625_MOESM1_ESM.zip › Original Images for BlotsGels/2.Figure 1/WB/LN229/BAX/1/1.BAX(叠加图).tif]

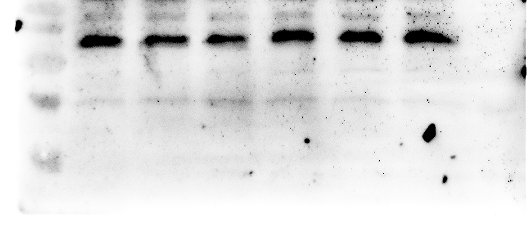

Supplement: Supplementary file 1 — Supplementary file1 (ZIP 36116 KB) [file 432_2024_5625_MOESM1_ESM.zip › Original Images for BlotsGels/2.Figure 1/WB/LN229/BAX/1/1.BAX(样品图).tif]

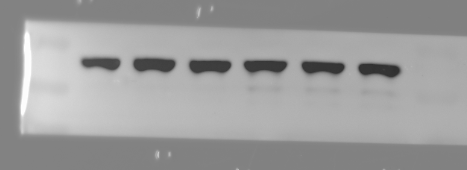

Supplement: Supplementary file 1 — Supplementary file1 (ZIP 36116 KB) [file 432_2024_5625_MOESM1_ESM.zip › Original Images for BlotsGels/2.Figure 1/WB/LN229/BAX/1/1.β-actin(叠加图).tif]

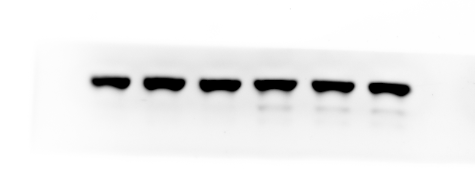

Supplement: Supplementary file 1 — Supplementary file1 (ZIP 36116 KB) [file 432_2024_5625_MOESM1_ESM.zip › Original Images for BlotsGels/2.Figure 1/WB/LN229/BAX/1/1.β-actin(样品图).tif]

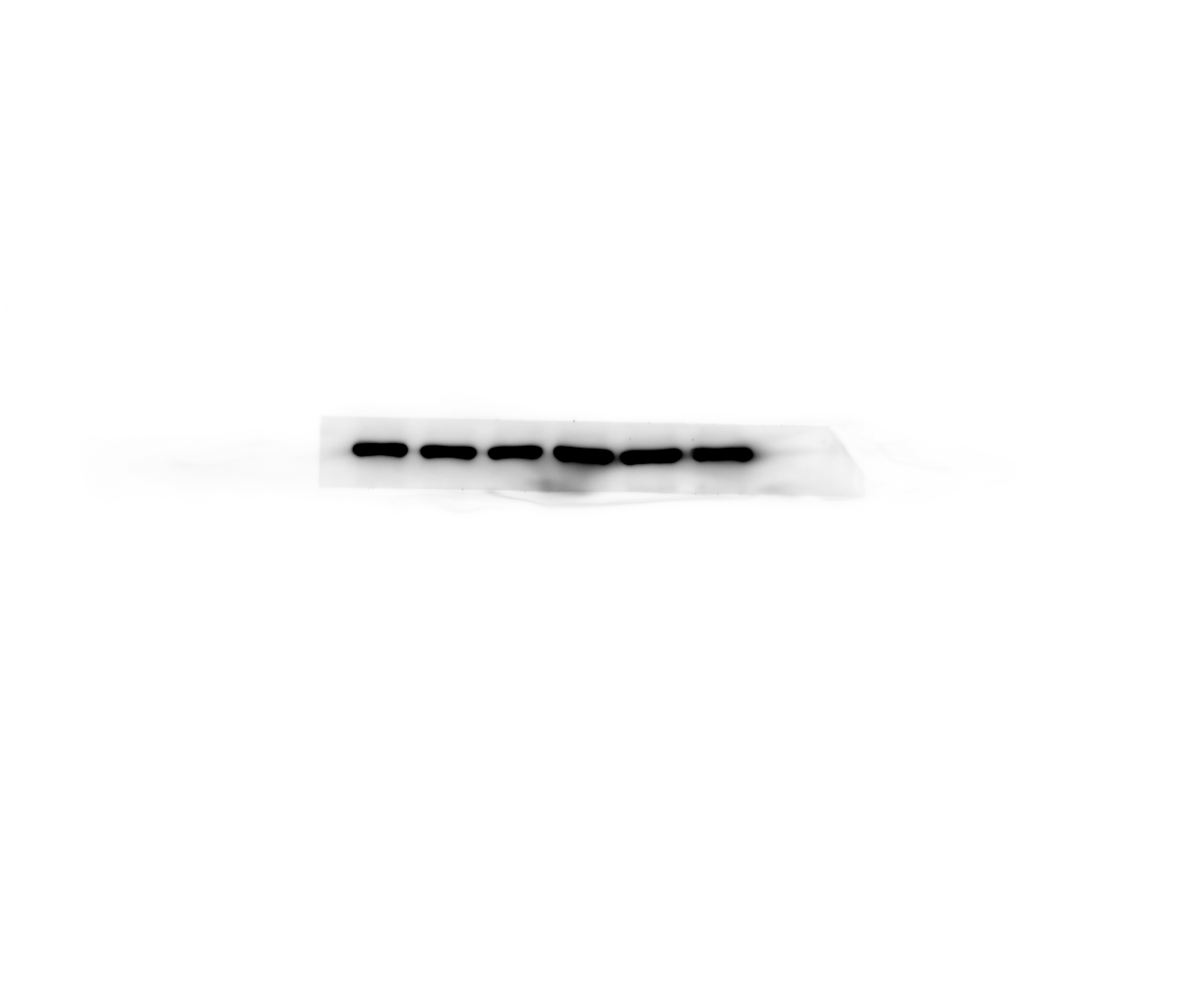

Supplement: Supplementary file 1 — Supplementary file1 (ZIP 36116 KB) [file 432_2024_5625_MOESM1_ESM.zip › Original Images for BlotsGels/2.Figure 1/WB/LN229/BCL2/1/1-2-A(Y).tif]

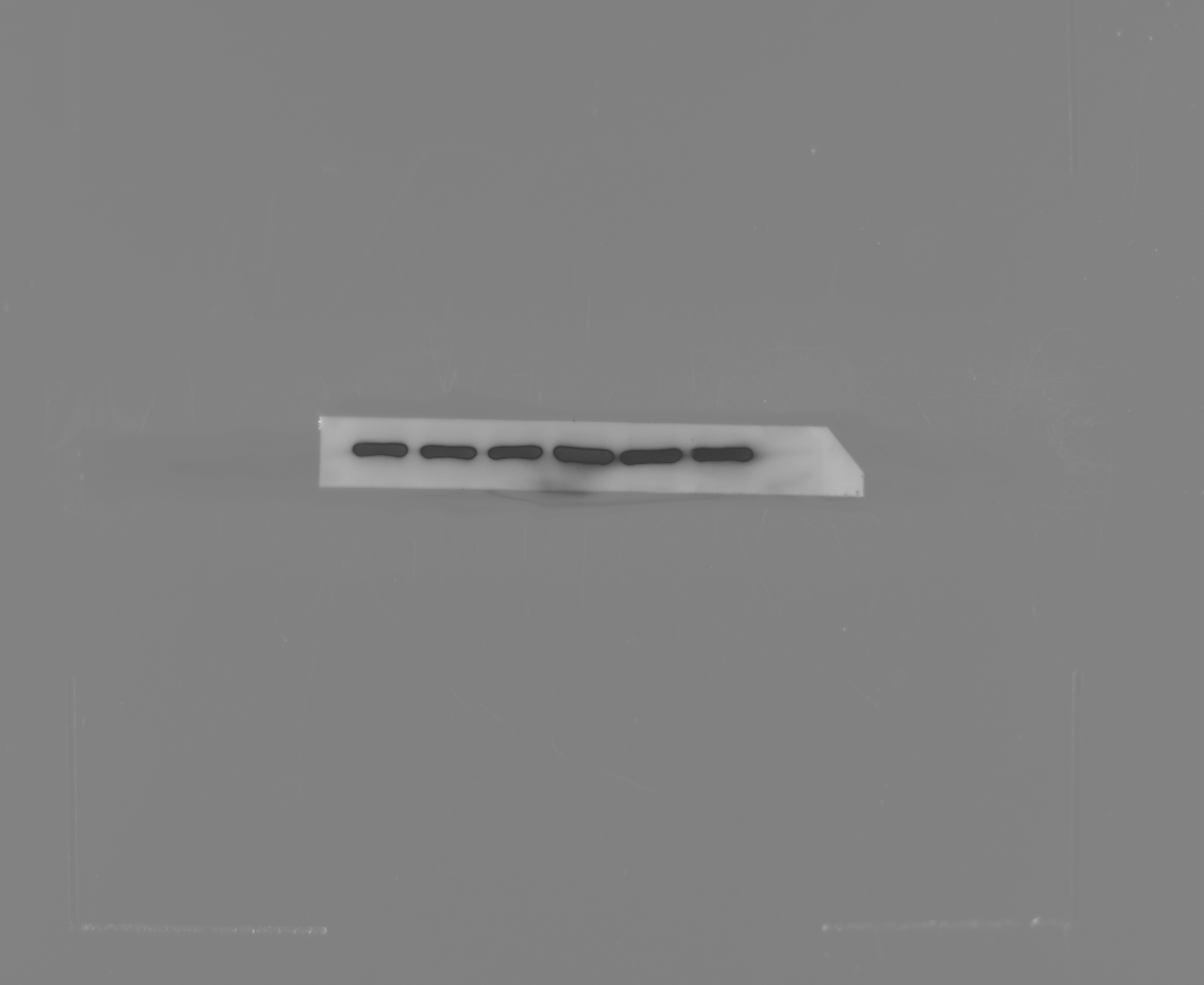

Supplement: Supplementary file 1 — Supplementary file1 (ZIP 36116 KB) [file 432_2024_5625_MOESM1_ESM.zip › Original Images for BlotsGels/2.Figure 1/WB/LN229/BCL2/1/1-2-A.tif]

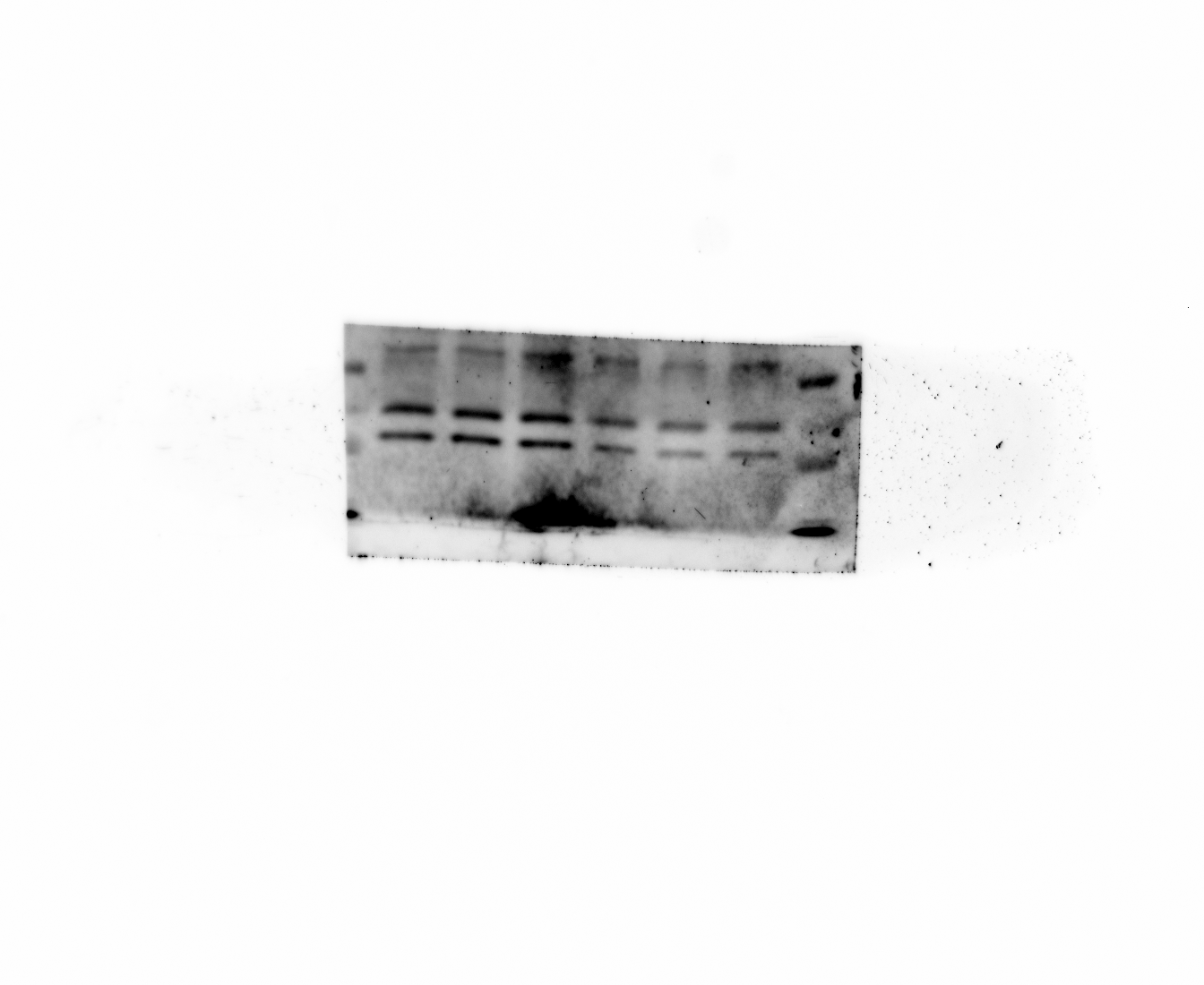

Supplement: Supplementary file 1 — Supplementary file1 (ZIP 36116 KB) [file 432_2024_5625_MOESM1_ESM.zip › Original Images for BlotsGels/2.Figure 1/WB/LN229/BCL2/1/1-2-BCL2(Y).tif]

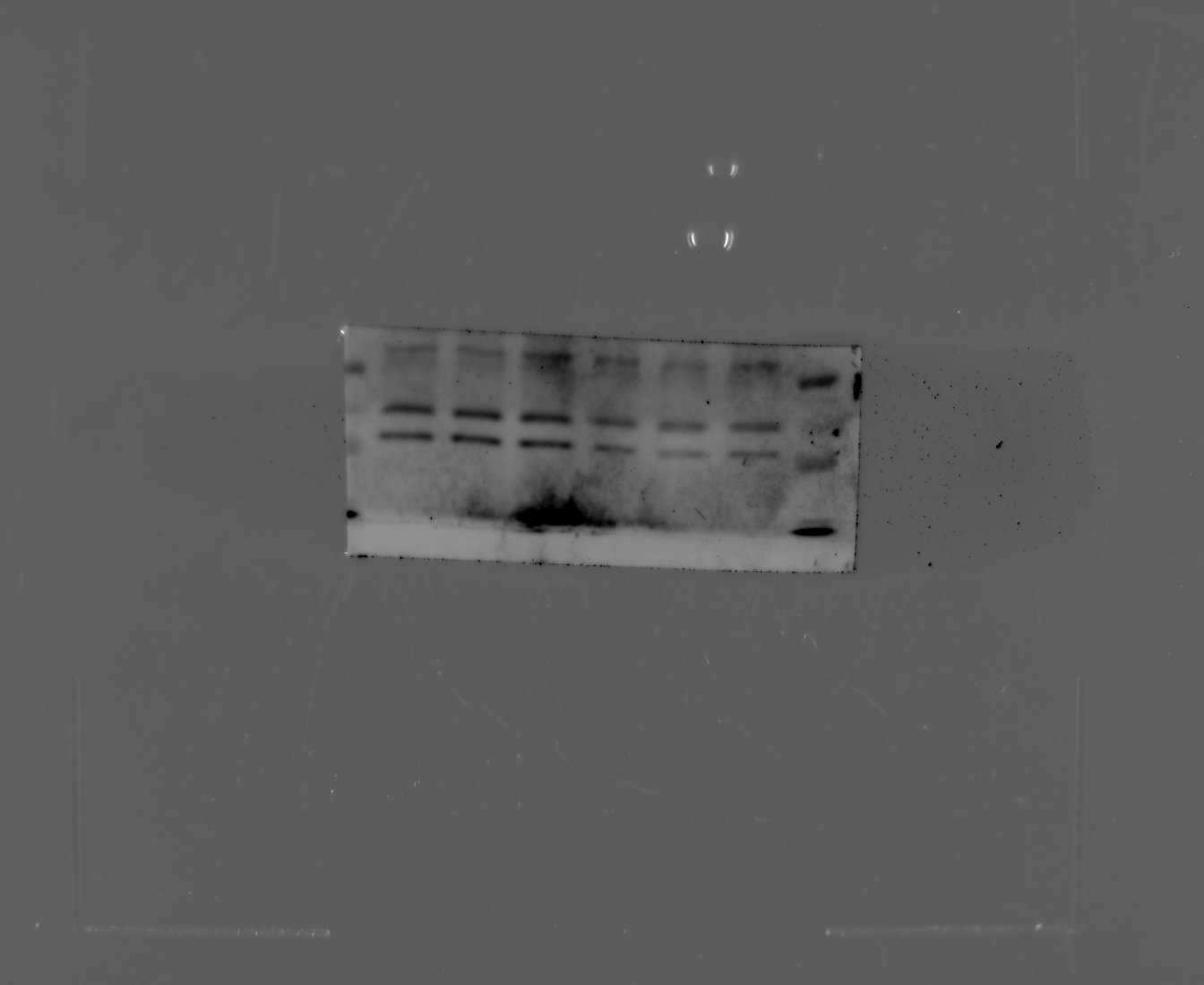

Supplement: Supplementary file 1 — Supplementary file1 (ZIP 36116 KB) [file 432_2024_5625_MOESM1_ESM.zip › Original Images for BlotsGels/2.Figure 1/WB/LN229/BCL2/1/1-2-BCL2.tif]

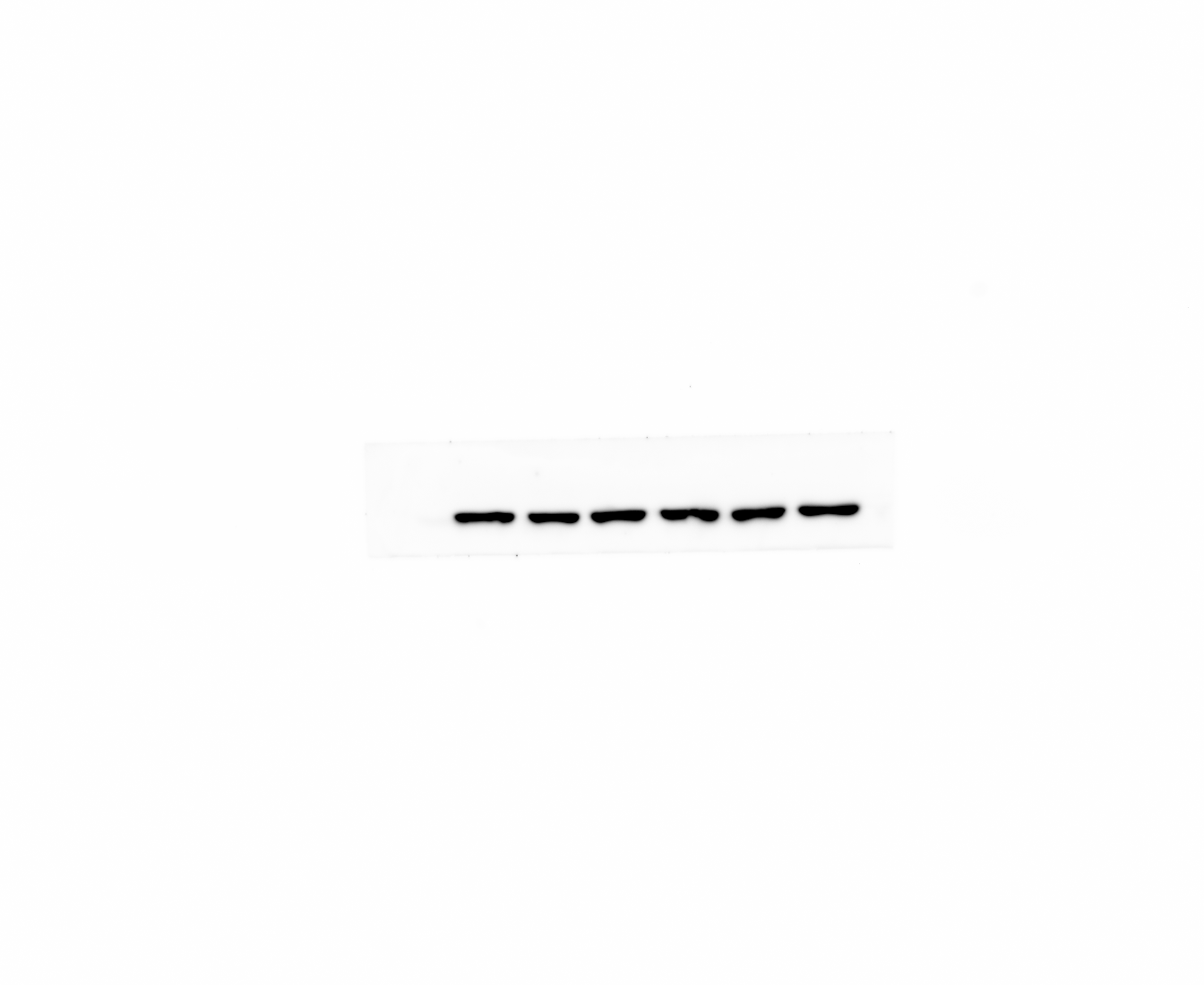

Supplement: Supplementary file 1 — Supplementary file1 (ZIP 36116 KB) [file 432_2024_5625_MOESM1_ESM.zip › Original Images for BlotsGels/3.Figure 3/LN229/1.JAK2/1/1-1-A(Y).tif]

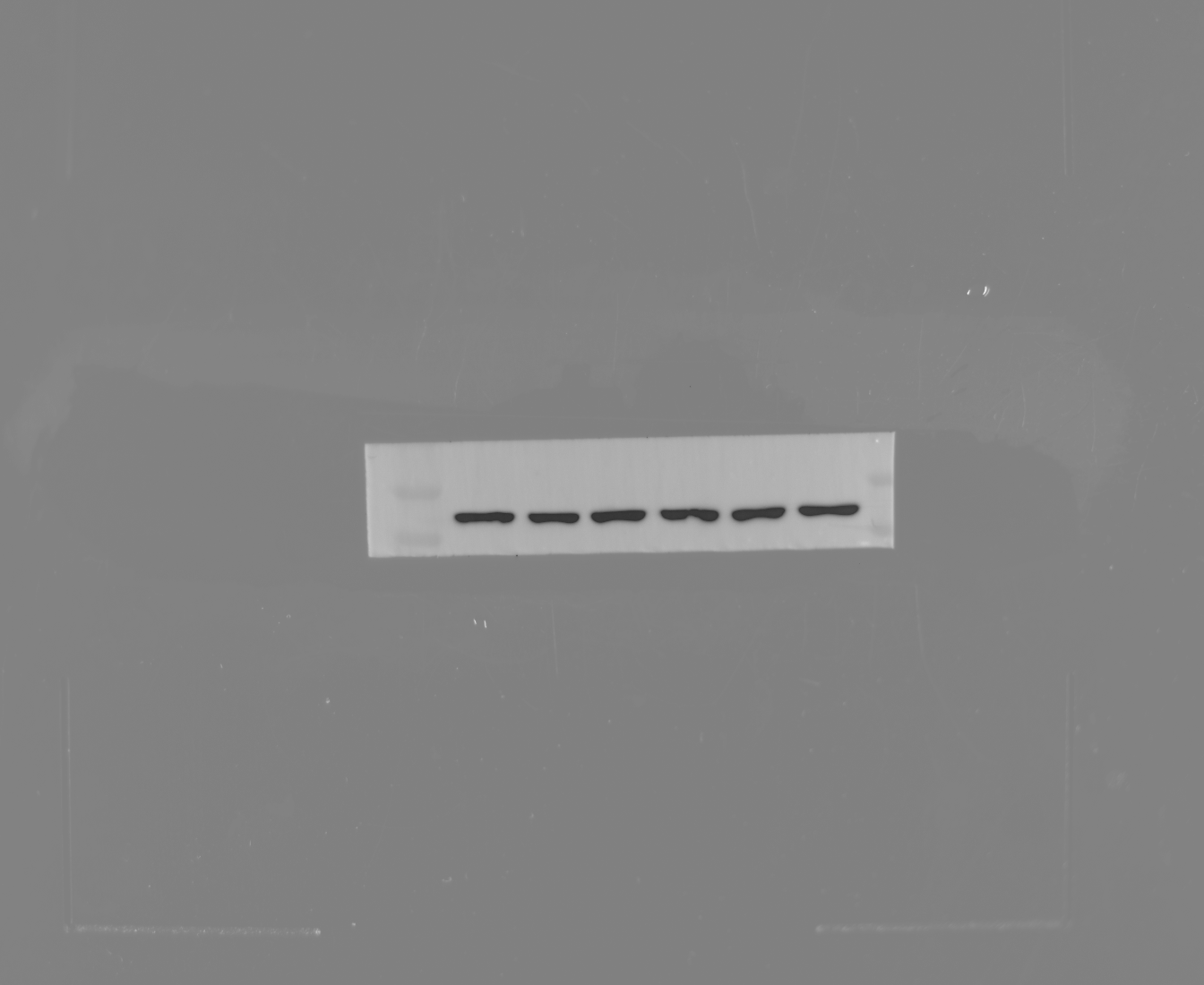

Supplement: Supplementary file 1 — Supplementary file1 (ZIP 36116 KB) [file 432_2024_5625_MOESM1_ESM.zip › Original Images for BlotsGels/3.Figure 3/LN229/1.JAK2/1/1-1-A.tif]

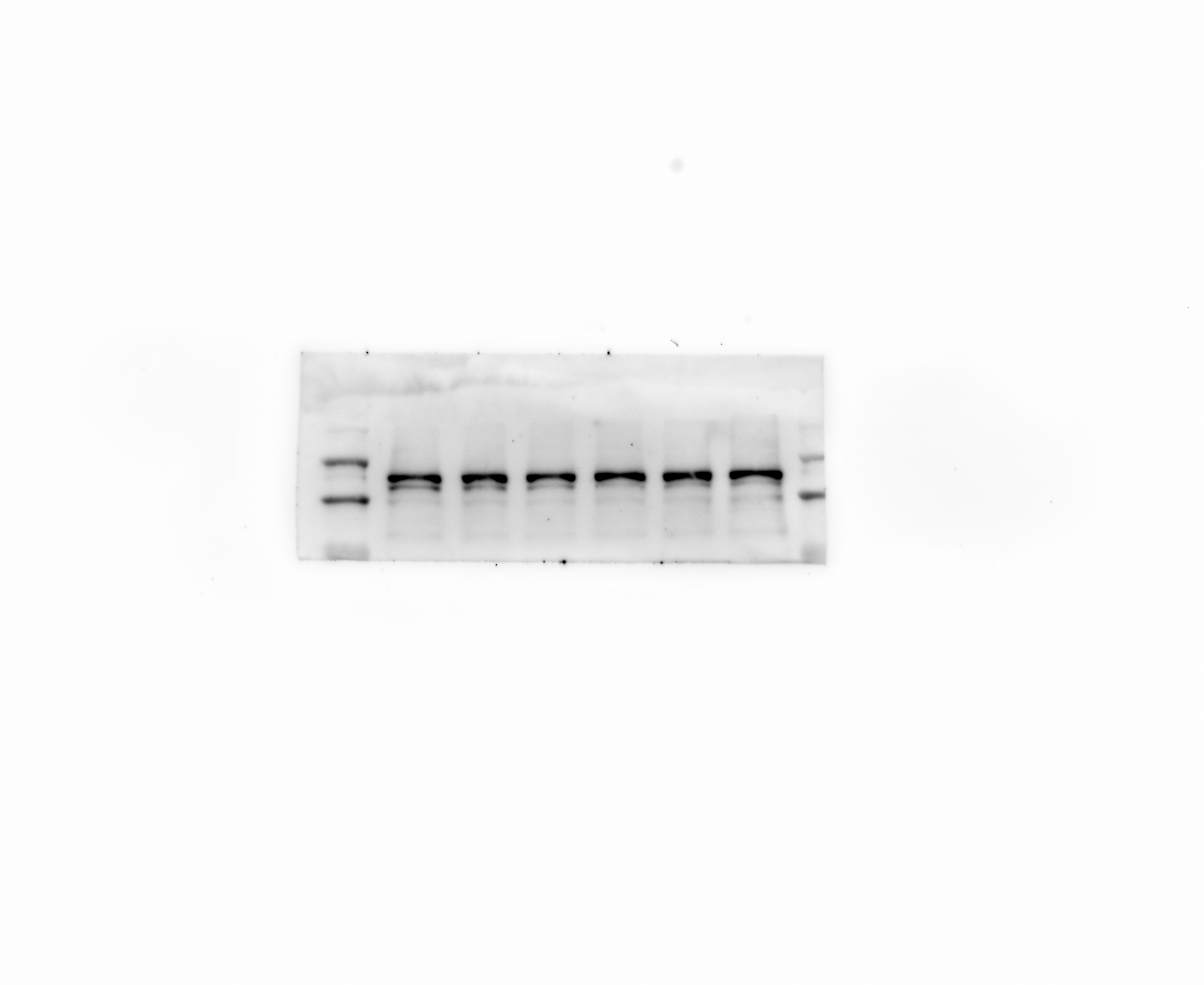

Supplement: Supplementary file 1 — Supplementary file1 (ZIP 36116 KB) [file 432_2024_5625_MOESM1_ESM.zip › Original Images for BlotsGels/3.Figure 3/LN229/1.JAK2/1/1-1-JAK2(Y).tif]

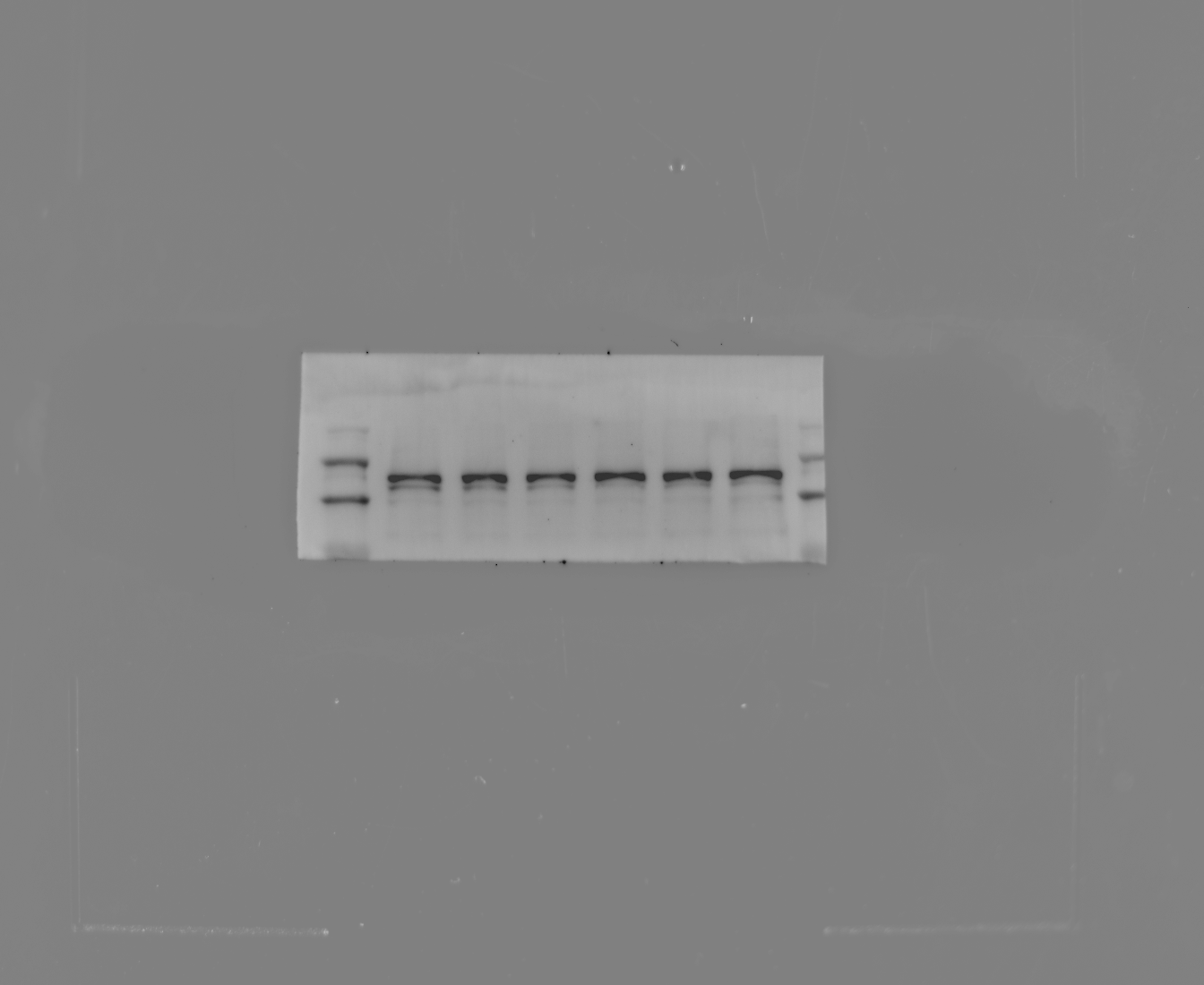

Supplement: Supplementary file 1 — Supplementary file1 (ZIP 36116 KB) [file 432_2024_5625_MOESM1_ESM.zip › Original Images for BlotsGels/3.Figure 3/LN229/1.JAK2/1/1-1-JAK2.tif]

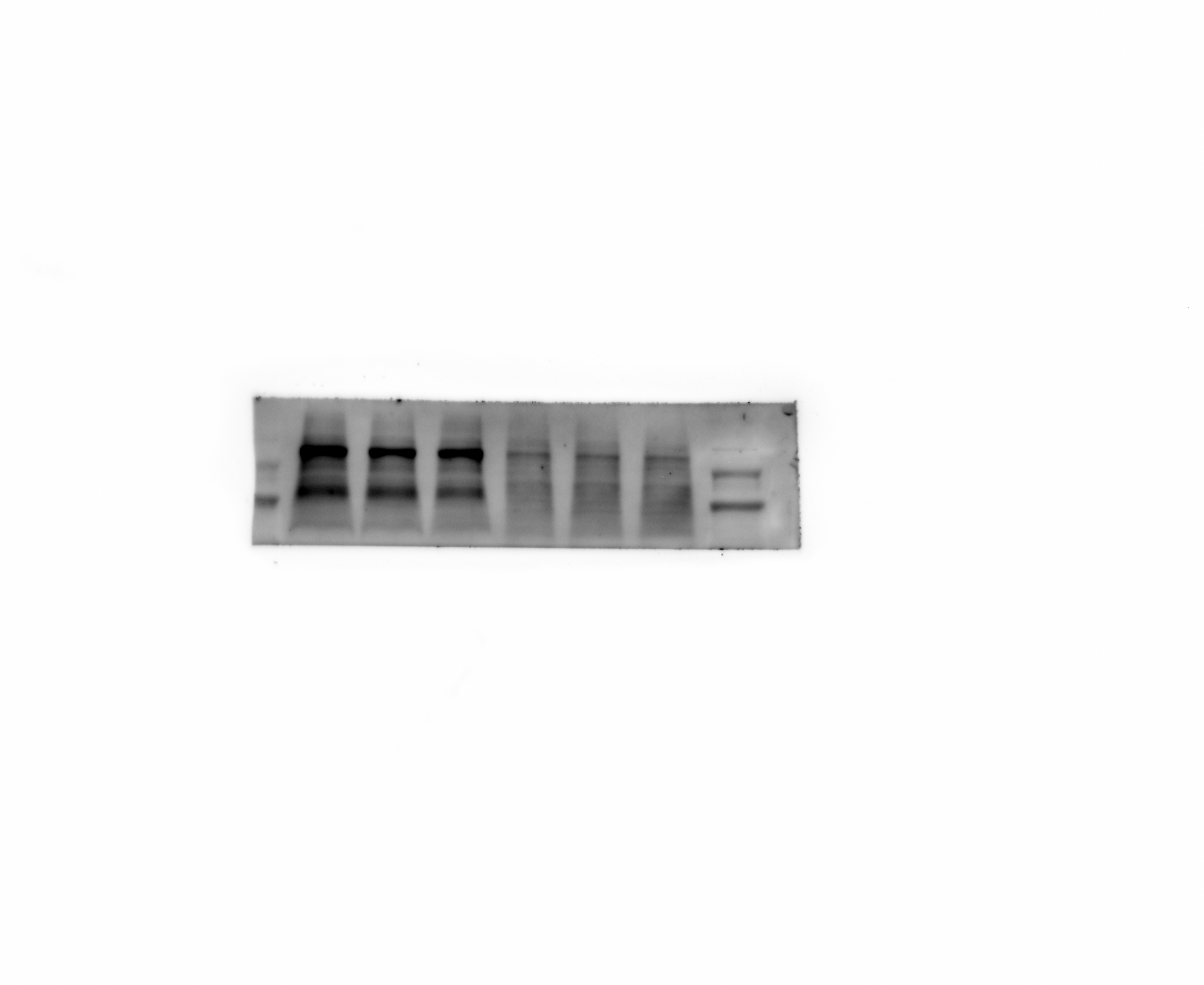

Supplement: Supplementary file 1 — Supplementary file1 (ZIP 36116 KB) [file 432_2024_5625_MOESM1_ESM.zip › Original Images for BlotsGels/3.Figure 3/LN229/2.P-JAK2/1/2-2-P-JAK2(Y).tif]

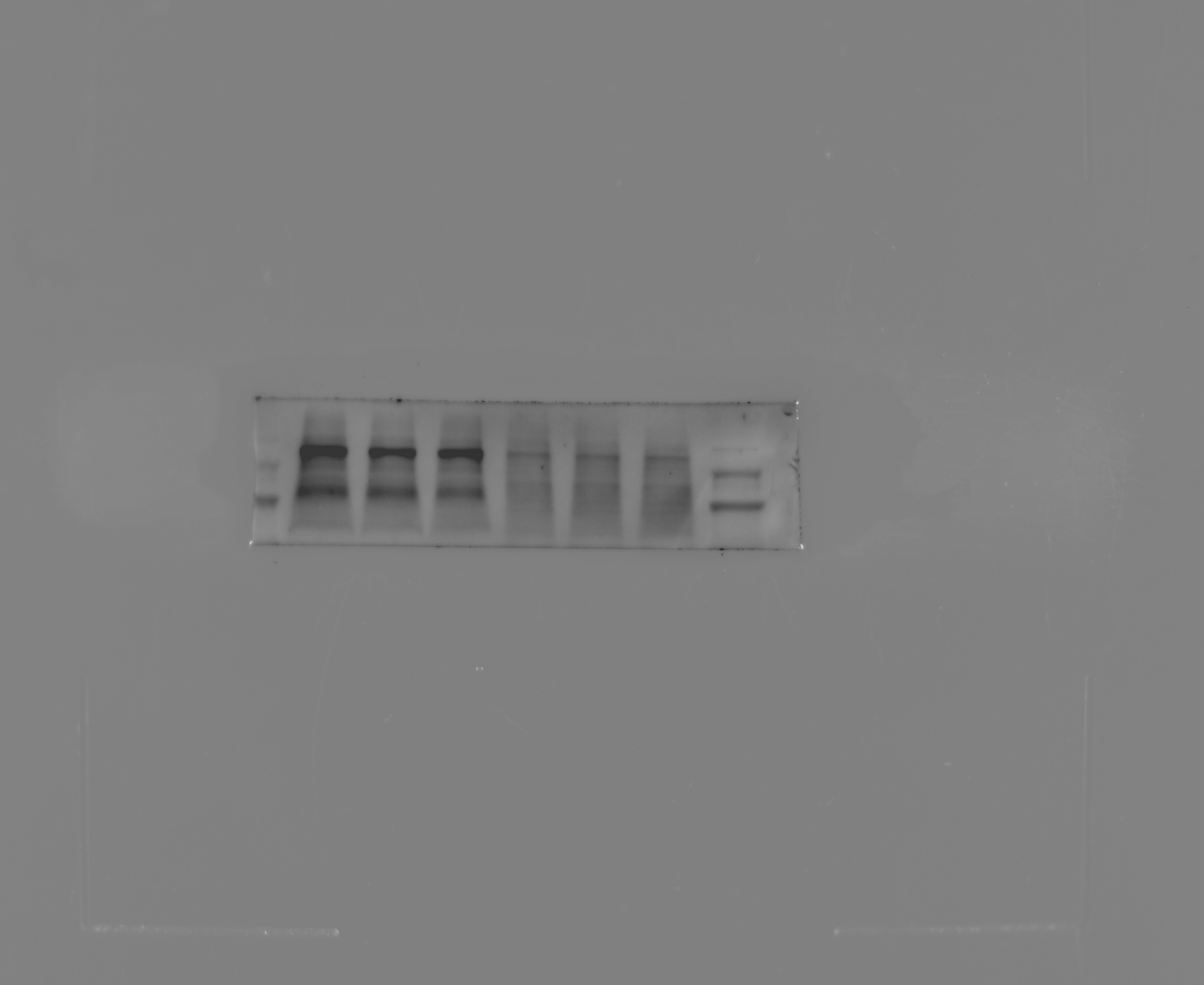

Supplement: Supplementary file 1 — Supplementary file1 (ZIP 36116 KB) [file 432_2024_5625_MOESM1_ESM.zip › Original Images for BlotsGels/3.Figure 3/LN229/2.P-JAK2/1/2-2-P-JAK2.tif]

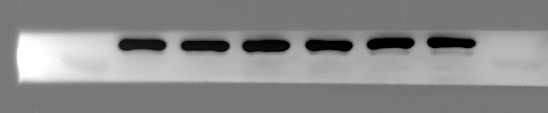

Supplement: Supplementary file 1 — Supplementary file1 (ZIP 36116 KB) [file 432_2024_5625_MOESM1_ESM.zip › Original Images for BlotsGels/3.Figure 3/LN229/2.P-JAK2/1/2-2-β-anctin(叠加图).tif]

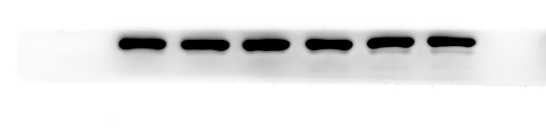

Supplement: Supplementary file 1 — Supplementary file1 (ZIP 36116 KB) [file 432_2024_5625_MOESM1_ESM.zip › Original Images for BlotsGels/3.Figure 3/LN229/2.P-JAK2/1/2-2-β-anctin(样品图).tif]

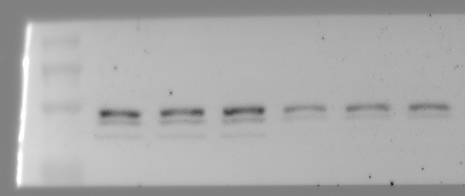

Supplement: Supplementary file 1 — Supplementary file1 (ZIP 36116 KB) [file 432_2024_5625_MOESM1_ESM.zip › Original Images for BlotsGels/3.Figure 3/LN229/3.P-STAT3/1/1.P-STAT3(叠加图).tif]

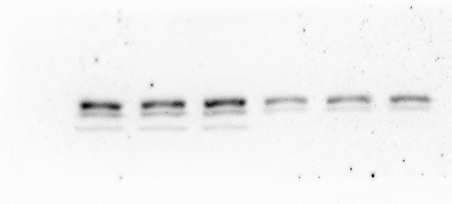

Supplement: Supplementary file 1 — Supplementary file1 (ZIP 36116 KB) [file 432_2024_5625_MOESM1_ESM.zip › Original Images for BlotsGels/3.Figure 3/LN229/3.P-STAT3/1/1.P-STAT3(样品图).tif]

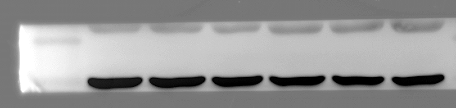

Supplement: Supplementary file 1 — Supplementary file1 (ZIP 36116 KB) [file 432_2024_5625_MOESM1_ESM.zip › Original Images for BlotsGels/3.Figure 3/LN229/3.P-STAT3/1/1.β-anctin(叠加图).tif]

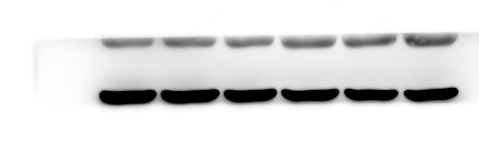

Supplement: Supplementary file 1 — Supplementary file1 (ZIP 36116 KB) [file 432_2024_5625_MOESM1_ESM.zip › Original Images for BlotsGels/3.Figure 3/LN229/3.P-STAT3/1/1.β-anctin(样品图).tif]

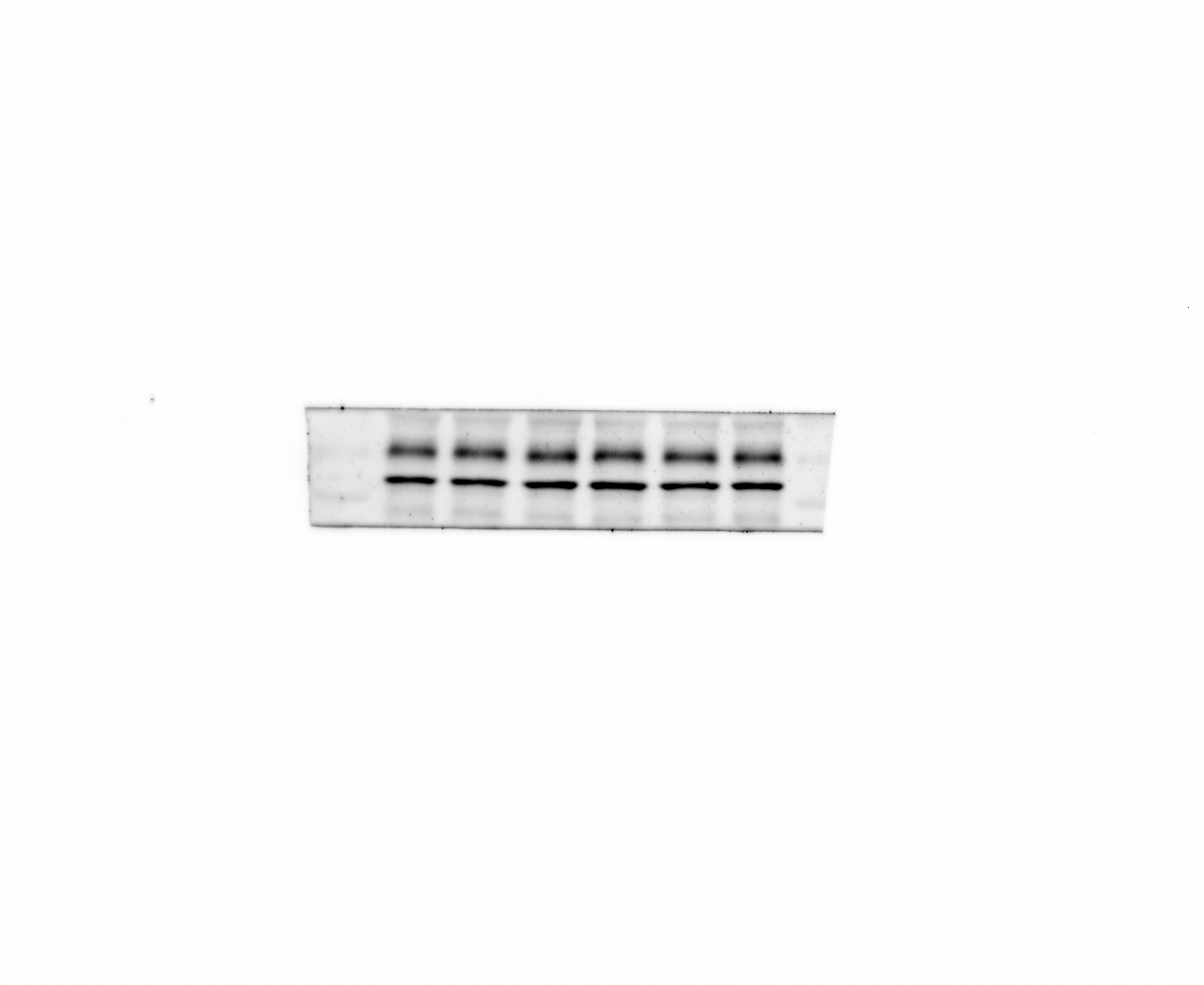

Supplement: Supplementary file 1 — Supplementary file1 (ZIP 36116 KB) [file 432_2024_5625_MOESM1_ESM.zip › Original Images for BlotsGels/3.Figure 3/LN229/4.STAT3/1/1-1-A(Y).tif]

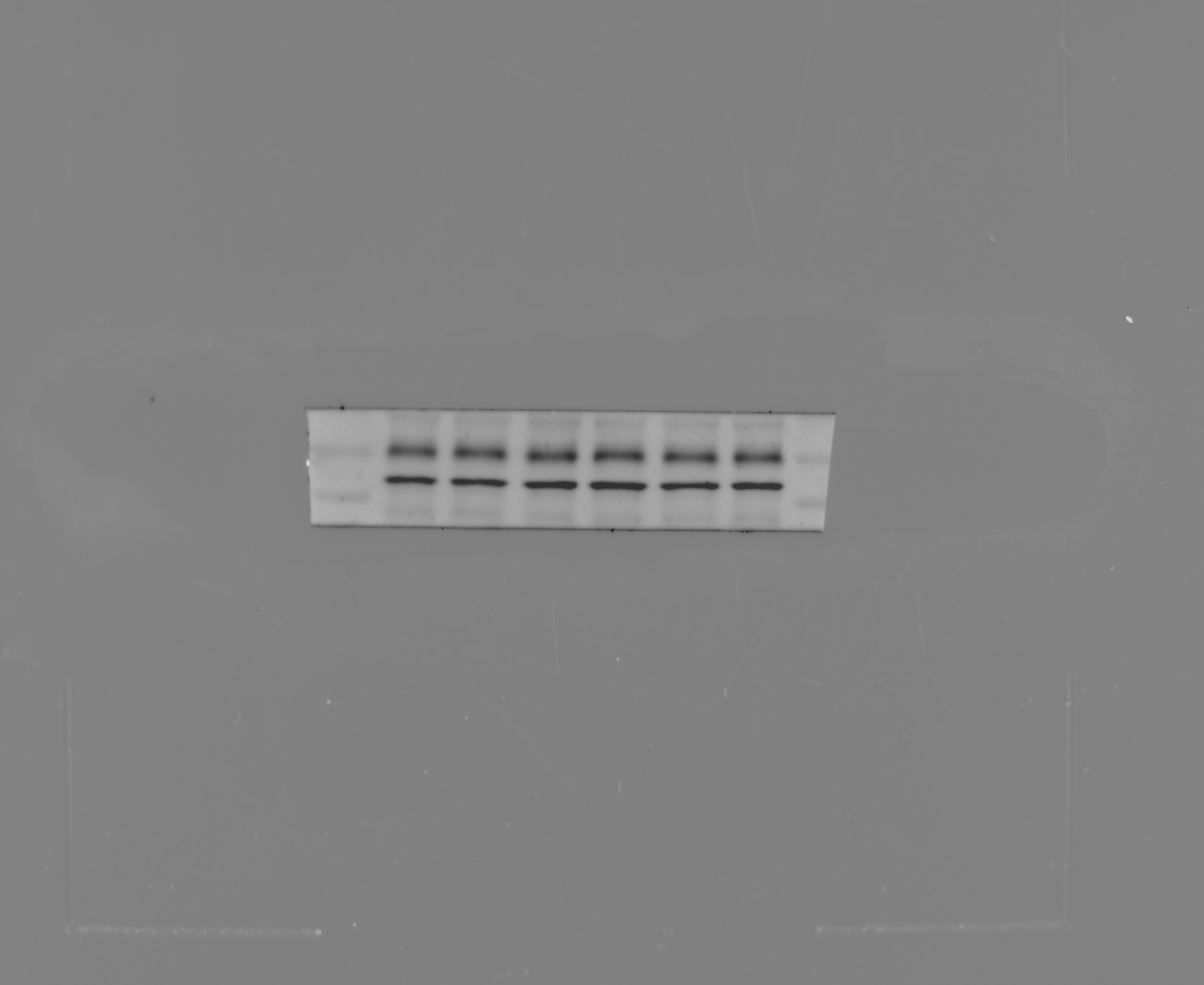

Supplement: Supplementary file 1 — Supplementary file1 (ZIP 36116 KB) [file 432_2024_5625_MOESM1_ESM.zip › Original Images for BlotsGels/3.Figure 3/LN229/4.STAT3/1/1-1-A.tif]

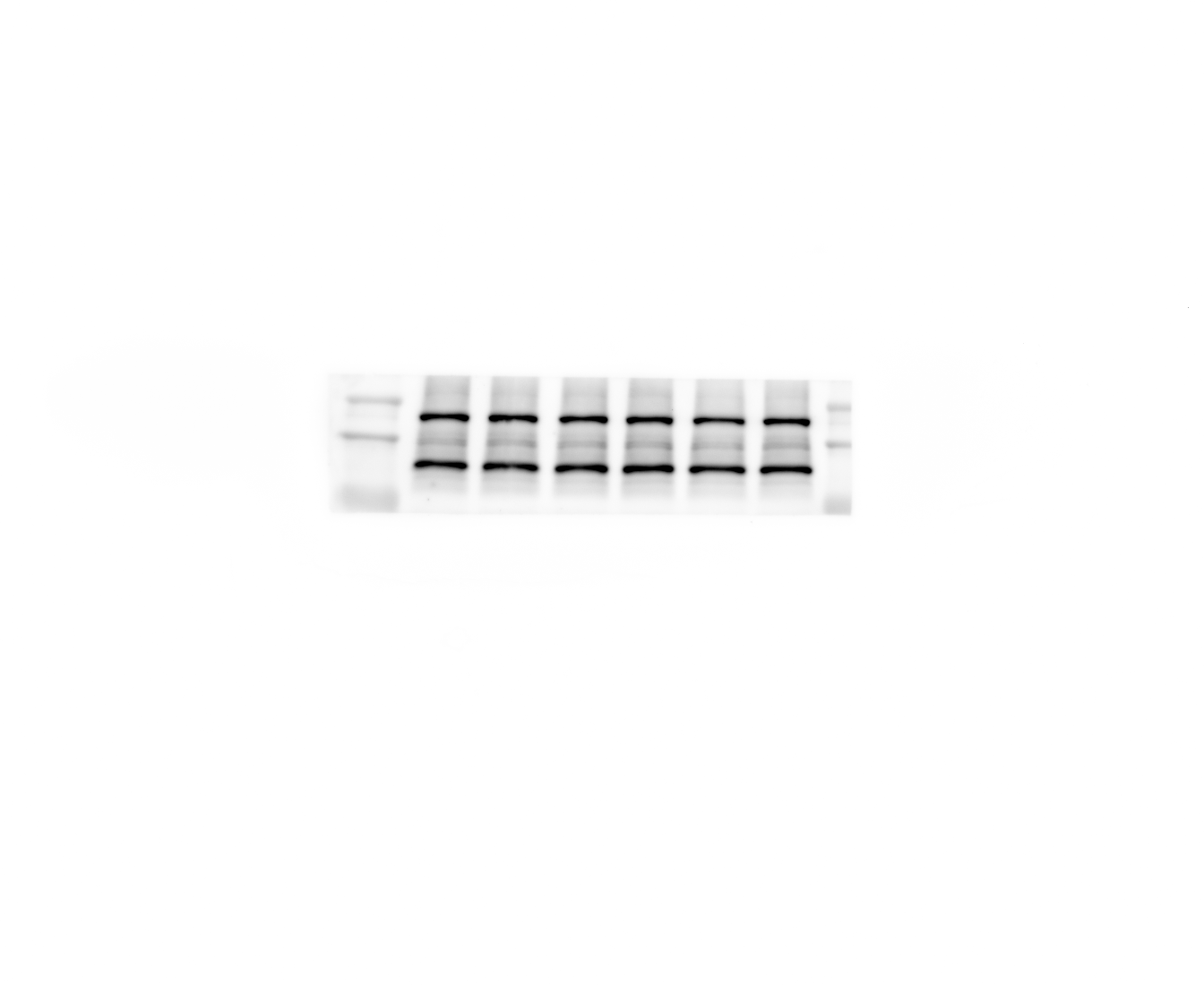

Supplement: Supplementary file 1 — Supplementary file1 (ZIP 36116 KB) [file 432_2024_5625_MOESM1_ESM.zip › Original Images for BlotsGels/3.Figure 3/LN229/4.STAT3/1/1-1-STAT3(Y).tif]

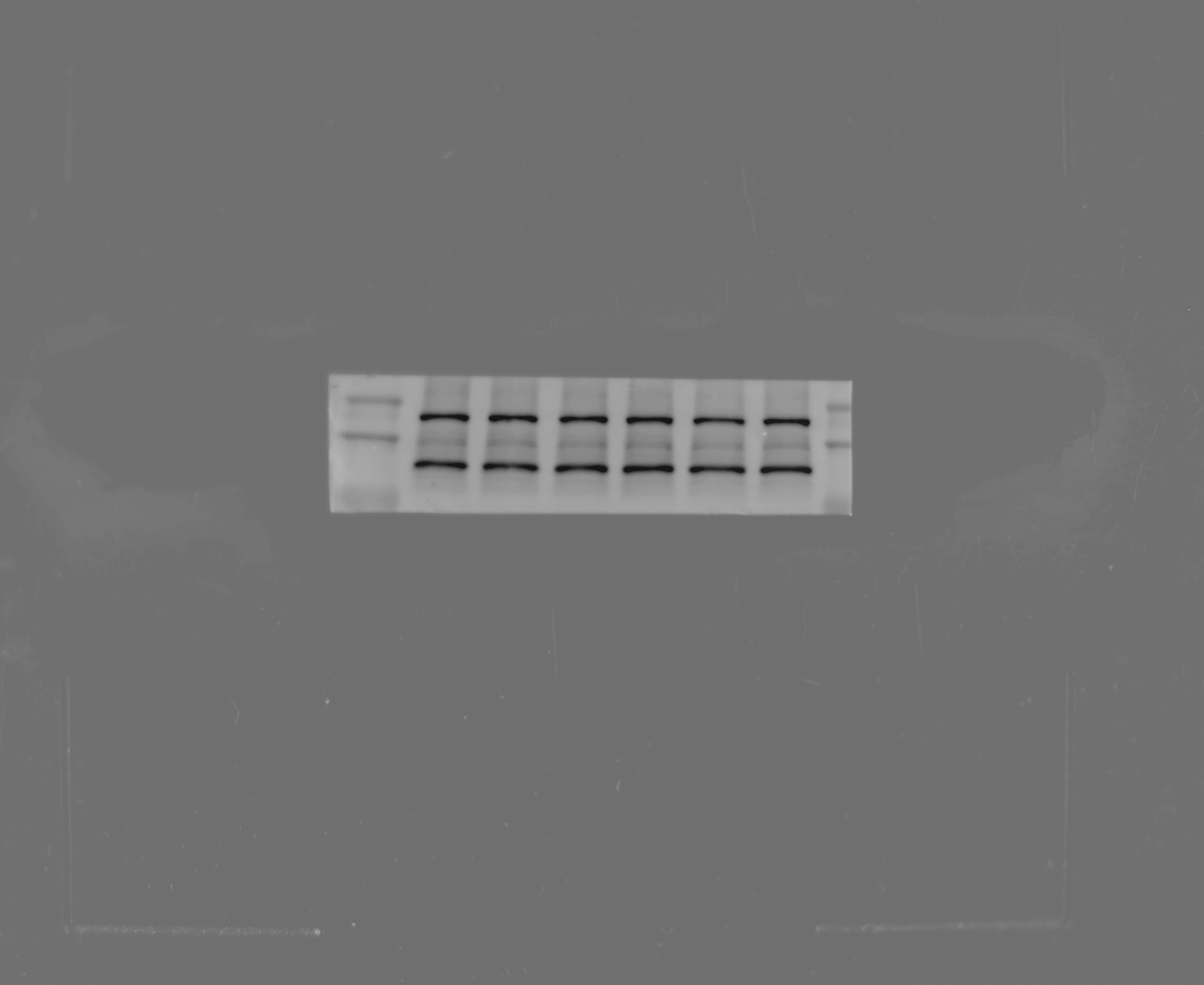

Supplement: Supplementary file 1 — Supplementary file1 (ZIP 36116 KB) [file 432_2024_5625_MOESM1_ESM.zip › Original Images for BlotsGels/3.Figure 3/LN229/4.STAT3/1/1-1-STAT3.tif]

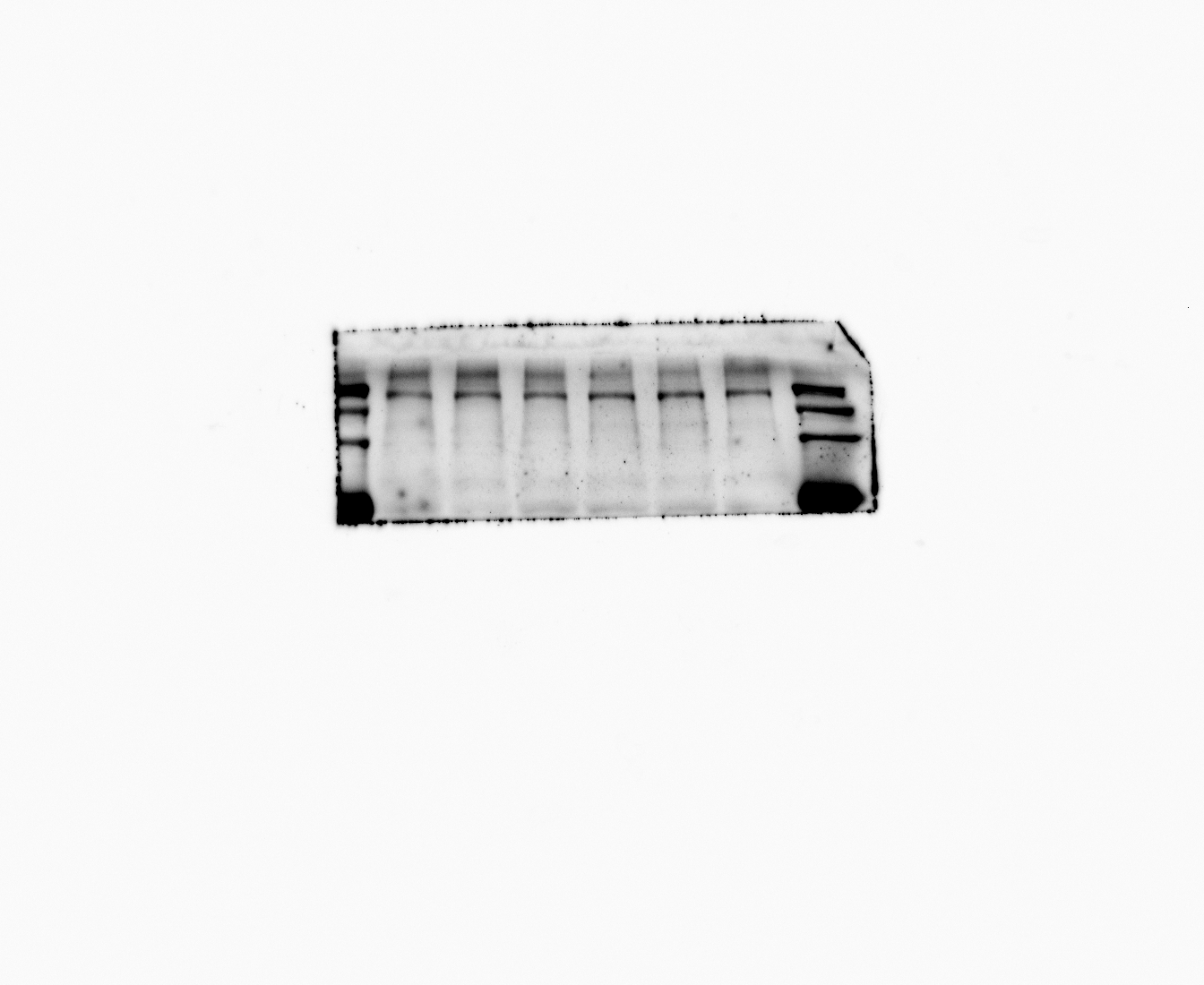

Supplement: Supplementary file 1 — Supplementary file1 (ZIP 36116 KB) [file 432_2024_5625_MOESM1_ESM.zip › Original Images for BlotsGels/3.Figure 3/U87/1.JAK2/1/2-1-JAK2(Y).tif]

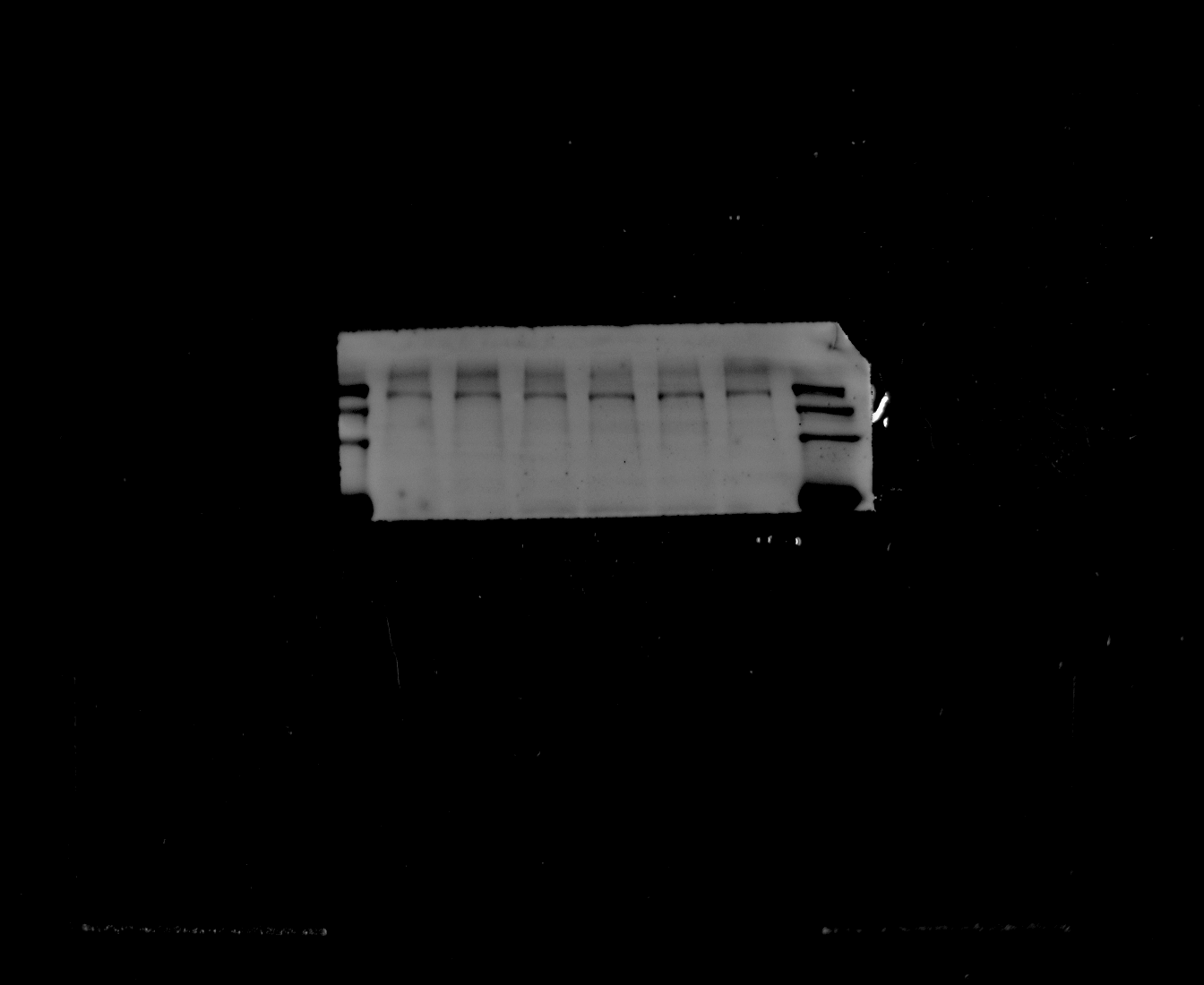

Supplement: Supplementary file 1 — Supplementary file1 (ZIP 36116 KB) [file 432_2024_5625_MOESM1_ESM.zip › Original Images for BlotsGels/3.Figure 3/U87/1.JAK2/1/2-1-JAK2.tif]

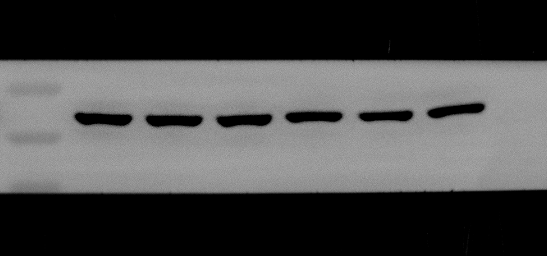

Supplement: Supplementary file 1 — Supplementary file1 (ZIP 36116 KB) [file 432_2024_5625_MOESM1_ESM.zip › Original Images for BlotsGels/3.Figure 3/U87/1.JAK2/1/2-1-β-anctin(叠加图)1.tif]

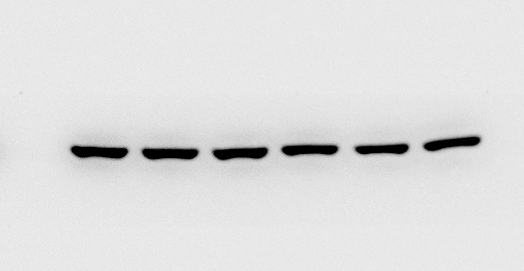

Supplement: Supplementary file 1 — Supplementary file1 (ZIP 36116 KB) [file 432_2024_5625_MOESM1_ESM.zip › Original Images for BlotsGels/3.Figure 3/U87/1.JAK2/1/2-1-β-anctin(样品图).tif]

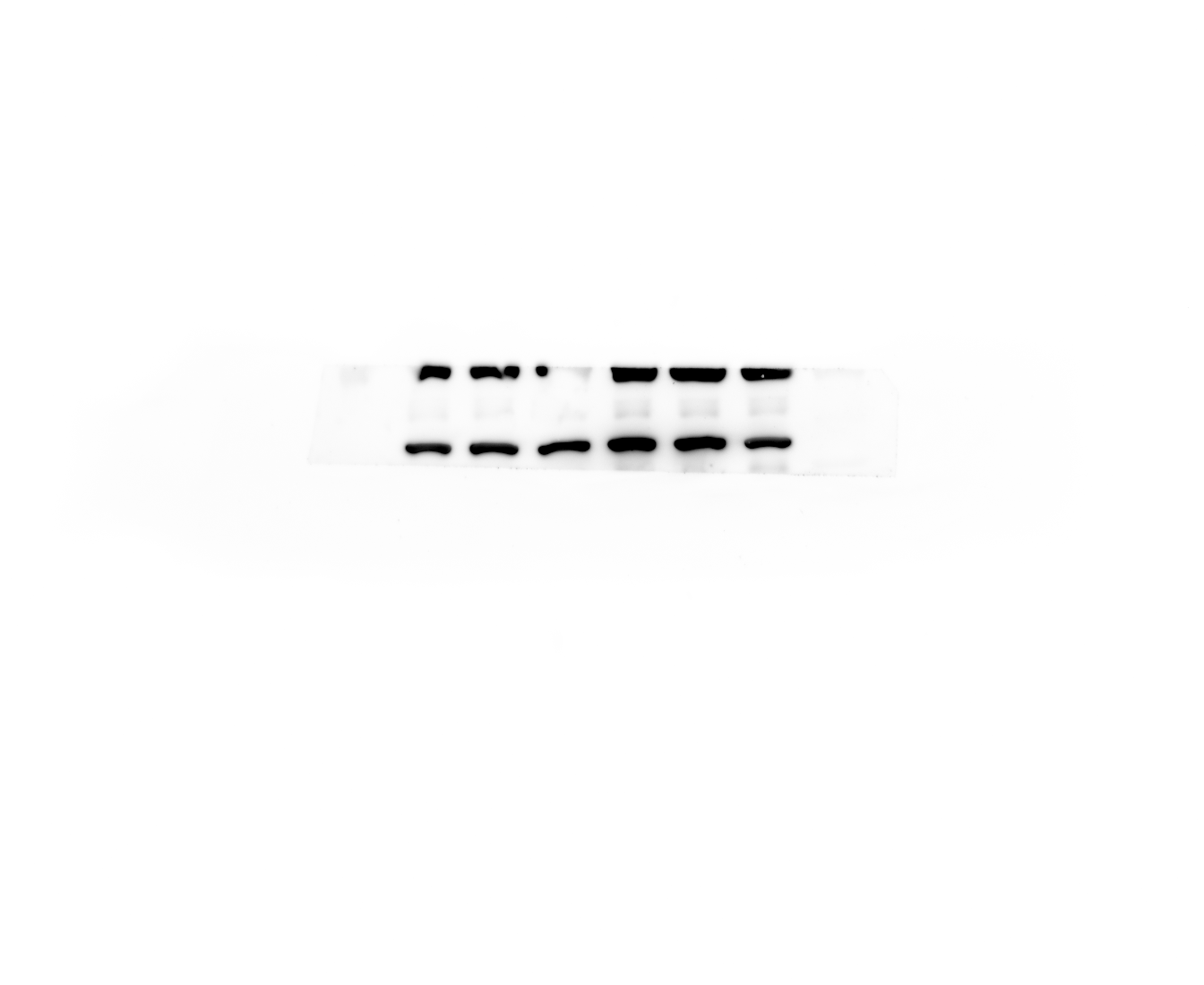

Supplement: Supplementary file 1 — Supplementary file1 (ZIP 36116 KB) [file 432_2024_5625_MOESM1_ESM.zip › Original Images for BlotsGels/3.Figure 3/U87/2.P-JAK2/1/2-2-A(Y).tif]

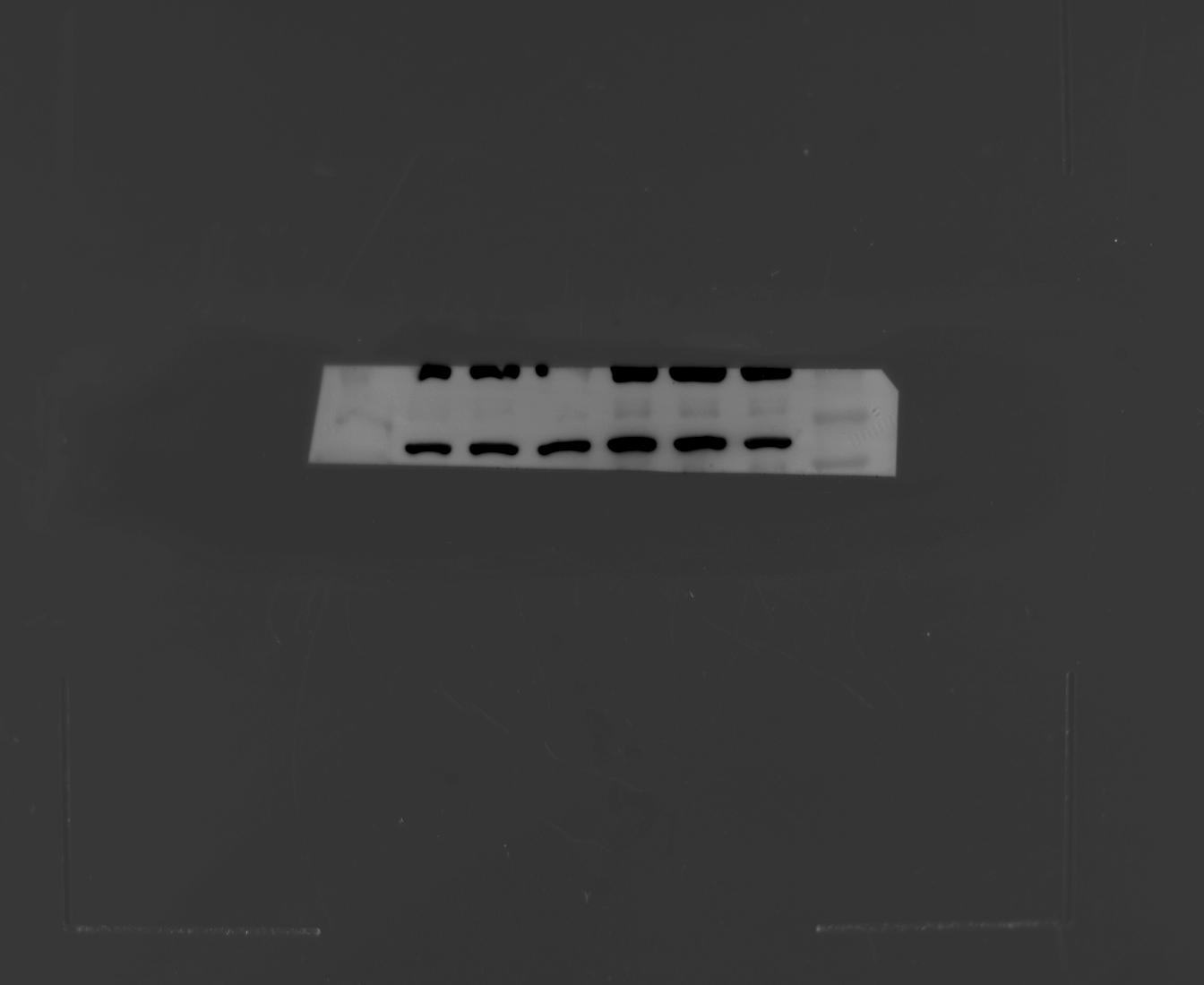

Supplement: Supplementary file 1 — Supplementary file1 (ZIP 36116 KB) [file 432_2024_5625_MOESM1_ESM.zip › Original Images for BlotsGels/3.Figure 3/U87/2.P-JAK2/1/2-2-A.tif]

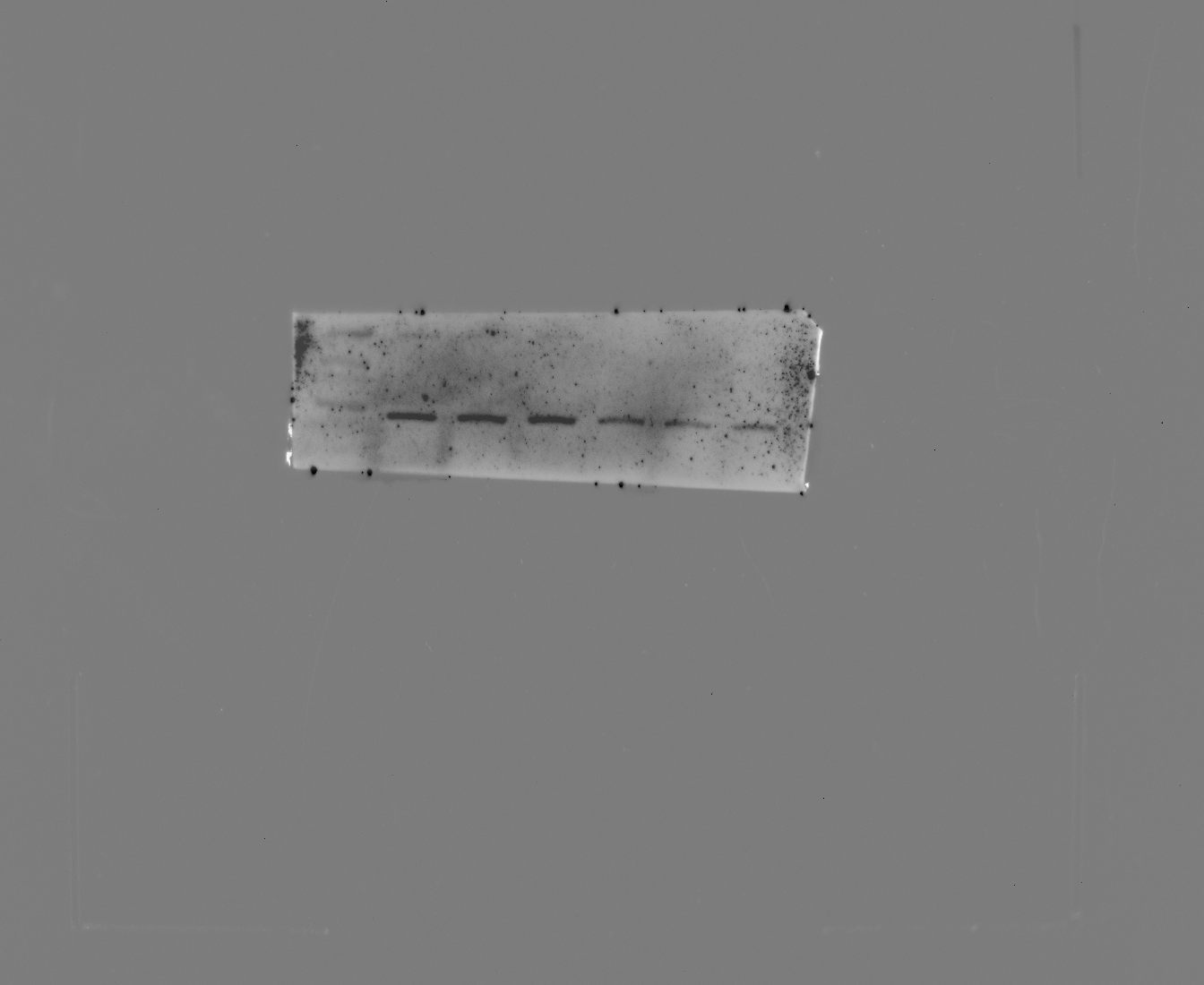

Supplement: Supplementary file 1 — Supplementary file1 (ZIP 36116 KB) [file 432_2024_5625_MOESM1_ESM.zip › Original Images for BlotsGels/3.Figure 3/U87/2.P-JAK2/1/2-2-P-JAK2(叠加图).tif]

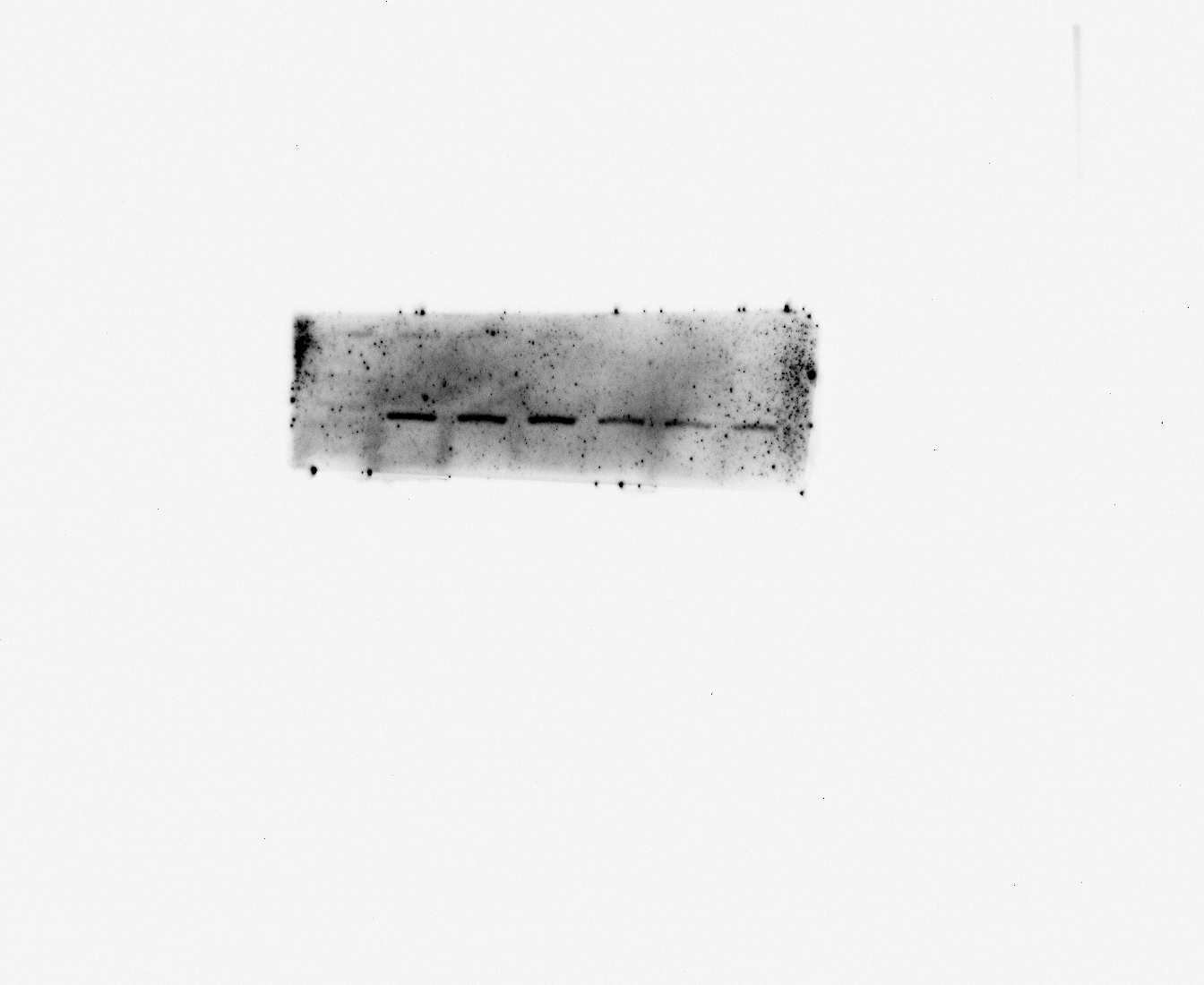

Supplement: Supplementary file 1 — Supplementary file1 (ZIP 36116 KB) [file 432_2024_5625_MOESM1_ESM.zip › Original Images for BlotsGels/3.Figure 3/U87/2.P-JAK2/1/2-2-P-JAK2(样品图).tif]

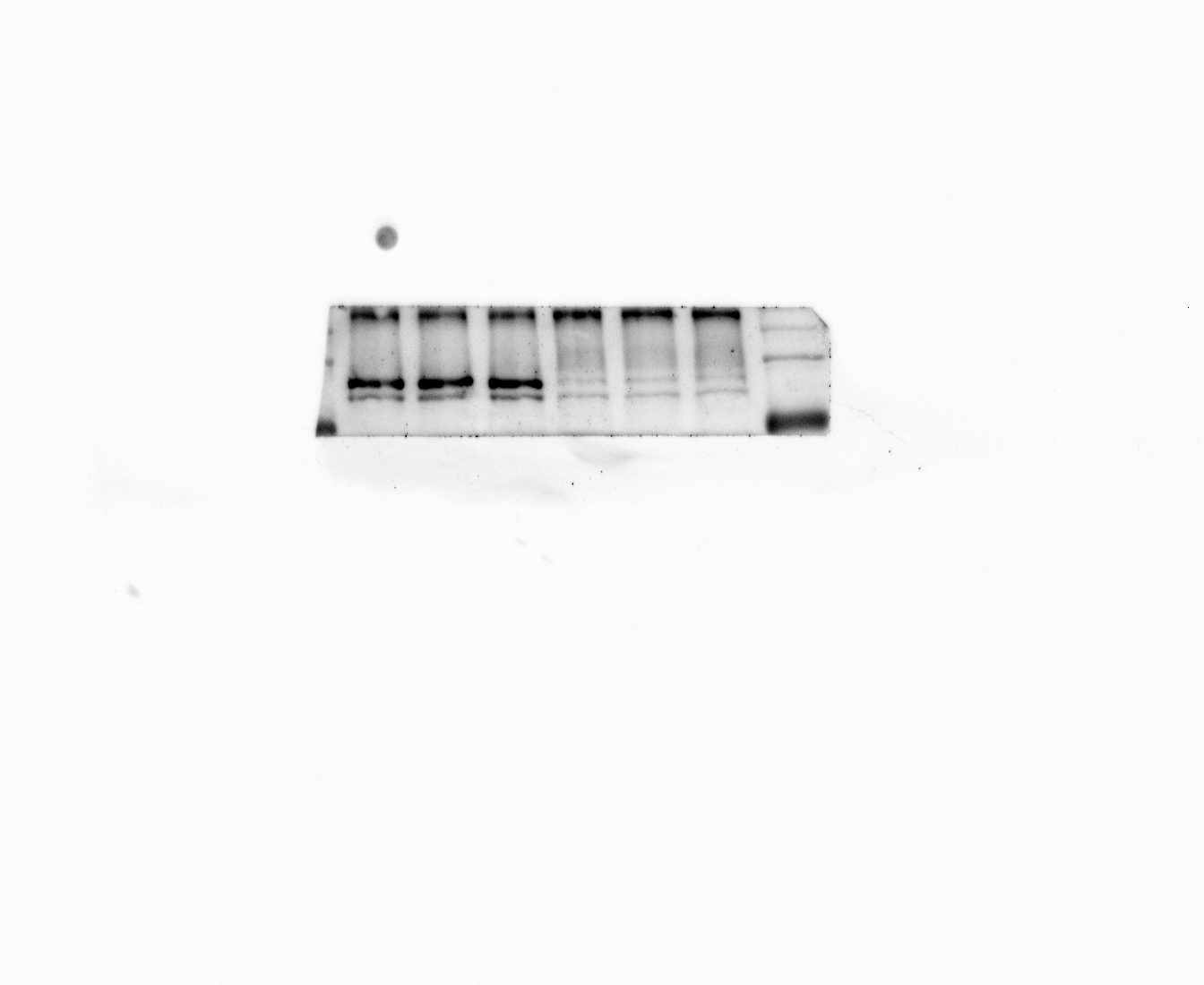

Supplement: Supplementary file 1 — Supplementary file1 (ZIP 36116 KB) [file 432_2024_5625_MOESM1_ESM.zip › Original Images for BlotsGels/3.Figure 3/U87/3.P-STAT3/1/1-2-P-STAT3(Y).tif]

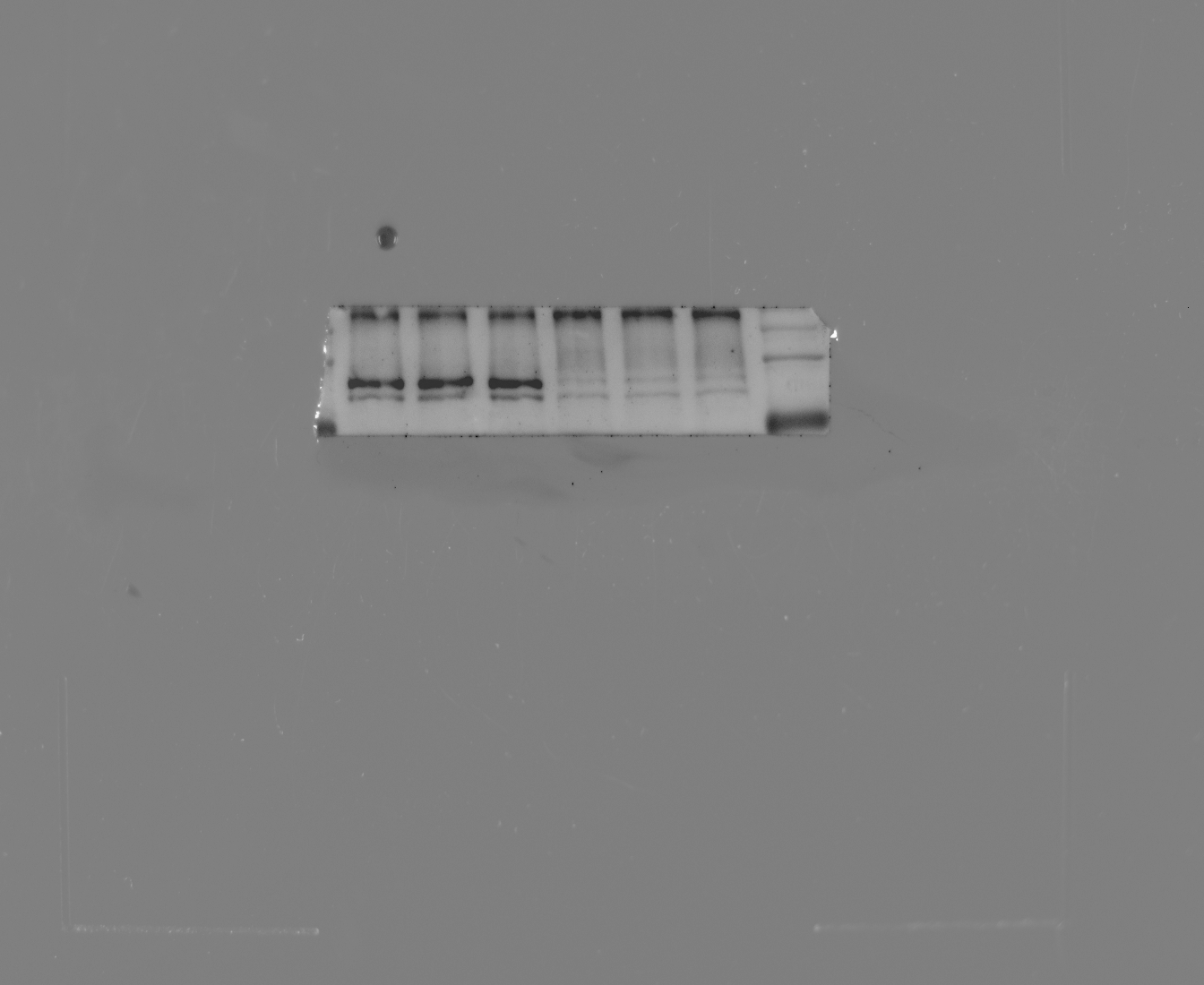

Supplement: Supplementary file 1 — Supplementary file1 (ZIP 36116 KB) [file 432_2024_5625_MOESM1_ESM.zip › Original Images for BlotsGels/3.Figure 3/U87/3.P-STAT3/1/1-2-P-STAT3.tif]

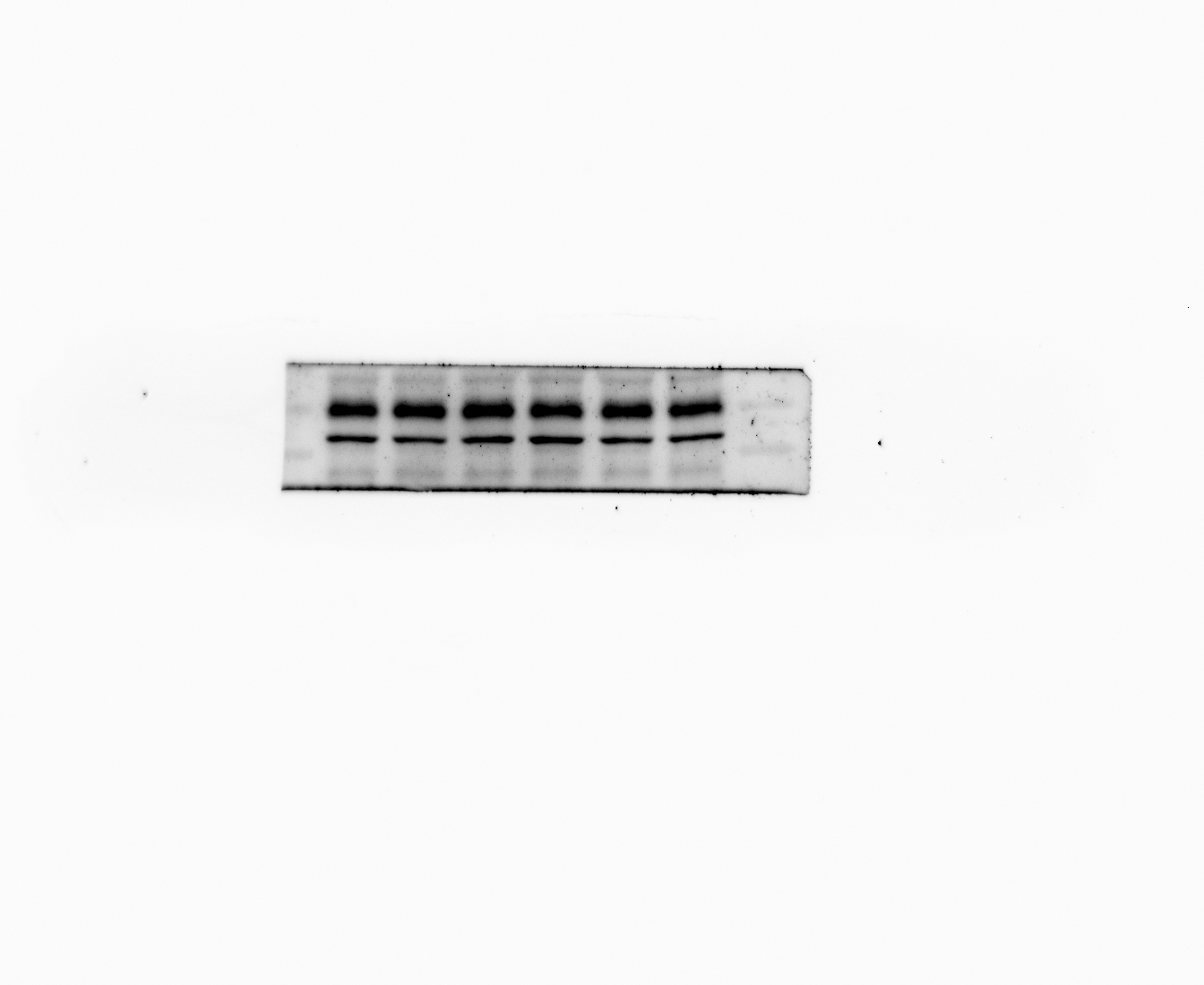

Supplement: Supplementary file 1 — Supplementary file1 (ZIP 36116 KB) [file 432_2024_5625_MOESM1_ESM.zip › Original Images for BlotsGels/3.Figure 3/U87/4.STAT3/1/2-2-A(Y).tif]

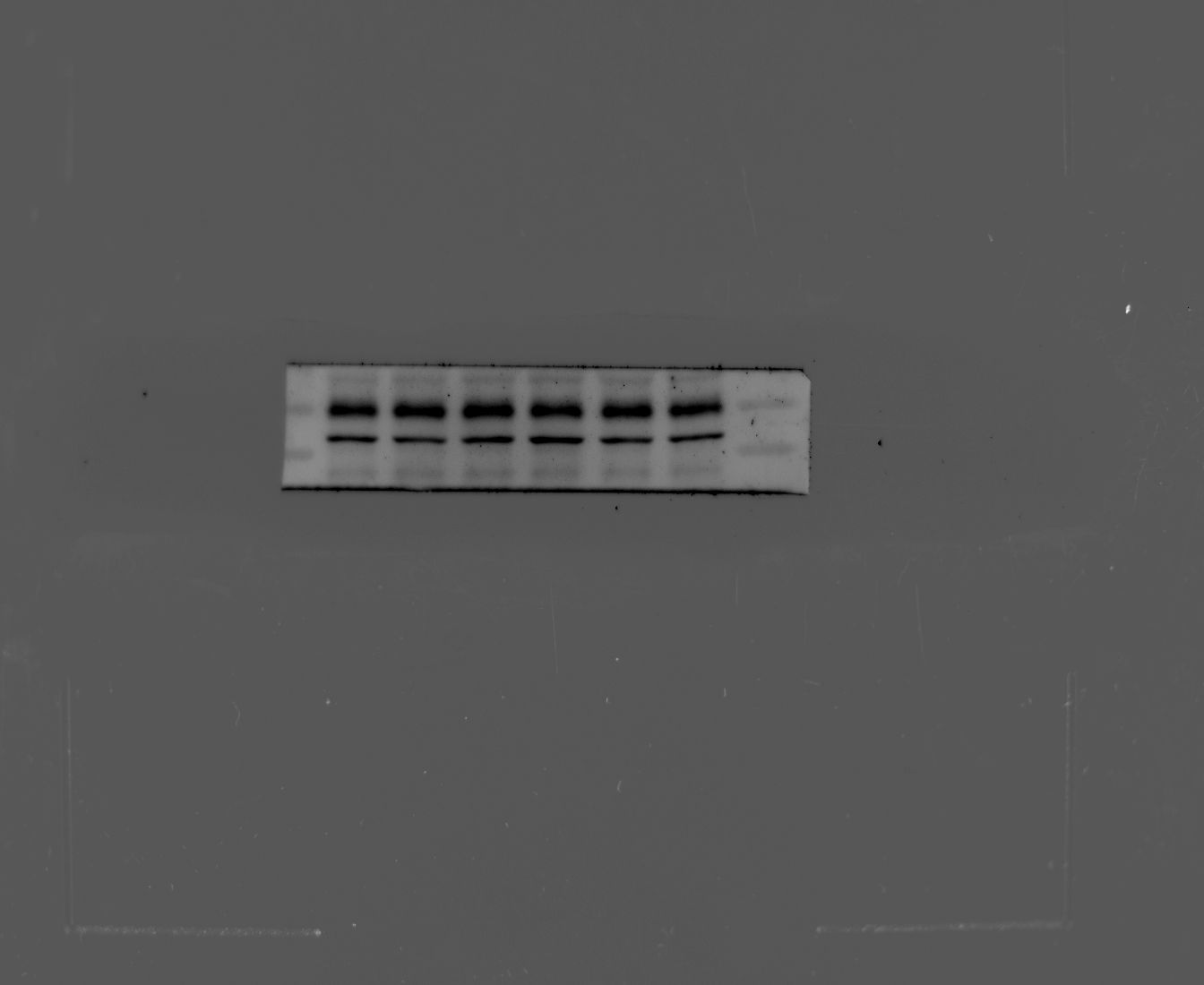

Supplement: Supplementary file 1 — Supplementary file1 (ZIP 36116 KB) [file 432_2024_5625_MOESM1_ESM.zip › Original Images for BlotsGels/3.Figure 3/U87/4.STAT3/1/2-2-A.tif]

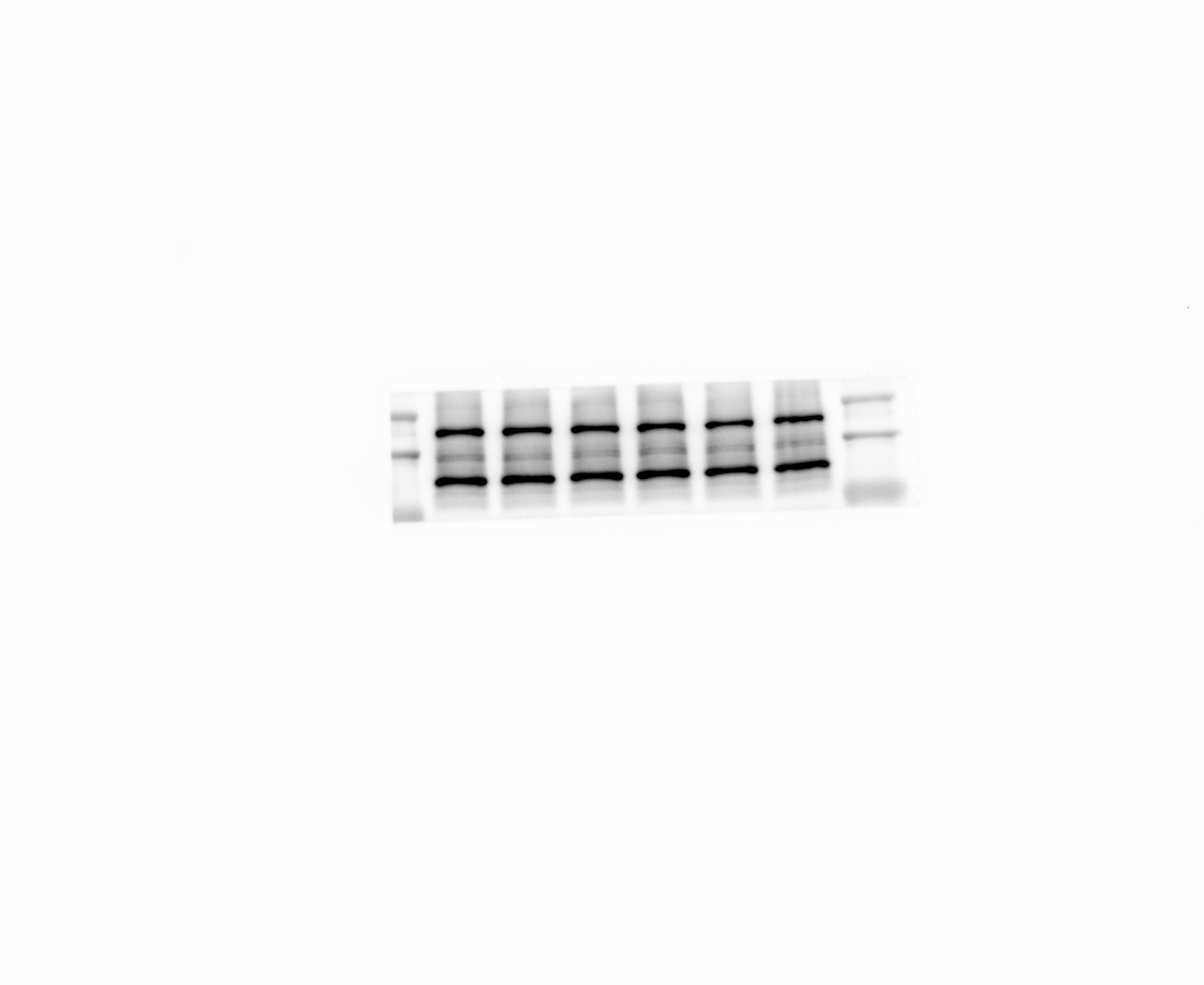

Supplement: Supplementary file 1 — Supplementary file1 (ZIP 36116 KB) [file 432_2024_5625_MOESM1_ESM.zip › Original Images for BlotsGels/3.Figure 3/U87/4.STAT3/1/2-2-STAT3(Y).tif]

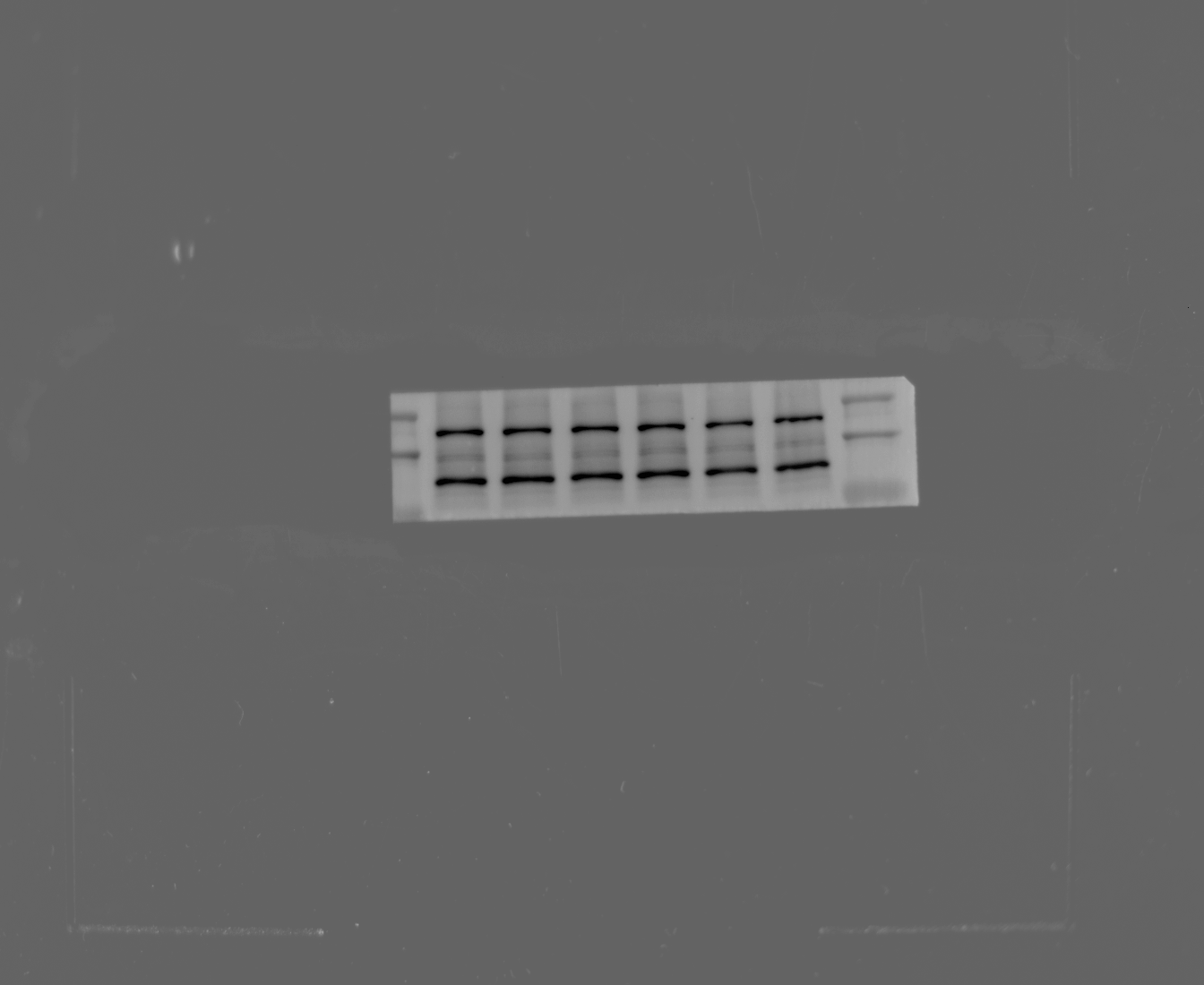

Supplement: Supplementary file 1 — Supplementary file1 (ZIP 36116 KB) [file 432_2024_5625_MOESM1_ESM.zip › Original Images for BlotsGels/3.Figure 3/U87/4.STAT3/1/2-2-STAT3.tif]

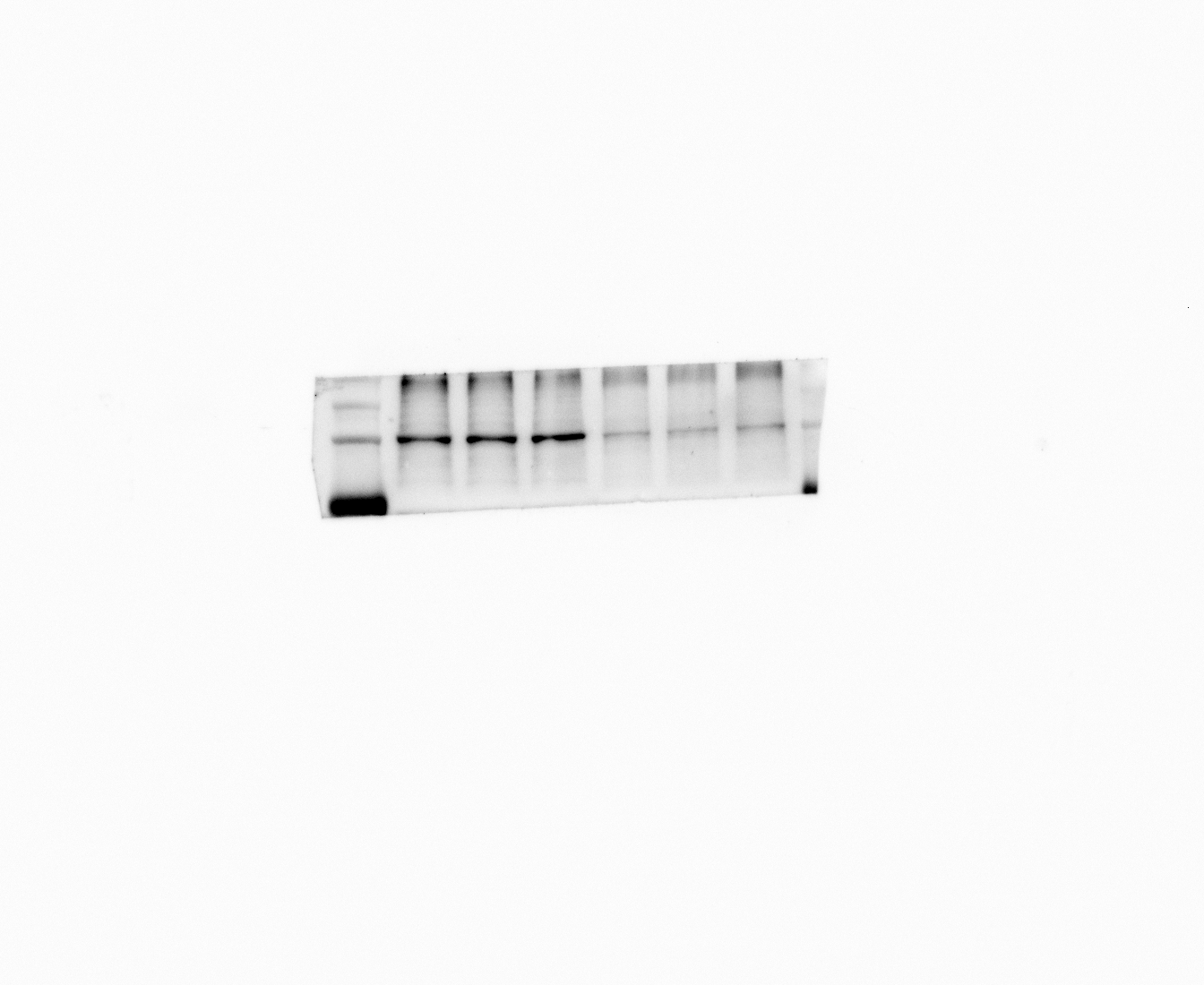

Supplement: Supplementary file 1 — Supplementary file1 (ZIP 36116 KB) [file 432_2024_5625_MOESM1_ESM.zip › Original Images for BlotsGels/4.Figure 4/LN229/1.NLRP3/1/1.NLRP3(Y).tif]

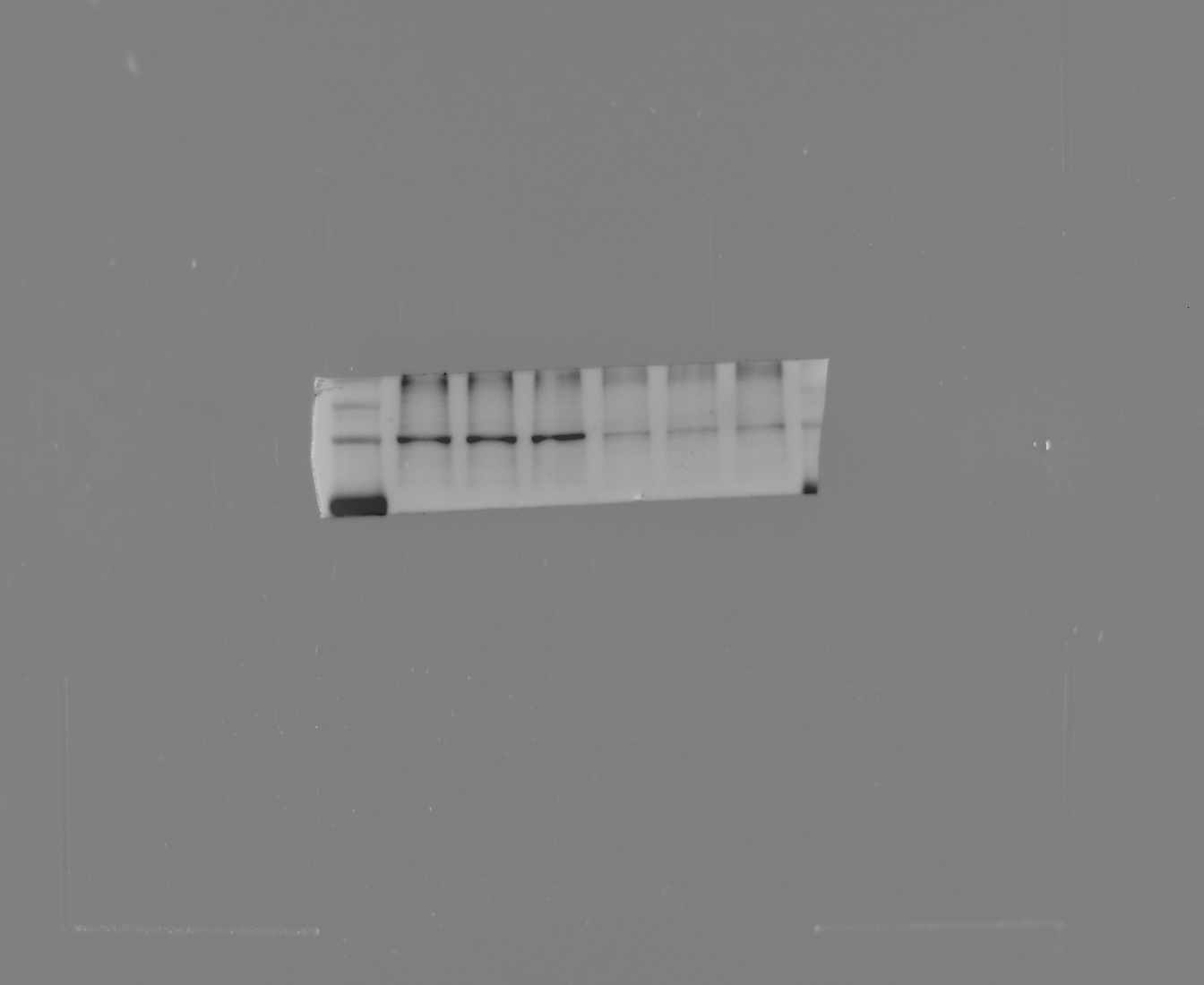

Supplement: Supplementary file 1 — Supplementary file1 (ZIP 36116 KB) [file 432_2024_5625_MOESM1_ESM.zip › Original Images for BlotsGels/4.Figure 4/LN229/1.NLRP3/1/1.NLRP3.tif]

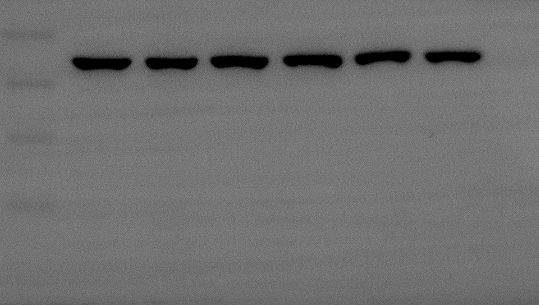

Supplement: Supplementary file 1 — Supplementary file1 (ZIP 36116 KB) [file 432_2024_5625_MOESM1_ESM.zip › Original Images for BlotsGels/4.Figure 4/LN229/1.NLRP3/1/1.β-anctin(叠加图).tif]

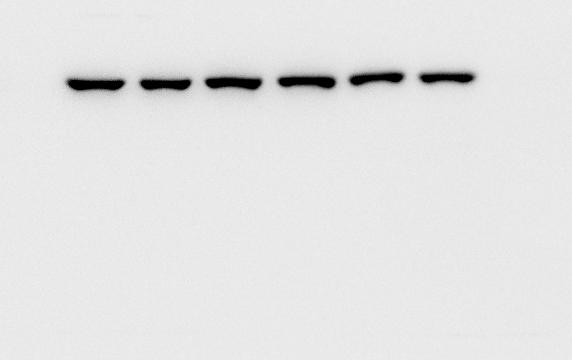

Supplement: Supplementary file 1 — Supplementary file1 (ZIP 36116 KB) [file 432_2024_5625_MOESM1_ESM.zip › Original Images for BlotsGels/4.Figure 4/LN229/1.NLRP3/1/1.β-anctin(样品图).tif]

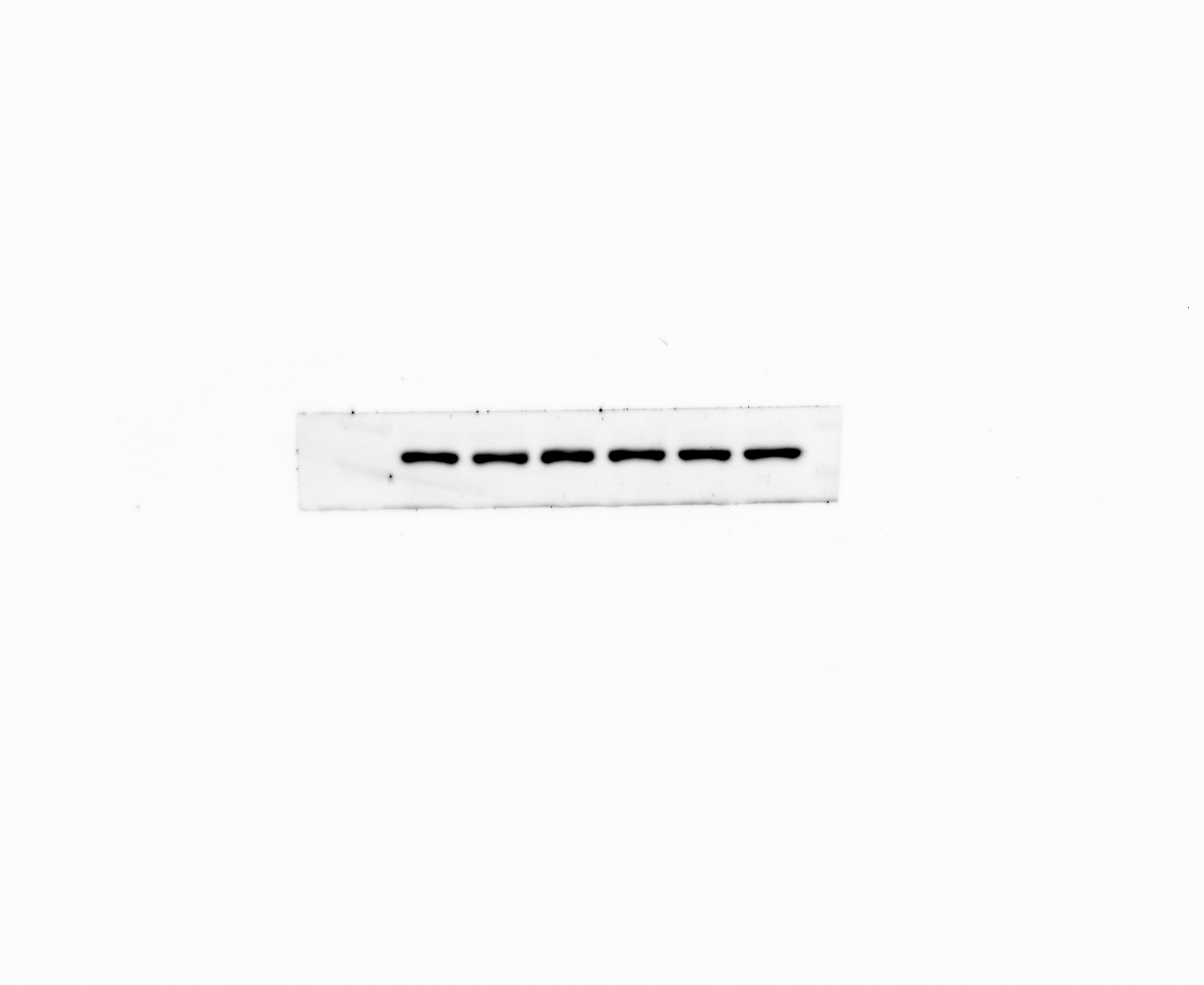

Supplement: Supplementary file 1 — Supplementary file1 (ZIP 36116 KB) [file 432_2024_5625_MOESM1_ESM.zip › Original Images for BlotsGels/4.Figure 4/LN229/2.IL1/il1/4-1-A(Y).tif]

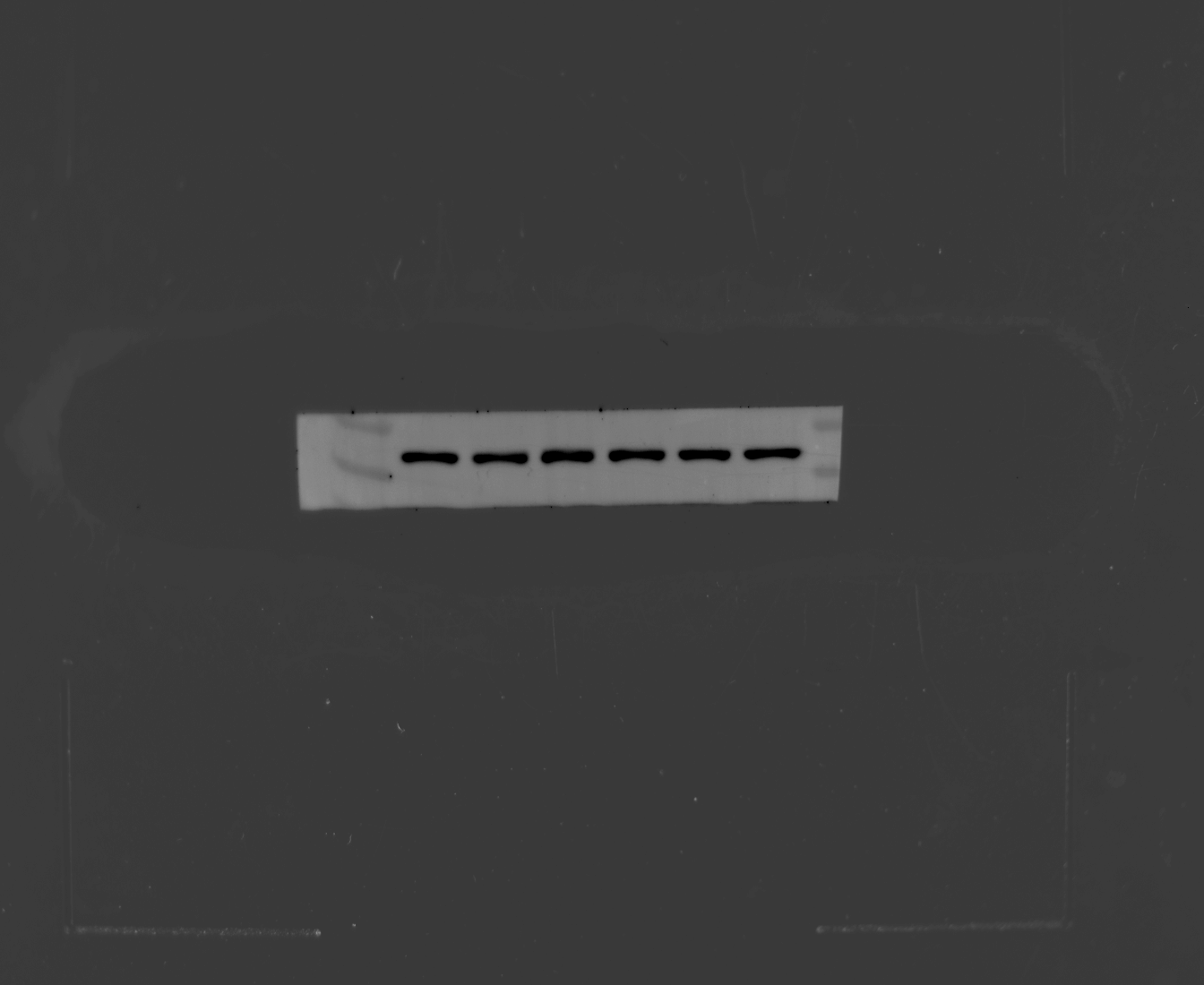

Supplement: Supplementary file 1 — Supplementary file1 (ZIP 36116 KB) [file 432_2024_5625_MOESM1_ESM.zip › Original Images for BlotsGels/4.Figure 4/LN229/2.IL1/il1/4-1-A.tif]

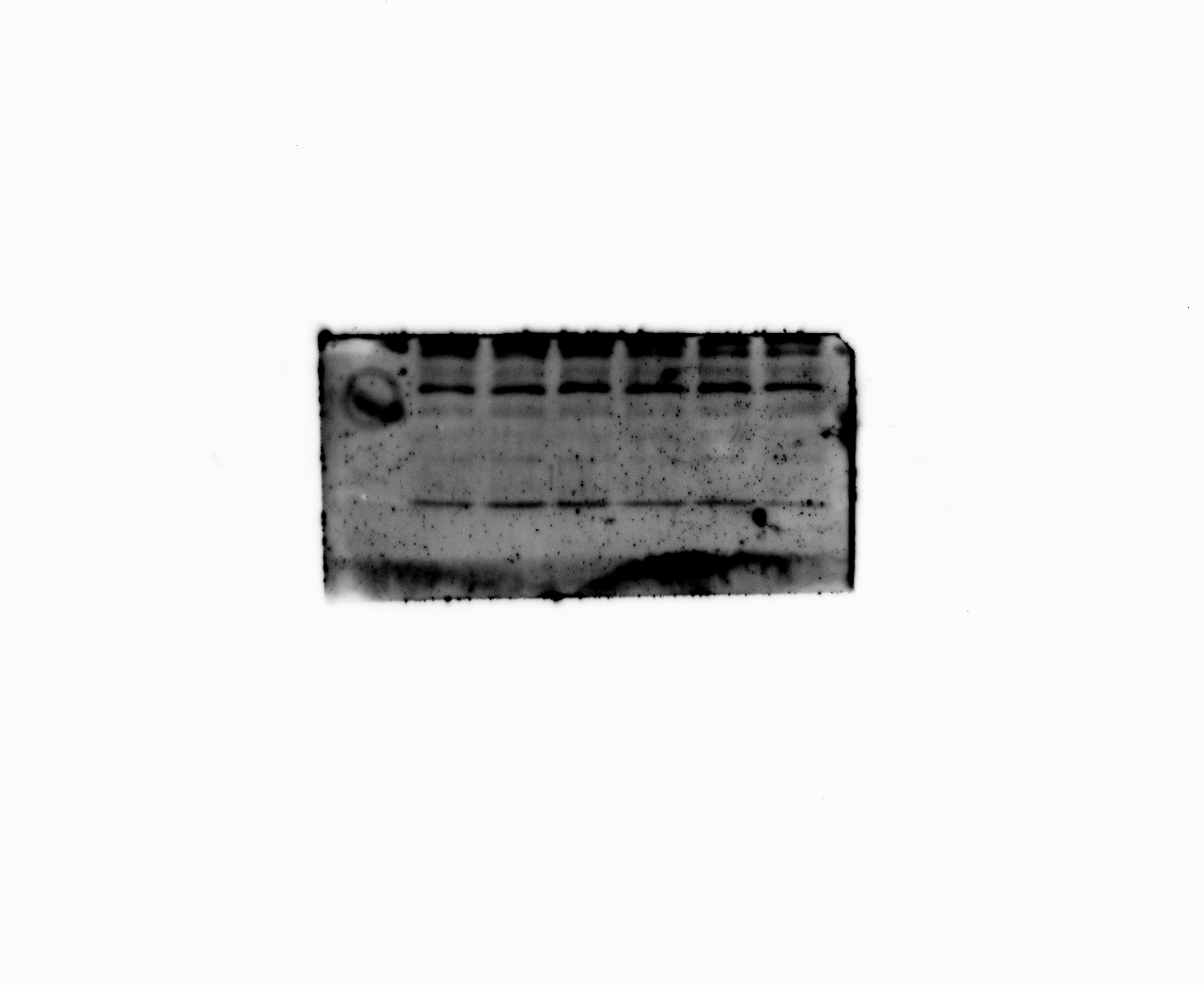

Supplement: Supplementary file 1 — Supplementary file1 (ZIP 36116 KB) [file 432_2024_5625_MOESM1_ESM.zip › Original Images for BlotsGels/4.Figure 4/LN229/2.IL1/il1/4-1-IL1(Y).tif]

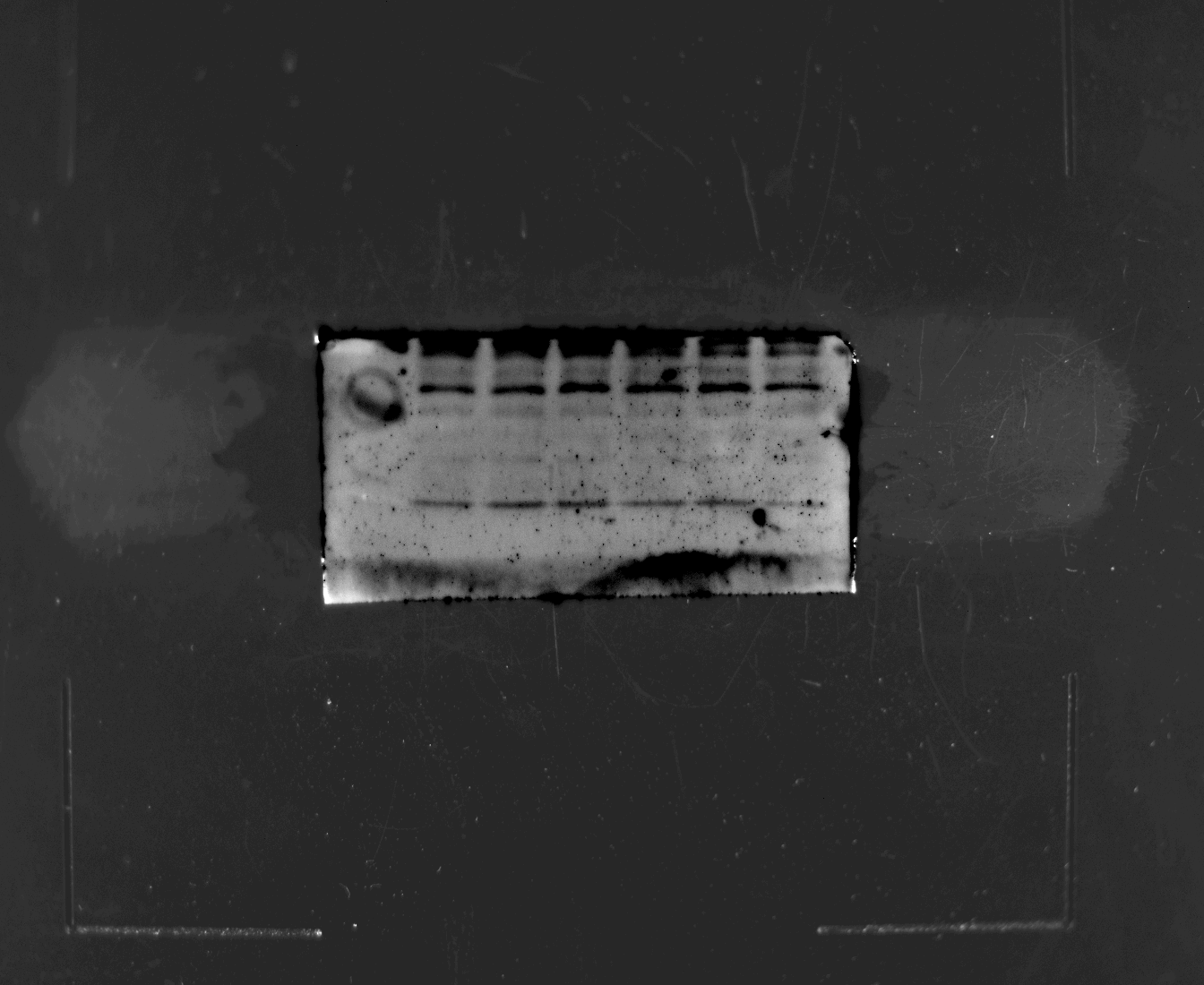

Supplement: Supplementary file 1 — Supplementary file1 (ZIP 36116 KB) [file 432_2024_5625_MOESM1_ESM.zip › Original Images for BlotsGels/4.Figure 4/LN229/2.IL1/il1/4-1-IL1.tif]

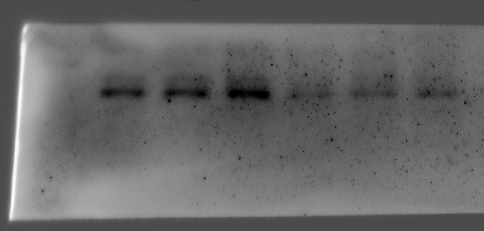

Supplement: Supplementary file 1 — Supplementary file1 (ZIP 36116 KB) [file 432_2024_5625_MOESM1_ESM.zip › Original Images for BlotsGels/4.Figure 4/LN229/3.IL18/1/1.IL18(叠加图).tif]

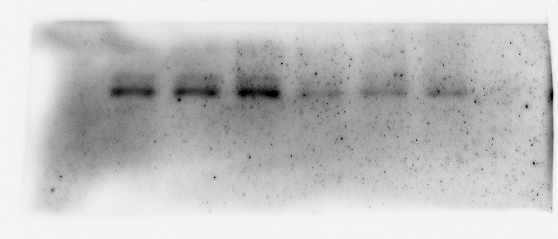

Supplement: Supplementary file 1 — Supplementary file1 (ZIP 36116 KB) [file 432_2024_5625_MOESM1_ESM.zip › Original Images for BlotsGels/4.Figure 4/LN229/3.IL18/1/1.IL18(样品图).tif]

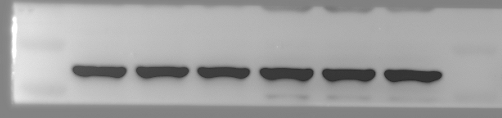

Supplement: Supplementary file 1 — Supplementary file1 (ZIP 36116 KB) [file 432_2024_5625_MOESM1_ESM.zip › Original Images for BlotsGels/4.Figure 4/LN229/3.IL18/1/1.β-anctin(叠加图).tif]

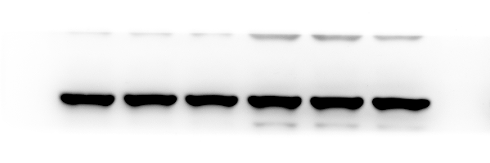

Supplement: Supplementary file 1 — Supplementary file1 (ZIP 36116 KB) [file 432_2024_5625_MOESM1_ESM.zip › Original Images for BlotsGels/4.Figure 4/LN229/3.IL18/1/1.β-anctin(样品图).tif]

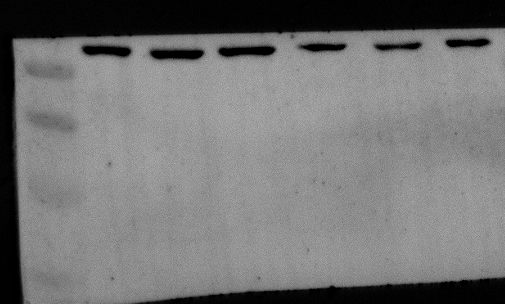

Supplement: Supplementary file 1 — Supplementary file1 (ZIP 36116 KB) [file 432_2024_5625_MOESM1_ESM.zip › Original Images for BlotsGels/4.Figure 4/LN229/4.IL6/1/1.IL6(叠加图).tif]

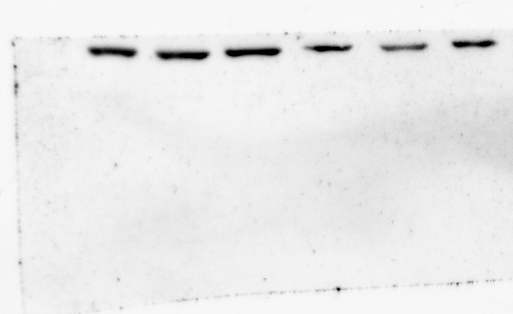

Supplement: Supplementary file 1 — Supplementary file1 (ZIP 36116 KB) [file 432_2024_5625_MOESM1_ESM.zip › Original Images for BlotsGels/4.Figure 4/LN229/4.IL6/1/1.IL6(样品图).tif]

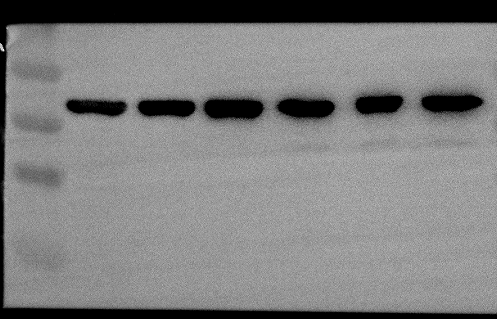

Supplement: Supplementary file 1 — Supplementary file1 (ZIP 36116 KB) [file 432_2024_5625_MOESM1_ESM.zip › Original Images for BlotsGels/4.Figure 4/LN229/4.IL6/1/1.β-anctin(叠加图).tif]

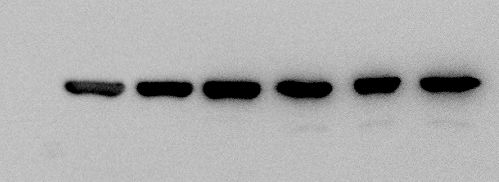

Supplement: Supplementary file 1 — Supplementary file1 (ZIP 36116 KB) [file 432_2024_5625_MOESM1_ESM.zip › Original Images for BlotsGels/4.Figure 4/LN229/4.IL6/1/1.β-anctin(样品图).tif]

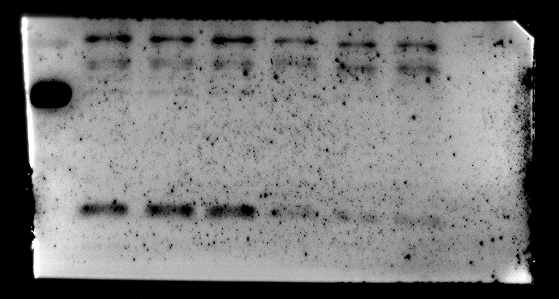

Supplement: Supplementary file 1 — Supplementary file1 (ZIP 36116 KB) [file 432_2024_5625_MOESM1_ESM.zip › Original Images for BlotsGels/4.Figure 4/LN229/5.TNFα/1/1.TNFα(叠加图).tif]

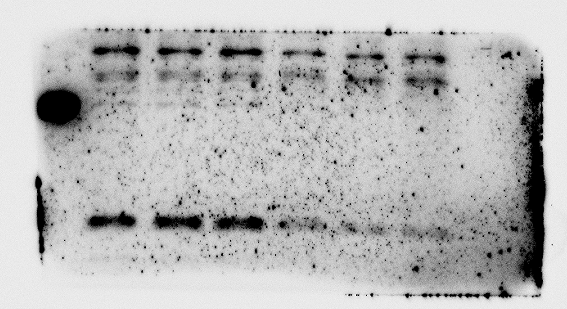

Supplement: Supplementary file 1 — Supplementary file1 (ZIP 36116 KB) [file 432_2024_5625_MOESM1_ESM.zip › Original Images for BlotsGels/4.Figure 4/LN229/5.TNFα/1/1.TNFα(样品图).tif]

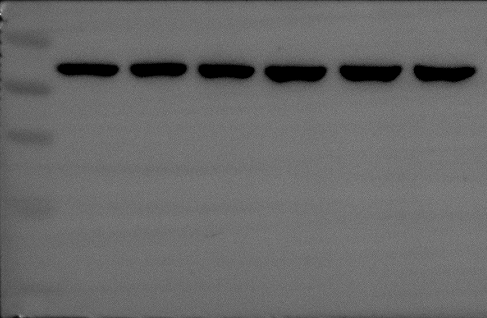

Supplement: Supplementary file 1 — Supplementary file1 (ZIP 36116 KB) [file 432_2024_5625_MOESM1_ESM.zip › Original Images for BlotsGels/4.Figure 4/LN229/5.TNFα/1/1.β-anctin(叠加图).tif]

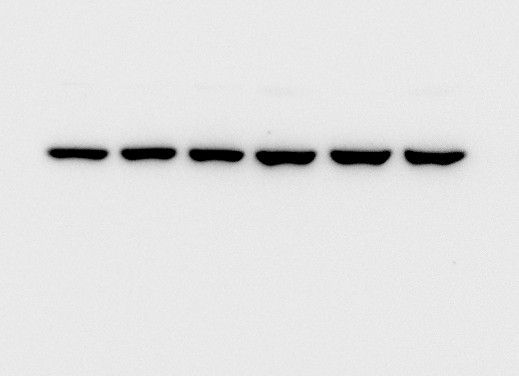

Supplement: Supplementary file 1 — Supplementary file1 (ZIP 36116 KB) [file 432_2024_5625_MOESM1_ESM.zip › Original Images for BlotsGels/4.Figure 4/LN229/5.TNFα/1/1.β-anctin(样品图).tif]

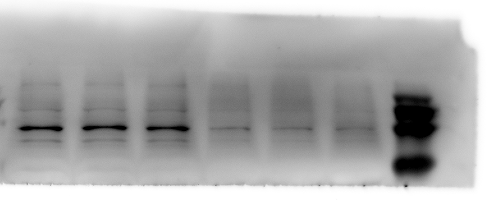

Supplement: Supplementary file 1 — Supplementary file1 (ZIP 36116 KB) [file 432_2024_5625_MOESM1_ESM.zip › Original Images for BlotsGels/4.Figure 4/U87/1.NLRP3/1/1.NLRP3(叠加图).tif]

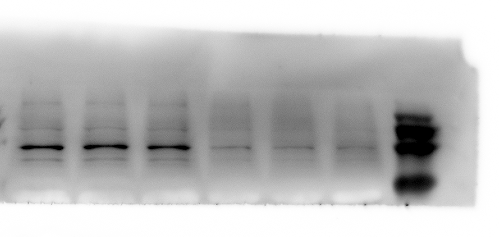

Supplement: Supplementary file 1 — Supplementary file1 (ZIP 36116 KB) [file 432_2024_5625_MOESM1_ESM.zip › Original Images for BlotsGels/4.Figure 4/U87/1.NLRP3/1/1.NLRP3(样品图).tif]

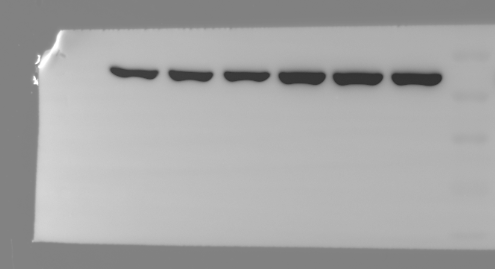

Supplement: Supplementary file 1 — Supplementary file1 (ZIP 36116 KB) [file 432_2024_5625_MOESM1_ESM.zip › Original Images for BlotsGels/4.Figure 4/U87/1.NLRP3/1/1.β-actin(叠加图).tif]

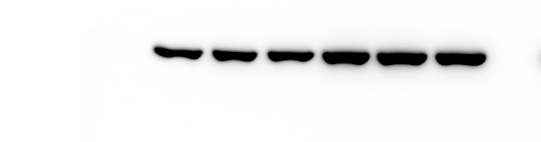

Supplement: Supplementary file 1 — Supplementary file1 (ZIP 36116 KB) [file 432_2024_5625_MOESM1_ESM.zip › Original Images for BlotsGels/4.Figure 4/U87/1.NLRP3/1/1.β-actin(样品图).tif]

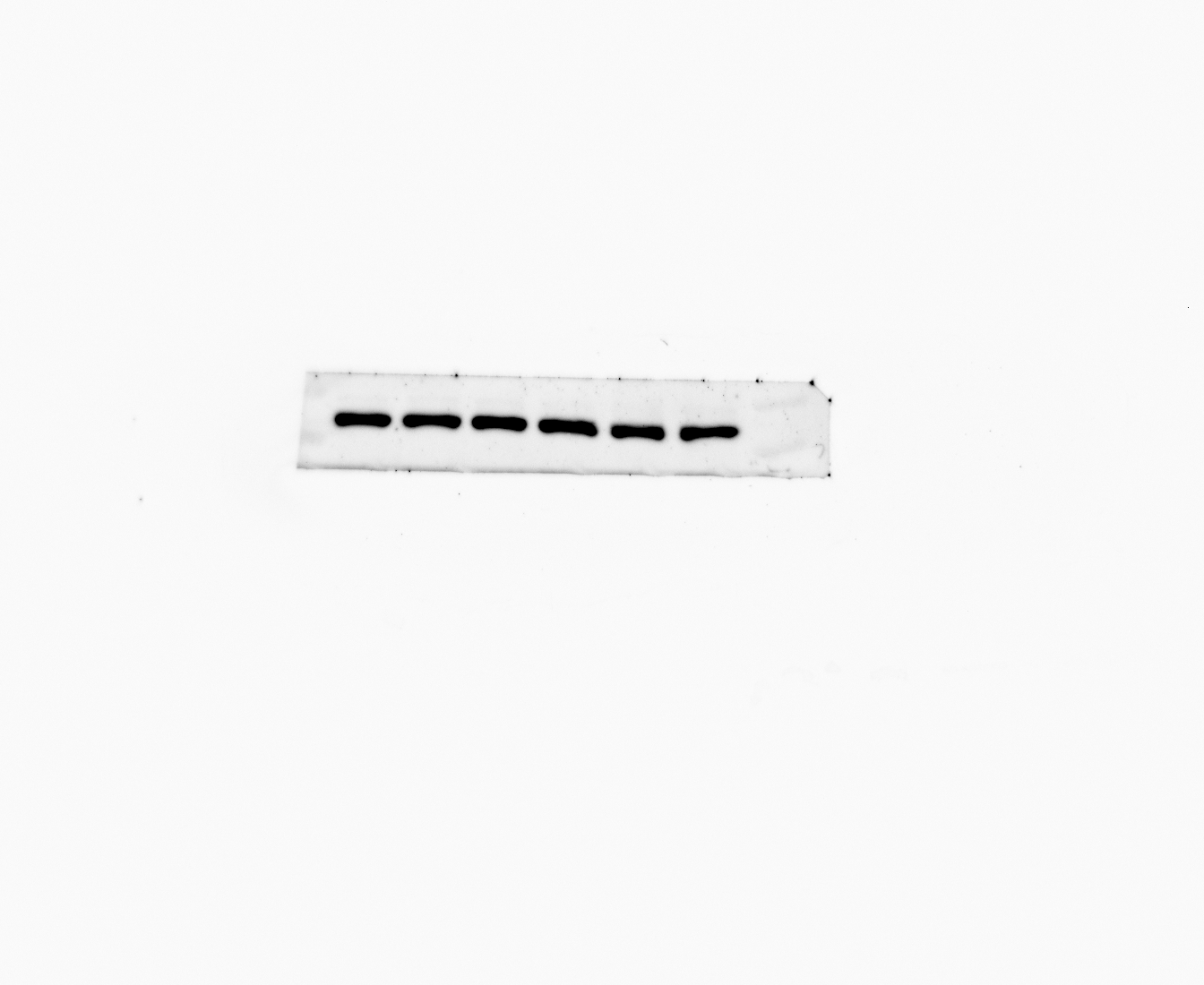

Supplement: Supplementary file 1 — Supplementary file1 (ZIP 36116 KB) [file 432_2024_5625_MOESM1_ESM.zip › Original Images for BlotsGels/4.Figure 4/U87/2.c-il1/2/4-2-A(Y).tif]

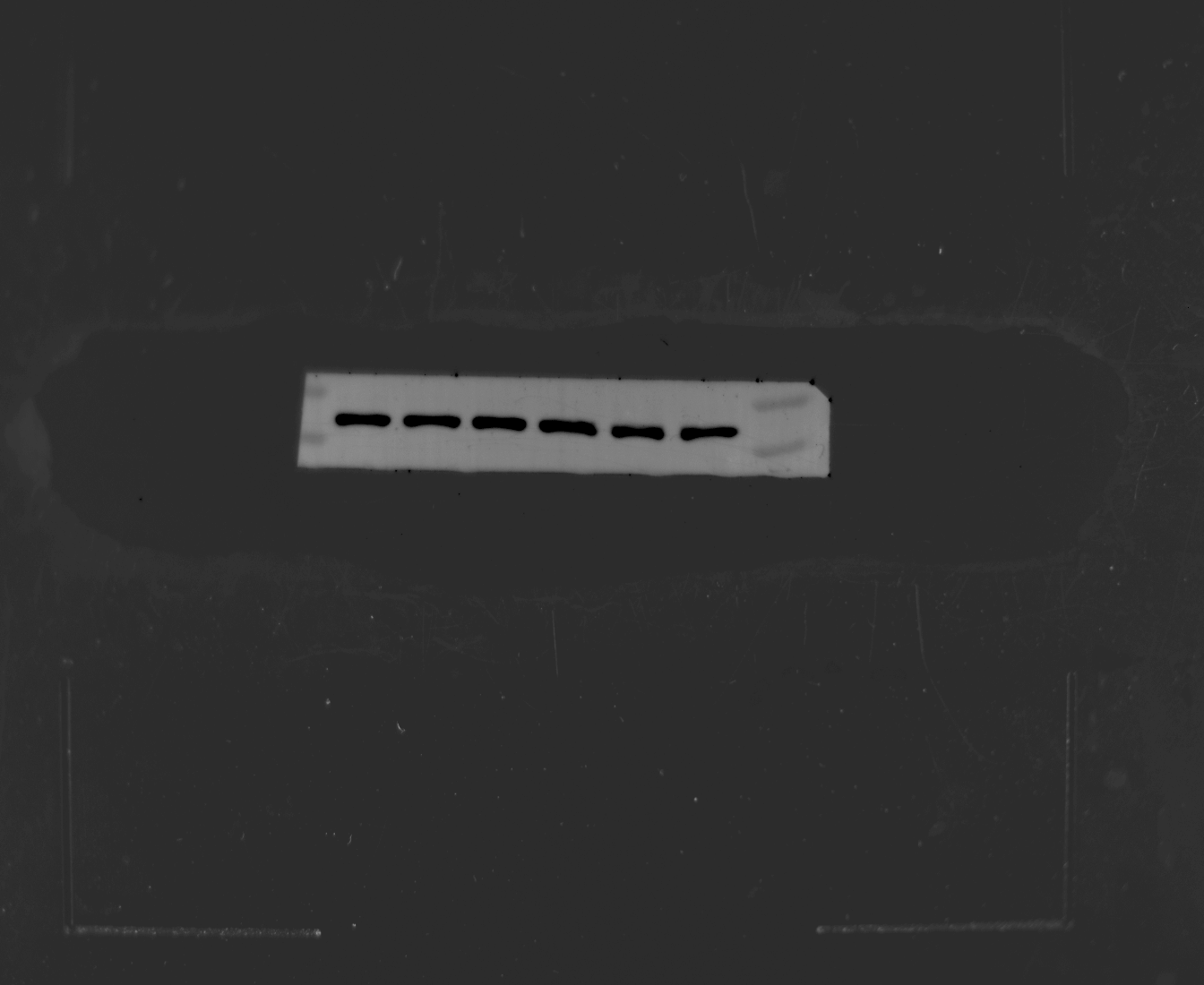

Supplement: Supplementary file 1 — Supplementary file1 (ZIP 36116 KB) [file 432_2024_5625_MOESM1_ESM.zip › Original Images for BlotsGels/4.Figure 4/U87/2.c-il1/2/4-2-A.tif]

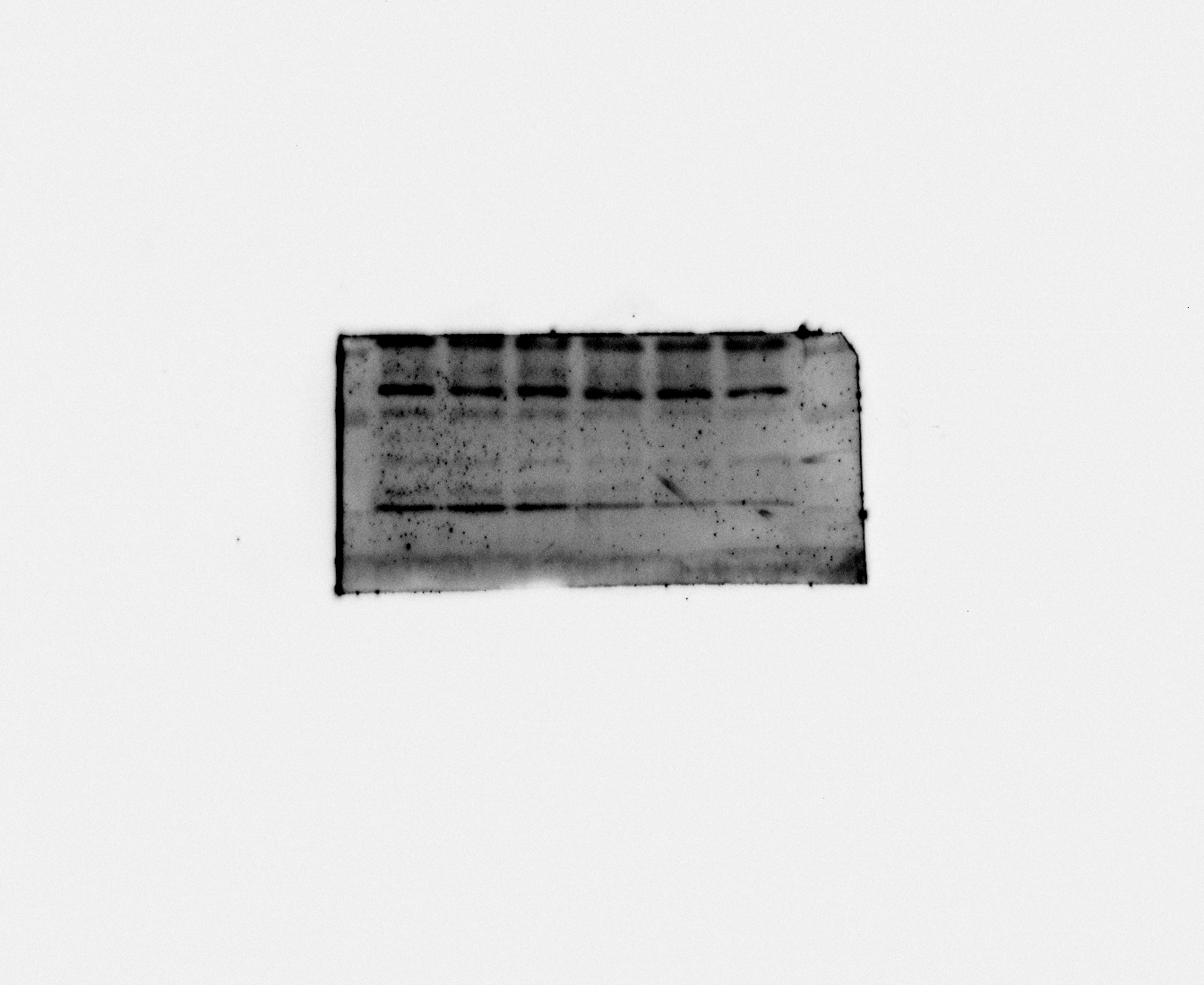

Supplement: Supplementary file 1 — Supplementary file1 (ZIP 36116 KB) [file 432_2024_5625_MOESM1_ESM.zip › Original Images for BlotsGels/4.Figure 4/U87/2.c-il1/2/4-2-IL1(Y).tif]

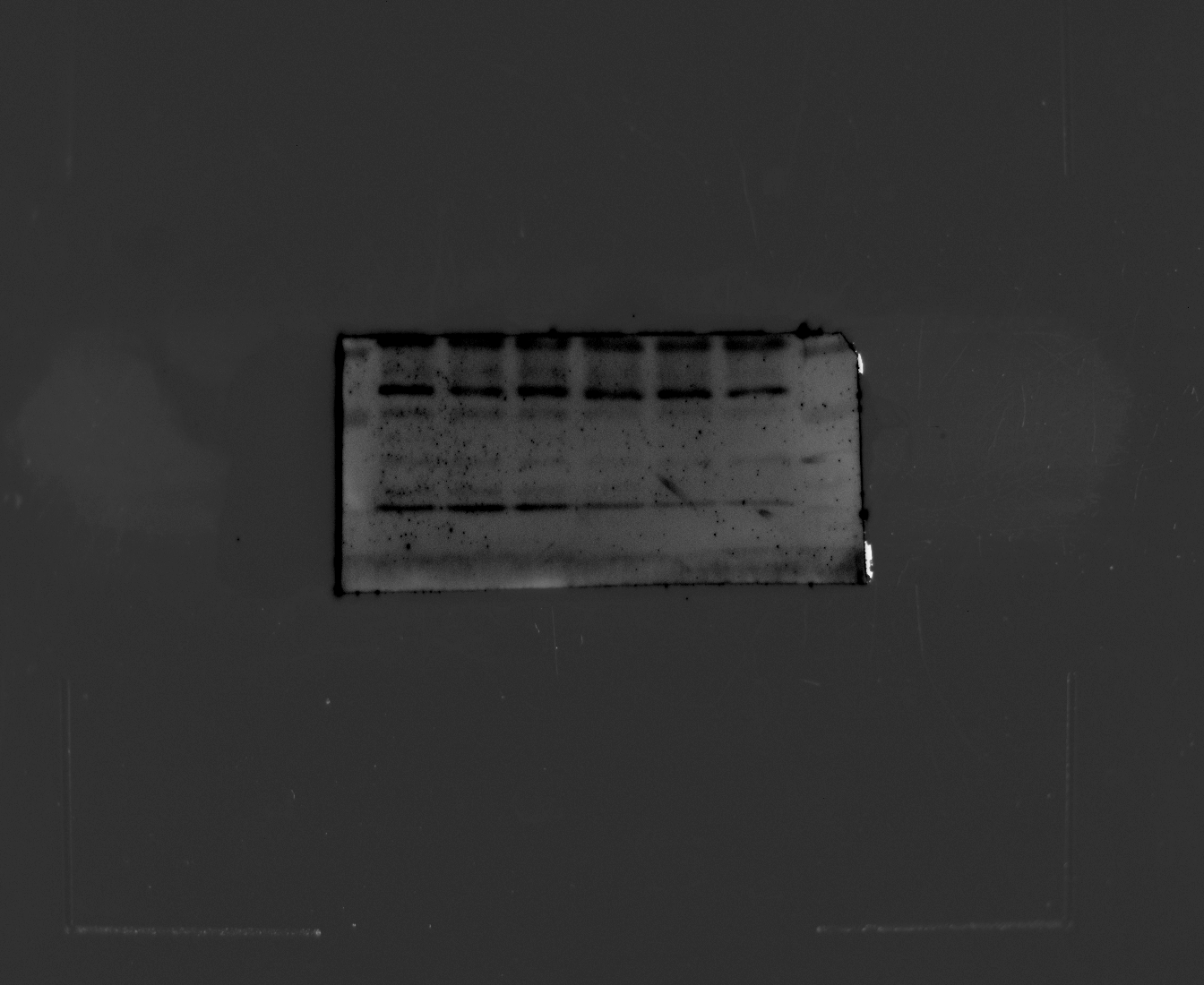

Supplement: Supplementary file 1 — Supplementary file1 (ZIP 36116 KB) [file 432_2024_5625_MOESM1_ESM.zip › Original Images for BlotsGels/4.Figure 4/U87/2.c-il1/2/4-2-IL1.tif]

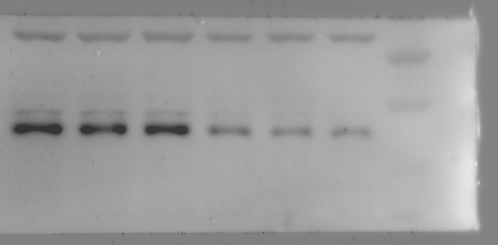

Supplement: Supplementary file 1 — Supplementary file1 (ZIP 36116 KB) [file 432_2024_5625_MOESM1_ESM.zip › Original Images for BlotsGels/4.Figure 4/U87/3.IL18/1/1.IL-18(叠加图).tif]

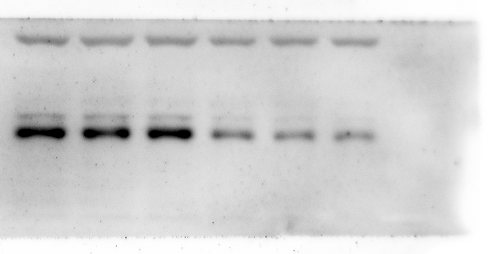

Supplement: Supplementary file 1 — Supplementary file1 (ZIP 36116 KB) [file 432_2024_5625_MOESM1_ESM.zip › Original Images for BlotsGels/4.Figure 4/U87/3.IL18/1/1.IL-18(样品图).tif]

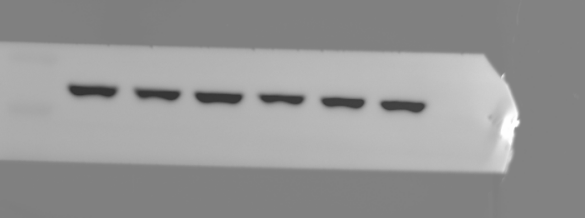

Supplement: Supplementary file 1 — Supplementary file1 (ZIP 36116 KB) [file 432_2024_5625_MOESM1_ESM.zip › Original Images for BlotsGels/4.Figure 4/U87/3.IL18/1/1.β-actin(叠加图).tif]

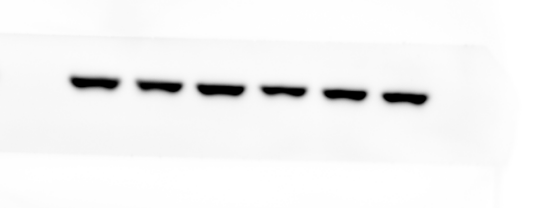

Supplement: Supplementary file 1 — Supplementary file1 (ZIP 36116 KB) [file 432_2024_5625_MOESM1_ESM.zip › Original Images for BlotsGels/4.Figure 4/U87/3.IL18/1/1.β-actin(样品图).tif]

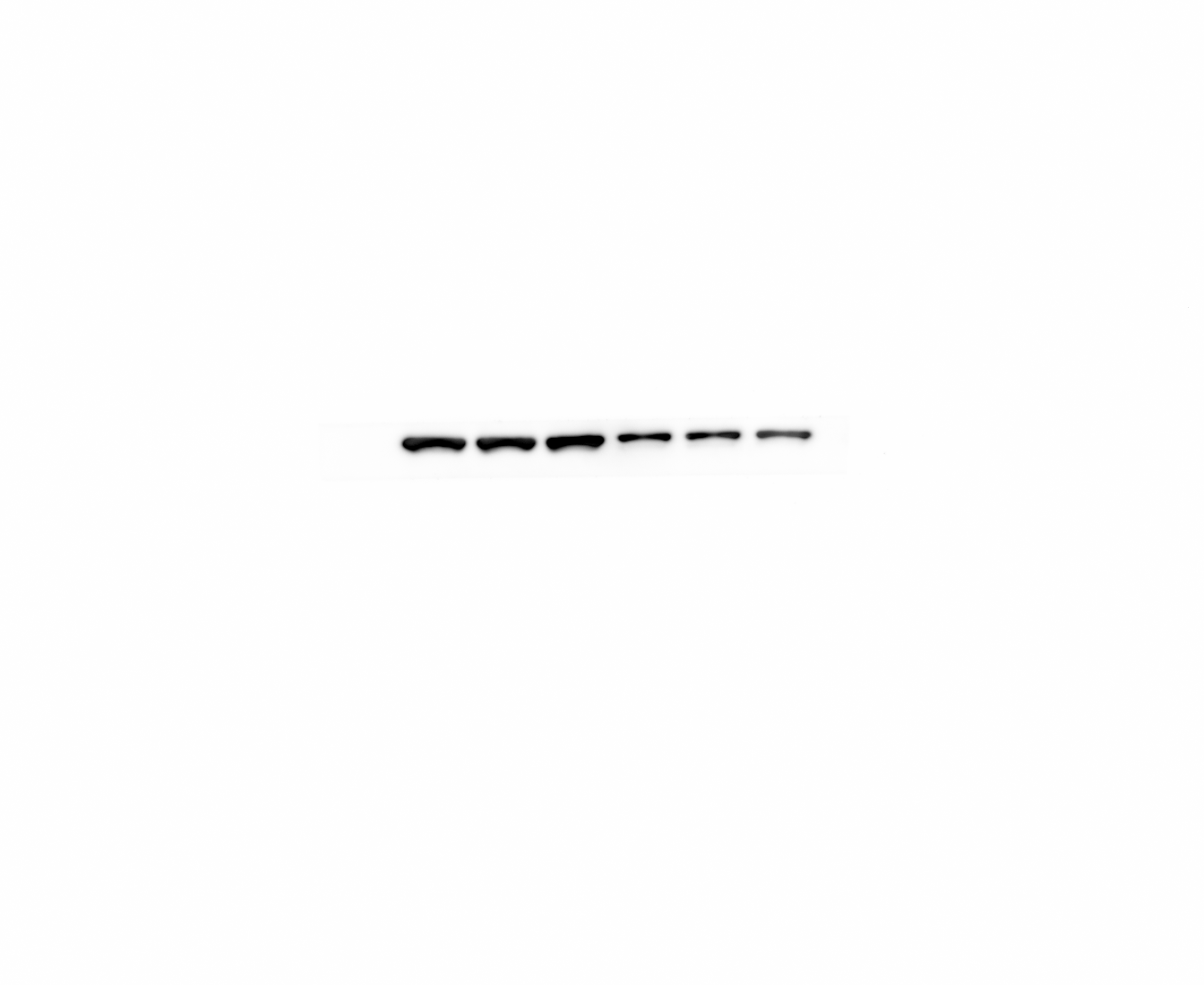

Supplement: Supplementary file 1 — Supplementary file1 (ZIP 36116 KB) [file 432_2024_5625_MOESM1_ESM.zip › Original Images for BlotsGels/4.Figure 4/U87/4.IL6/1/4-1-IL6(Y).tif]

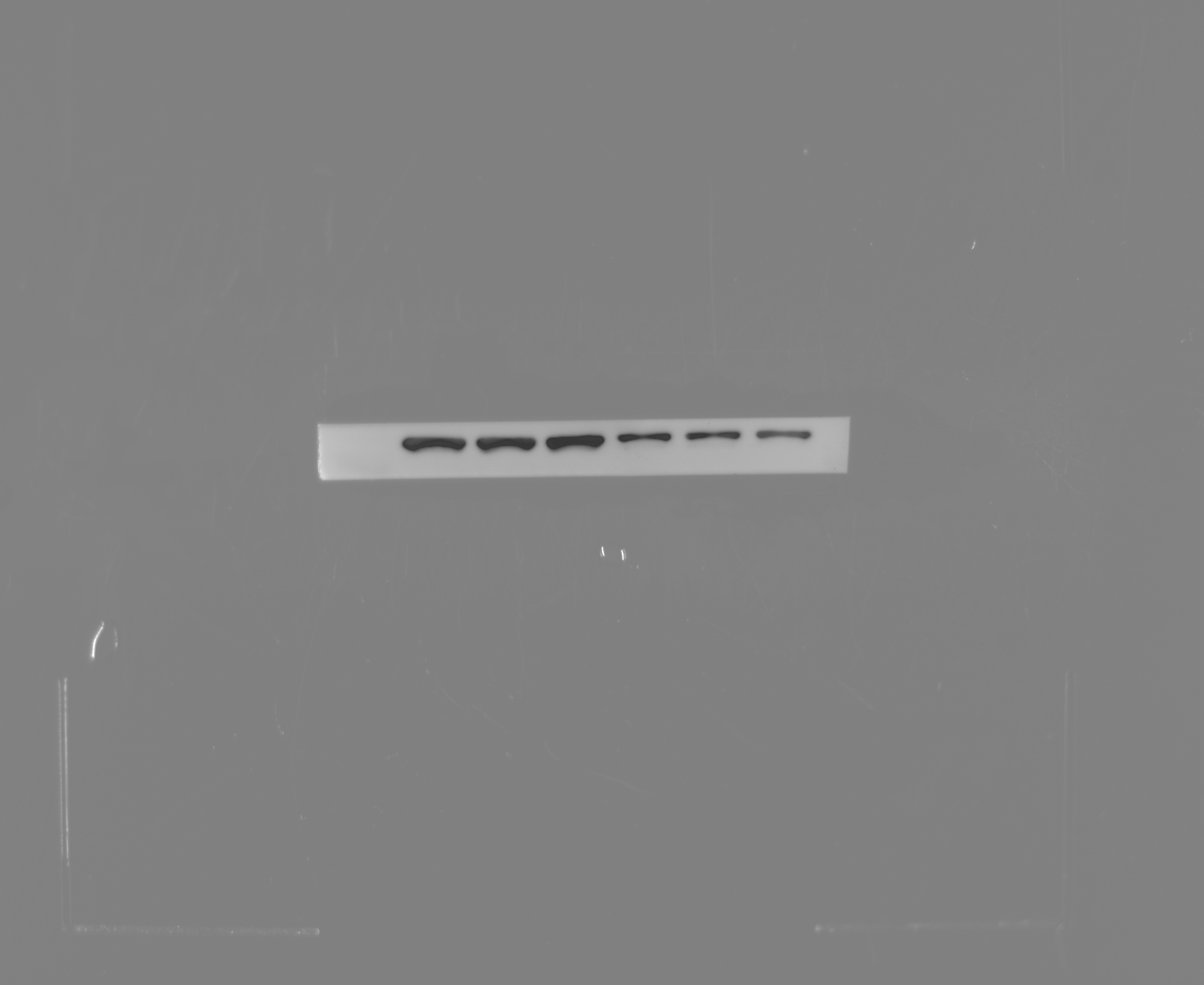

Supplement: Supplementary file 1 — Supplementary file1 (ZIP 36116 KB) [file 432_2024_5625_MOESM1_ESM.zip › Original Images for BlotsGels/4.Figure 4/U87/4.IL6/1/4-1-IL6.tif]

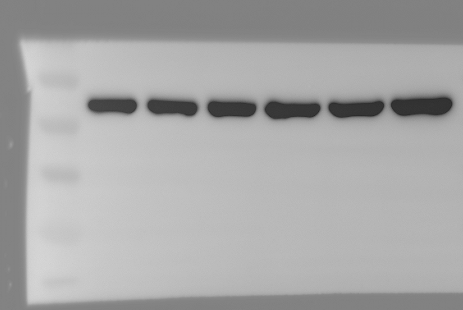

Supplement: Supplementary file 1 — Supplementary file1 (ZIP 36116 KB) [file 432_2024_5625_MOESM1_ESM.zip › Original Images for BlotsGels/4.Figure 4/U87/4.IL6/1/4-1-β-anctin(叠加图).tif]

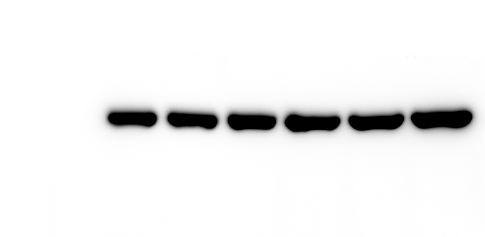

Supplement: Supplementary file 1 — Supplementary file1 (ZIP 36116 KB) [file 432_2024_5625_MOESM1_ESM.zip › Original Images for BlotsGels/4.Figure 4/U87/4.IL6/1/4-1-β-anctin(样品图).tif]

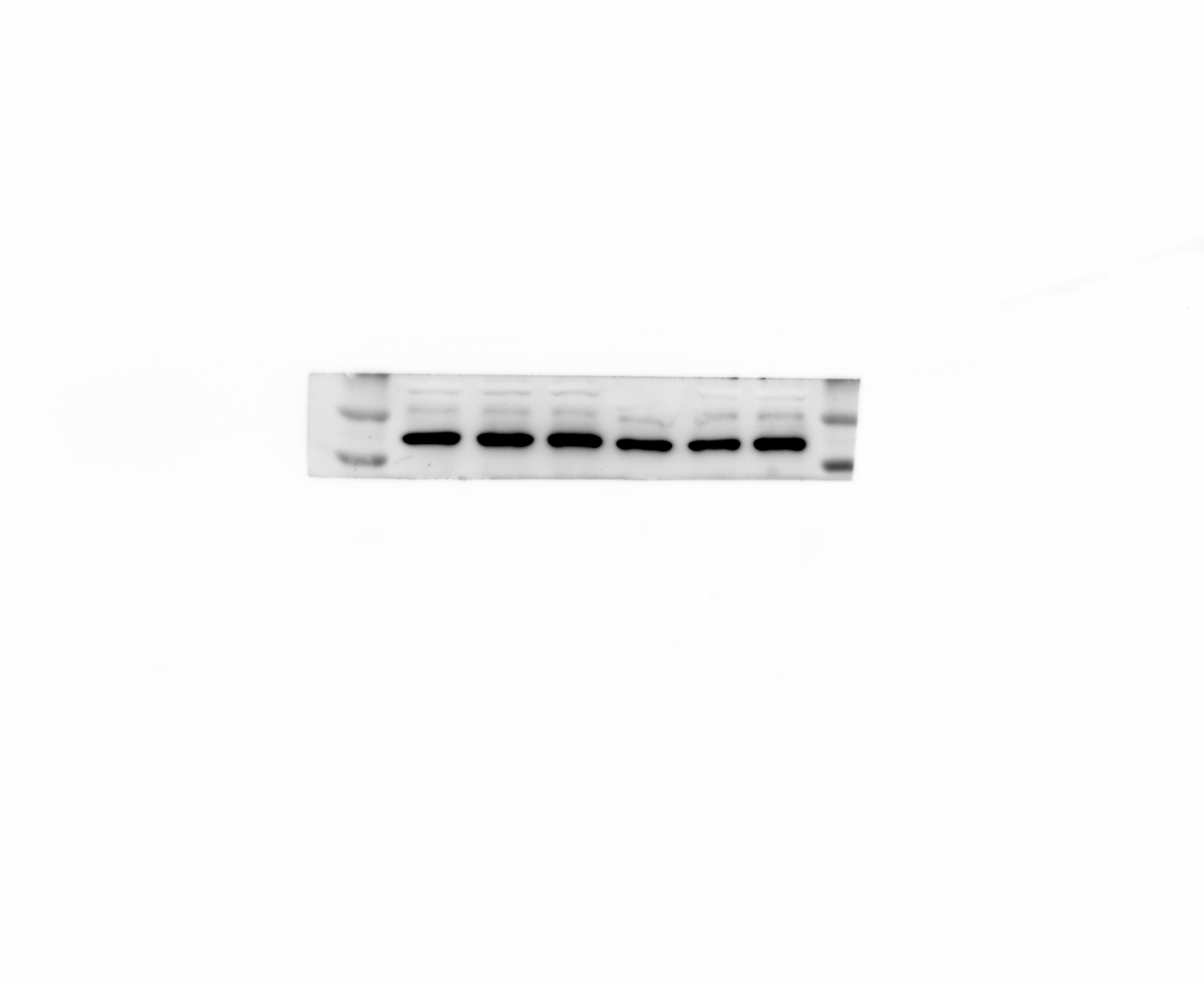

Supplement: Supplementary file 1 — Supplementary file1 (ZIP 36116 KB) [file 432_2024_5625_MOESM1_ESM.zip › Original Images for BlotsGels/4.Figure 4/U87/5.TNF/5-2-A(Y).tif]

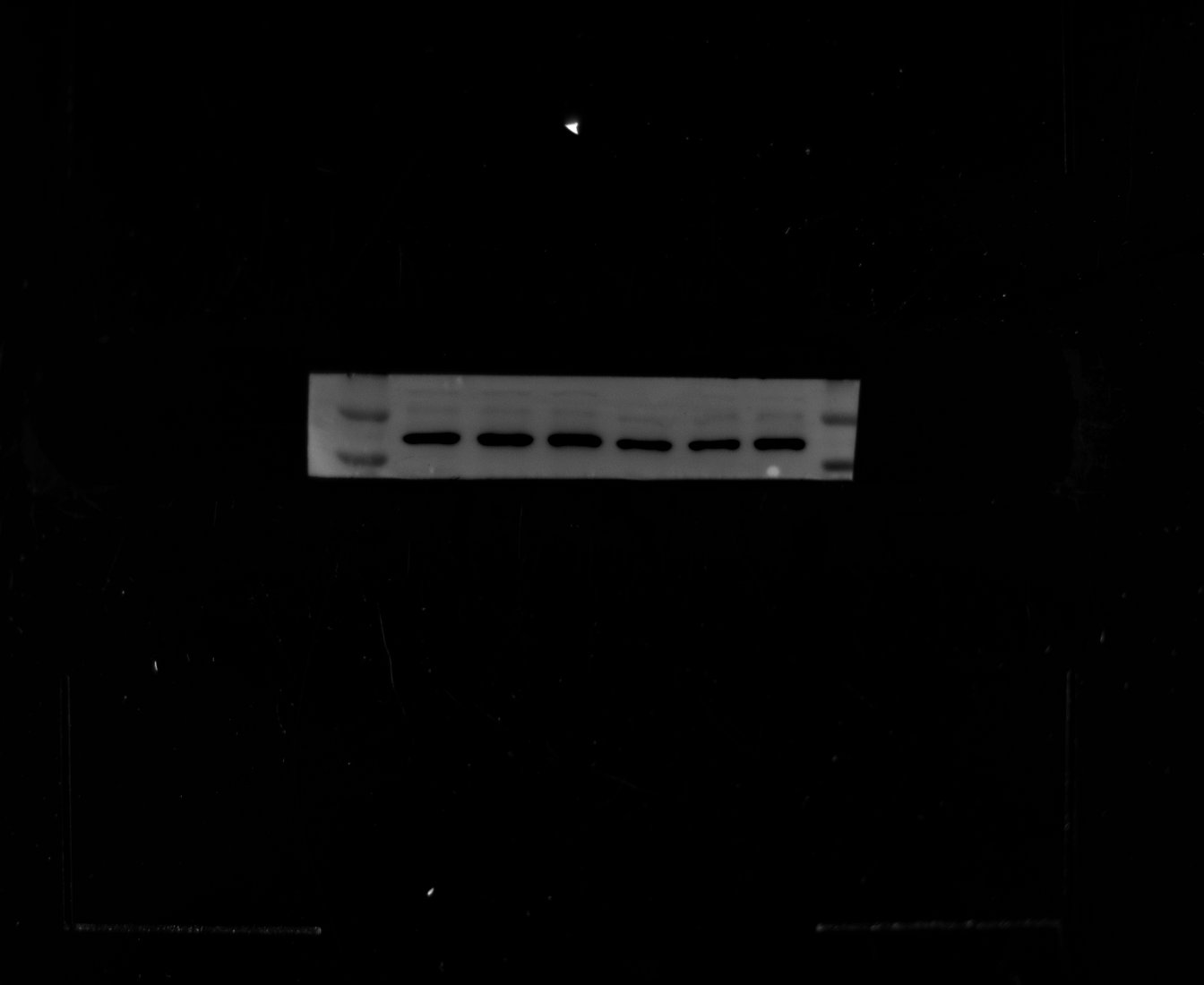

Supplement: Supplementary file 1 — Supplementary file1 (ZIP 36116 KB) [file 432_2024_5625_MOESM1_ESM.zip › Original Images for BlotsGels/4.Figure 4/U87/5.TNF/5-2-A.tif]

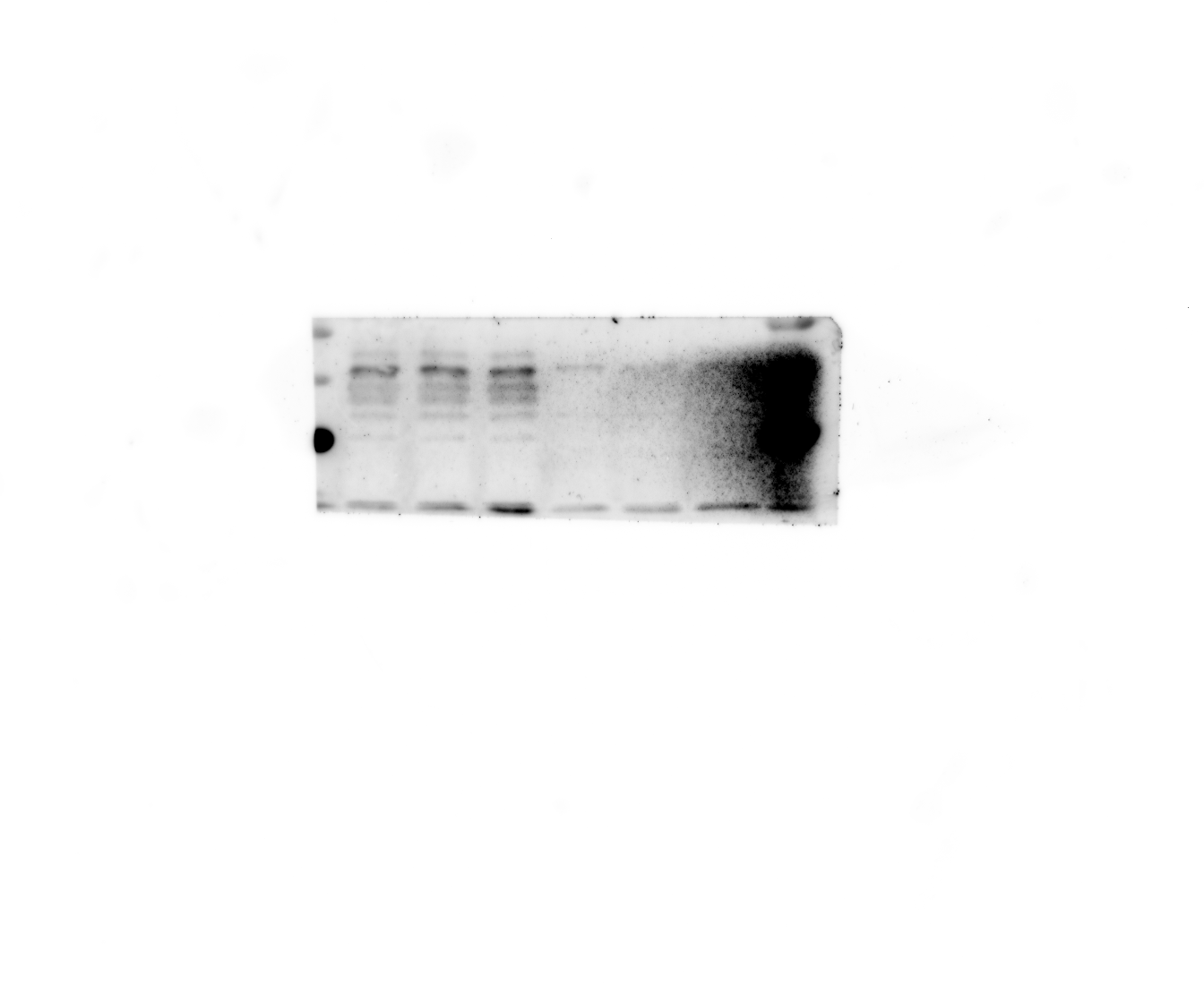

Supplement: Supplementary file 1 — Supplementary file1 (ZIP 36116 KB) [file 432_2024_5625_MOESM1_ESM.zip › Original Images for BlotsGels/4.Figure 4/U87/5.TNF/5-2-TNF(Y).tif]

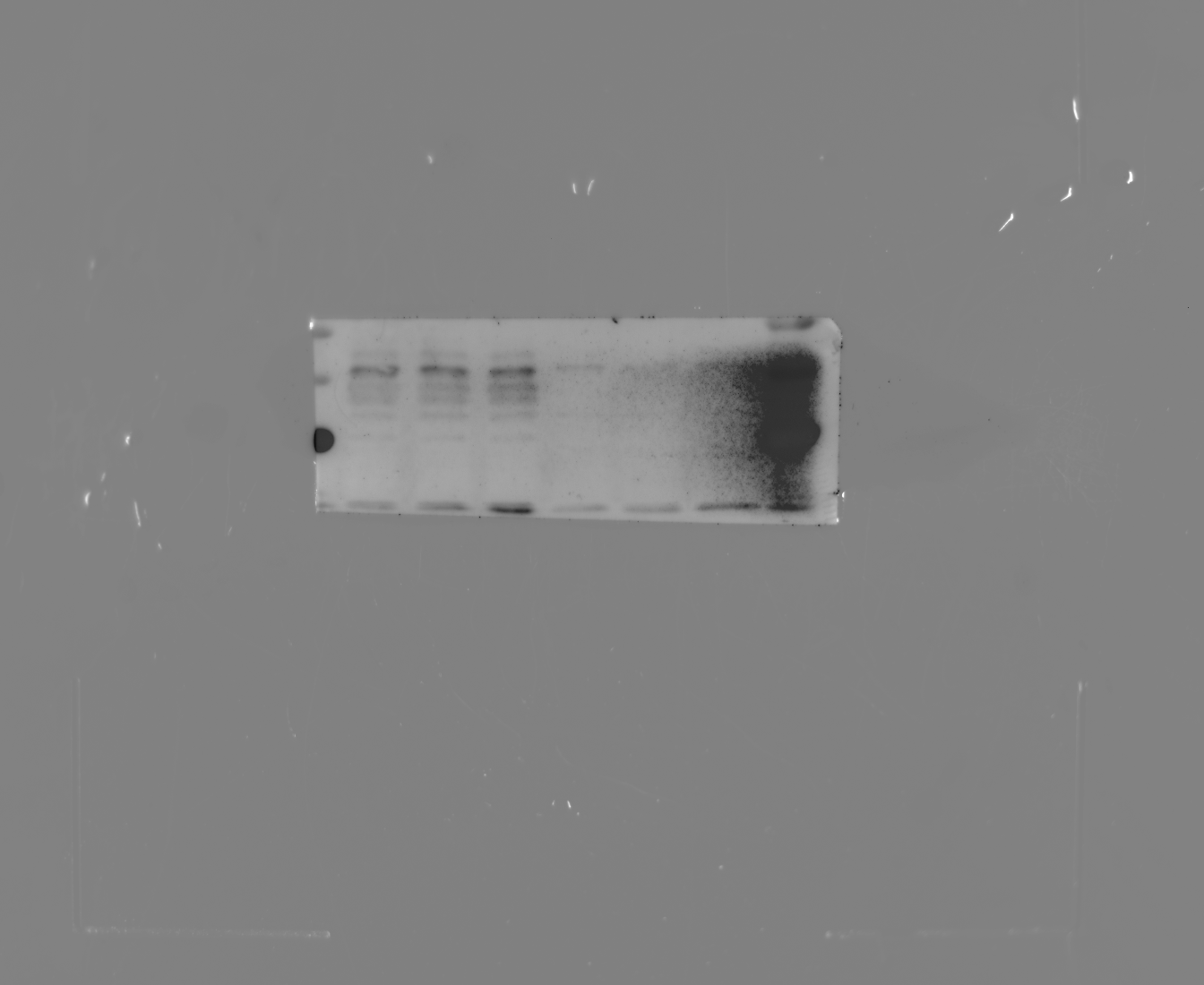

Supplement: Supplementary file 1 — Supplementary file1 (ZIP 36116 KB) [file 432_2024_5625_MOESM1_ESM.zip › Original Images for BlotsGels/4.Figure 4/U87/5.TNF/5-2-TNF.tif]

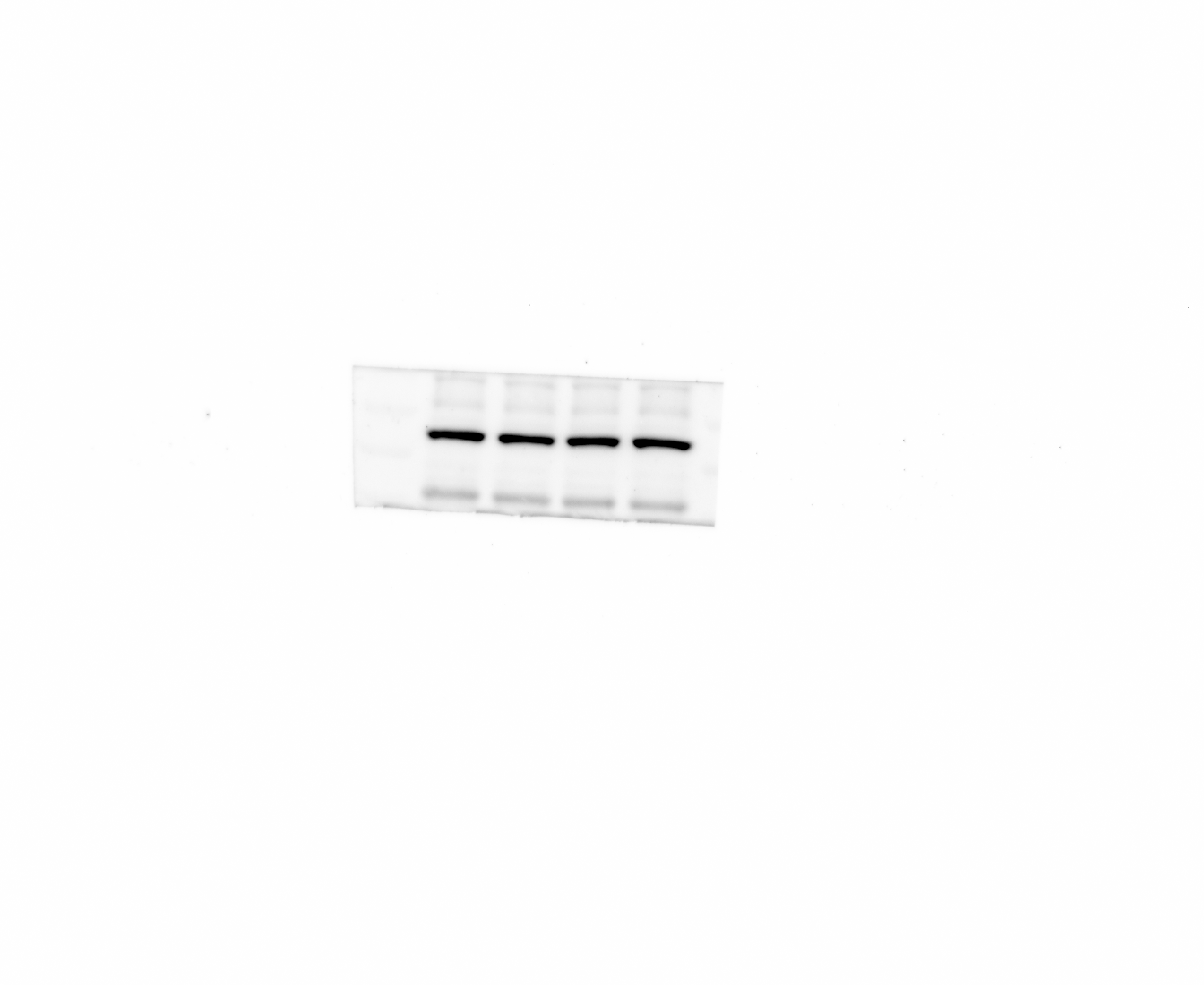

Supplement: Supplementary file 1 — Supplementary file1 (ZIP 36116 KB) [file 432_2024_5625_MOESM1_ESM.zip › Original Images for BlotsGels/5.Figure 5/LN229/1.JAK2/1/3-1-A(Y).tif]

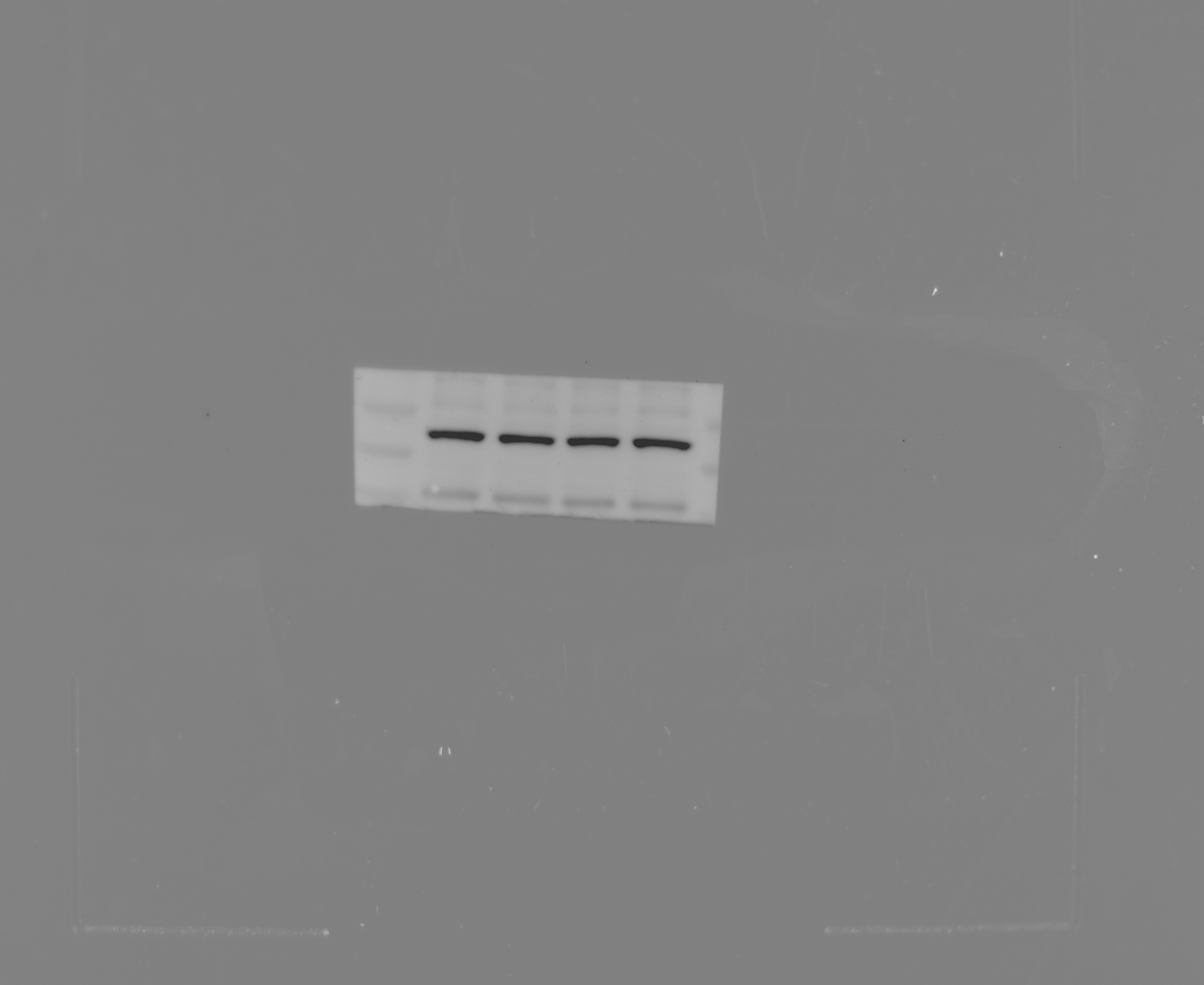

Supplement: Supplementary file 1 — Supplementary file1 (ZIP 36116 KB) [file 432_2024_5625_MOESM1_ESM.zip › Original Images for BlotsGels/5.Figure 5/LN229/1.JAK2/1/3-1-A.tif]

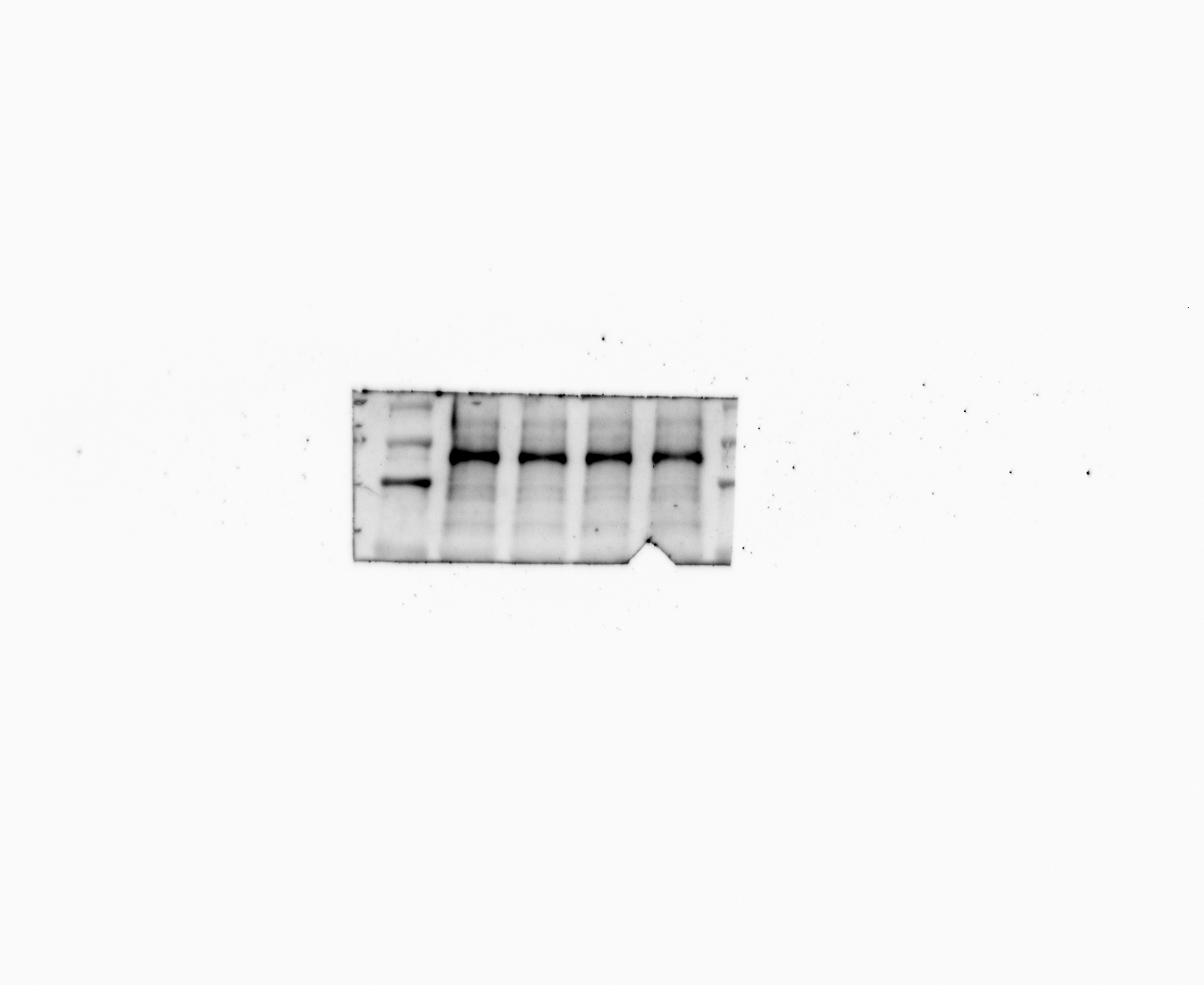

Supplement: Supplementary file 1 — Supplementary file1 (ZIP 36116 KB) [file 432_2024_5625_MOESM1_ESM.zip › Original Images for BlotsGels/5.Figure 5/LN229/1.JAK2/1/3-1-JAK2(Y).tif]

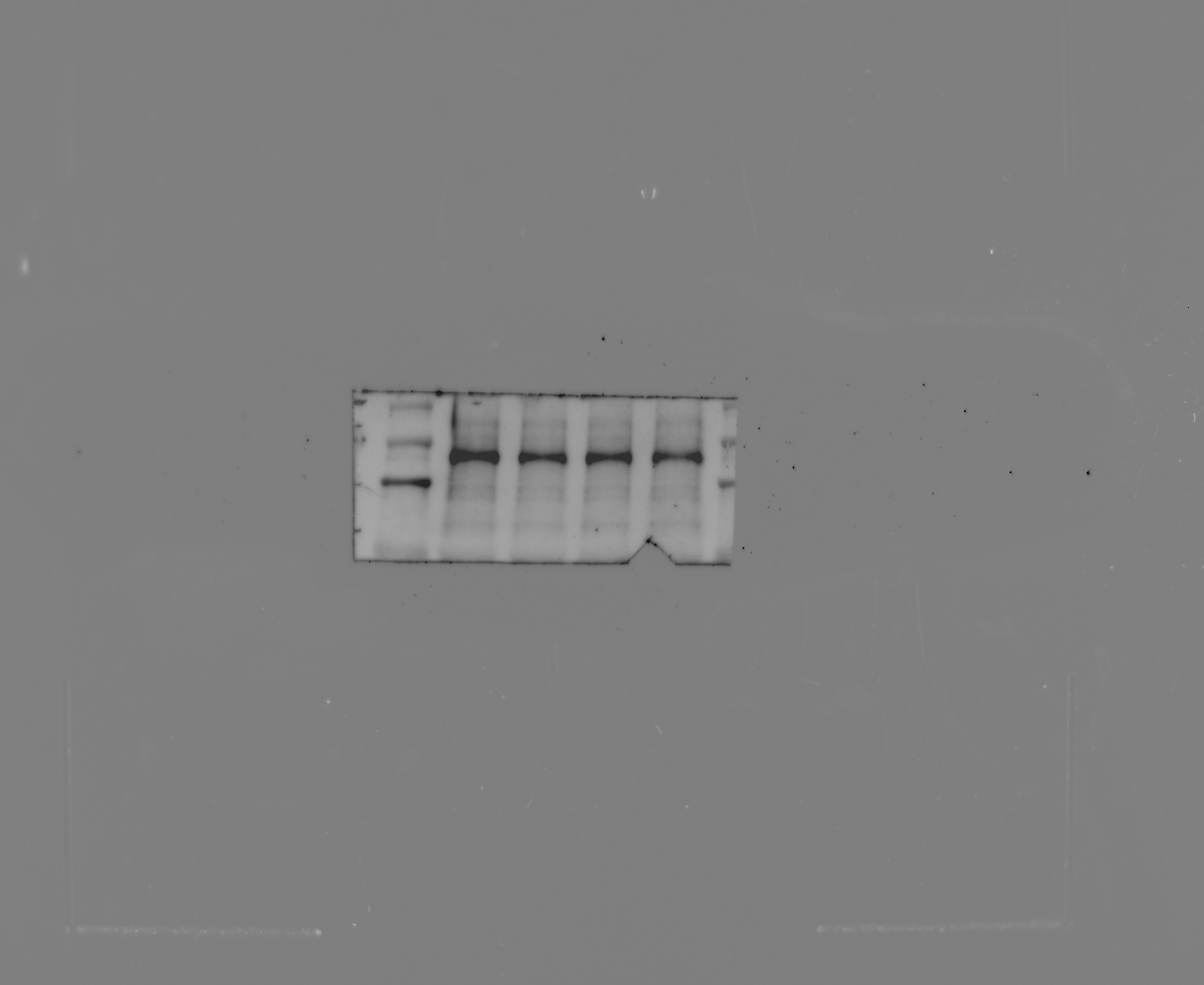

Supplement: Supplementary file 1 — Supplementary file1 (ZIP 36116 KB) [file 432_2024_5625_MOESM1_ESM.zip › Original Images for BlotsGels/5.Figure 5/LN229/1.JAK2/1/3-1-JAK2.tif]

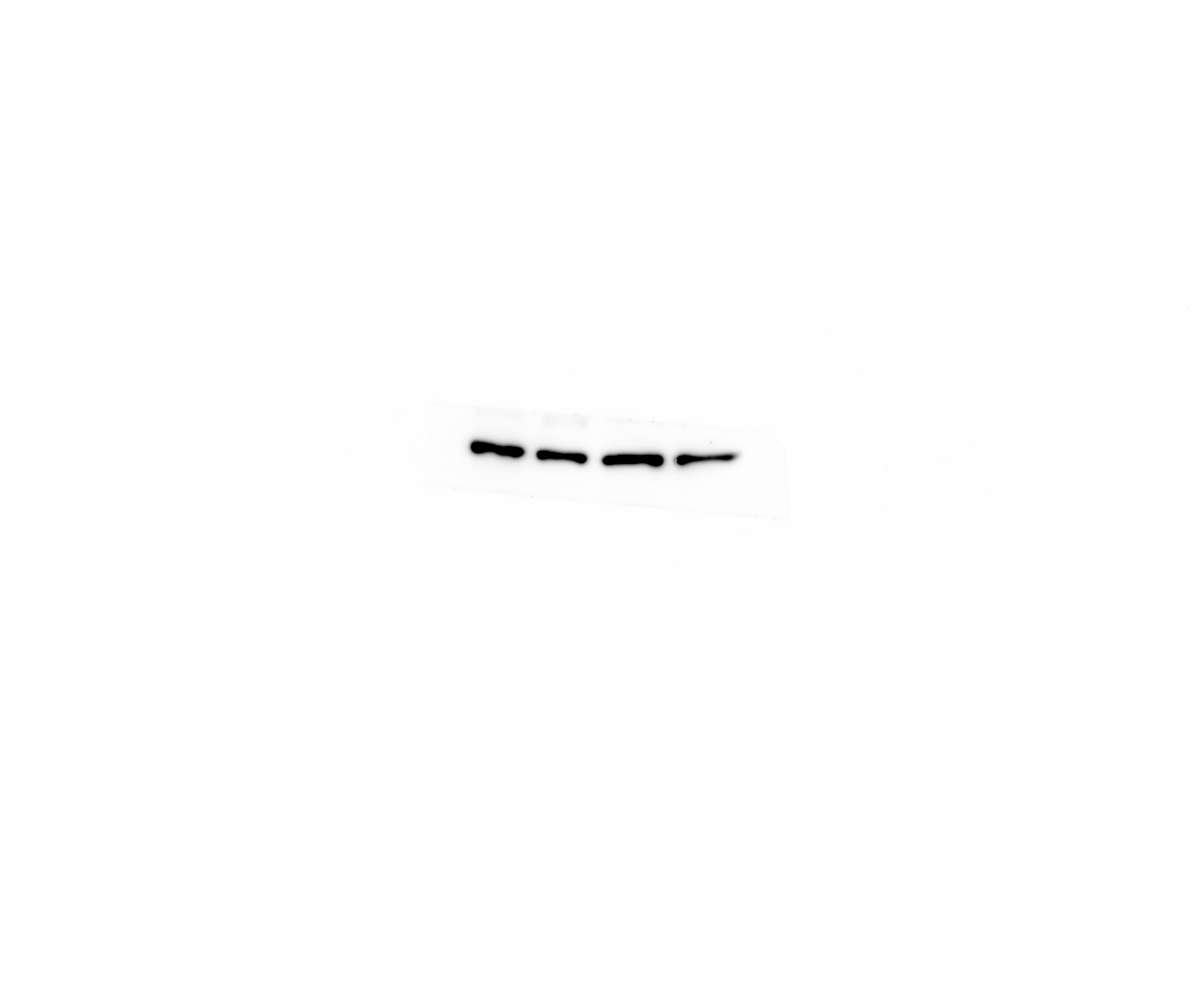

Supplement: Supplementary file 1 — Supplementary file1 (ZIP 36116 KB) [file 432_2024_5625_MOESM1_ESM.zip › Original Images for BlotsGels/5.Figure 5/LN229/2.P-JAK2/1/1-3-A(Y).tif]

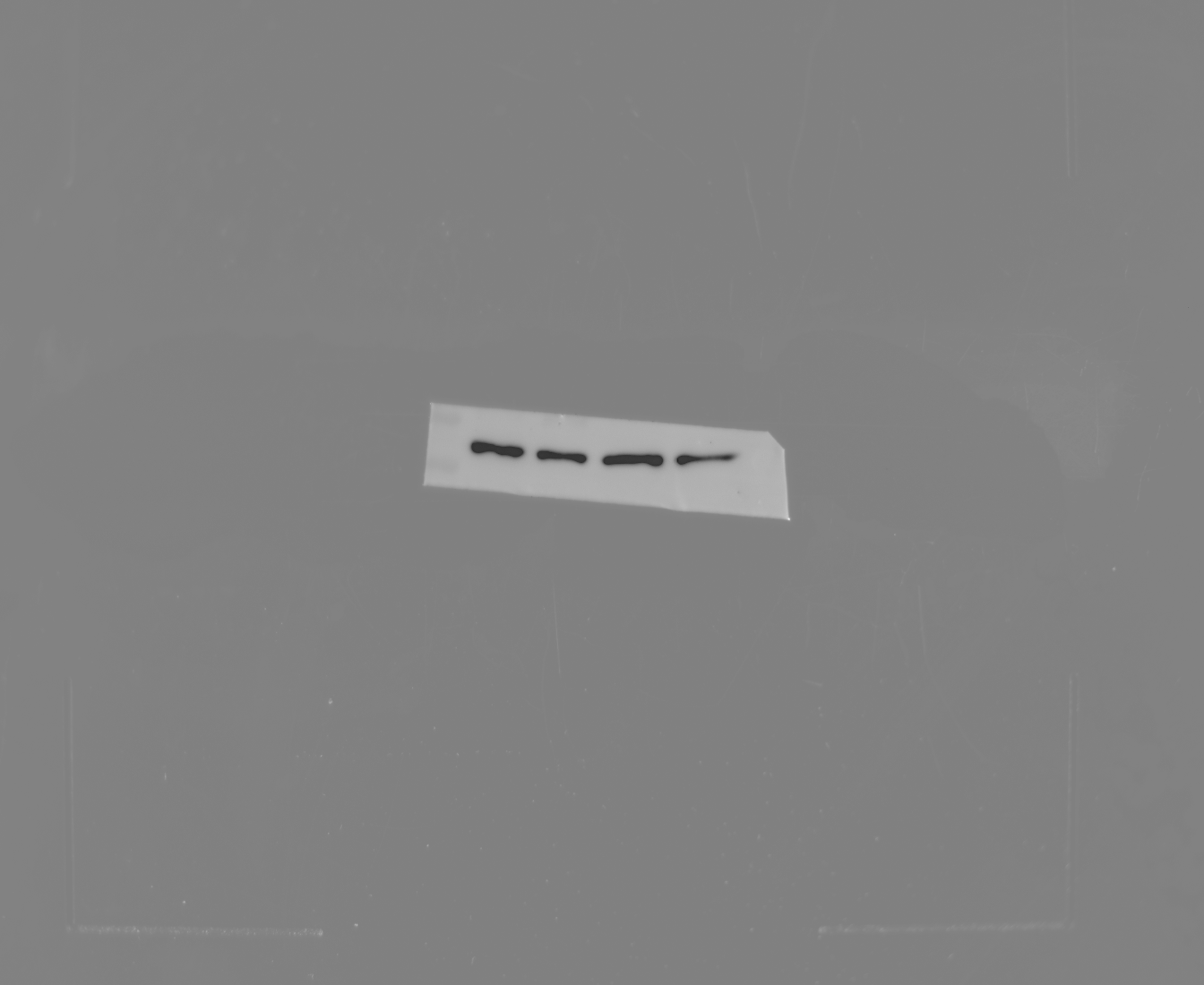

Supplement: Supplementary file 1 — Supplementary file1 (ZIP 36116 KB) [file 432_2024_5625_MOESM1_ESM.zip › Original Images for BlotsGels/5.Figure 5/LN229/2.P-JAK2/1/1-3-A.tif]
